# Supplementary material for: Synthesis of Pyrrolidine Monocyclic Analogues of Pochonicine and Its Stereoisomers: Pursuit of Simplified Structures and Potent β-N-Acetylhexosaminidase Inhibition
Source: Molecules. 2020 Mar 25;25(7):1498. doi: 10.3390/molecules25071498 (PMC7180638; doi:10.3390/molecules25071498)
Supplement: Supplementary file 1 [file molecules-25-01498-s001.pdf]

Synthesis of Pyrrolidine Monocyclic Analogues of Pochonicine and Its Stereoisomers: Pursuit of Simplified Structures and Potent β-*N*-Acetylhexosaminidase Inhibition

Xin Yan [1,2], Yuna Shimadate [3], Atsushi Kato [3,*], Yi-Xian Li [1,2,*], Yue-Mei Jia [1,2], George W. J. Fleet [4,5] and Chu-Yi Yu [1,2,5,*]

[1] Beijing National Laboratory for Molecular Science (BNLMS), CAS Key Laboratory of Molecular Recognition and Function, Institute of Chemistry, Chinese Academy of Sciences, Beijing 100190, China; yanxin2012@iccas.ac.cn (X.Y.); jiayuemei@iccas.ac.cn (Y.-M.J.)

[2] University of Chinese Academy of Sciences, Beijing 100049, China

[3] Department of Hospital Pharmacy, University of Toyama, 2630 Sugitani, Toyama 930-0194, Japan; m1961224@ems.u-toyama.ac.jp

[4] Chemistry Research Laboratory, Department of Chemistry, University of Oxford, Mansfield Road, Oxford, OX13TA, UK; george.fleet@chem.ox.ac.uk

[5] National Engineering Research Center for Carbohydrate Synthesis, Jiangxi Normal University, Nanchang 330022, PR China

* Correspondence: kato@med.u-toyama.ac.jp (A.K.); tamarali@iccas.ac.cn (Y.-X.L.); yucy@iccas.ac.cn (C.-Y.Y.); Tel.: +86-010-626-12893 (C.-Y.Y.)

Supporting Information

Contents

1. Copies of NMR spectra..2
2. X-Ray Crystallographic data for compound **D-2**, **F-2b** and **H-2**......................................96

1. Copies of NMR spectra

Compound **A-2:**

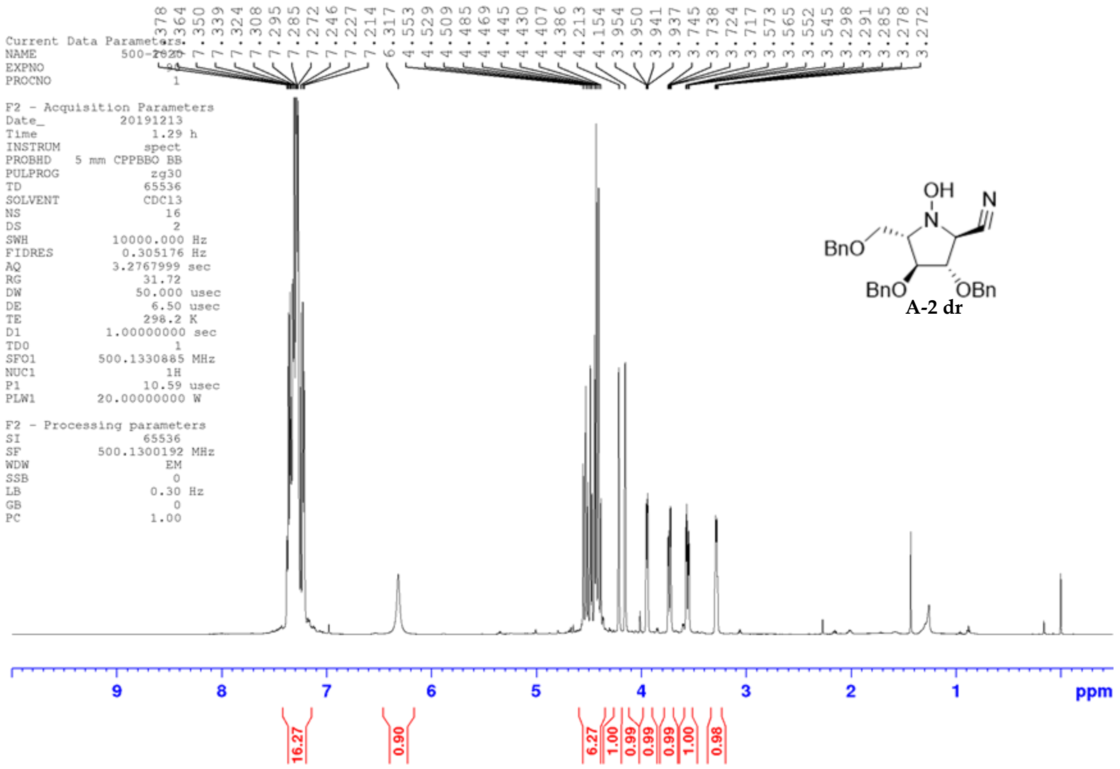

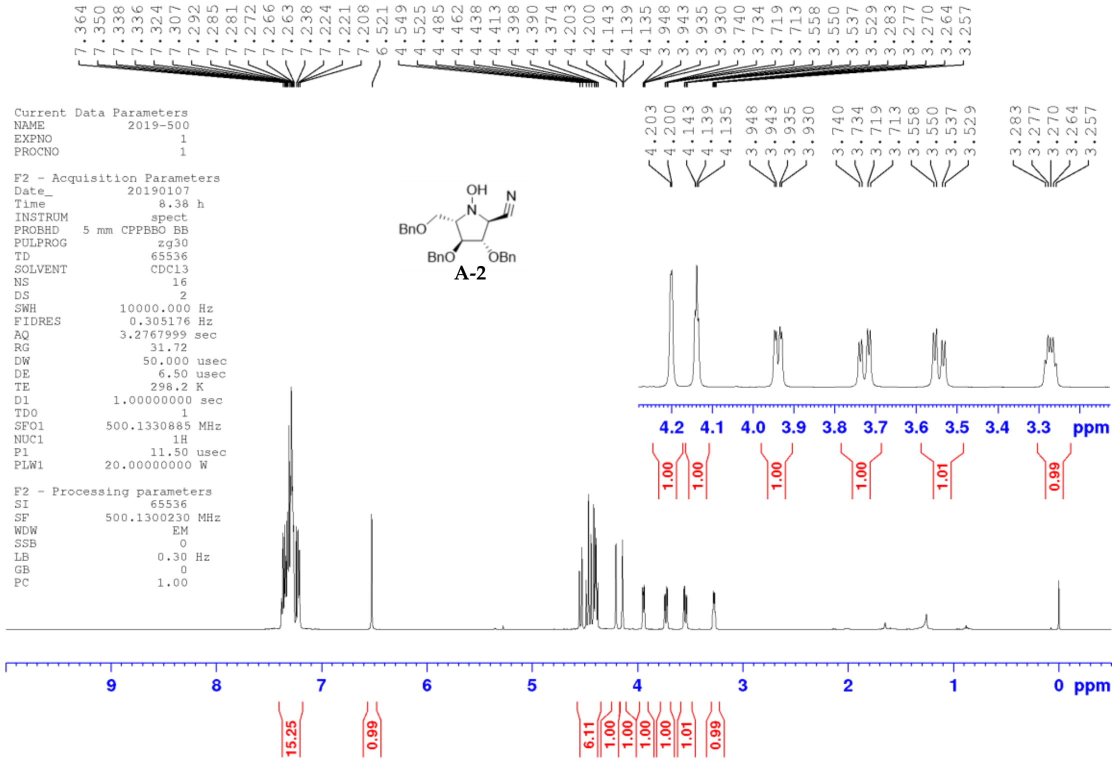

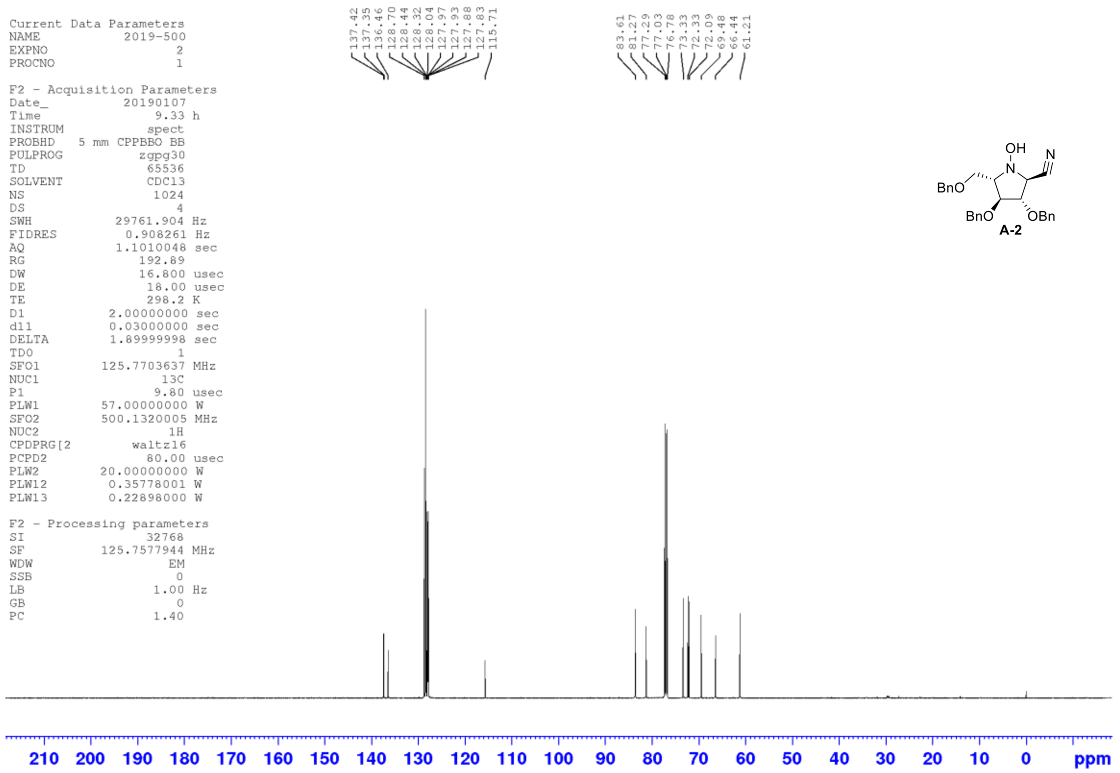
Current Data Parameters
NAME 2019-500
EXPNO 2
PROCNO 1
F2 - Acquisition Parameters
Date_ 20190107
Time 9.33 h
INSTRUM spect
PROBHD 5 mm CPPBBO BB
PULPROG zgpg30
TD 65536
SOLVENT CDCl3
NS 1024
DS 4
SWH 29761.904 Hz
FIDRES 0.908261 Hz
AQ 1.1010048 sec
RG 192.89
DW 16.800 usec
DE 18.00 usec
TE 298.2 K
D1 2.00000000 sec
d11 0.03000000 sec
DELTA 1.89999998 sec
TD0 1
SFO1 125.7703637 MHz
NUC1 13C
P1 9.80 usec
PLW1 57.00000000 W
SFO2 500.1320005 MHz
NUC2 1H
CPDPRG[2 waltz16
PCPD2 80.00 usec
PLW2 20.00000000 W
PLW12 0.35778001 W
PLW13 0.22898000 W
F2 - Processing parameters
SI 32768
SF 125.7577944 MHz
WDW EM
SSB 0
LB 1.00 Hz
GB 0
PC 1.40
137.42
137.35
136.46
128.70
128.44
128.32
128.04
127.97
127.93
127.88
127.83
115.71
83.61
81.27
77.29
77.03
76.78
73.33
72.33
72.09
69.48
66.44
61.21
OH
N
N
BnO
BnO
OBn
A-2
210 200 190 180 170 160 150 140 130 120 110 100 90 80 70 60 50 40 30 20 10 0 ppm

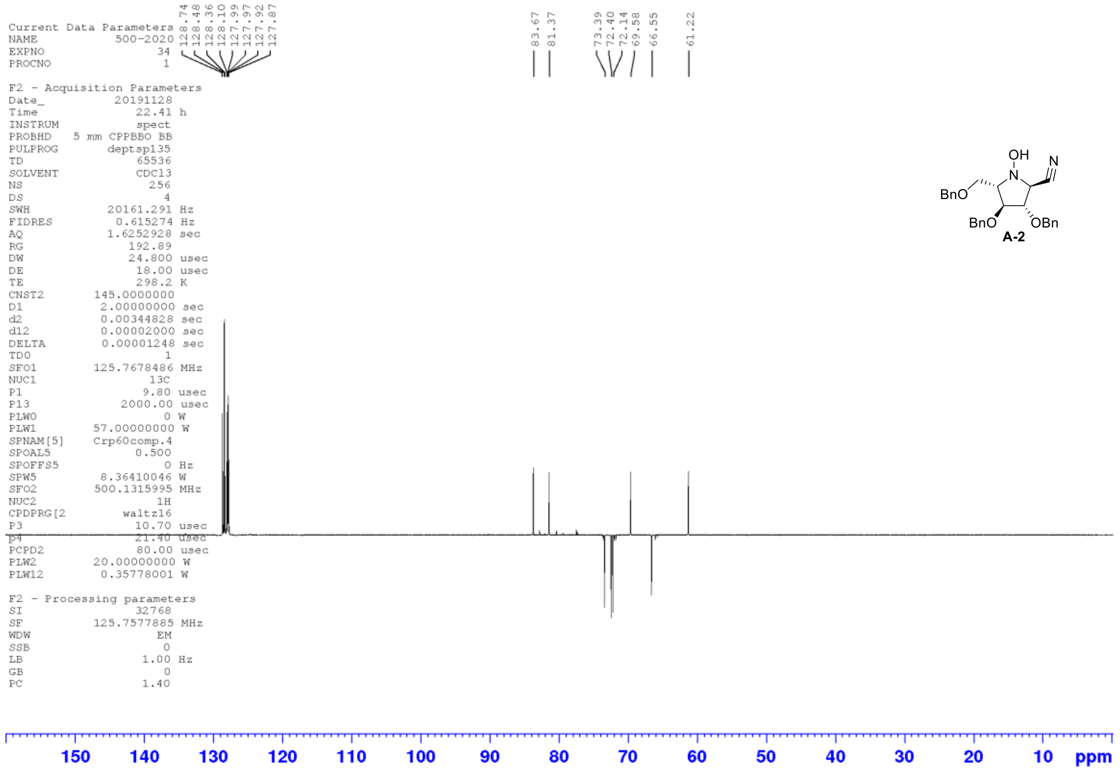
Current Data Parameters
NAME 500-2020
EXPNO 34
PROCNO 1
F2 - Acquisition Parameters
Date_ 20191128
Time 22.41 h
INSTRUM spect
PROBHD 5 mm CPPBBO BB
PULPROG deptsp135
TD 65536
SOLVENT CDCl3
NS 256
DS 4
SWH 20161.291 Hz
FIDRES 0.615274 Hz
AQ 1.6252928 sec
RG 192.89
DW 24.800 usec
DE 18.00 usec
TE 298.2 K
CNST2 145.0000000
D1 2.00000000 sec
d2 0.00344828 sec
d12 0.00002000 sec
DELTA 0.00001248 sec
TD0 1
SFO1 125.7678486 MHz
NUC1 13C
P1 9.80 usec
P13 2000.00 usec
PLW0 0 W
PLW1 57.00000000 W
SPNAM[5] Crp60comp.4
SPOAL5 0.500
SPOFFS5 0 Hz
SPW5 8.36410046 W
SFO2 500.1315995 MHz
NUC2 1H
CPDPRG[2 waltz16
P3 10.70 usec
p4 21.40 usec
PCPD2 80.00 usec
PLW2 20.00000000 W
PLW12 0.35778001 W
F2 - Processing parameters
SI 32768
SF 125.7577885 MHz
WDW EM
SSB 0
LB 1.00 Hz
GB 0
PC 1.40
128.74
128.48
128.36
128.10
127.99
127.97
127.92
127.87
83.67
81.37
73.39
72.40
72.14
69.58
66.55
61.22
OH
N
N
BnO
BnO
OBn
A-2
150 140 130 120 110 100 90 80 70 60 50 40 30 20 10 ppm

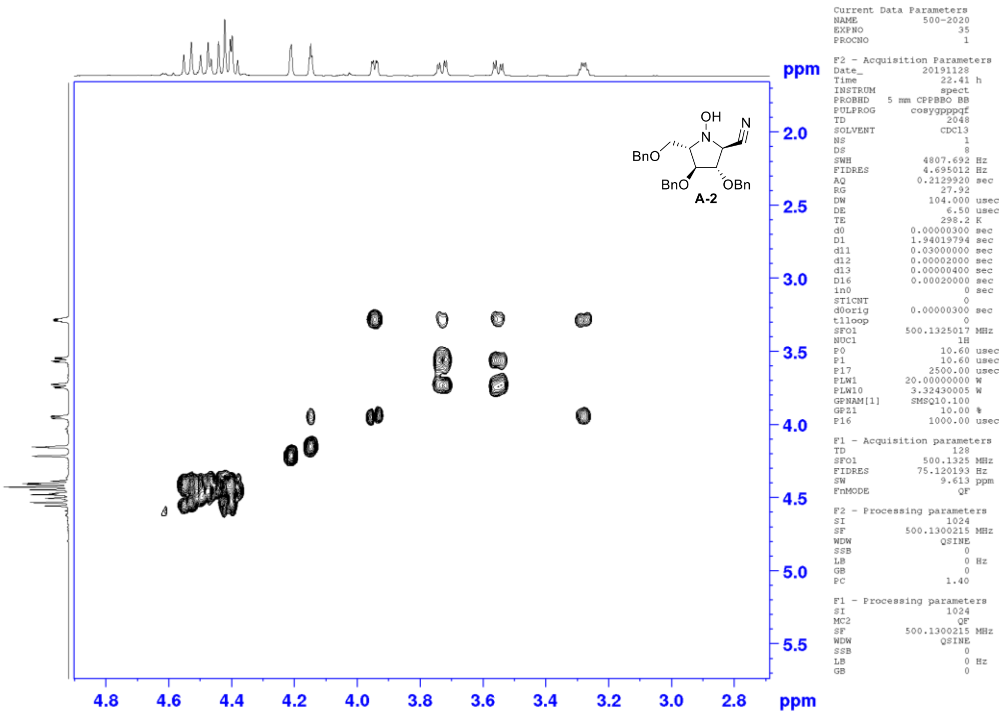
OH
N
BnO
BnO
OBn
A-2
ppm
2.0
2.5
3.0
3.5
4.0
4.5
5.0
5.5
4.8 4.6 4.4 4.2 4.0 3.8 3.6 3.4 3.2 3.0 2.8 ppm
Current Data Parameters
NAME 500-2020
EXPNO 35
PROCNO 1
F2 - Acquisition Parameters
Date_ 20191128
Time 22.41 h
INSTRUM spect
PROBHD 5 mm CPPBBO BB
PULPROG cosygpppqf
TD 2048
SOLVENT CDCl3
NS 1
DS 8
SWH 4807.692 Hz
FIDRES 4.695012 Hz
AQ 0.2129920 sec
RG 27.92
DW 104.000 usec
DE 6.50 usec
TE 298.2 K
d0 0.00000300 sec
D1 1.94019794 sec
d11 0.03000000 sec
d12 0.00002000 sec
d13 0.00000400 sec
D16 0.00020000 sec
in0 0 sec
ST1CNT 0
d0orig 0.00000300 sec
t1loop 0
SFO1 500.1325017 MHz
NUC1 1H
P0 10.60 usec
P1 10.60 usec
P17 2500.00 usec
PLW1 20.00000000 W
PLW10 3.32430005 W
GPNAM[1] SMSQ10.100
GPZ1 10.00 %
P16 1000.00 usec
F1 - Acquisition parameters
TD 128
SFO1 500.1325 MHz
FIDRES 75.120193 Hz
SW 9.613 ppm
FnMODE QF
F2 - Processing parameters
SI 1024
SF 500.1300215 MHz
WDW QSINE
SSB 0
LB 0 Hz
GB 0
PC 1.40
F1 - Processing parameters
SI 1024
MC2 QF
SF 500.1300215 MHz
WDW QSINE
SSB 0
LB 0 Hz
GB 0

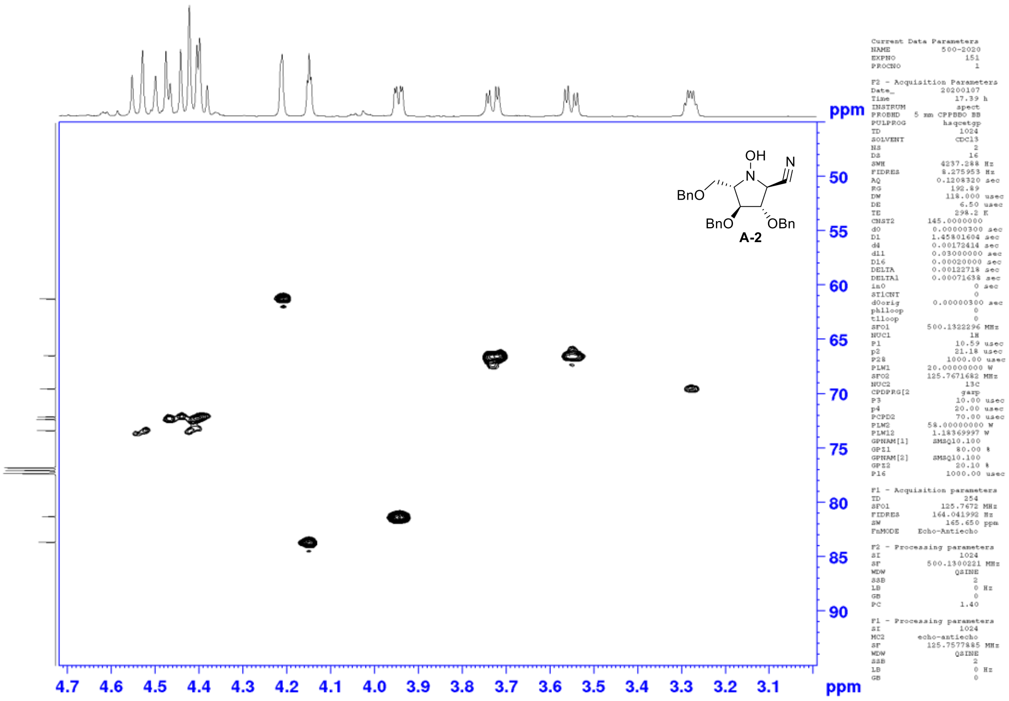
OH
N
BnO
BnO
OBn
A-2
ppm
50
55
60
65
70
75
80
85
90
4.7 4.6 4.5 4.4 4.3 4.2 4.1 4.0 3.9 3.8 3.7 3.6 3.5 3.4 3.3 3.2 3.1 ppm
Current Data Parameters
NAME 500-2020
EXPNO 151
PROCNO 1
F2 - Acquisition Parameters
Date_ 20200107
Time 17.39 h
INSTRUM spect
PROBHD 5 mm CPPBBO BB
PULPROG hsqcetgp
TD 1024
SOLVENT CDCl3
NS 2
DS 16
SWH 4237.288 Hz
FIDRES 8.275953 Hz
AQ 0.1208320 sec
RG 192.89
DW 118.000 usec
DE 6.50 usec
TE 298.2 K
CNST2 145.0000000
d0 0.00000300 sec
D1 1.45801604 sec
d4 0.00172414 sec
d11 0.03000000 sec
D16 0.00020000 sec
DELTA 0.00122718 sec
DELTA1 0.00071638 sec
in0 0 sec
ST1CNT 0
d0orig 0.00000300 sec
ph1loop 0
t1loop 0
SFO1 500.1322296 MHz
NUC1 1H
P1 10.59 usec
p2 21.18 usec
P28 1000.00 usec
PLW1 20.00000000 W
SFO2 125.7671682 MHz
NUC2 13C
CPDPRG[2 garp
P3 10.00 usec
p4 20.00 usec
PCPD2 70.00 usec
PLW2 58.00000000 W
PLW12 1.18369997 W
GPNAM[1] SMSQ10.100
GPZ1 80.00 %
GPNAM[2] SMSQ10.100
GPZ2 20.10 %
P16 1000.00 usec
F1 - Acquisition parameters
TD 254
SFO1 125.7672 MHz
FIDRES 164.041992 Hz
SW 165.650 ppm
FnMODE Echo-Antiecho
F2 - Processing parameters
SI 1024
SF 500.1300221 MHz
WDW QSINE
SSB 2
LB 0 Hz
GB 0
PC 1.40
F1 - Processing parameters
SI 1024
MC2 echo-antiecho
SF 125.7577885 MHz
WDW QSINE
SSB 2
LB 0 Hz
GB 0

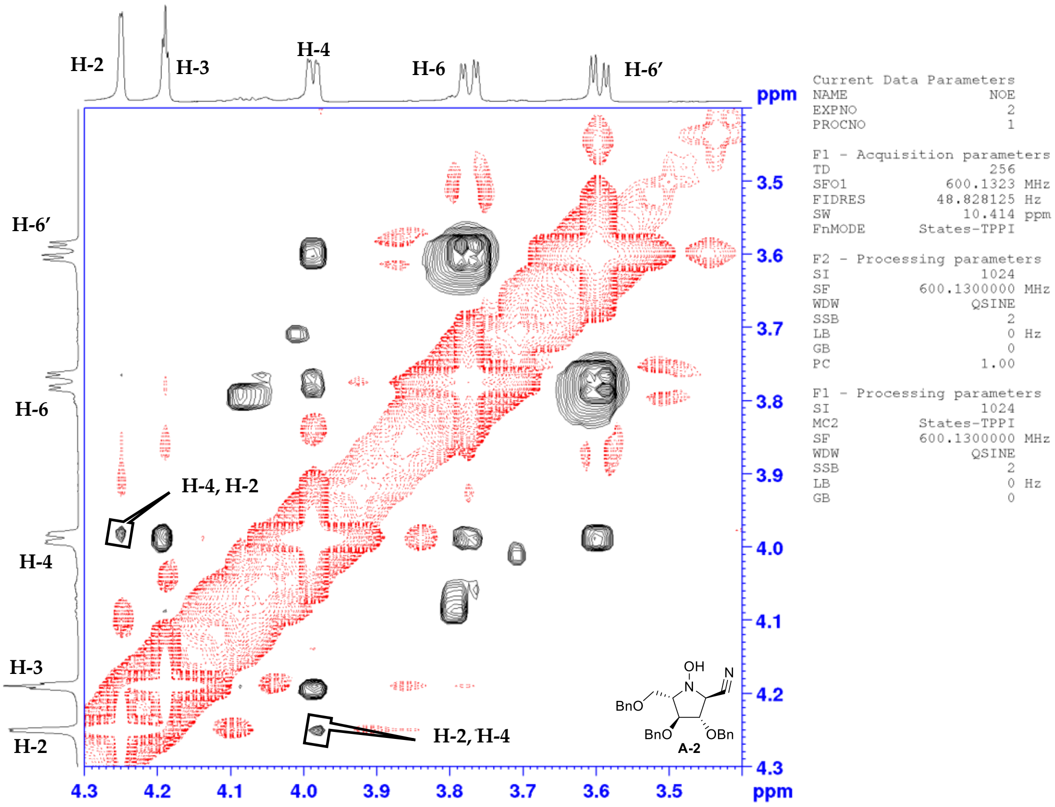
H-2
H-3
H-4
H-6
H-6′
H-4, H-2
H-2, H-4
ppm
BnO
BnO
OBn
OH
N
N
A-2
Current Data Parameters
NAME NOE
EXPNO 2
PROCNO 1
F1 - Acquisition parameters
TD 256
SFO1 600.1323 MHz
FIDRES 48.828125 Hz
SW 10.414 ppm
FnMODE States-TPPI
F2 - Processing parameters
SI 1024
SF 600.1300000 MHz
WDW QSINE
SSB 2
LB 0 Hz
GB 0
PC 1.00
F1 - Processing parameters
SI 1024
MC2 States-TPPI
SF 600.1300000 MHz
WDW QSINE
SSB 2
LB 0 Hz
GB 0

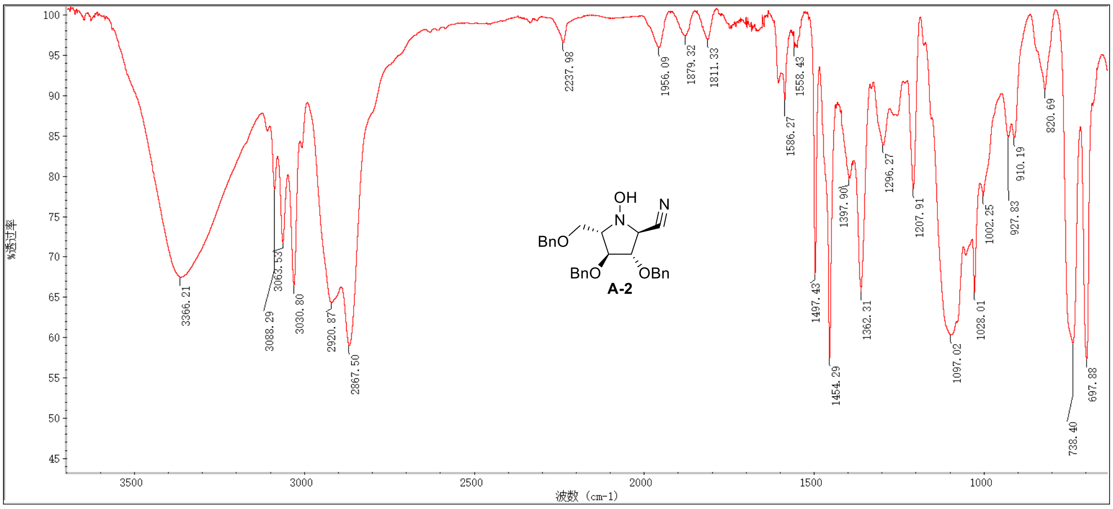
%透过率
波数（cm-1)
3366.21
3088.29
3063.53
3030.80
2920.87
2867.50
2237.98
1956.09
1879.32
1811.33
1586.27
1558.43
1497.43
1454.29
1397.90
1362.31
1296.27
1207.91
1097.02
1028.01
1002.25
927.83
910.19
820.69
738.40
697.88
BnO
BnO
OBn
OH
N
N
A-2

Compound **B-2:**

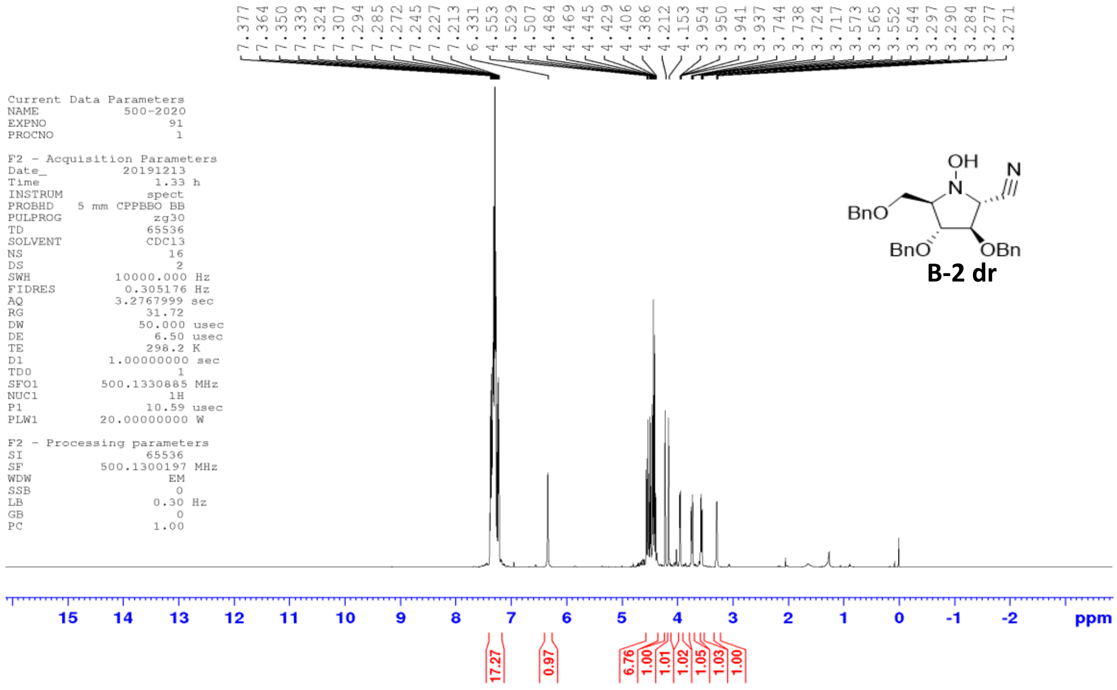
7.377 7.364 7.350 7.339 7.324 7.307 7.294 7.285 7.272 7.245 7.227 7.213 6.331 4.553 4.529 4.507 4.484 4.469 4.445 4.429 4.406 4.386 4.212 4.153 3.954 3.950 3.941 3.937 3.744 3.738 3.724 3.717 3.573 3.565 3.552 3.544 3.297 3.290 3.284 3.277 3.271
Current Data Parameters
NAME 500-2020
EXPNO 91
PROCNO 1
F2 - Acquisition Parameters
Date_ 20191213
Time 1.33 h
INSTRUM spect
PROBHD 5 mm CPPBBO BB
PULPROG zg30
TD 65536
SOLVENT CDCl3
NS 16
DS 2
SWH 10000.000 Hz
FIDRES 0.305176 Hz
AQ 3.2767999 sec
RG 31.72
DW 50.000 usec
DE 6.50 usec
TE 298.2 K
D1 1.00000000 sec
TD0 1
SFO1 500.1330885 MHz
NUC1 1H
P1 10.59 usec
PLW1 20.00000000 W
F2 - Processing parameters
SI 65536
SF 500.1300197 MHz
WDW EM
SSB 0
LB 0.30 Hz
GB 0
PC 1.00
OH
N
N
BnO
BnO
OBn
B-2 dr
15 14 13 12 11 10 9 8 7 6 5 4 3 2 1 0 -1 -2 ppm
17.27 0.97 6.76 1.00 1.01 1.02 1.05 1.03 1.00

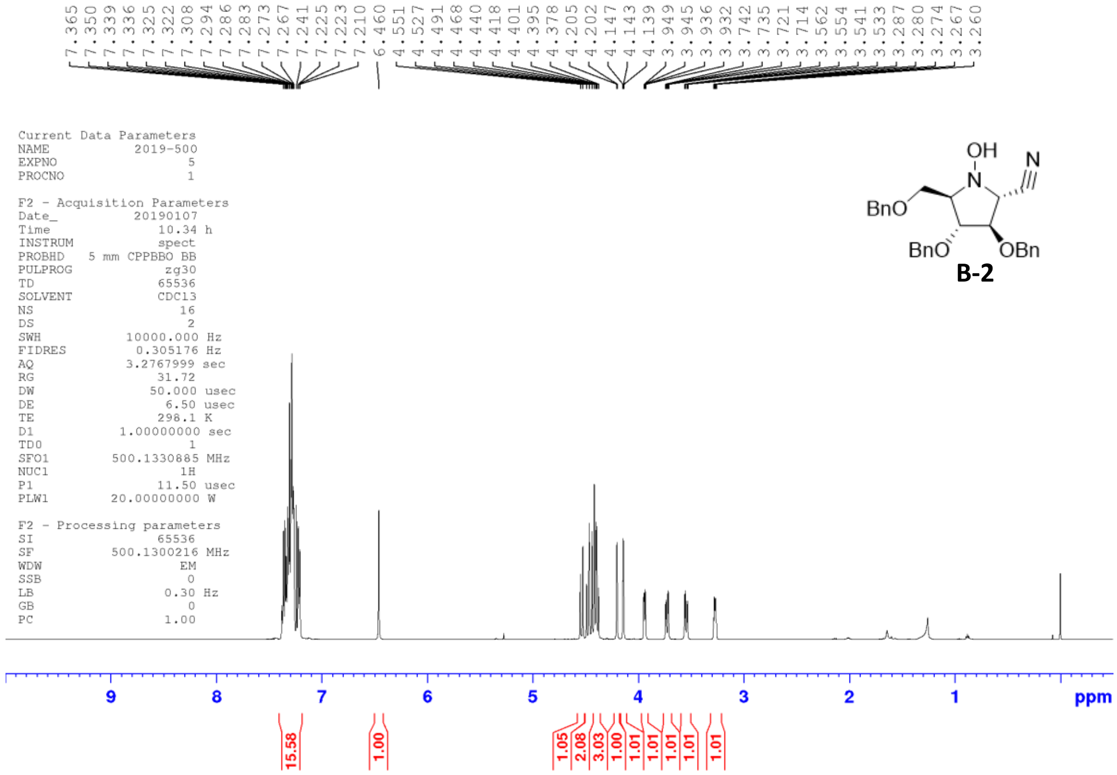
7.365 7.350 7.339 7.336 7.325 7.322 7.308 7.294 7.286 7.283 7.273 7.267 7.241 7.225 7.223 7.210 6.460 4.551 4.527 4.491 4.468 4.440 4.418 4.401 4.395 4.378 4.205 4.202 4.147 4.143 4.139 3.949 3.945 3.936 3.932 3.742 3.735 3.721 3.714 3.562 3.554 3.541 3.533 3.287 3.280 3.274 3.267 3.260
Current Data Parameters
NAME 2019-500
EXPNO 5
PROCNO 1
F2 - Acquisition Parameters
Date_ 20190107
Time 10.34 h
INSTRUM spect
PROBHD 5 mm CPPBBO BB
PULPROG zg30
TD 65536
SOLVENT CDCl3
NS 16
DS 2
SWH 10000.000 Hz
FIDRES 0.305176 Hz
AQ 3.2767999 sec
RG 31.72
DW 50.000 usec
DE 6.50 usec
TE 298.1 K
D1 1.00000000 sec
TD0 1
SFO1 500.1330885 MHz
NUC1 1H
P1 11.50 usec
PLW1 20.00000000 W
F2 - Processing parameters
SI 65536
SF 500.1300216 MHz
WDW EM
SSB 0
LB 0.30 Hz
GB 0
PC 1.00
OH
N
N
BnO
BnO
OBn
B-2
9 8 7 6 5 4 3 2 1 ppm
15.58 1.00 1.05 2.08 3.03 1.00 1.01 1.01 1.01 1.01 1.01

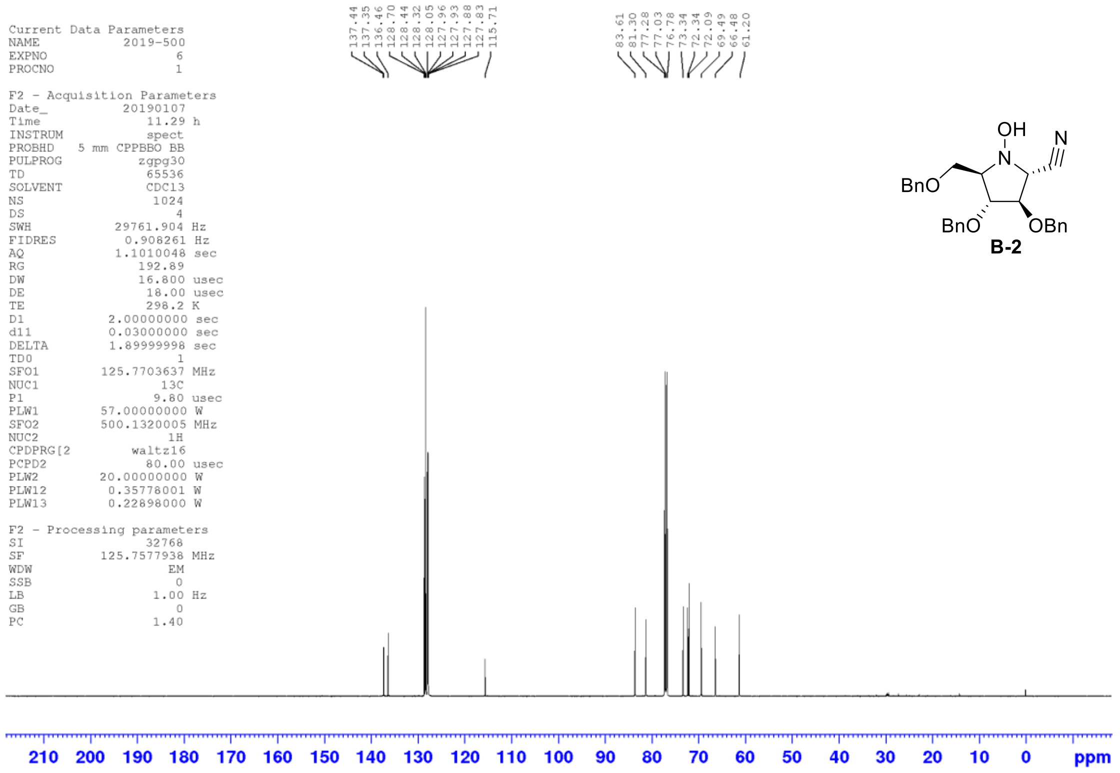

Current Data Parameters
NAME 2019-500
EXPNO 6
PROCNO 1
F2 - Acquisition Parameters
Date_ 20190107
Time 11.29 h
INSTRUM spect
PROBHD 5 mm CPPBBO BB
PULPROG zgpg30
TD 65536
SOLVENT CDCl3
NS 1024
DS 4
SWH 29761.904 Hz
FIDRES 0.908261 Hz
AQ 1.1010048 sec
RG 192.89
DW 16.800 usec
DE 18.00 usec
TE 298.2 K
D1 2.00000000 sec
d11 0.03000000 sec
DELTA 1.89999998 sec
TD0 1
SFO1 125.7703637 MHz
NUC1 13C
P1 9.80 usec
PLW1 57.00000000 W
SFO2 500.1320005 MHz
NUC2 1H
CPDPRG[2 waltz16
PCPD2 80.00 usec
PLW2 20.00000000 W
PLW12 0.35778001 W
PLW13 0.22898000 W
F2 - Processing parameters
SI 32768
SF 125.7577938 MHz
WDW EM
SSB 0
LB 1.00 Hz
GB 0
PC 1.40
137.44
137.35
136.46
128.70
128.44
128.32
128.05
127.96
127.93
127.88
127.83
115.71
83.61
81.30
77.28
77.03
76.78
73.34
72.34
72.09
69.49
66.48
61.20
OH
N
N
BnO
BnO
OBn
B-2
210 200 190 180 170 160 150 140 130 120 110 100 90 80 70 60 50 40 30 20 10 0 ppm

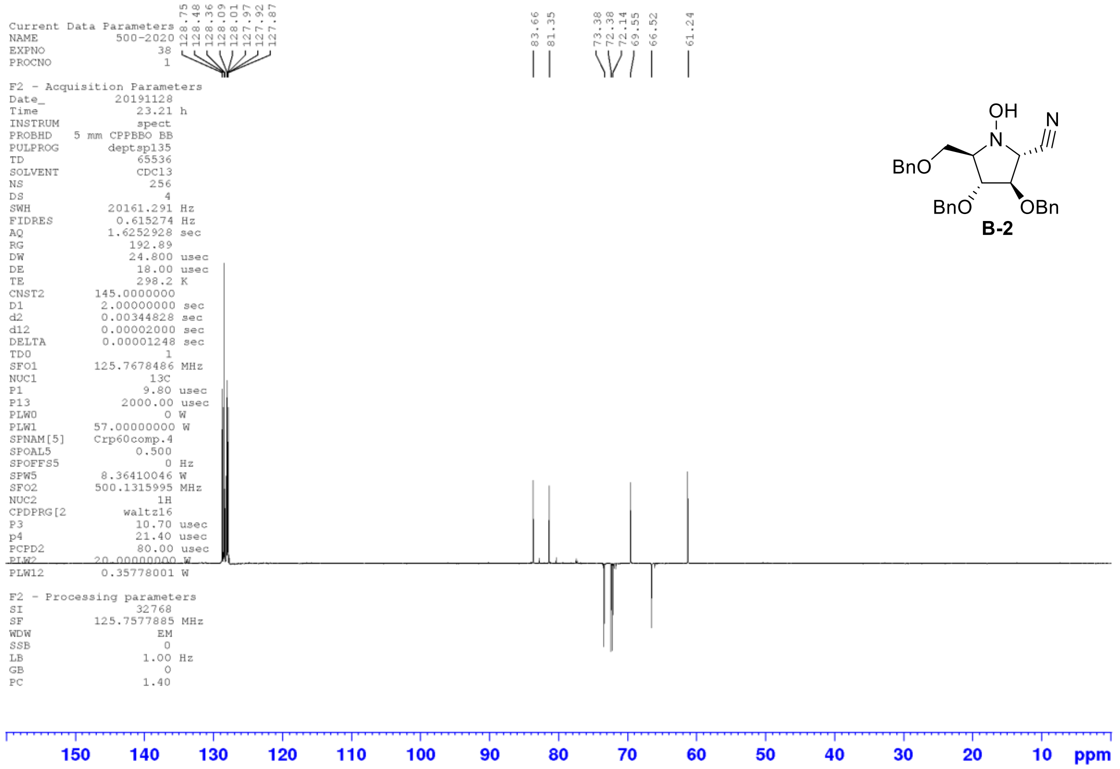

Current Data Parameters
NAME 500-2020
EXPNO 38
PROCNO 1
F2 - Acquisition Parameters
Date_ 20191128
Time 23.21 h
INSTRUM spect
PROBHD 5 mm CPPBBO BB
PULPROG deptsp135
TD 65536
SOLVENT CDCl3
NS 256
DS 4
SWH 20161.291 Hz
FIDRES 0.615274 Hz
AQ 1.6252928 sec
RG 192.89
DW 24.800 usec
DE 18.00 usec
TE 298.2 K
CNST2 145.0000000
D1 2.00000000 sec
d2 0.00344828 sec
d12 0.00002000 sec
DELTA 0.00001248 sec
TD0 1
SFO1 125.7678486 MHz
NUC1 13C
P1 9.80 usec
P13 2000.00 usec
PLW0 0 W
PLW1 57.00000000 W
SPNAM[5] Crp60comp.4
SPOAL5 0.500
SPOFFS5 0 Hz
SPW5 8.36410046 W
SFO2 500.1315995 MHz
NUC2 1H
CPDPRG[2 waltz16
P3 10.70 usec
p4 21.40 usec
PCPD2 80.00 usec
PLW2 20.00000000 W
PLW12 0.35778001 W
F2 - Processing parameters
SI 32768
SF 125.7577885 MHz
WDW EM
SSB 0
LB 1.00 Hz
GB 0
PC 1.40
128.75
128.48
128.36
128.09
128.01
127.97
127.92
127.87
83.66
81.35
73.38
72.38
72.14
69.55
66.52
61.24
OH
N
N
BnO
BnO
OBn
B-2
150 140 130 120 110 100 90 80 70 60 50 40 30 20 10 ppm

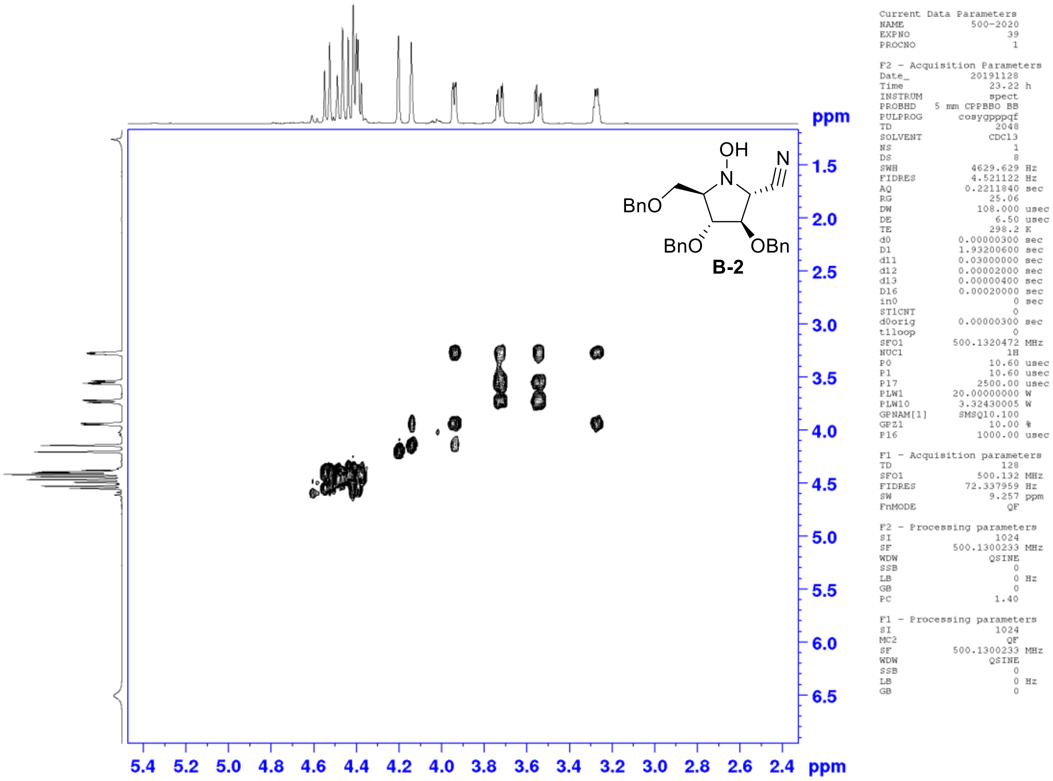
OH
N
N
BnO
BnO
OBn
B-2
ppm
Current Data Parameters
NAME 500-2020
EXPNO 39
PROCNO 1
F2 - Acquisition Parameters
Date_ 20191128
Time 23.22 h
INSTRUM spect
PROBHD 5 mm CPPBBO BB
PULPROG cosygpppqf
TD 2048
SOLVENT CDCl3
NS 1
DS 8
SWH 4629.629 Hz
FIDRES 4.521122 Hz
AQ 0.2211840 sec
RG 25.06
DW 108.000 usec
DE 6.50 usec
TE 298.2 K
d0 0.00000300 sec
D1 1.93200600 sec
d11 0.03000000 sec
d12 0.00002000 sec
d13 0.00000400 sec
D16 0.00020000 sec
in0 0 sec
ST1CNT 0
d0orig 0.00000300 sec
t1loop 0
SFO1 500.1320472 MHz
NUC1 1H
P0 10.60 usec
P1 10.60 usec
P17 2500.00 usec
PLW1 20.00000000 W
PLW10 3.32430005 W
GPNAM[1] SMSQ10.100
GPZ1 10.00 %
P16 1000.00 usec
F1 - Acquisition parameters
TD 128
SFO1 500.132 MHz
FIDRES 72.337959 Hz
SW 9.257 ppm
FnMODE QF
F2 - Processing parameters
SI 1024
SF 500.1300233 MHz
WDW QSINE
SSB 0
LB 0 Hz
GB 0
PC 1.40
F1 - Processing parameters
SI 1024
MC2 QF
SF 500.1300233 MHz
WDW QSINE
SSB 0
LB 0 Hz
GB 0

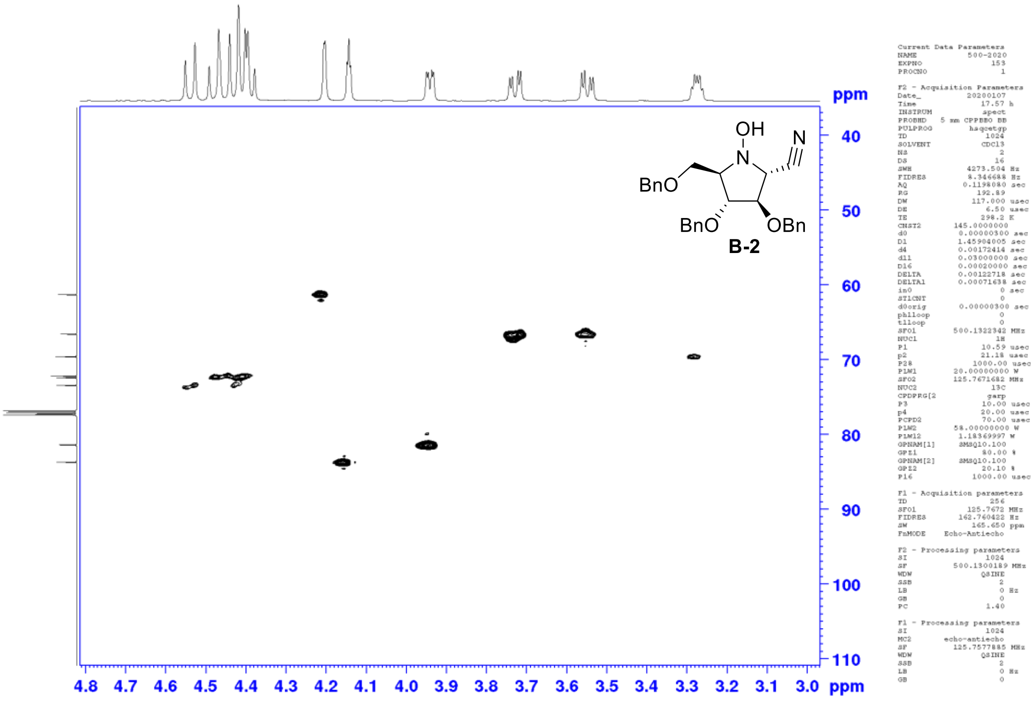
OH
N
N
BnO
BnO
OBn
B-2
ppm
Current Data Parameters
NAME 500-2020
EXPNO 153
PROCNO 1
F2 - Acquisition Parameters
Date_ 20200107
Time 17.57 h
INSTRUM spect
PROBHD 5 mm CPPBBO BB
PULPROG hsqcetgp
TD 1024
SOLVENT CDCl3
NS 2
DS 16
SWH 4273.504 Hz
FIDRES 8.346688 Hz
AQ 0.1198080 sec
RG 192.89
DW 117.000 usec
DE 6.50 usec
TE 298.2 K
CNST2 145.0000000
d0 0.00000300 sec
D1 1.45904005 sec
d4 0.00172414 sec
d11 0.03000000 sec
D16 0.00020000 sec
DELTA 0.00122718 sec
DELTA1 0.00071638 sec
in0 0 sec
ST1CNT 0
d0orig 0.00000300 sec
ph1loop 0
t1loop 0
SFO1 500.1322342 MHz
NUC1 1H
P1 10.59 usec
p2 21.18 usec
P28 1000.00 usec
PLW1 20.00000000 W
SFO2 125.7671682 MHz
NUC2 13C
CPDPRG[2 garp
P3 10.00 usec
p4 20.00 usec
PCPD2 70.00 usec
PLW2 58.00000000 W
PLW12 1.18369997 W
GPNAM[1] SMSQ10.100
GPZ1 80.00 %
GPNAM[2] SMSQ10.100
GPZ2 20.10 %
P16 1000.00 usec
F1 - Acquisition parameters
TD 256
SFO1 125.7672 MHz
FIDRES 162.760422 Hz
SW 165.650 ppm
FnMODE Echo-Antiecho
F2 - Processing parameters
SI 1024
SF 500.1300189 MHz
WDW QSINE
SSB 2
LB 0 Hz
GB 0
PC 1.40
F1 - Processing parameters
SI 1024
MC2 echo-antiecho
SF 125.7577885 MHz
WDW QSINE
SSB 2
LB 0 Hz
GB 0

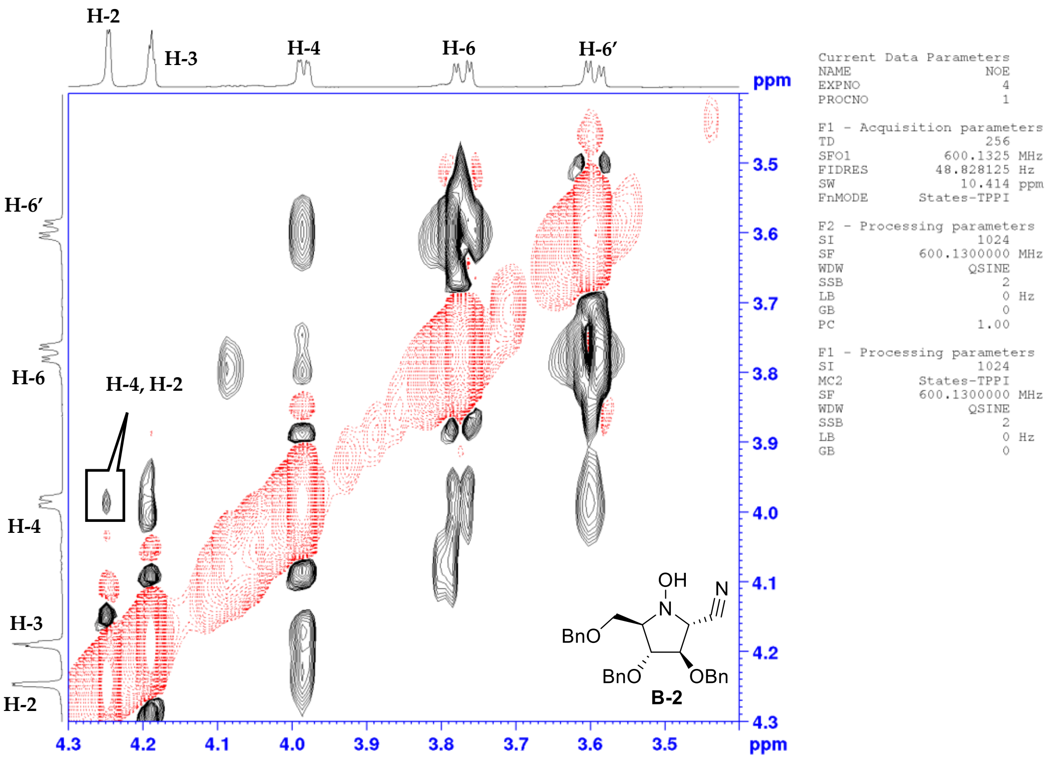

H-2
H-3
H-4
H-6
H-6′
H-4, H-2
ppm
4.3 4.2 4.1 4.0 3.9 3.8 3.7 3.6 3.5
OH
N
N
BnO
BnO
OBn
B-2
Current Data Parameters
NAME NOE
EXPNO 4
PROCNO 1
F1 - Acquisition parameters
TD 256
SFO1 600.1325 MHz
FIDRES 48.828125 Hz
SW 10.414 ppm
FnMODE States-TPPI
F2 - Processing parameters
SI 1024
SF 600.1300000 MHz
WDW QSINE
SSB 2
LB 0 Hz
GB 0
PC 1.00
F1 - Processing parameters
SI 1024
MC2 States-TPPI
SF 600.1300000 MHz
WDW QSINE
SSB 2
LB 0 Hz
GB 0

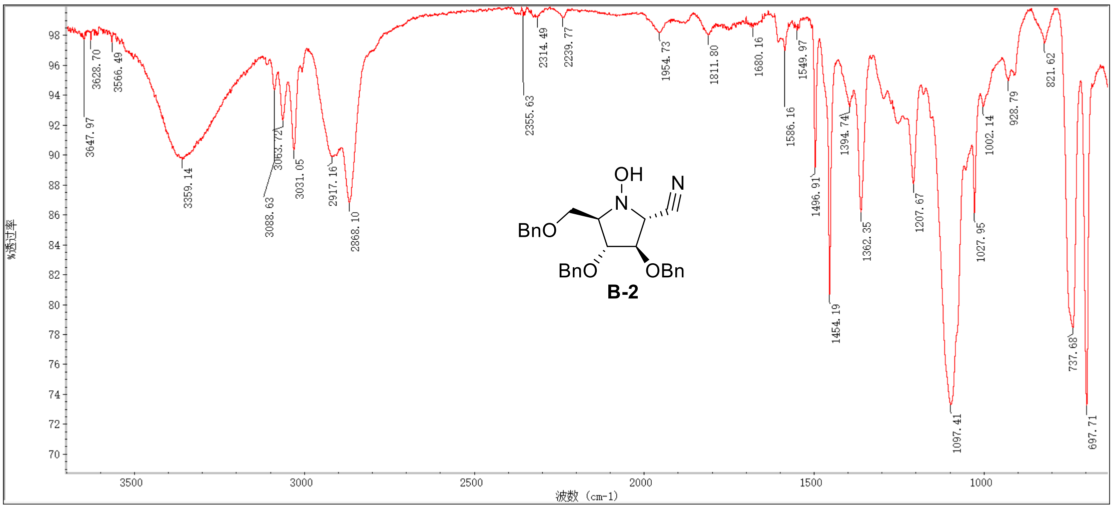

%透过率
波数 (cm-1)
3647.97
3628.70
3566.49
3359.14
3088.63
3063.72
3031.05
2917.16
2868.10
2355.63
2314.49
2239.77
1954.73
1811.80
1680.16
1586.16
1549.97
1496.91
1454.19
1394.74
1362.35
1207.67
1097.41
1027.95
1002.14
928.79
821.62
737.68
697.71
OH
N
N
BnO
BnO
OBn
B-2

Compound **C-2:**

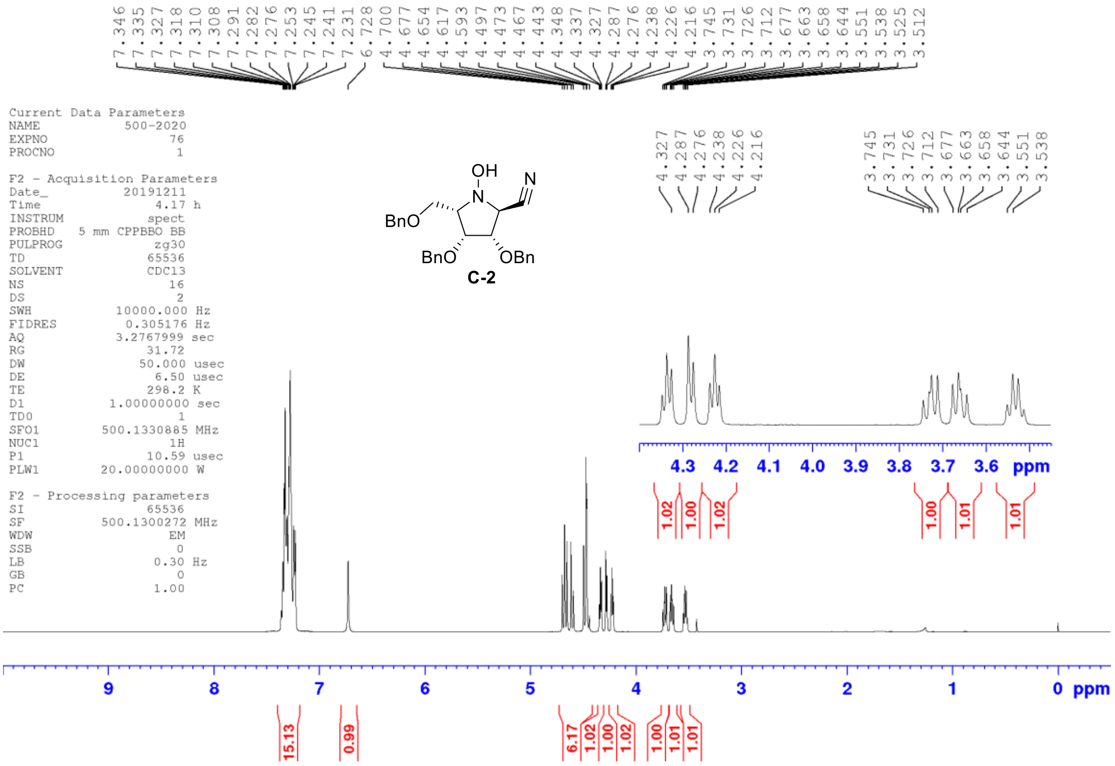
7.346 7.335 7.327 7.318 7.310 7.308 7.291 7.282 7.276 7.253 7.245 7.241 7.231 6.728 4.700 4.677 4.654 4.617 4.593 4.497 4.473 4.467 4.443 4.348 4.337 4.327 4.287 4.276 4.238 4.226 4.216 3.745 3.731 3.726 3.712 3.677 3.663 3.658 3.644 3.551 3.538 3.525 3.512
Current Data Parameters
NAME 500-2020
EXPNO 76
PROCNO 1
F2 - Acquisition Parameters
Date_ 20191211
Time 4.17 h
INSTRUM spect
PROBHD 5 mm CPPBBO BB
PULPROG zg30
TD 65536
SOLVENT CDCl3
NS 16
DS 2
SWH 10000.000 Hz
FIDRES 0.305176 Hz
AQ 3.2767999 sec
RG 31.72
DW 50.000 usec
DE 6.50 usec
TE 298.2 K
D1 1.00000000 sec
TD0 1
SFO1 500.1330885 MHz
NUC1 1H
P1 10.59 usec
PLW1 20.00000000 W
F2 - Processing parameters
SI 65536
SF 500.1300272 MHz
WDW EM
SSB 0
LB 0.30 Hz
GB 0
PC 1.00
OH
N
N
BnO
BnO
OBn
C-2
4.327 4.287 4.276 4.238 4.226 4.216
3.745 3.731 3.726 3.712 3.677 3.663 3.658 3.644 3.551 3.538
4.3 4.2 4.1 4.0 3.9 3.8 3.7 3.6 ppm
1.02 1.00 1.02 1.00 1.01 1.01
9 8 7 6 5 4 3 2 1 0 ppm
15.13 0.99 6.17 1.02 1.00 1.02 1.00 1.01 1.01

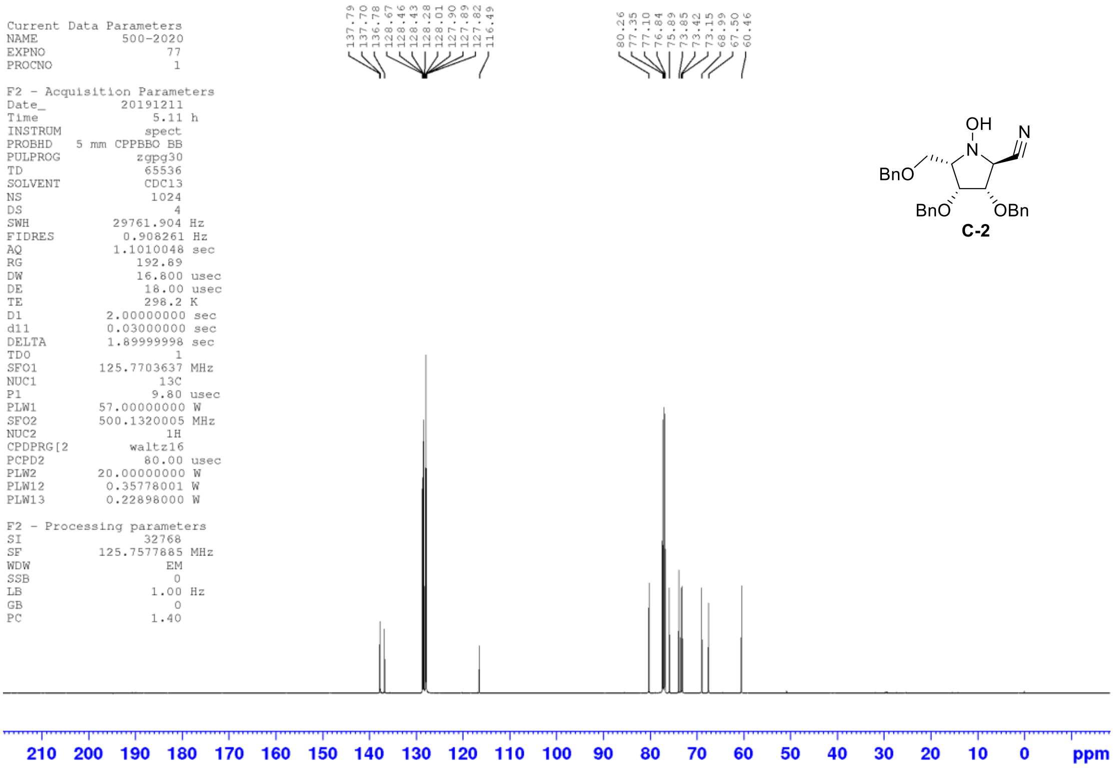
137.79 137.70 136.78 128.67 128.46 128.43 128.28 128.01 127.90 127.89 127.82 116.49
80.26 77.35 77.10 76.84 75.89 73.85 73.42 73.15 68.99 67.50 60.46
Current Data Parameters
NAME 500-2020
EXPNO 77
PROCNO 1
F2 - Acquisition Parameters
Date_ 20191211
Time 5.11 h
INSTRUM spect
PROBHD 5 mm CPPBBO BB
PULPROG zgpg30
TD 65536
SOLVENT CDCl3
NS 1024
DS 4
SWH 29761.904 Hz
FIDRES 0.908261 Hz
AQ 1.1010048 sec
RG 192.89
DW 16.800 usec
DE 18.00 usec
TE 298.2 K
D1 2.00000000 sec
d11 0.03000000 sec
DELTA 1.89999998 sec
TD0 1
SFO1 125.7703637 MHz
NUC1 13C
P1 9.80 usec
PLW1 57.00000000 W
SFO2 500.1320005 MHz
NUC2 1H
CPDPRG[2 waltz16
PCPD2 80.00 usec
PLW2 20.00000000 W
PLW12 0.35778001 W
PLW13 0.22898000 W
F2 - Processing parameters
SI 32768
SF 125.7577885 MHz
WDW EM
SSB 0
LB 1.00 Hz
GB 0
PC 1.40
OH
N
N
BnO
BnO
OBn
C-2
210 200 190 180 170 160 150 140 130 120 110 100 90 80 70 60 50 40 30 20 10 0 ppm

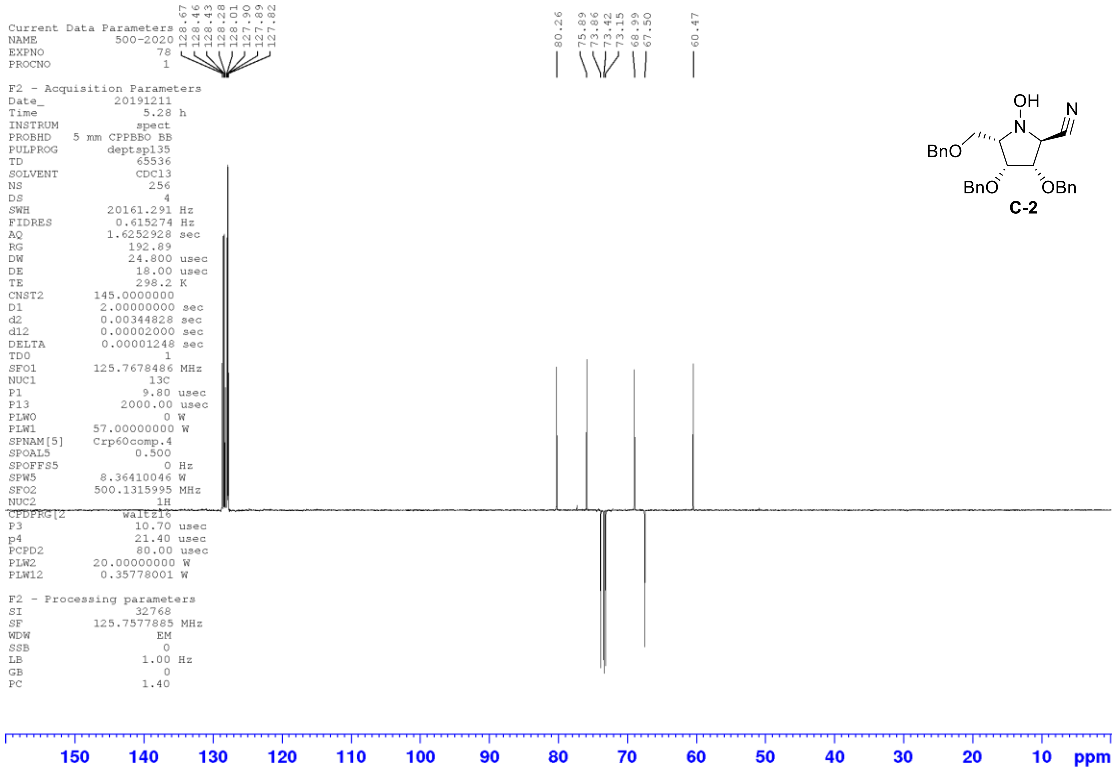
128.67
128.46
128.43
128.28
128.01
127.90
127.89
127.82
80.26
75.89
73.86
73.42
73.15
68.99
67.50
60.47
OH
N
BnO
BnO
OBn
C-2
ppm

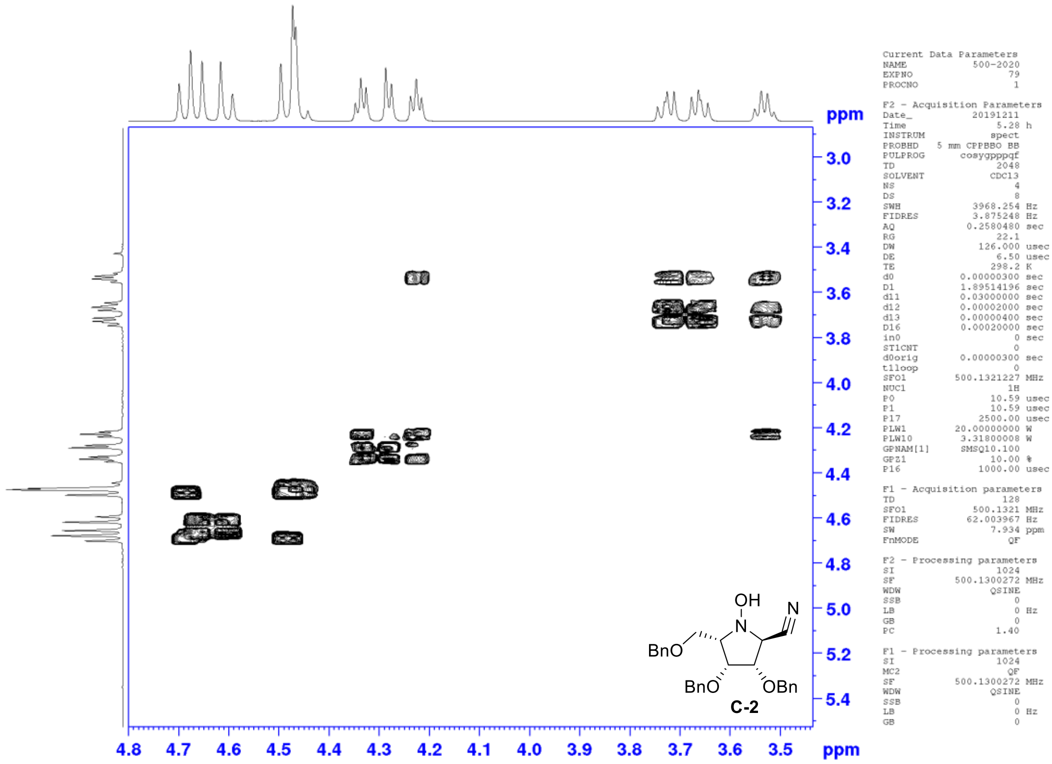
ppm
OH
N
BnO
BnO
OBn
C-2
ppm

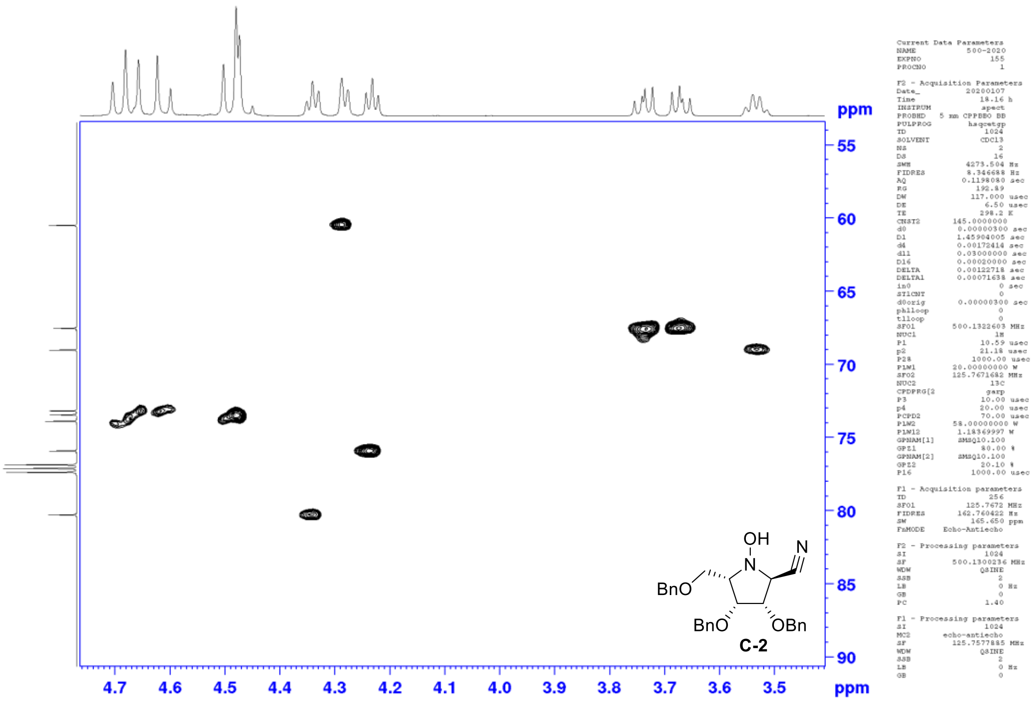
ppm
OH
N
BnO
BnO
OBn
C-2

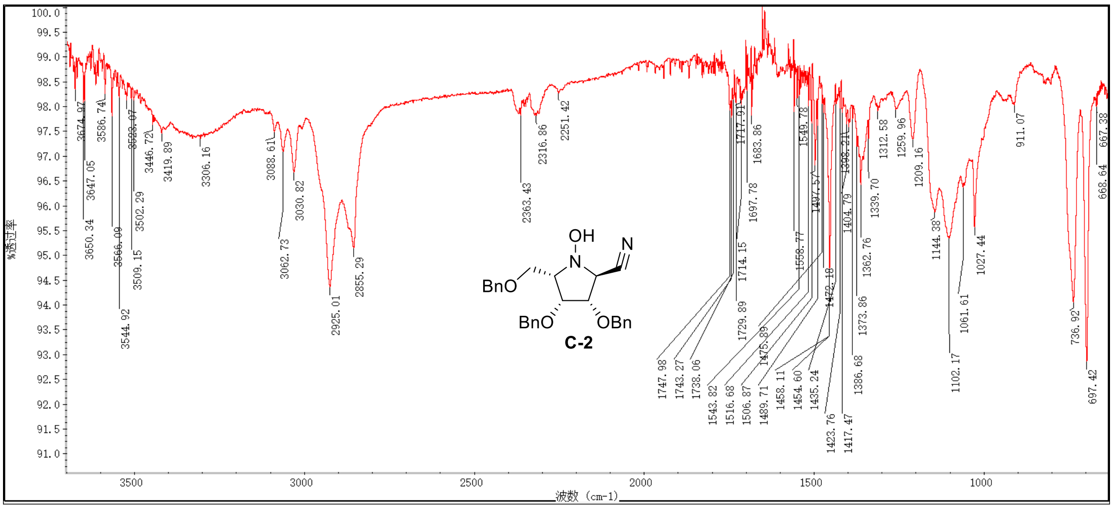
%透过率
OH
N
BnO
BnO
OBn
C-2
波数（cm-1）

Compound **D-2**:

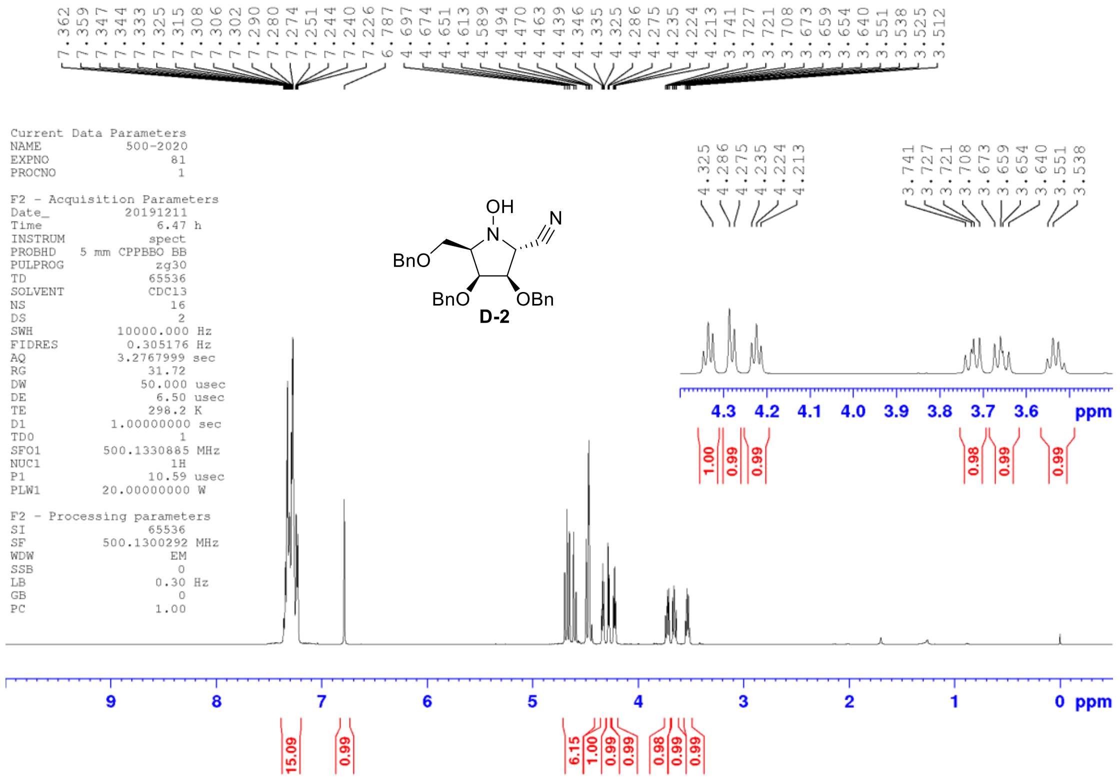

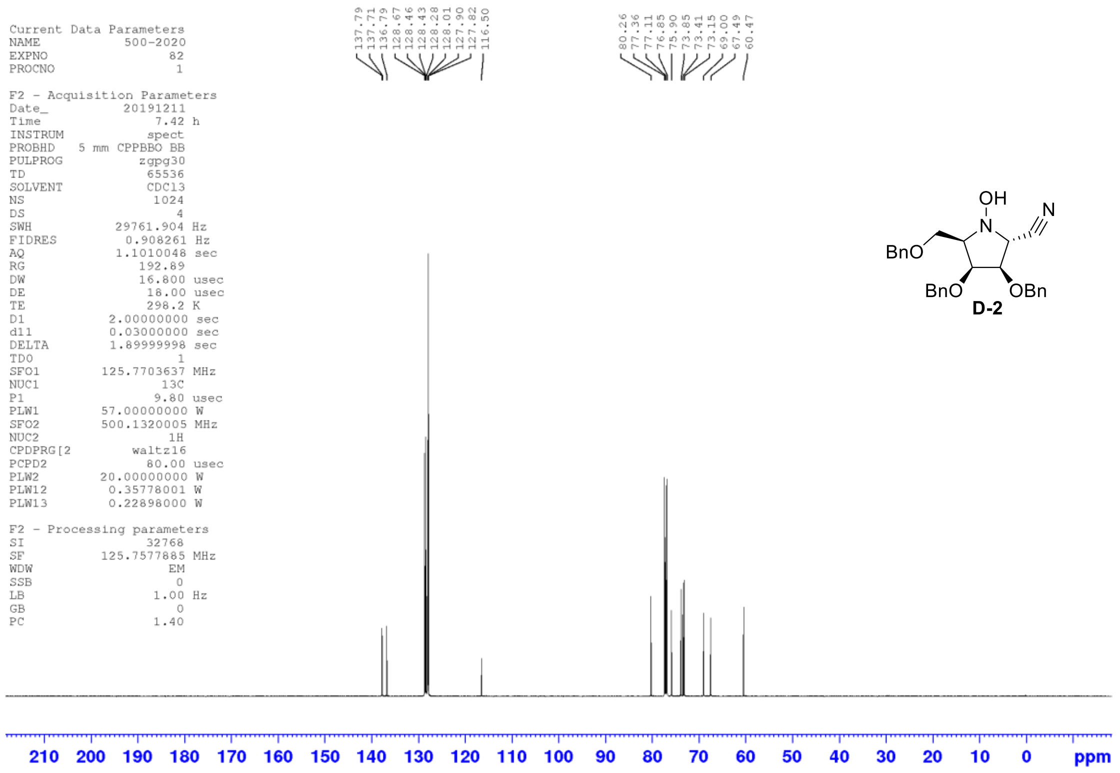

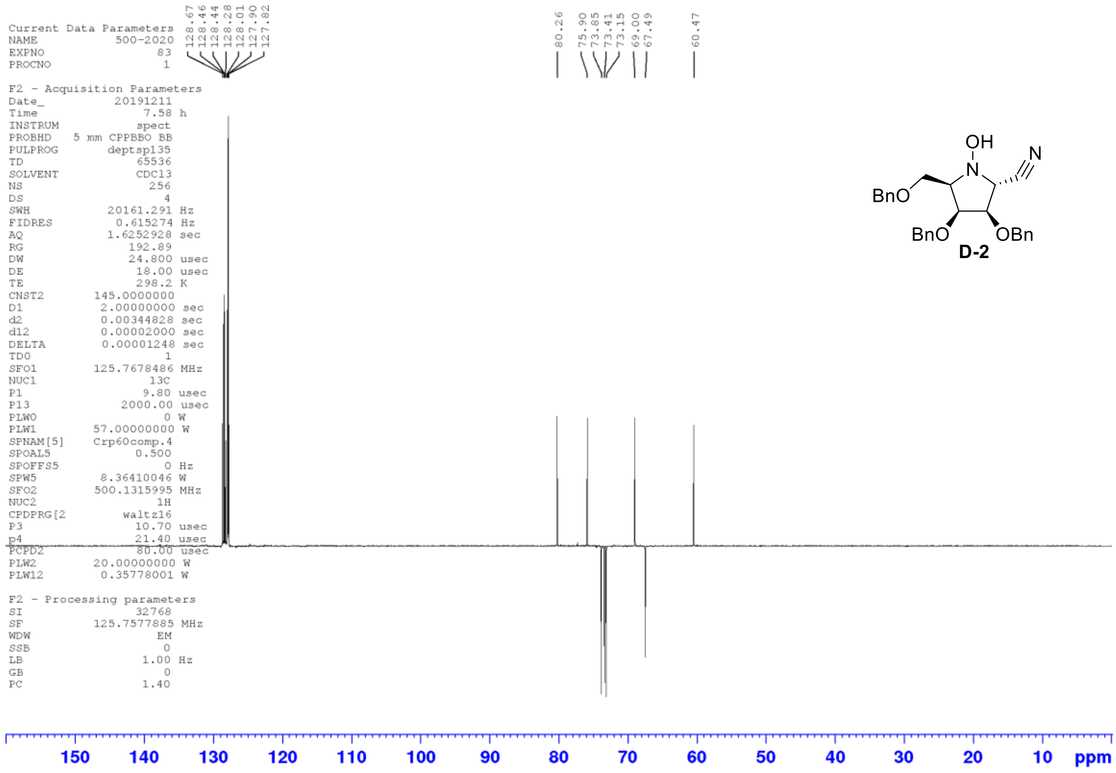

Current Data Parameters
NAME 500-2020
EXPNO 83
PROCNO 1
F2 - Acquisition Parameters
Date_ 20191211
Time 7.58 h
INSTRUM spect
PROBHD 5 mm CPPBBO BB
PULPROG deptsp135
TD 65536
SOLVENT CDCl3
NS 256
DS 4
SWH 20161.291 Hz
FIDRES 0.615274 Hz
AQ 1.6252928 sec
RG 192.89
DW 24.800 usec
DE 18.00 usec
TE 298.2 K
CNST2 145.0000000
D1 2.00000000 sec
d2 0.00344828 sec
d12 0.00002000 sec
DELTA 0.00001248 sec
TD0 1
SFO1 125.7678486 MHz
NUC1 13C
P1 9.80 usec
P13 2000.00 usec
PLW0 0 W
PLW1 57.00000000 W
SPNAM[5] Crp60comp.4
SPOAL5 0.500
SPOFFS5 0 Hz
SPW5 8.36410046 W
SFO2 500.1315995 MHz
NUC2 1H
CPDPRG[2 waltz16
P3 10.70 usec
p4 21.40 usec
PCPD2 80.00 usec
PLW2 20.00000000 W
PLW12 0.35778001 W
F2 - Processing parameters
SI 32768
SF 125.7577885 MHz
WDW EM
SSB 0
LB 1.00 Hz
GB 0
PC 1.40
128.67
128.46
128.44
128.28
128.01
127.90
127.82
80.26
75.90
73.85
73.41
73.15
69.00
67.49
60.47
OH
N
N
BnO
BnO
OBn
D-2
150
140
130
120
110
100
90
80
70
60
50
40
30
20
10
ppm

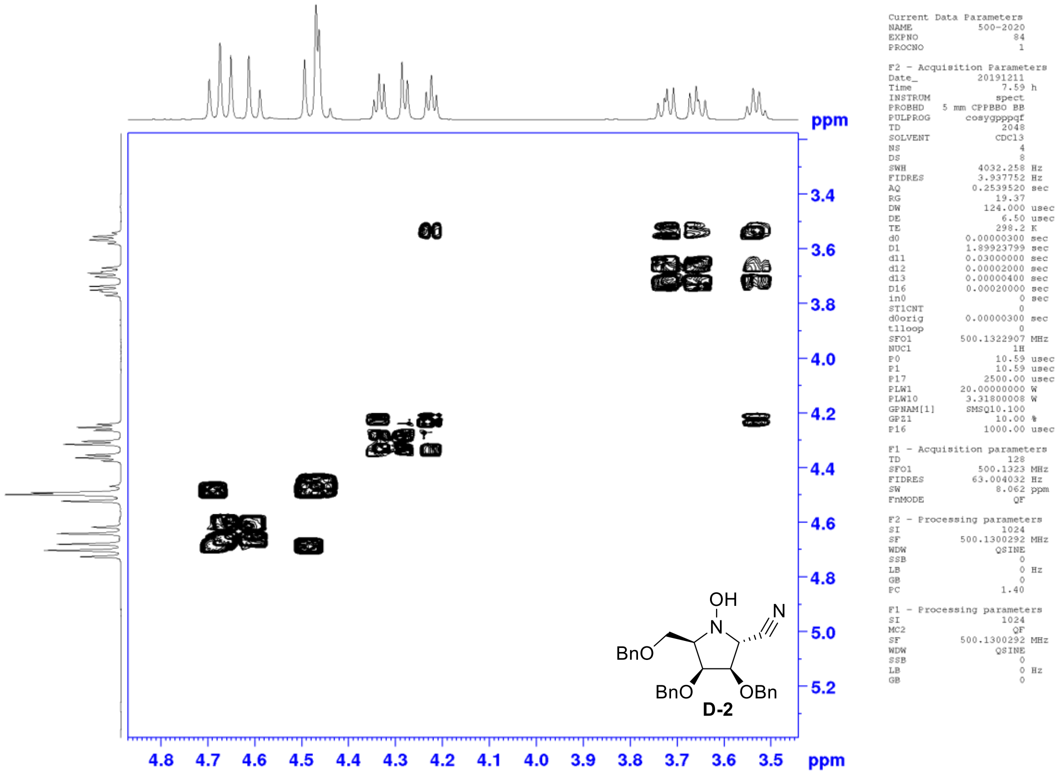

ppm
3.4
3.6
3.8
4.0
4.2
4.4
4.6
4.8
5.0
5.2
4.8
4.7
4.6
4.5
4.4
4.3
4.2
4.1
4.0
3.9
3.8
3.7
3.6
3.5
ppm
OH
N
N
BnO
BnO
OBn
D-2
Current Data Parameters
NAME 500-2020
EXPNO 84
PROCNO 1
F2 - Acquisition Parameters
Date_ 20191211
Time 7.59 h
INSTRUM spect
PROBHD 5 mm CPPBBO BB
PULPROG cosygpppqf
TD 2048
SOLVENT CDCl3
NS 4
DS 8
SWH 4032.258 Hz
FIDRES 3.937752 Hz
AQ 0.2539520 sec
RG 19.37
DW 124.000 usec
DE 6.50 usec
TE 298.2 K
d0 0.00000300 sec
D1 1.89923799 sec
d11 0.03000000 sec
d12 0.00002000 sec
d13 0.00000400 sec
D16 0.00020000 sec
in0 0 sec
ST1CNT 0
d0orig 0.00000300 sec
t1loop 0
SFO1 500.1322907 MHz
NUC1 1H
P0 10.59 usec
P1 10.59 usec
P17 2500.00 usec
PLW1 20.00000000 W
PLW10 3.31800008 W
GPNAM[1] SMSQ10.100
GPZ1 10.00 %
P16 1000.00 usec
F1 - Acquisition parameters
TD 128
SFO1 500.1323 MHz
FIDRES 63.004032 Hz
SW 8.062 ppm
FnMODE QF
F2 - Processing parameters
SI 1024
SF 500.1300292 MHz
WDW QSINE
SSB 0
LB 0 Hz
GB 0
PC 1.40
F1 - Processing parameters
SI 1024
MC2 QF
SF 500.1300292 MHz
WDW QSINE
SSB 0
LB 0 Hz
GB 0

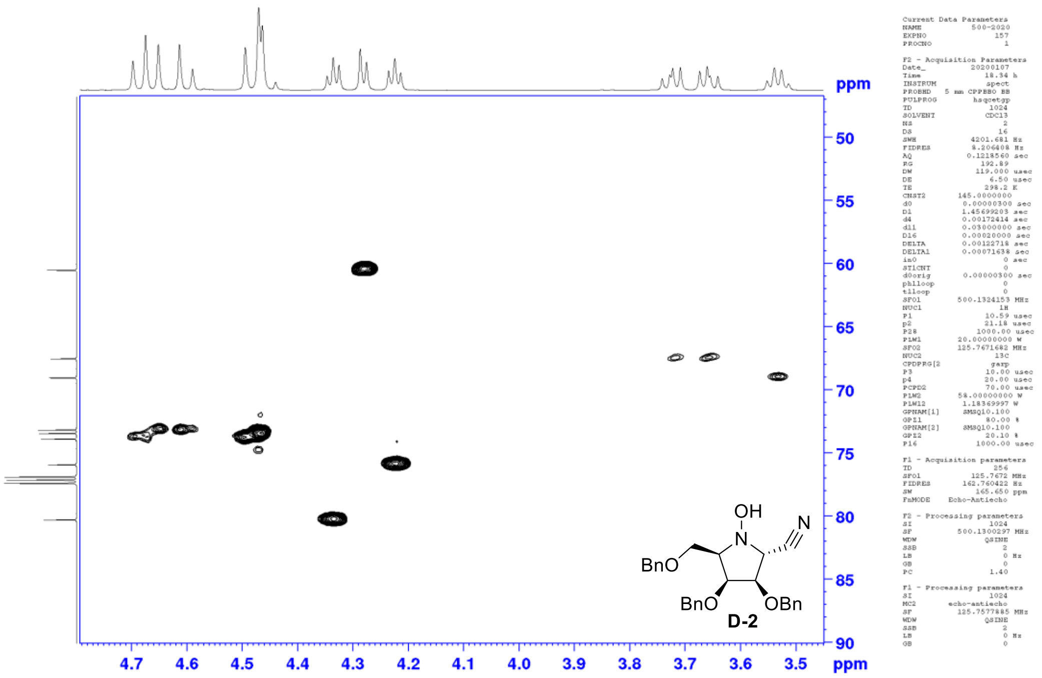

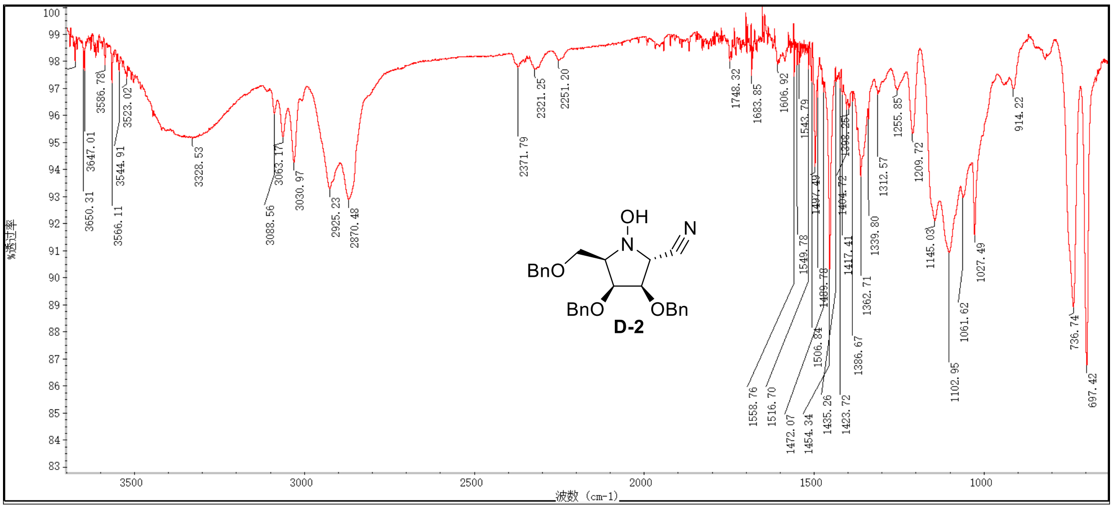

Compound **E-2a**:

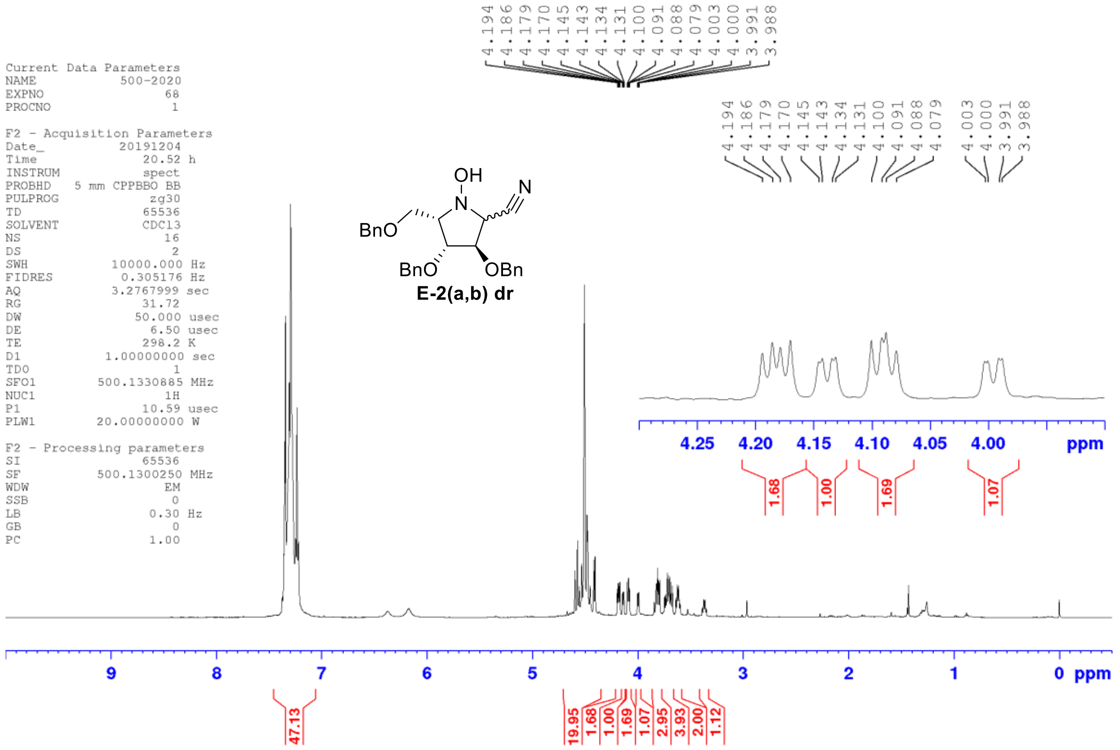

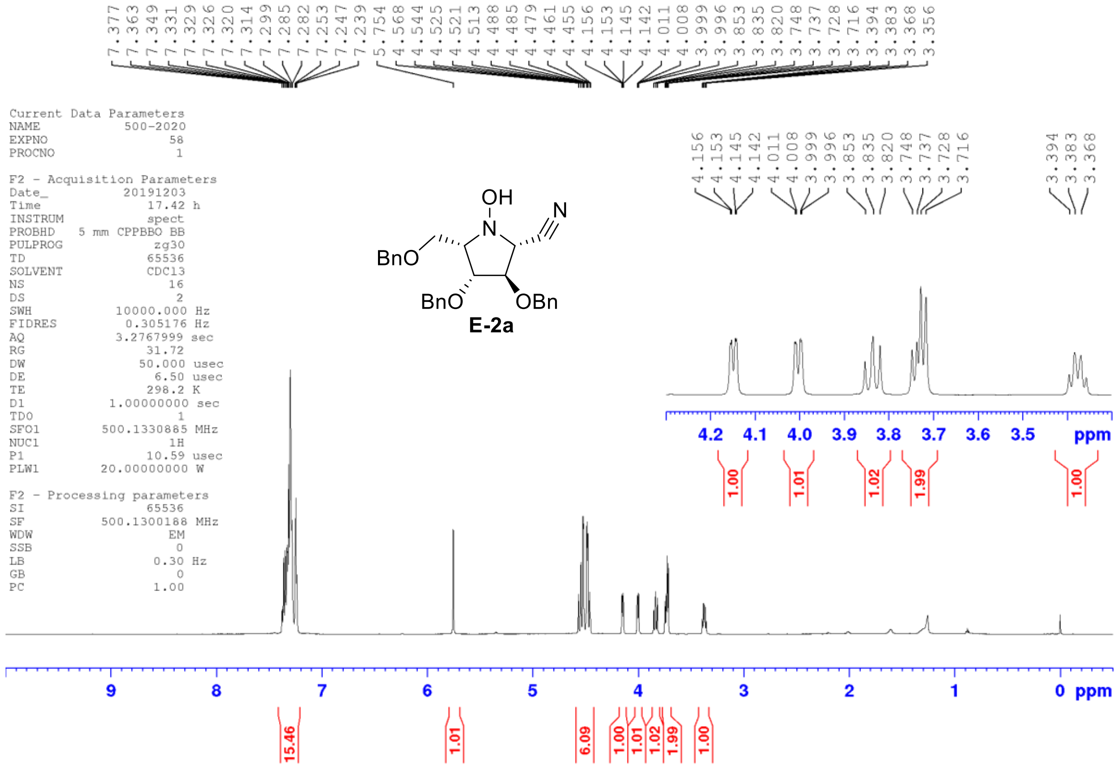

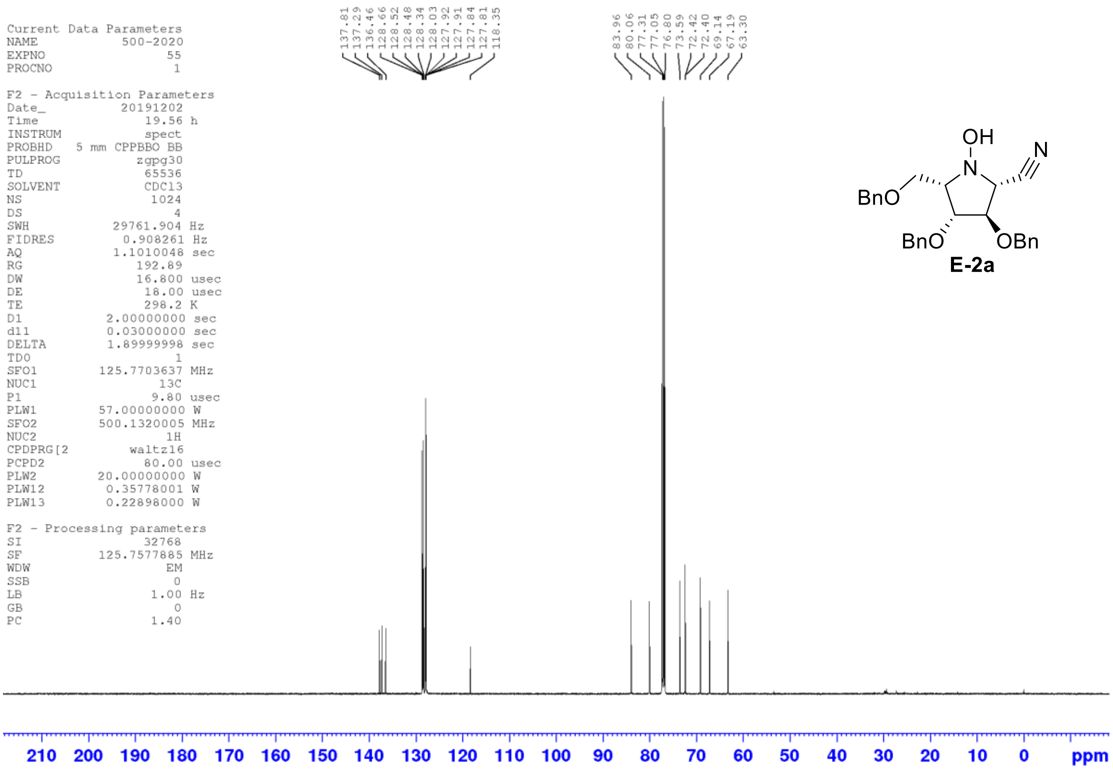

Current Data Parameters
NAME 500-2020
EXPNO 55
PROCNO 1
F2 - Acquisition Parameters
Date_ 20191202
Time 19.56 h
INSTRUM spect
PROBHD 5 mm CPPBBO BB
PULPROG zgpg30
TD 65536
SOLVENT CDCl3
NS 1024
DS 4
SWH 29761.904 Hz
FIDRES 0.908261 Hz
AQ 1.1010048 sec
RG 192.89
DW 16.800 usec
DE 18.00 usec
TE 298.2 K
D1 2.00000000 sec
d11 0.03000000 sec
DELTA 1.89999998 sec
TD0 1
SFO1 125.7703637 MHz
NUC1 13C
P1 9.80 usec
PLW1 57.00000000 W
SFO2 500.1320005 MHz
NUC2 1H
CPDPRG[2 waltz16
PCPD2 80.00 usec
PLW2 20.00000000 W
PLW12 0.35778001 W
PLW13 0.22898000 W
F2 - Processing parameters
SI 32768
SF 125.7577885 MHz
WDW EM
SSB 0
LB 1.00 Hz
GB 0
PC 1.40
137.81 137.29 136.46 128.66 128.52 128.48 128.34 128.03 127.92 127.91 127.84 127.81 118.35
83.96 80.06 77.31 77.05 76.80 73.59 72.42 72.40 69.14 67.19 63.30
OH
N
BnO
BnO
OBn
E-2a
210 200 190 180 170 160 150 140 130 120 110 100 90 80 70 60 50 40 30 20 10 0 ppm

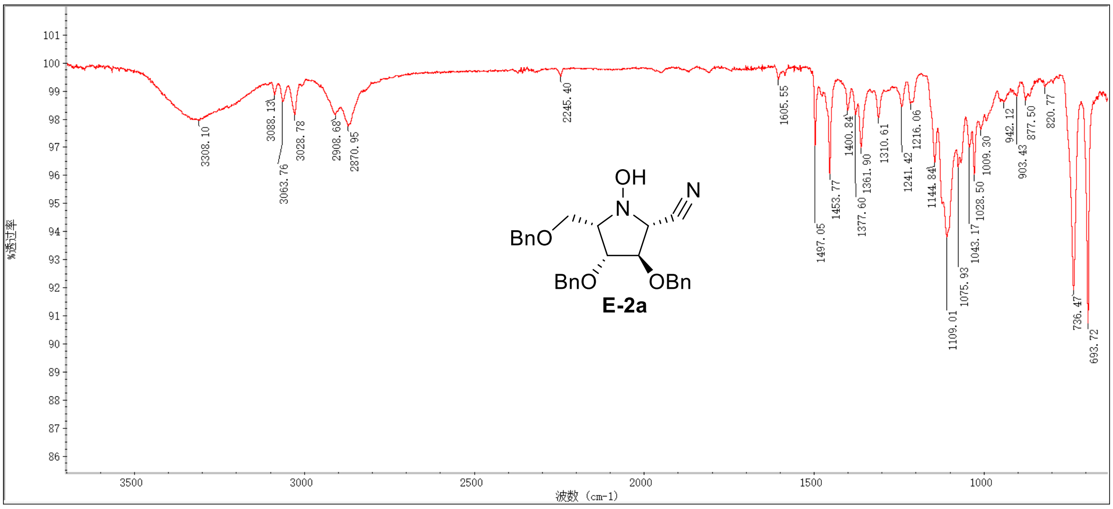

3308.10
3088.13
3063.76
3028.78
2908.68
2870.95
2245.40
1605.55
1497.05
1453.77
1400.84
1377.60
1361.90
1310.61
1241.42
1216.06
1144.84
1109.01
1075.93
1043.17
1028.50
1009.30
942.12
903.43
877.50
820.77
736.47
693.72
OH
N
BnO
BnO
OBn
E-2a
%透过率
波数 (cm-1)

Compound **E-2b**:

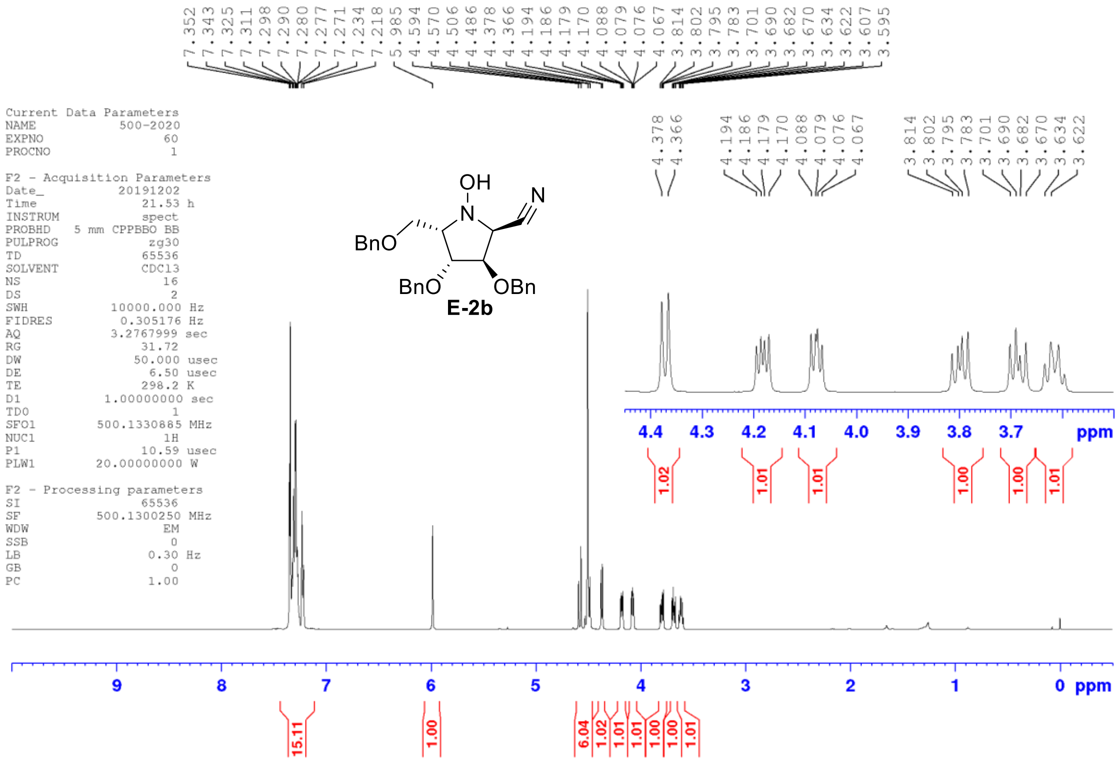

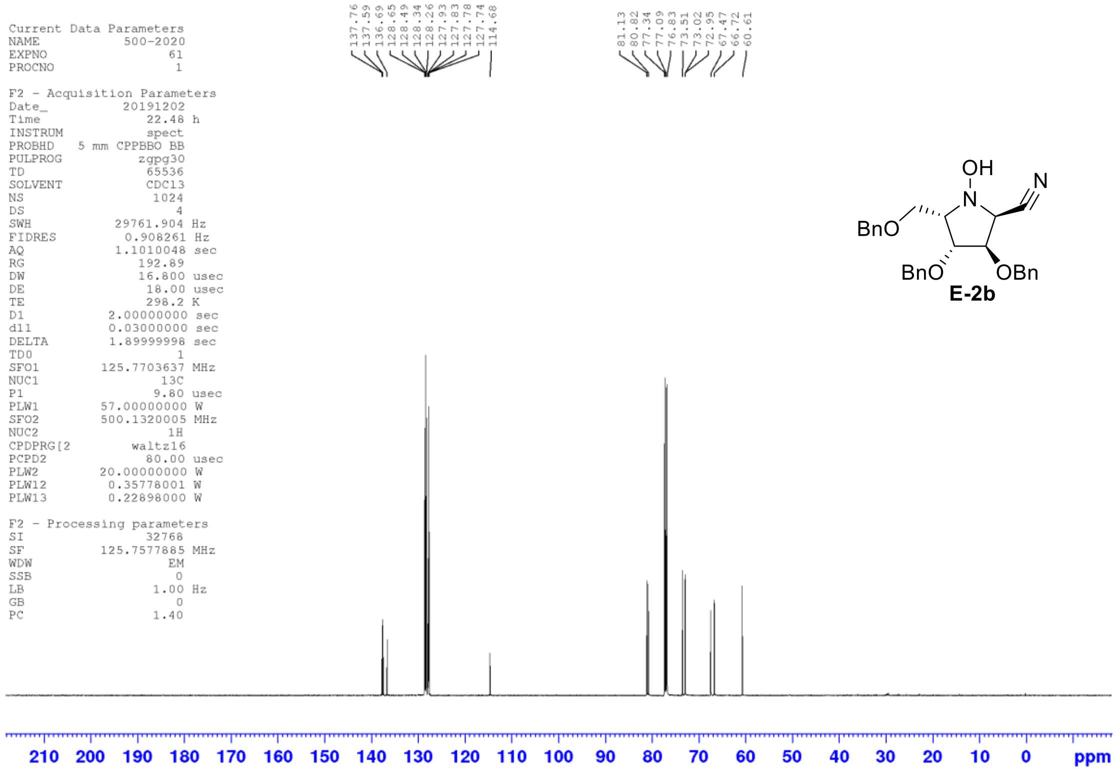

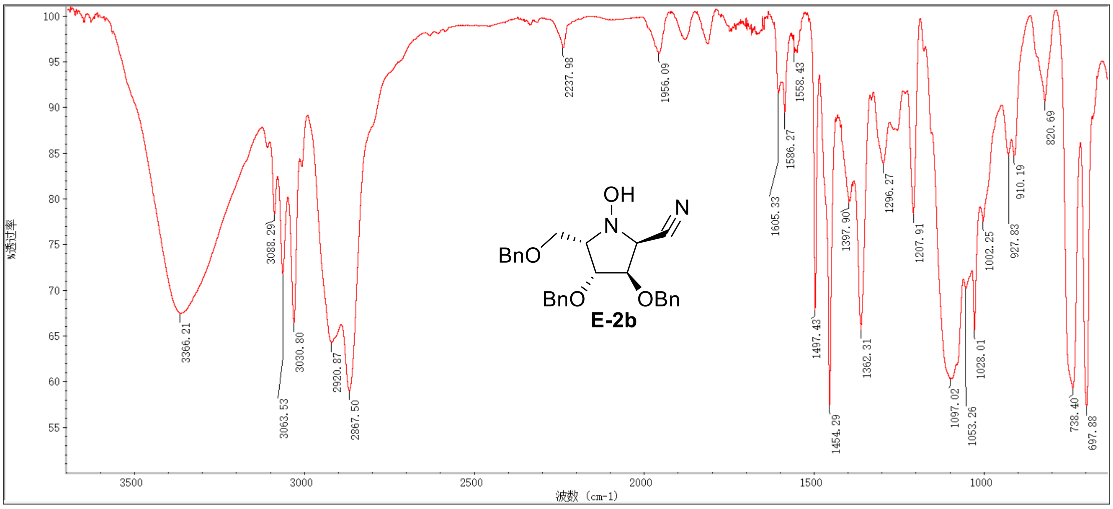

Compound **F-2a**:

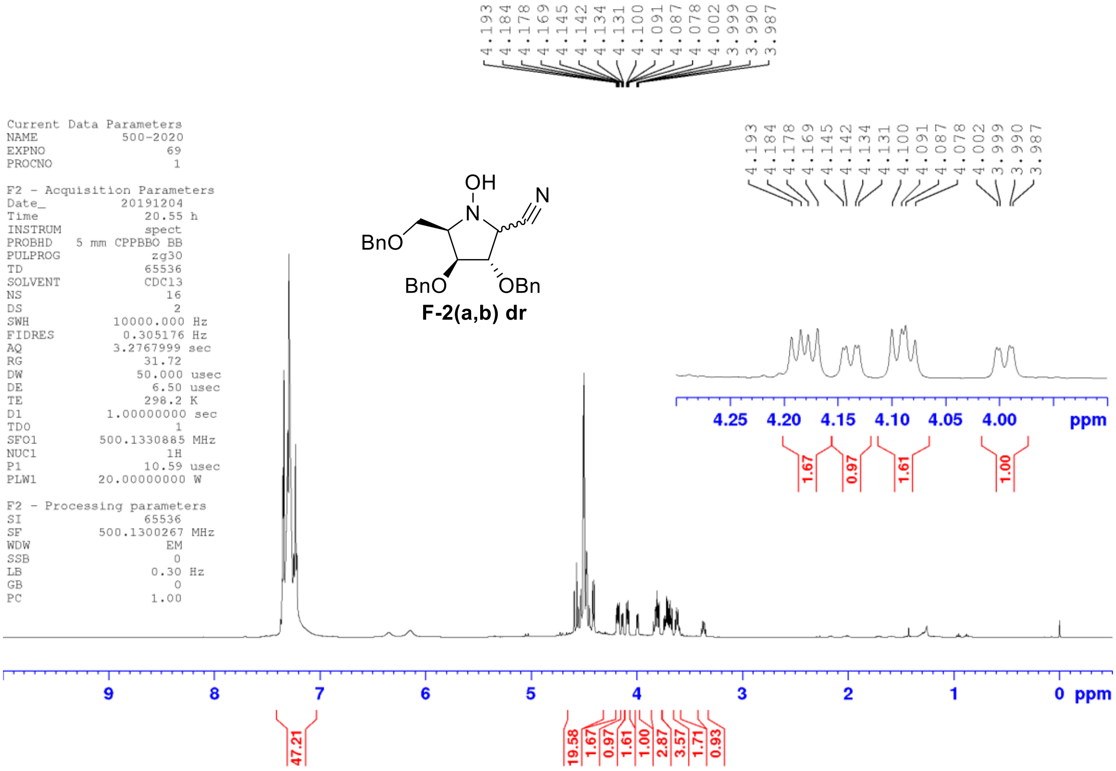

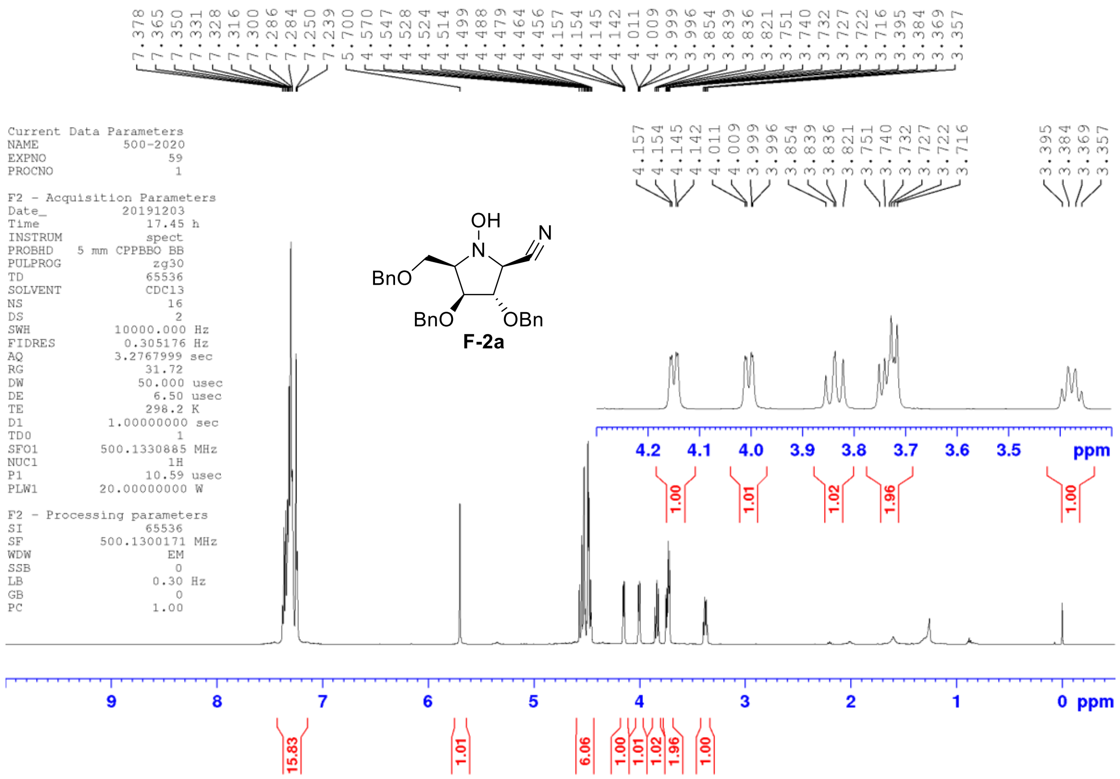

Current Data Parameters
NAME 500-2020
EXPNO 59
PROCNO 1
F2 - Acquisition Parameters
Date_ 20191203
Time 17.45 h
INSTRUM spect
PROBHD 5 mm CPPBBO BB
PULPROG zg30
TD 65536
SOLVENT CDCl3
NS 16
DS 2
SWH 10000.000 Hz
FIDRES 0.305176 Hz
AQ 3.2767999 sec
RG 31.72
DW 50.000 usec
DE 6.50 usec
TE 298.2 K
D1 1.00000000 sec
TD0 1
SFO1 500.1330885 MHz
NUC1 1H
P1 10.59 usec
PLW1 20.00000000 W
F2 - Processing parameters
SI 65536
SF 500.1300171 MHz
WDW EM
SSB 0
LB 0.30 Hz
GB 0
PC 1.00
OH
N
BnO
BnO
OBn
F-2a
ppm

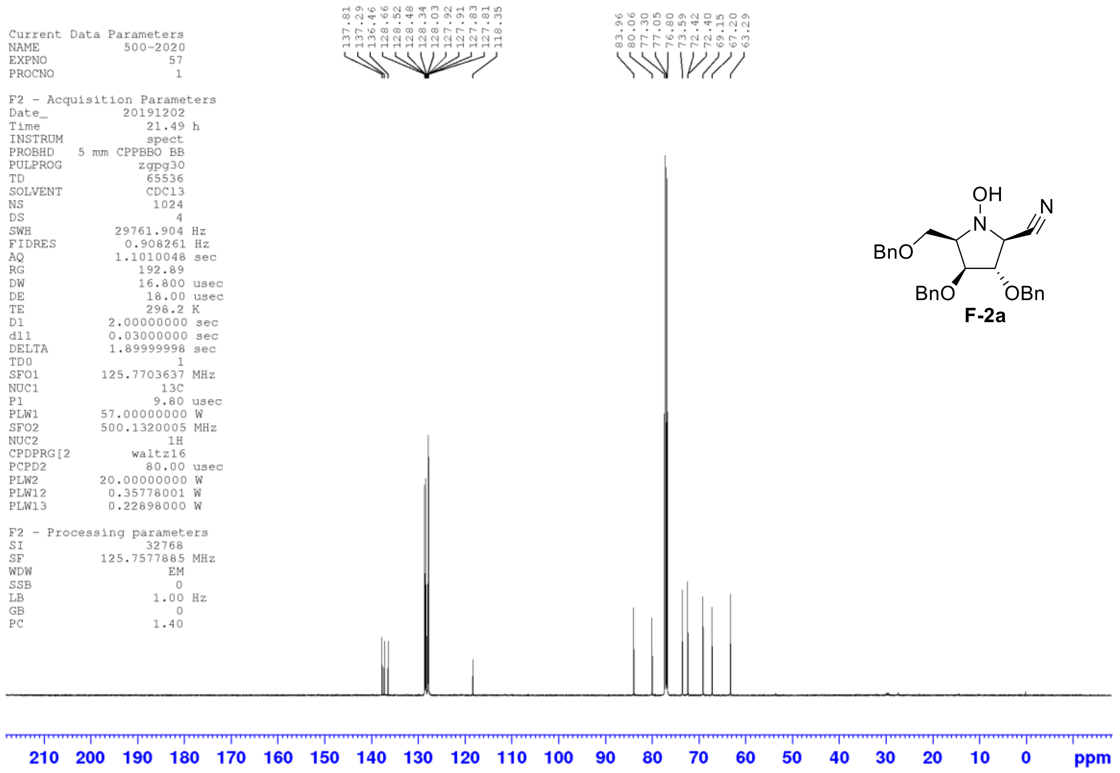

Current Data Parameters
NAME 500-2020
EXPNO 57
PROCNO 1
F2 - Acquisition Parameters
Date_ 20191202
Time 21.49 h
INSTRUM spect
PROBHD 5 mm CPPBBO BB
PULPROG zgpg30
TD 65536
SOLVENT CDCl3
NS 1024
DS 4
SWH 29761.904 Hz
FIDRES 0.908261 Hz
AQ 1.1010048 sec
RG 192.89
DW 16.800 usec
DE 18.00 usec
TE 298.2 K
D1 2.00000000 sec
d11 0.03000000 sec
DELTA 1.89999998 sec
TD0 1
SFO1 125.7703637 MHz
NUC1 13C
P1 9.80 usec
PLW1 57.00000000 W
SFO2 500.1320005 MHz
NUC2 1H
CPDPRG[2 waltz16
PCPD2 80.00 usec
PLW2 20.00000000 W
PLW12 0.35778001 W
PLW13 0.22898000 W
F2 - Processing parameters
SI 32768
SF 125.7577885 MHz
WDW EM
SSB 0
LB 1.00 Hz
GB 0
PC 1.40
OH
N
BnO
BnO
OBn
F-2a
ppm

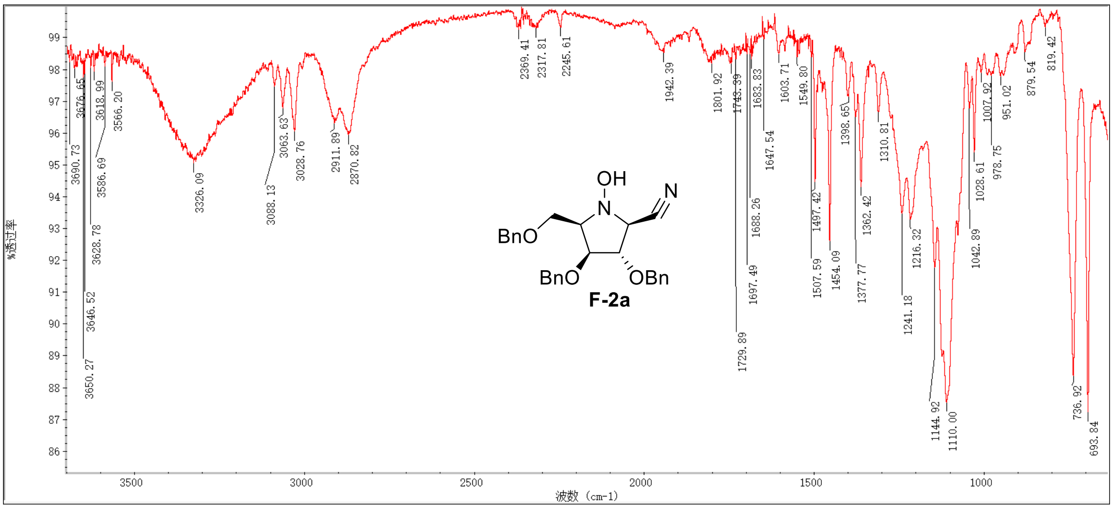

Compound **F-2b**:

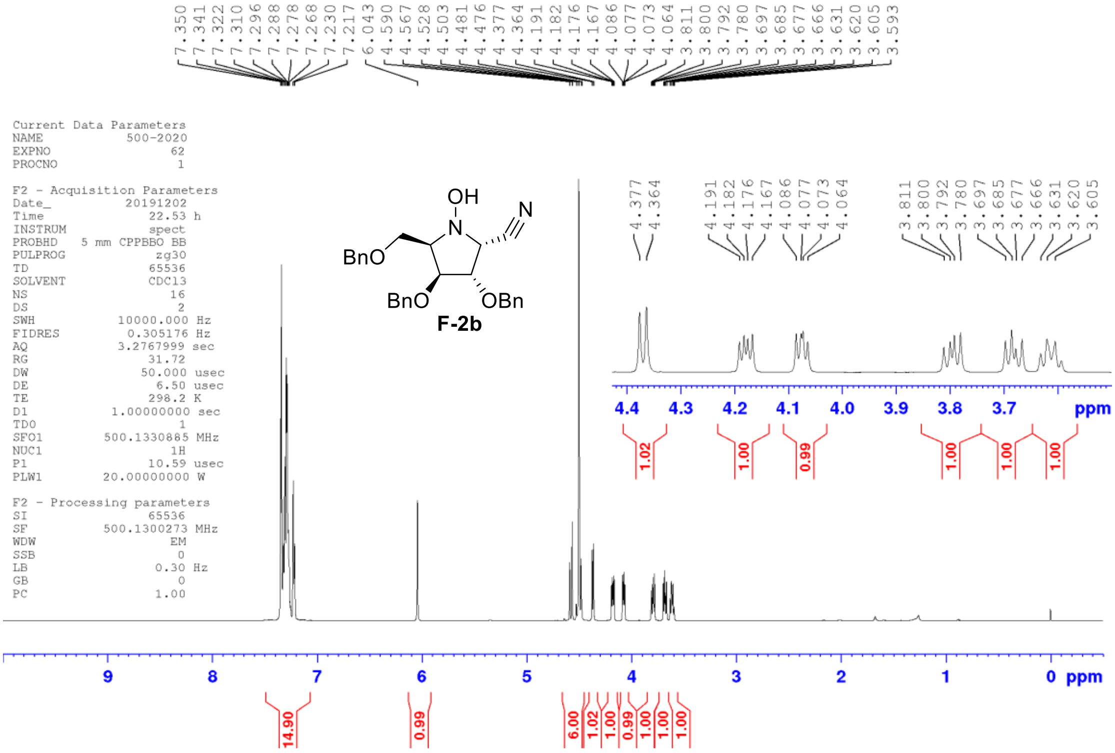

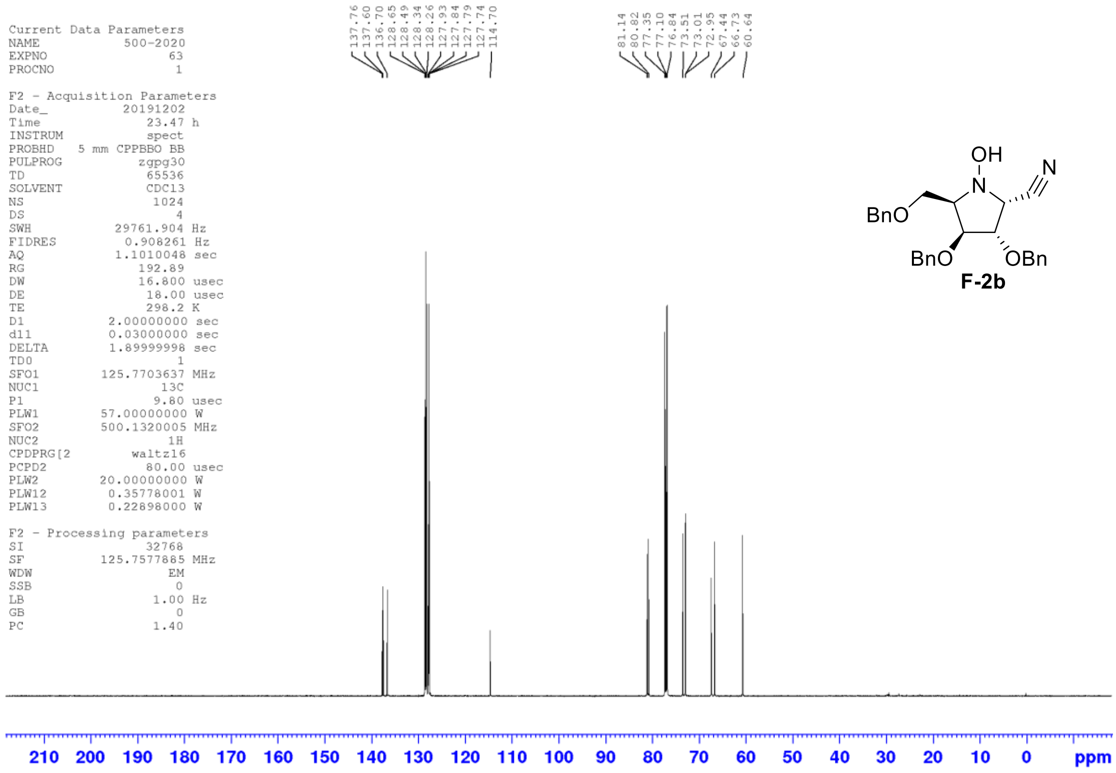
Current Data Parameters
NAME 500-2020
EXPNO 63
PROCNO 1
F2 - Acquisition Parameters
Date_ 20191202
Time 23.47 h
INSTRUM spect
PROBHD 5 mm CPPBBO BB
PULPROG zgpg30
TD 65536
SOLVENT CDCl3
NS 1024
DS 4
SWH 29761.904 Hz
FIDRES 0.908261 Hz
AQ 1.1010048 sec
RG 192.89
DW 16.800 usec
DE 18.00 usec
TE 298.2 K
D1 2.00000000 sec
d11 0.03000000 sec
DELTA 1.89999998 sec
TD0 1
SFO1 125.7703637 MHz
NUC1 13C
P1 9.80 usec
PLW1 57.00000000 W
SFO2 500.1320005 MHz
NUC2 1H
CPDPRG[2 waltz16
PCPD2 80.00 usec
PLW2 20.00000000 W
PLW12 0.35778001 W
PLW13 0.22898000 W
F2 - Processing parameters
SI 32768
SF 125.7577885 MHz
WDW EM
SSB 0
LB 1.00 Hz
GB 0
PC 1.40
137.76
137.60
136.70
128.65
128.49
128.34
128.26
127.93
127.84
127.79
127.74
114.70
81.14
80.82
77.35
77.10
76.84
73.51
73.01
72.95
67.44
66.73
60.64
OH
N
N
BnO
BnO
OBn
F-2b
210 200 190 180 170 160 150 140 130 120 110 100 90 80 70 60 50 40 30 20 10 0 ppm

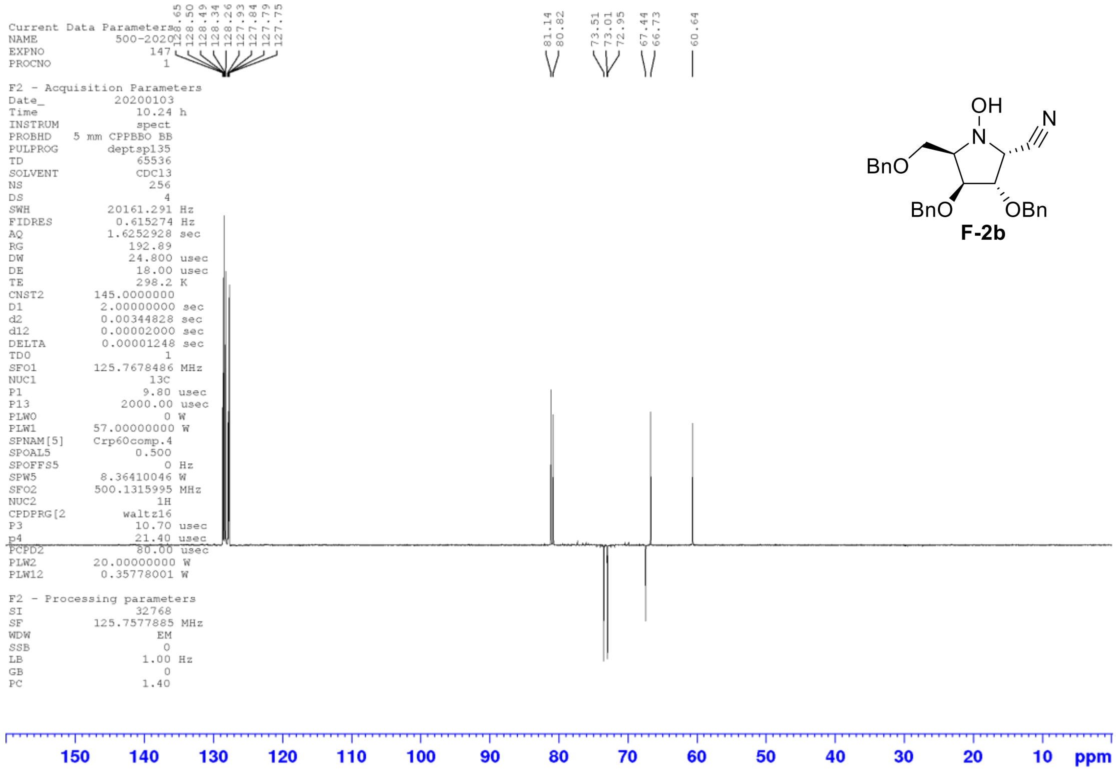
Current Data Parameters
NAME 500-2020
EXPNO 147
PROCNO 1
F2 - Acquisition Parameters
Date_ 20200103
Time 10.24 h
INSTRUM spect
PROBHD 5 mm CPPBBO BB
PULPROG deptsp135
TD 65536
SOLVENT CDCl3
NS 256
DS 4
SWH 20161.291 Hz
FIDRES 0.615274 Hz
AQ 1.6252928 sec
RG 192.89
DW 24.800 usec
DE 18.00 usec
TE 298.2 K
CNST2 145.0000000
D1 2.00000000 sec
d2 0.00344828 sec
d12 0.00002000 sec
DELTA 0.00001248 sec
TD0 1
SFO1 125.7678486 MHz
NUC1 13C
P1 9.80 usec
P13 2000.00 usec
PLW0 0 W
PLW1 57.00000000 W
SPNAM[5] Crp60comp.4
SPOAL5 0.500
SPOFFS5 0 Hz
SPW5 8.36410046 W
SFO2 500.1315995 MHz
NUC2 1H
CPDPRG[2 waltz16
P3 10.70 usec
p4 21.40 usec
PCPD2 80.00 usec
PLW2 20.00000000 W
PLW12 0.35778001 W
F2 - Processing parameters
SI 32768
SF 125.7577885 MHz
WDW EM
SSB 0
LB 1.00 Hz
GB 0
PC 1.40
128.65
128.50
128.49
128.34
128.26
127.93
127.84
127.79
127.75
81.14
80.82
73.51
73.01
72.95
67.44
66.73
60.64
OH
N
N
BnO
BnO
OBn
F-2b
150 140 130 120 110 100 90 80 70 60 50 40 30 20 10 ppm

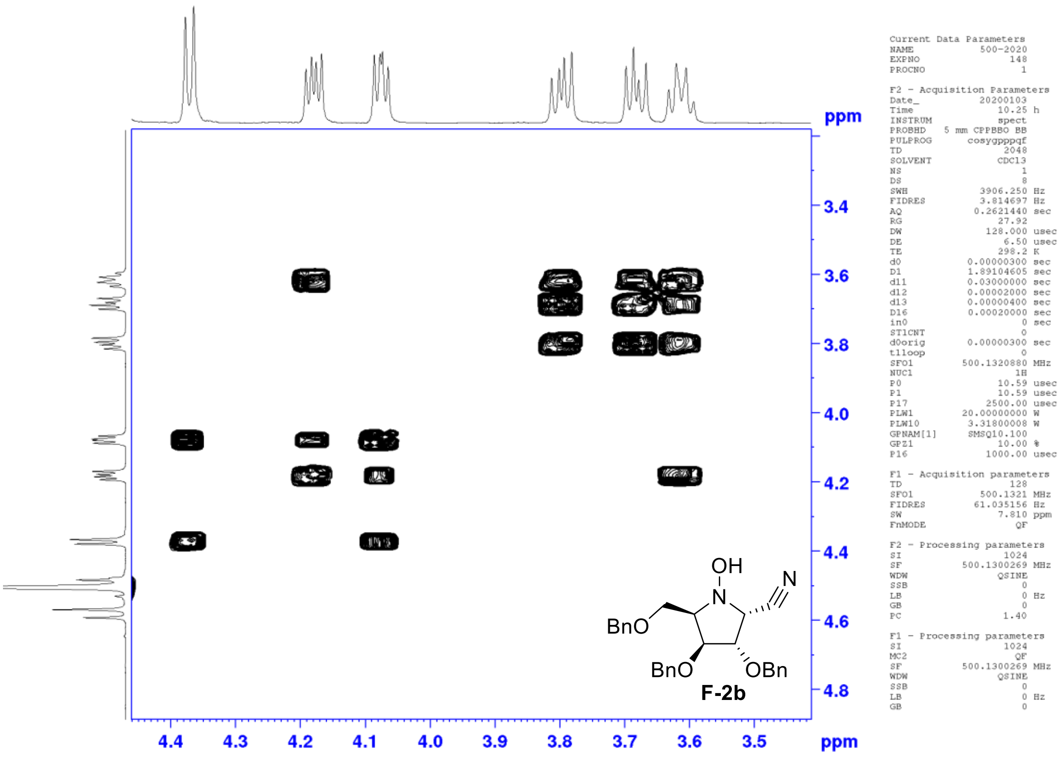
Current Data Parameters
NAME 500-2020
EXPNO 148
PROCNO 1
F2 - Acquisition Parameters
Date_ 20200103
Time 10.25 h
INSTRUM spect
PROBHD 5 mm CPPBBO BB
PULPROG cosygpppqf
TD 2048
SOLVENT CDCl3
NS 1
DS 8
SWH 3906.250 Hz
FIDRES 3.814697 Hz
AQ 0.2621440 sec
RG 27.92
DW 128.000 usec
DE 6.50 usec
TE 298.2 K
d0 0.00000300 sec
D1 1.89104605 sec
d11 0.03000000 sec
d12 0.00002000 sec
d13 0.00000400 sec
D16 0.00020000 sec
in0 0 sec
ST1CNT 0
d0orig 0.00000300 sec
t1loop 0
SFO1 500.1320880 MHz
NUC1 1H
P0 10.59 usec
P1 10.59 usec
P17 2500.00 usec
PLW1 20.00000000 W
PLW10 3.31800008 W
GPNAM[1] SMSQ10.100
GPZ1 10.00 %
P16 1000.00 usec
F1 - Acquisition parameters
TD 128
SFO1 500.1321 MHz
FIDRES 61.035156 Hz
SW 7.810 ppm
FnMODE QF
F2 - Processing parameters
SI 1024
SF 500.1300269 MHz
WDW QSINE
SSB 0
LB 0 Hz
GB 0
PC 1.40
F1 - Processing parameters
SI 1024
MC2 QF
SF 500.1300269 MHz
WDW QSINE
SSB 0
LB 0 Hz
GB 0
ppm
3.4
3.6
3.8
4.0
4.2
4.4
4.6
4.8
4.4 4.3 4.2 4.1 4.0 3.9 3.8 3.7 3.6 3.5
OH
N
BnO
BnO
OBn
F-2b

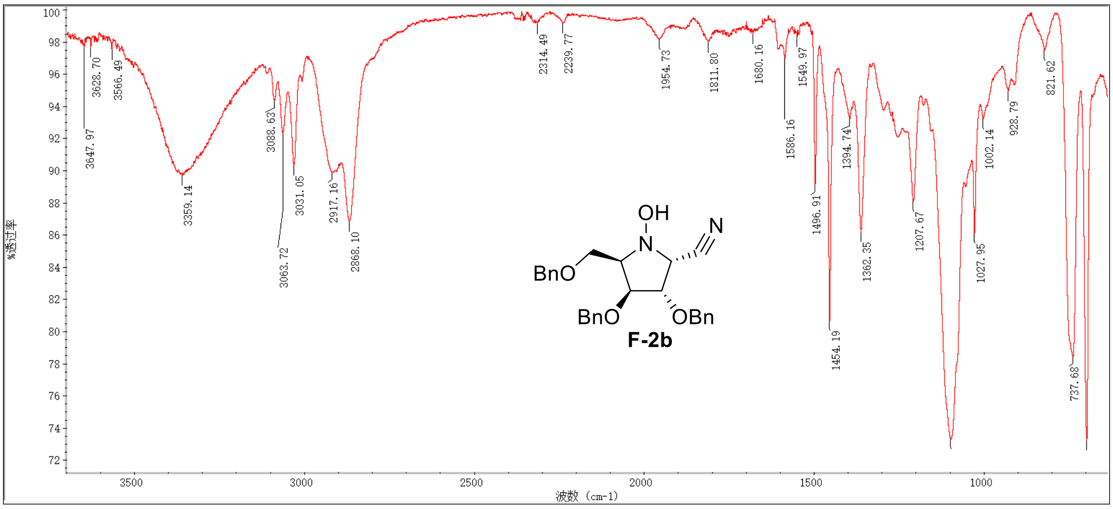
%透过率
100
98
96
94
92
90
88
86
84
82
80
78
76
74
72
3647.97
3628.70
3566.49
3359.14
3088.63
3063.72
3031.05
2917.16
2868.10
2314.49
2239.77
1954.73
1811.80
1680.16
1586.16
1549.97
1496.91
1454.19
1394.74
1362.35
1207.67
1027.95
1002.14
928.79
821.62
737.68
3500
3000
2500
2000
1500
1000
波数（cm-1）
OH
N
BnO
BnO
OBn
F-2b

Compound **G-2**:

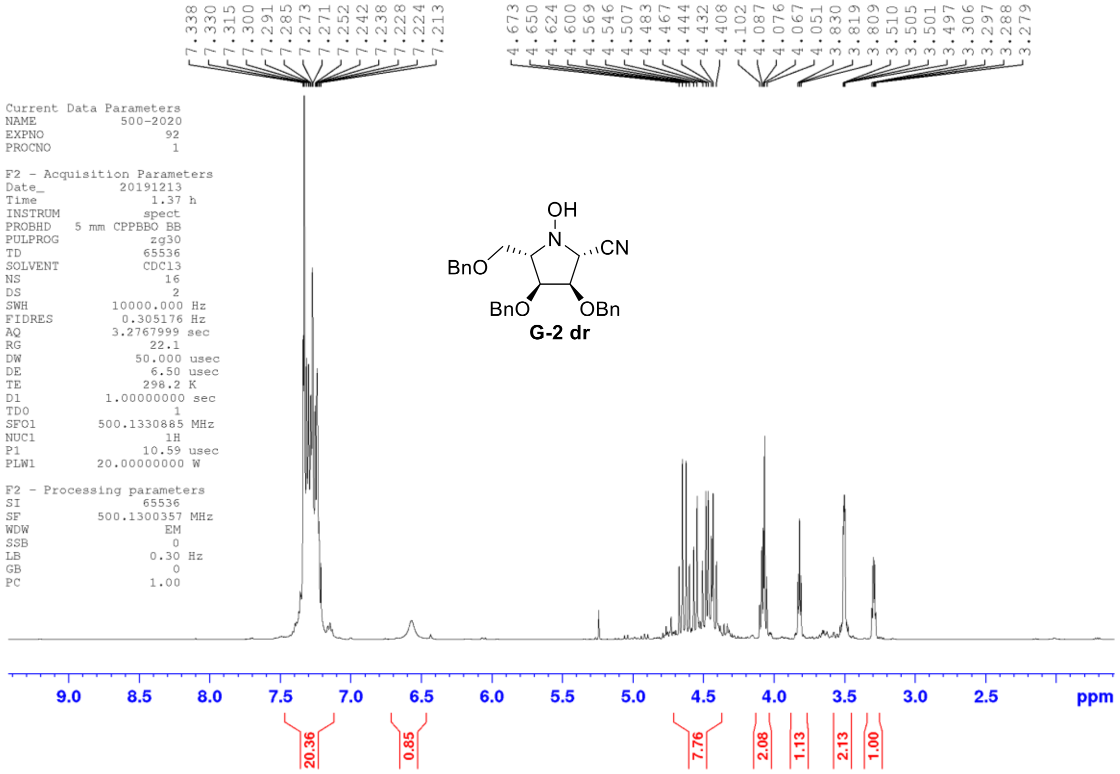
7.338 7.330 7.315 7.300 7.291 7.285 7.273 7.271 7.252 7.242 7.238 7.228 7.224 7.213
4.673 4.650 4.624 4.600 4.569 4.546 4.507 4.483 4.467 4.444 4.432 4.408 4.102 4.087 4.076 4.067 4.051 3.830 3.819 3.809 3.510 3.505 3.501 3.497 3.306 3.297 3.288 3.279
Current Data Parameters
NAME 500-2020
EXPNO 92
PROCNO 1
F2 - Acquisition Parameters
Date_ 20191213
Time 1.37 h
INSTRUM spect
PROBHD 5 mm CPPBBO BB
PULPROG zg30
TD 65536
SOLVENT CDCl3
NS 16
DS 2
SWH 10000.000 Hz
FIDRES 0.305176 Hz
AQ 3.2767999 sec
RG 22.1
DW 50.000 usec
DE 6.50 usec
TE 298.2 K
D1 1.00000000 sec
TD0 1
SFO1 500.1330885 MHz
NUC1 1H
P1 10.59 usec
PLW1 20.00000000 W
F2 - Processing parameters
SI 65536
SF 500.1300357 MHz
WDW EM
SSB 0
LB 0.30 Hz
GB 0
PC 1.00
OH
N
CN
BnO
BnO
OBn
G-2 dr
9.0 8.5 8.0 7.5 7.0 6.5 6.0 5.5 5.0 4.5 4.0 3.5 3.0 2.5 ppm
20.36 0.85 7.76 2.08 1.13 2.13 1.00

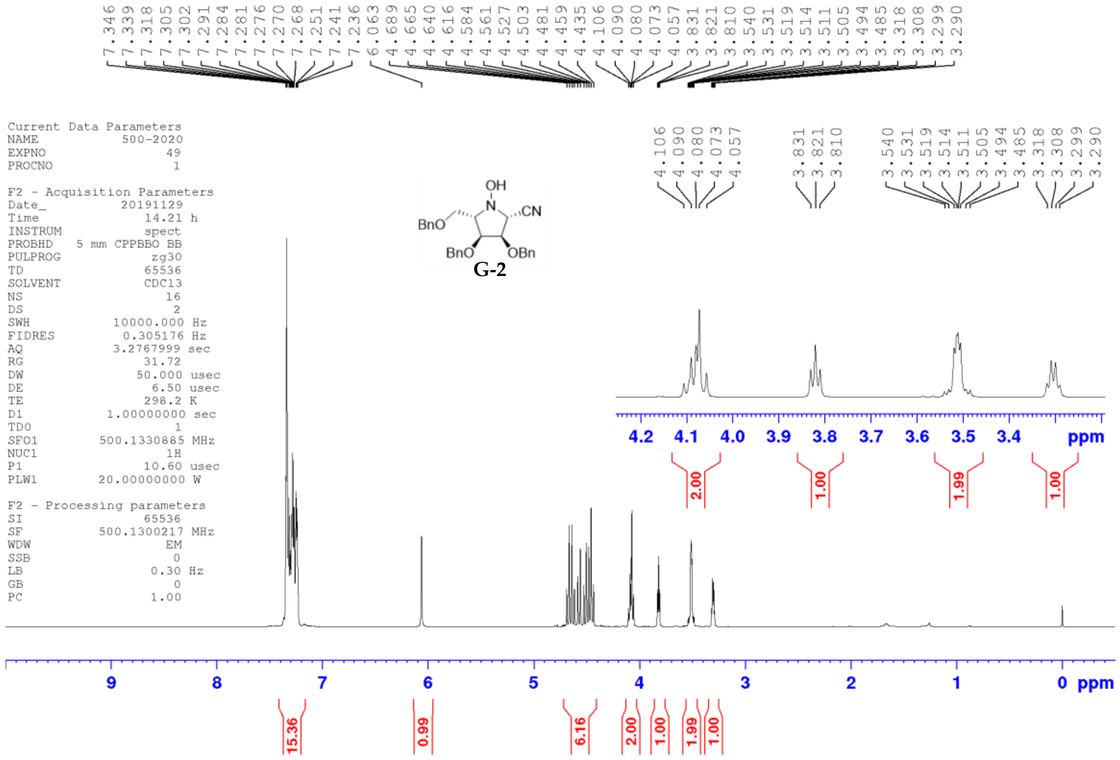
7.346 7.339 7.318 7.305 7.302 7.291 7.284 7.281 7.276 7.270 7.268 7.251 7.241 7.236 6.063 4.689 4.665 4.640 4.616 4.584 4.561 4.527 4.503 4.481 4.459 4.435 4.106 4.090 4.080 4.073 4.057 3.831 3.821 3.810 3.540 3.531 3.519 3.514 3.511 3.505 3.494 3.485 3.318 3.308 3.299 3.290
Current Data Parameters
NAME 500-2020
EXPNO 49
PROCNO 1
F2 - Acquisition Parameters
Date_ 20191129
Time 14.21 h
INSTRUM spect
PROBHD 5 mm CPPBBO BB
PULPROG zg30
TD 65536
SOLVENT CDCl3
NS 16
DS 2
SWH 10000.000 Hz
FIDRES 0.305176 Hz
AQ 3.2767999 sec
RG 31.72
DW 50.000 usec
DE 6.50 usec
TE 298.2 K
D1 1.00000000 sec
TD0 1
SFO1 500.1330885 MHz
NUC1 1H
P1 10.60 usec
PLW1 20.00000000 W
F2 - Processing parameters
SI 65536
SF 500.1300217 MHz
WDW EM
SSB 0
LB 0.30 Hz
GB 0
PC 1.00
OH
N
CN
BnO
BnO
OBn
G-2
4.106 4.090 4.080 4.073 4.057
3.831 3.821 3.810
3.540 3.531 3.519 3.514 3.511 3.505 3.494 3.485 3.318 3.308 3.299 3.290
4.2 4.1 4.0 3.9 3.8 3.7 3.6 3.5 3.4 ppm
2.00 1.00 1.99 1.00
9 8 7 6 5 4 3 2 1 0 ppm
15.36 0.99 6.16 2.00 1.00 1.99 1.00

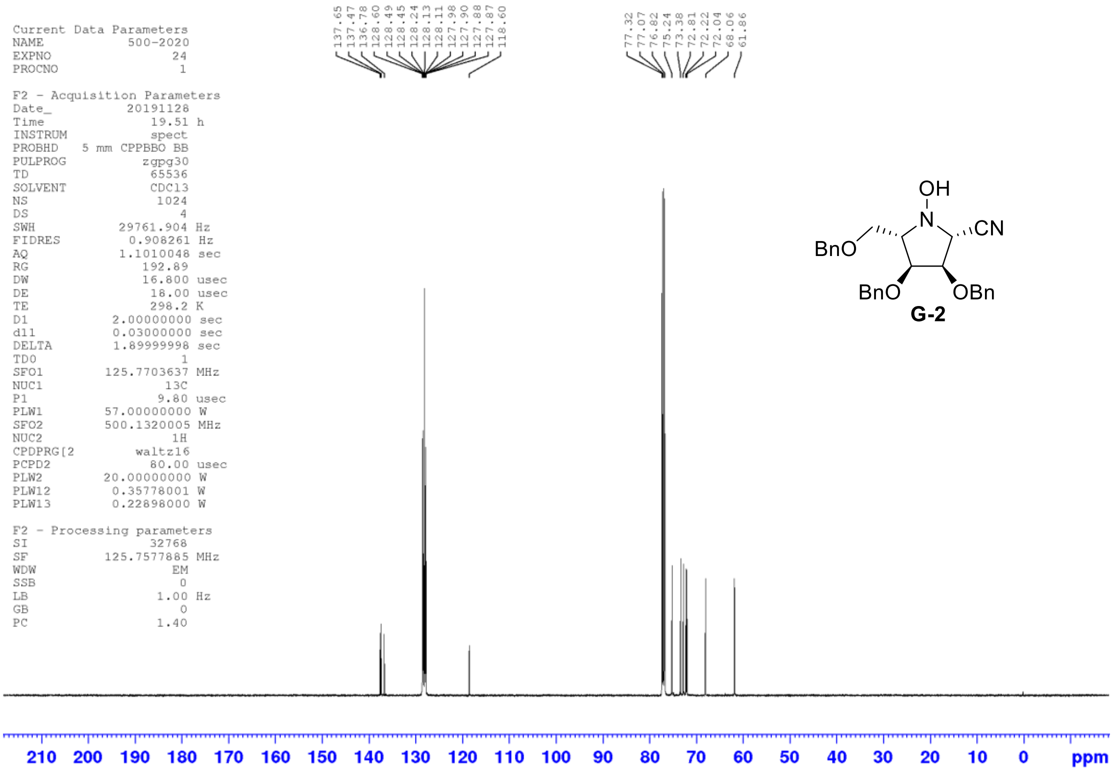

137.65
137.47
136.78
128.60
128.49
128.45
128.24
128.13
128.11
127.98
127.90
127.88
127.87
118.60
77.32
77.07
76.82
75.24
73.38
72.81
72.22
72.04
68.06
61.86
Current Data Parameters
NAME 500-2020
EXPNO 24
PROCNO 1
F2 - Acquisition Parameters
Date_ 20191128
Time 19.51 h
INSTRUM spect
PROBHD 5 mm CPPBBO BB
PULPROG zgpg30
TD 65536
SOLVENT CDCl3
NS 1024
DS 4
SWH 29761.904 Hz
FIDRES 0.908261 Hz
AQ 1.1010048 sec
RG 192.89
DW 16.800 usec
DE 18.00 usec
TE 298.2 K
D1 2.00000000 sec
d11 0.03000000 sec
DELTA 1.89999998 sec
TD0 1
SFO1 125.7703637 MHz
NUC1 13C
P1 9.80 usec
PLW1 57.00000000 W
SFO2 500.1320005 MHz
NUC2 1H
CPDPRG[2 waltz16
PCPD2 80.00 usec
PLW2 20.00000000 W
PLW12 0.35778001 W
PLW13 0.22898000 W
F2 - Processing parameters
SI 32768
SF 125.7577885 MHz
WDW EM
SSB 0
LB 1.00 Hz
GB 0
PC 1.40
OH
N
CN
BnO
BnO
OBn
G-2
210 200 190 180 170 160 150 140 130 120 110 100 90 80 70 60 50 40 30 20 10 0 ppm

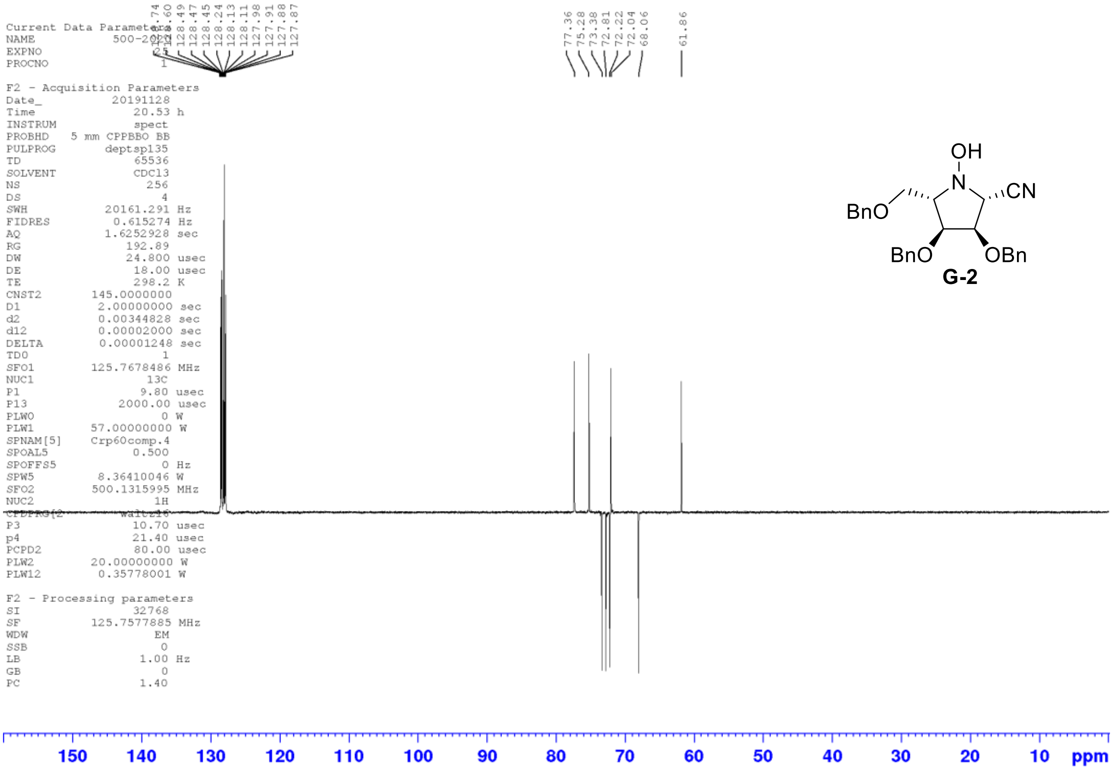

128.60
128.49
128.47
128.45
128.24
128.13
128.11
127.98
127.91
127.88
127.87
77.36
75.28
73.38
72.81
72.22
72.04
68.06
61.86
Current Data Parameters
NAME 500-2020
EXPNO
PROCNO 1
F2 - Acquisition Parameters
Date_ 20191128
Time 20.53 h
INSTRUM spect
PROBHD 5 mm CPPBBO BB
PULPROG deptsp135
TD 65536
SOLVENT CDCl3
NS 256
DS 4
SWH 20161.291 Hz
FIDRES 0.615274 Hz
AQ 1.6252928 sec
RG 192.89
DW 24.800 usec
DE 18.00 usec
TE 298.2 K
CNST2 145.0000000
D1 2.00000000 sec
d2 0.00344828 sec
d12 0.00002000 sec
DELTA 0.00001248 sec
TD0 1
SFO1 125.7678486 MHz
NUC1 13C
P1 9.80 usec
P13 2000.00 usec
PLW0 0 W
PLW1 57.00000000 W
SPNAM[5] Crp60comp.4
SPOAL5 0.500
SPOFFS5 0 Hz
SPW5 8.36410046 W
SFO2 500.1315995 MHz
NUC2 1H
P3 10.70 usec
p4 21.40 usec
PCPD2 80.00 usec
PLW2 20.00000000 W
PLW12 0.35778001 W
F2 - Processing parameters
SI 32768
SF 125.7577885 MHz
WDW EM
SSB 0
LB 1.00 Hz
GB 0
PC 1.40
OH
N
CN
BnO
BnO
OBn
G-2
150 140 130 120 110 100 90 80 70 60 50 40 30 20 10 ppm

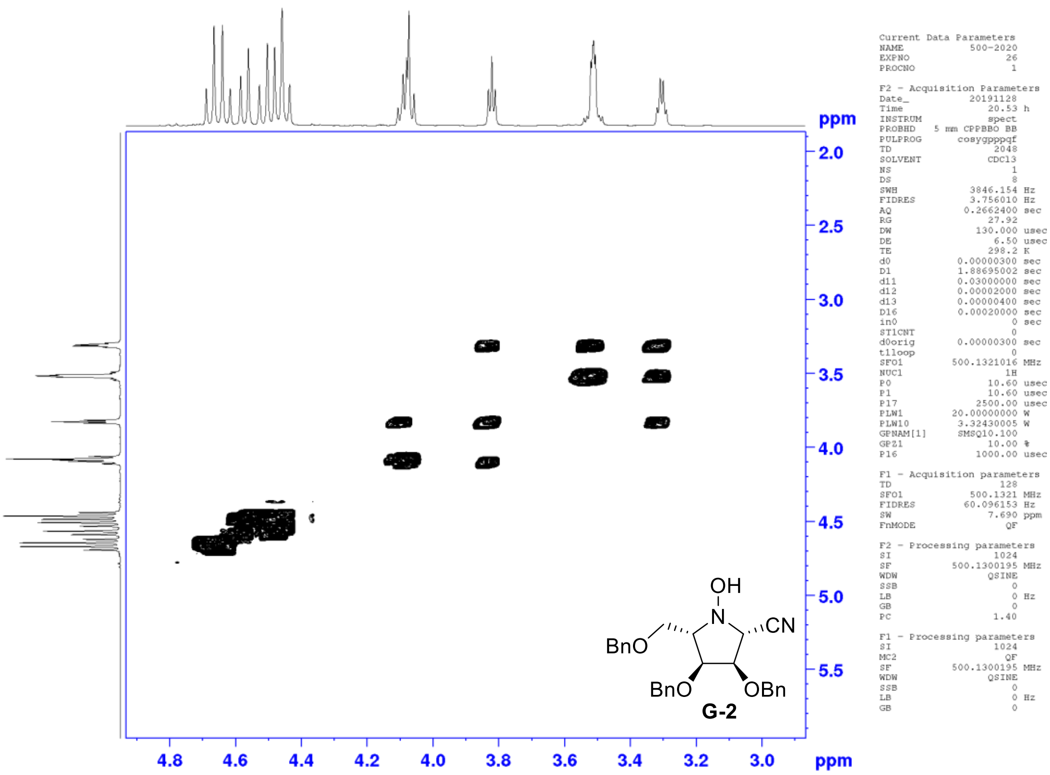

ppm
2.0
2.5
3.0
3.5
4.0
4.5
5.0
5.5
4.8
4.6
4.4
4.2
4.0
3.8
3.6
3.4
3.2
3.0
ppm
OH
N
CN
BnO
BnO
OBn
G-2
Current Data Parameters
NAME 500-2020
EXPNO 26
PROCNO 1
F2 - Acquisition Parameters
Date_ 20191128
Time 20.53 h
INSTRUM spect
PROBHD 5 mm CPPBBO BB
PULPROG cosygpppqf
TD 2048
SOLVENT CDCl3
NS 1
DS 8
SWH 3846.154 Hz
FIDRES 3.756010 Hz
AQ 0.2662400 sec
RG 27.92
DW 130.000 usec
DE 6.50 usec
TE 298.2 K
d0 0.00000300 sec
D1 1.88695002 sec
d11 0.03000000 sec
d12 0.00002000 sec
d13 0.00000400 sec
D16 0.00020000 sec
in0 0 sec
ST1CNT 0
d0orig 0.00000300 sec
t1loop 0
SFO1 500.1321016 MHz
NUC1 1H
P0 10.60 usec
P1 10.60 usec
P17 2500.00 usec
PLW1 20.00000000 W
PLW10 3.32430005 W
GPNAM[1] SMSQ10.100
GPZ1 10.00 %
P16 1000.00 usec
F1 - Acquisition parameters
TD 128
SFO1 500.1321 MHz
FIDRES 60.096153 Hz
SW 7.690 ppm
FnMODE QF
F2 - Processing parameters
SI 1024
SF 500.1300195 MHz
WDW QSINE
SSB 0
LB 0 Hz
GB 0
PC 1.40
F1 - Processing parameters
SI 1024
MC2 QF
SF 500.1300195 MHz
WDW QSINE
SSB 0
LB 0 Hz
GB 0

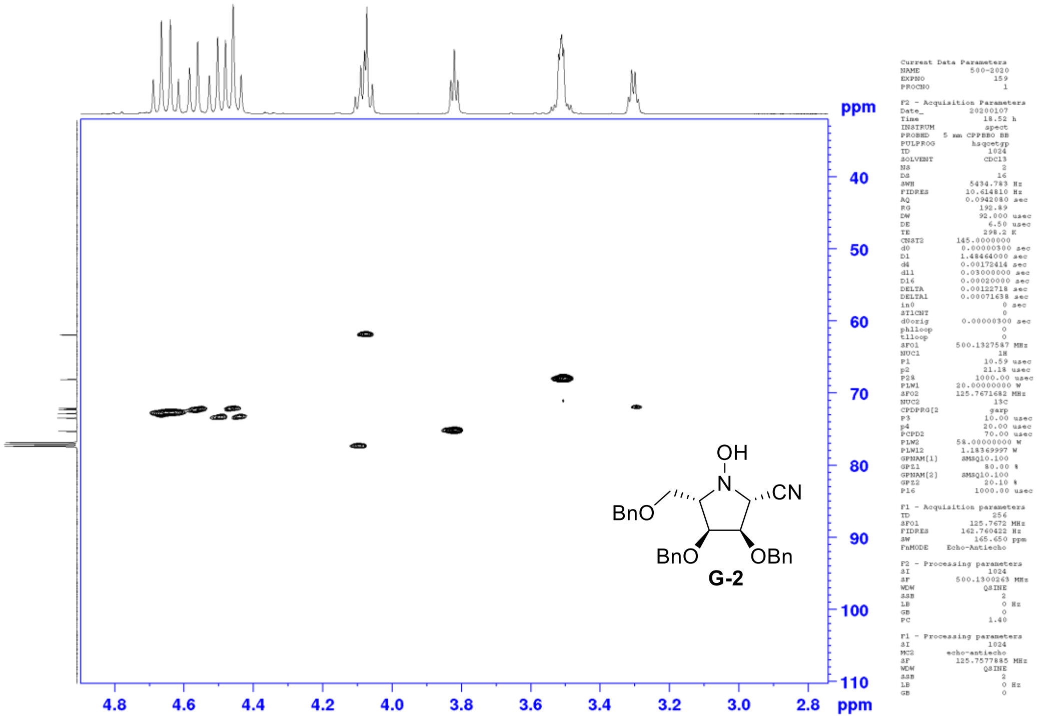

ppm
40
50
60
70
80
90
100
110
4.8
4.6
4.4
4.2
4.0
3.8
3.6
3.4
3.2
3.0
2.8
ppm
OH
N
CN
BnO
BnO
OBn
G-2
Current Data Parameters
NAME 500-2020
EXPNO 159
PROCNO 1
F2 - Acquisition Parameters
Date_ 20200107
Time 18.52 h
INSTRUM spect
PROBHD 5 mm CPPBBO BB
PULPROG hsqcetgp
TD 1024
SOLVENT CDCl3
NS 2
DS 16
SWH 5434.783 Hz
FIDRES 10.614810 Hz
AQ 0.0942080 sec
RG 192.89
DW 92.000 usec
DE 6.50 usec
TE 298.2 K
CNST2 145.0000000
d0 0.00000300 sec
D1 1.48464000 sec
d4 0.00172414 sec
d11 0.03000000 sec
D16 0.00020000 sec
DELTA 0.00122718 sec
DELTA1 0.00071638 sec
in0 0 sec
ST1CNT 0
d0orig 0.00000300 sec
ph1loop 0
t1loop 0
SFO1 500.1327587 MHz
NUC1 1H
P1 10.59 usec
p2 21.18 usec
P28 1000.00 usec
PLW1 20.00000000 W
SFO2 125.7671682 MHz
NUC2 13C
CPDPRG[2 garp
P3 10.00 usec
p4 20.00 usec
PCPD2 70.00 usec
PLW2 58.00000000 W
PLW12 1.18369997 W
GPNAM[1] SMSQ10.100
GPZ1 80.00 %
GPNAM[2] SMSQ10.100
GPZ2 20.10 %
P16 1000.00 usec
F1 - Acquisition parameters
TD 256
SFO1 125.7672 MHz
FIDRES 162.760422 Hz
SW 165.650 ppm
FnMODE Echo-Antiecho
F2 - Processing parameters
SI 1024
SF 500.1300263 MHz
WDW QSINE
SSB 2
LB 0 Hz
GB 0
PC 1.40
F1 - Processing parameters
SI 1024
MC2 echo-antiecho
SF 125.7577885 MHz
WDW QSINE
SSB 2
LB 0 Hz
GB 0

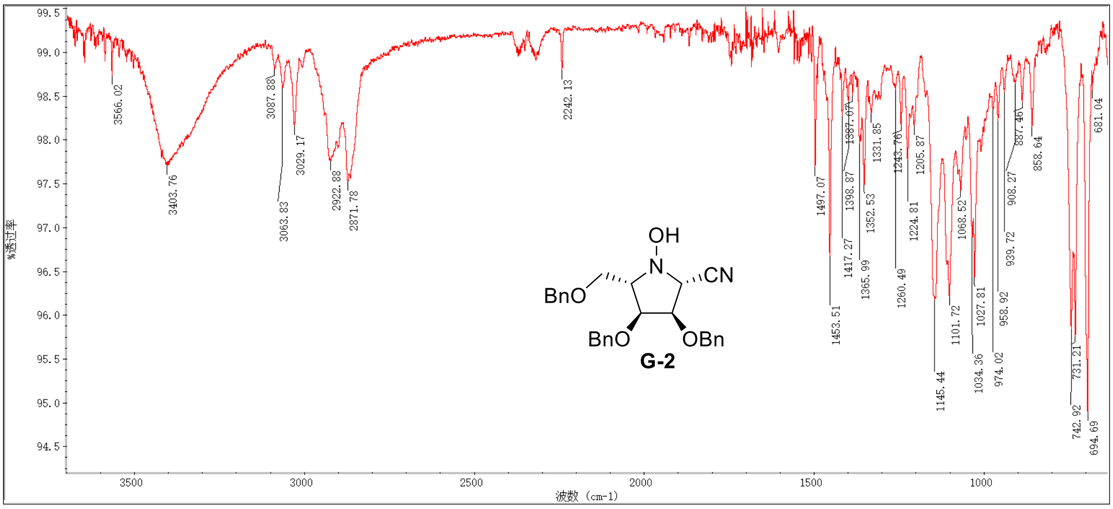

%透过率
波数 (cm-1)
3566.02
3403.76
3087.88
3063.83
3029.17
2922.88
2871.78
2242.13
1497.07
1453.51
1417.27
1398.87
1387.07
1365.99
1352.53
1331.85
1260.49
1243.76
1224.81
1205.87
1145.44
1101.72
1068.52
1034.36
1027.81
974.02
958.92
939.72
908.27
887.46
858.64
742.92
731.21
694.69
681.04
OH
N
CN
BnO
BnO
OBn
G-2

Compound **H-2**:

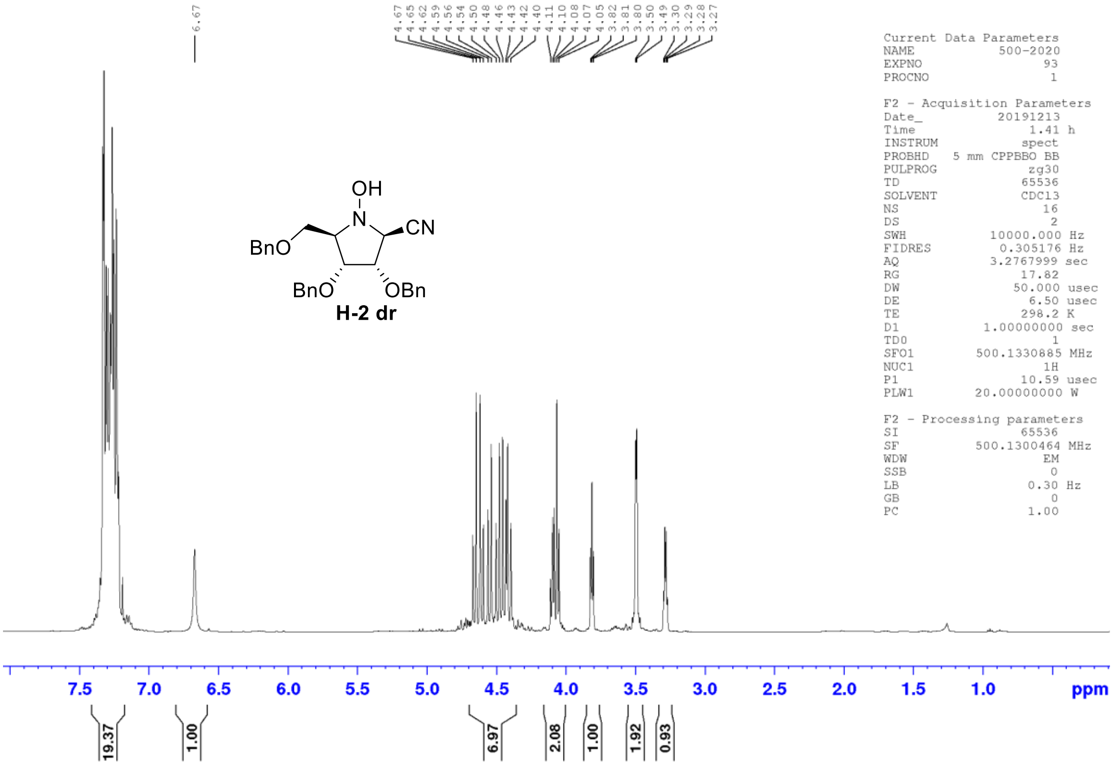

6.67
4.67 4.65 4.62 4.59 4.56 4.54 4.50 4.48 4.46 4.43 4.42 4.40 4.11 4.10 4.08 4.07 4.05 3.82 3.81 3.80 3.50 3.49 3.30 3.29 3.28 3.27
OH
N
CN
BnO
BnO
OBn
H-2 dr
Current Data Parameters
NAME 500-2020
EXPNO 93
PROCNO 1
F2 - Acquisition Parameters
Date_ 20191213
Time 1.41 h
INSTRUM spect
PROBHD 5 mm CPPBBO BB
PULPROG zg30
TD 65536
SOLVENT CDCl3
NS 16
DS 2
SWH 10000.000 Hz
FIDRES 0.305176 Hz
AQ 3.2767999 sec
RG 17.82
DW 50.000 usec
DE 6.50 usec
TE 298.2 K
D1 1.00000000 sec
TD0 1
SFO1 500.1330885 MHz
NUC1 1H
P1 10.59 usec
PLW1 20.00000000 W
F2 - Processing parameters
SI 65536
SF 500.1300464 MHz
WDW EM
SSB 0
LB 0.30 Hz
GB 0
PC 1.00
7.5 7.0 6.5 6.0 5.5 5.0 4.5 4.0 3.5 3.0 2.5 2.0 1.5 1.0 ppm
19.37
1.00
6.97
2.08
1.00
1.92
0.93

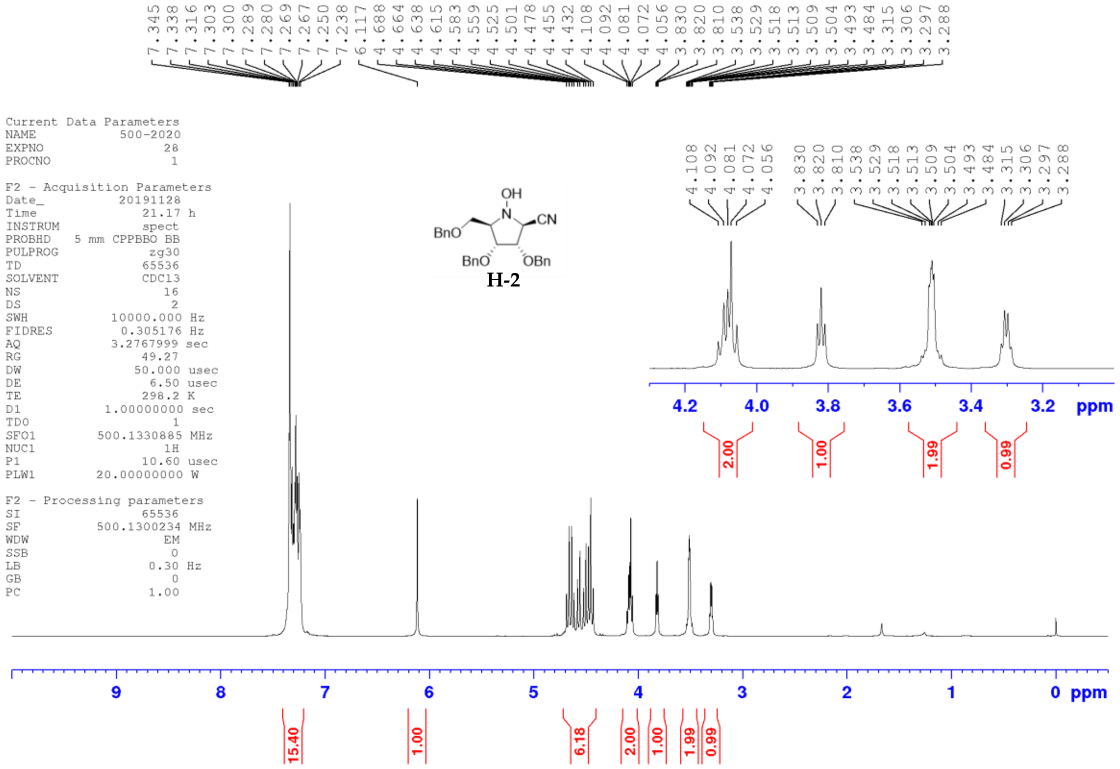
7.345 7.338 7.316 7.303 7.300 7.289 7.280 7.269 7.267 7.250 7.238 6.117 4.688 4.664 4.638 4.615 4.583 4.559 4.525 4.501 4.478 4.455 4.432 4.108 4.092 4.081 4.072 4.056 3.830 3.820 3.810 3.538 3.529 3.518 3.513 3.509 3.504 3.493 3.484 3.315 3.306 3.297 3.288
Current Data Parameters
NAME 500-2020
EXPNO 28
PROCNO 1
F2 - Acquisition Parameters
Date_ 20191128
Time 21.17 h
INSTRUM spect
PROBHD 5 mm CPPBBO BB
PULPROG zg30
TD 65536
SOLVENT CDCl3
NS 16
DS 2
SWH 10000.000 Hz
FIDRES 0.305176 Hz
AQ 3.2767999 sec
RG 49.27
DW 50.000 usec
DE 6.50 usec
TE 298.2 K
D1 1.00000000 sec
TD0 1
SFO1 500.1330885 MHz
NUC1 1H
P1 10.60 usec
PLW1 20.00000000 W
F2 - Processing parameters
SI 65536
SF 500.1300234 MHz
WDW EM
SSB 0
LB 0.30 Hz
GB 0
PC 1.00
OH
N
CN
BnO
BnO
OBn
H-2
4.2 4.0 3.8 3.6 3.4 3.2 ppm
2.00 1.00 1.99 0.99
9 8 7 6 5 4 3 2 1 0 ppm
15.40 1.00 6.18 2.00 1.00 1.99 0.99

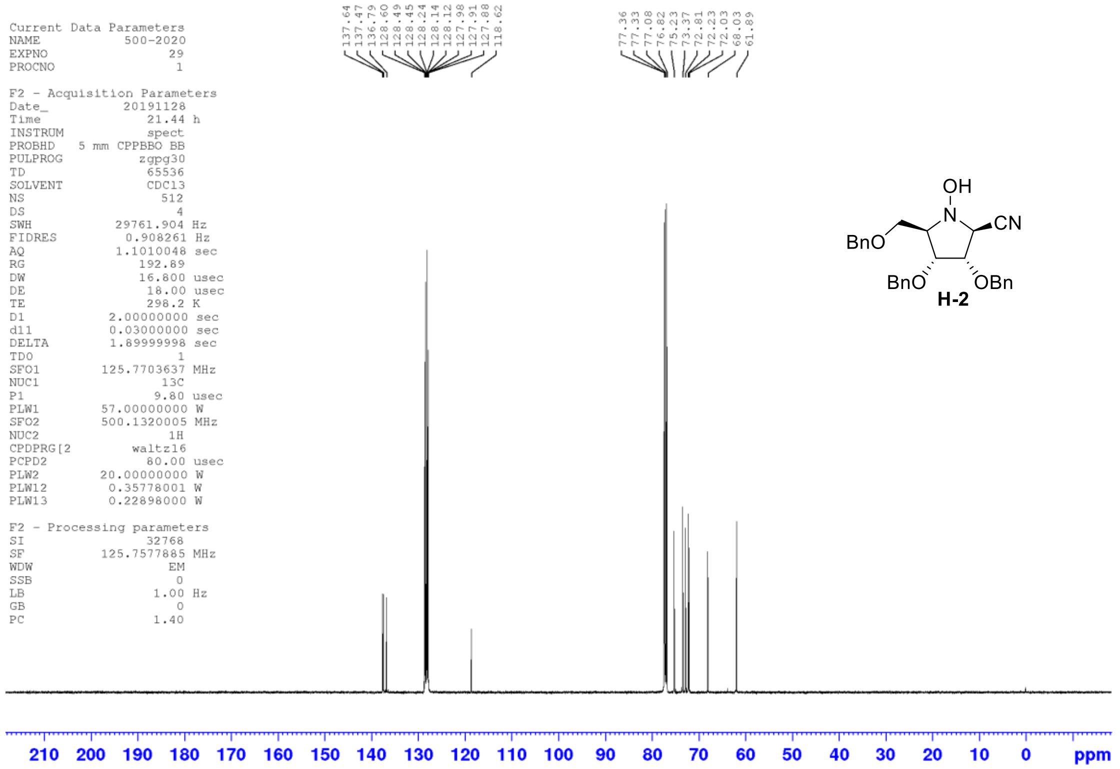
137.64 137.47 136.79 128.60 128.49 128.45 128.24 128.14 128.12 127.98 127.91 127.88 118.62
77.36 77.33 77.08 76.82 75.23 73.37 72.81 72.23 72.03 68.03 61.89
Current Data Parameters
NAME 500-2020
EXPNO 29
PROCNO 1
F2 - Acquisition Parameters
Date_ 20191128
Time 21.44 h
INSTRUM spect
PROBHD 5 mm CPPBBO BB
PULPROG zgpg30
TD 65536
SOLVENT CDCl3
NS 512
DS 4
SWH 29761.904 Hz
FIDRES 0.908261 Hz
AQ 1.1010048 sec
RG 192.89
DW 16.800 usec
DE 18.00 usec
TE 298.2 K
D1 2.00000000 sec
d11 0.03000000 sec
DELTA 1.89999998 sec
TD0 1
SFO1 125.7703637 MHz
NUC1 13C
P1 9.80 usec
PLW1 57.00000000 W
SFO2 500.1320005 MHz
NUC2 1H
CPDPRG[2 waltz16
PCPD2 80.00 usec
PLW2 20.00000000 W
PLW12 0.35778001 W
PLW13 0.22898000 W
F2 - Processing parameters
SI 32768
SF 125.7577885 MHz
WDW EM
SSB 0
LB 1.00 Hz
GB 0
PC 1.40
OH
N
CN
BnO
BnO
OBn
H-2
210 200 190 180 170 160 150 140 130 120 110 100 90 80 70 60 50 40 30 20 10 0 ppm

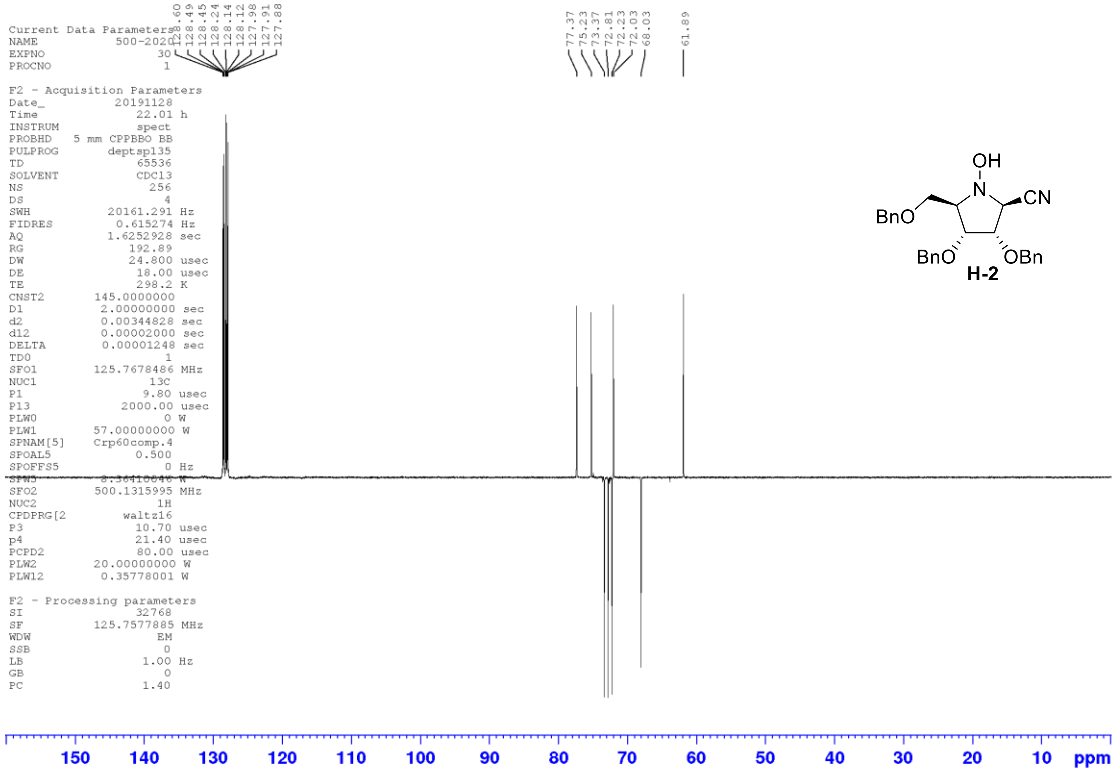

128.60
128.49
128.45
128.24
128.14
128.12
127.98
127.91
127.88
77.37
75.23
73.37
72.81
72.23
72.03
68.03
61.89
Current Data Parameters
NAME 500-2020
EXPNO 30
PROCNO 1
F2 - Acquisition Parameters
Date_ 20191128
Time 22.01 h
INSTRUM spect
PROBHD 5 mm CPPBBO BB
PULPROG deptsp135
TD 65536
SOLVENT CDCl3
NS 256
DS 4
SWH 20161.291 Hz
FIDRES 0.615274 Hz
AQ 1.6252928 sec
RG 192.89
DW 24.800 usec
DE 18.00 usec
TE 298.2 K
CNST2 145.0000000
D1 2.00000000 sec
d2 0.00344828 sec
d12 0.00002000 sec
DELTA 0.00001248 sec
TD0 1
SFO1 125.7678486 MHz
NUC1 13C
P1 9.80 usec
P13 2000.00 usec
PLW0 0 W
PLW1 57.00000000 W
SPNAM[5] Crp60comp.4
SPOAL5 0.500
SPOFFS5 0 Hz
SFO2 500.1315995 MHz
NUC2 1H
CPDPRG[2 waltz16
P3 10.70 usec
p4 21.40 usec
PCPD2 80.00 usec
PLW2 20.00000000 W
PLW12 0.35778001 W
F2 - Processing parameters
SI 32768
SF 125.7577885 MHz
WDW EM
SSB 0
LB 1.00 Hz
GB 0
PC 1.40
OH
N
CN
BnO
BnO
OBn
H-2
150 140 130 120 110 100 90 80 70 60 50 40 30 20 10 ppm

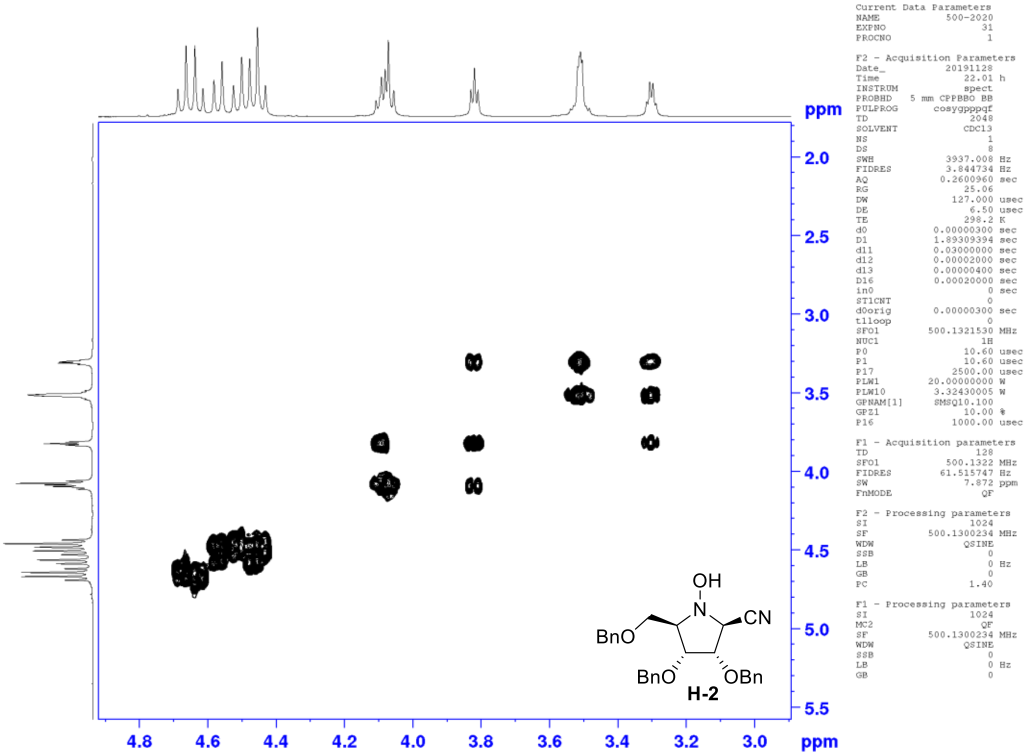

ppm
2.0 2.5 3.0 3.5 4.0 4.5 5.0 5.5
4.8 4.6 4.4 4.2 4.0 3.8 3.6 3.4 3.2 3.0 ppm
OH
N
CN
BnO
BnO
OBn
H-2
Current Data Parameters
NAME 500-2020
EXPNO 31
PROCNO 1
F2 - Acquisition Parameters
Date_ 20191128
Time 22.01 h
INSTRUM spect
PROBHD 5 mm CPPBBO BB
PULPROG cosygpppqf
TD 2048
SOLVENT CDCl3
NS 1
DS 8
SWH 3937.008 Hz
FIDRES 3.844734 Hz
AQ 0.2600960 sec
RG 25.06
DW 127.000 usec
DE 6.50 usec
TE 298.2 K
d0 0.00000300 sec
D1 1.89309394 sec
d11 0.03000000 sec
d12 0.00002000 sec
d13 0.00000400 sec
D16 0.00020000 sec
in0 0 sec
ST1CNT 0
d0orig 0.00000300 sec
t1loop 0
SFO1 500.1321530 MHz
NUC1 1H
P0 10.60 usec
P1 10.60 usec
P17 2500.00 usec
PLW1 20.00000000 W
PLW10 3.32430005 W
GPNAM[1] SMSQ10.100
GPZ1 10.00 %
P16 1000.00 usec
F1 - Acquisition parameters
TD 128
SFO1 500.1322 MHz
FIDRES 61.515747 Hz
SW 7.872 ppm
FnMODE QF
F2 - Processing parameters
SI 1024
SF 500.1300234 MHz
WDW QSINE
SSB 0
LB 0 Hz
GB 0
PC 1.40
F1 - Processing parameters
SI 1024
MC2 QF
SF 500.1300234 MHz
WDW QSINE
SSB 0
LB 0 Hz
GB 0

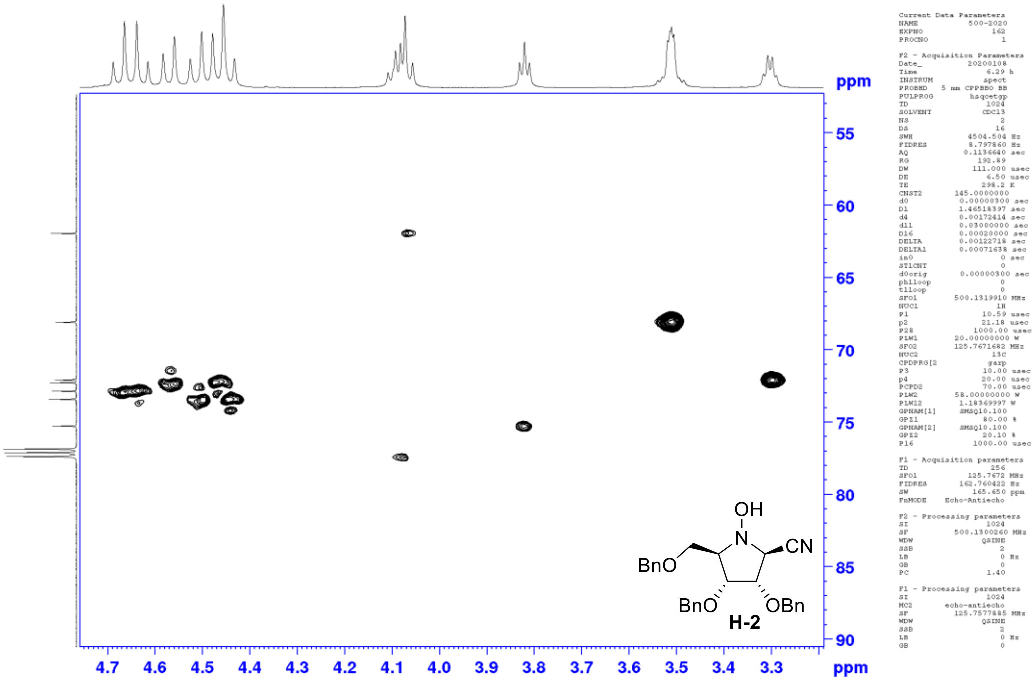
ppm
OH
N
CN
BnO
BnO
OBn
H-2
4.7 4.6 4.5 4.4 4.3 4.2 4.1 4.0 3.9 3.8 3.7 3.6 3.5 3.4 3.3
ppm
55 60 65 70 75 80 85 90

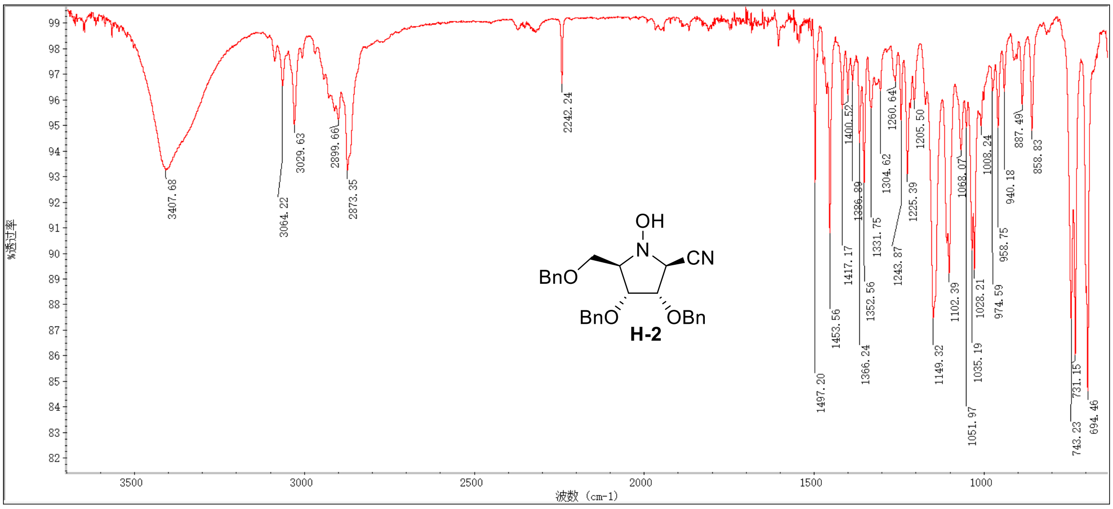
%透过率
波数（cm-1）
3407.68
3064.22
3029.63
2899.66
2873.35
2242.24
1497.20
1453.56
1417.17
1400.52
1386.89
1366.24
1352.56
1331.75
1304.62
1260.64
1243.87
1225.39
1205.50
1149.32
1102.39
1068.07
1051.97
1035.19
1028.21
1008.24
974.59
958.75
940.18
887.49
858.83
743.23
731.15
694.46
OH
N
CN
BnO
BnO
OBn
H-2

Compound **A-3**:

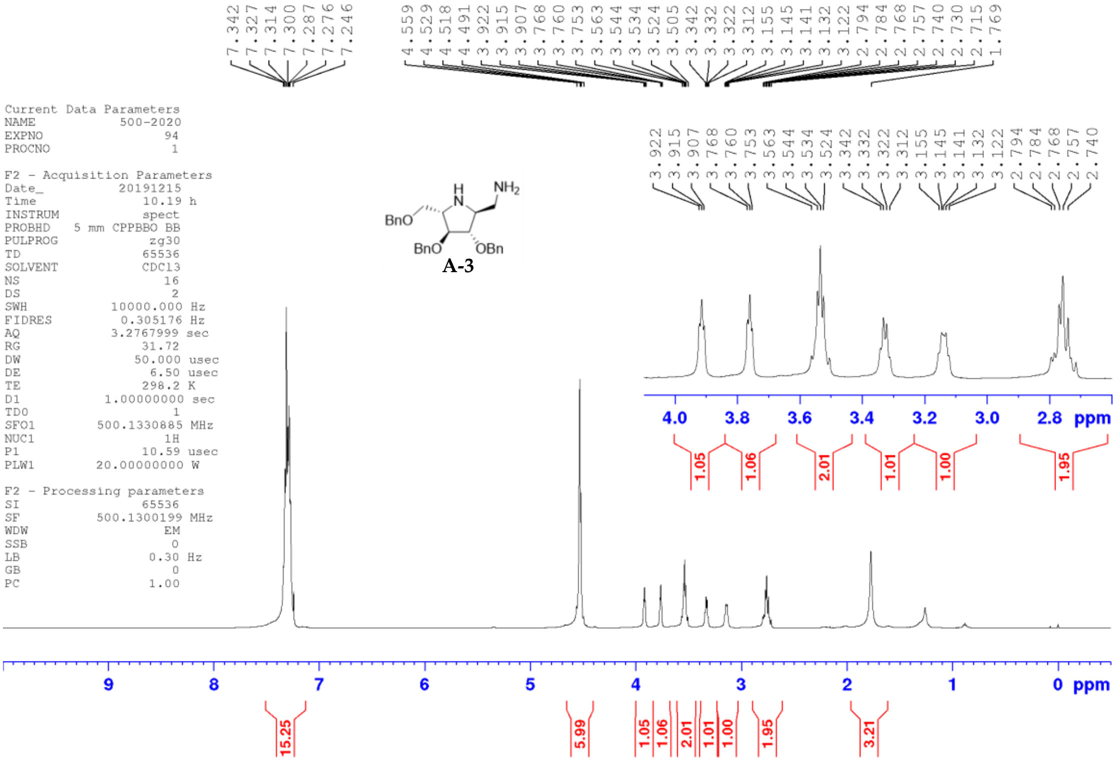
7.342 7.327 7.314 7.300 7.287 7.276 7.246
4.559 4.529 4.518 4.491 3.922 3.915 3.907 3.768 3.760 3.753 3.563 3.544 3.534 3.524 3.505 3.342 3.332 3.322 3.312 3.155 3.145 3.141 3.132 3.122 2.794 2.784 2.768 2.757 2.740 2.730 2.715 1.769
Current Data Parameters
NAME 500-2020
EXPNO 94
PROCNO 1
F2 - Acquisition Parameters
Date_ 20191215
Time 10.19 h
INSTRUM spect
PROBHD 5 mm CPPBBO BB
PULPROG zg30
TD 65536
SOLVENT CDCl3
NS 16
DS 2
SWH 10000.000 Hz
FIDRES 0.305176 Hz
AQ 3.2767999 sec
RG 31.72
DW 50.000 usec
DE 6.50 usec
TE 298.2 K
D1 1.00000000 sec
TD0 1
SFO1 500.1330885 MHz
NUC1 1H
P1 10.59 usec
PLW1 20.00000000 W
F2 - Processing parameters
SI 65536
SF 500.1300199 MHz
WDW EM
SSB 0
LB 0.30 Hz
GB 0
PC 1.00
BnO
H
N
NH2
BnO
OBn
A-3
4.0 3.8 3.6 3.4 3.2 3.0 2.8 ppm
1.05 1.06 2.01 1.01 1.00 1.95
9 8 7 6 5 4 3 2 1 0 ppm
15.25 5.99 1.05 1.06 2.01 1.01 1.00 1.95 3.21

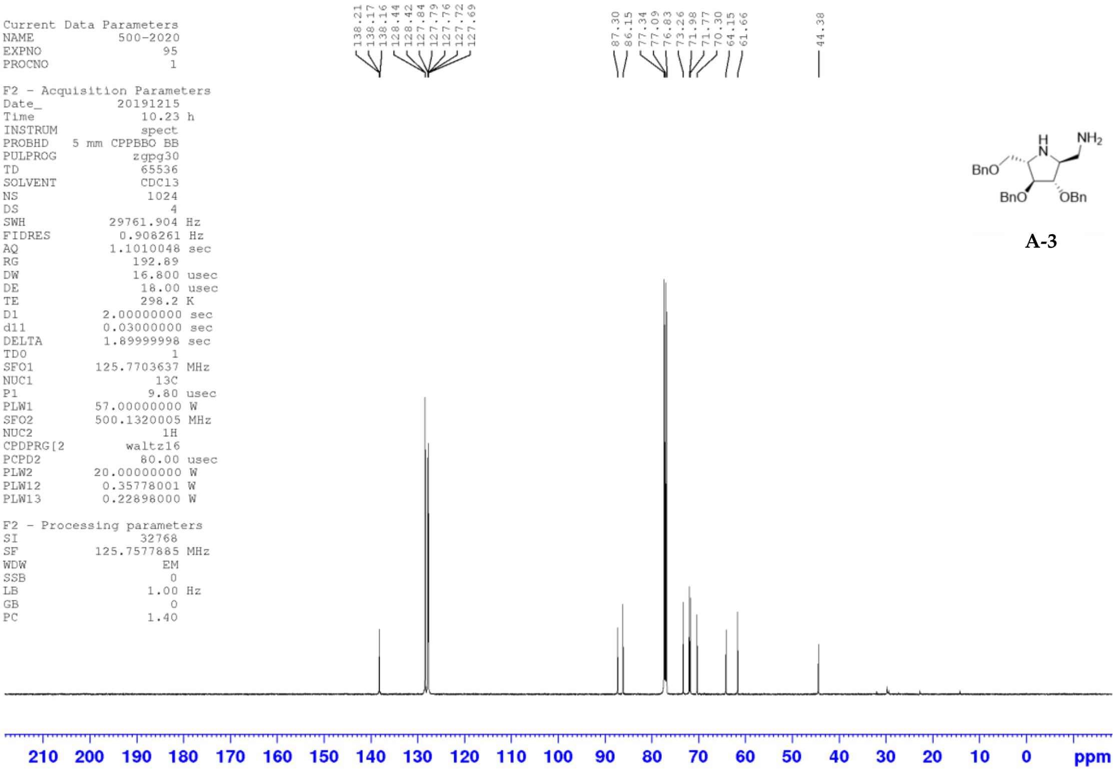
138.21 138.17 138.16 128.44 128.42 127.84 127.79 127.76 127.72 127.69
87.30 86.15 77.34 77.09 76.83 73.26 71.98 71.77 70.30 64.15 61.66
44.38
Current Data Parameters
NAME 500-2020
EXPNO 95
PROCNO 1
F2 - Acquisition Parameters
Date_ 20191215
Time 10.23 h
INSTRUM spect
PROBHD 5 mm CPPBBO BB
PULPROG zgpg30
TD 65536
SOLVENT CDCl3
NS 1024
DS 4
SWH 29761.904 Hz
FIDRES 0.908261 Hz
AQ 1.1010048 sec
RG 192.89
DW 16.800 usec
DE 18.00 usec
TE 298.2 K
D1 2.00000000 sec
d11 0.03000000 sec
DELTA 1.89999998 sec
TD0 1
SFO1 125.7703637 MHz
NUC1 13C
P1 9.80 usec
PLW1 57.00000000 W
SFO2 500.1320005 MHz
NUC2 1H
CPDPRG[2 waltz16
PCPD2 80.00 usec
PLW2 20.00000000 W
PLW12 0.35778001 W
PLW13 0.22898000 W
F2 - Processing parameters
SI 32768
SF 125.7577885 MHz
WDW EM
SSB 0
LB 1.00 Hz
GB 0
PC 1.40
BnO
H
N
NH2
BnO
OBn
A-3
210 200 190 180 170 160 150 140 130 120 110 100 90 80 70 60 50 40 30 20 10 0 ppm

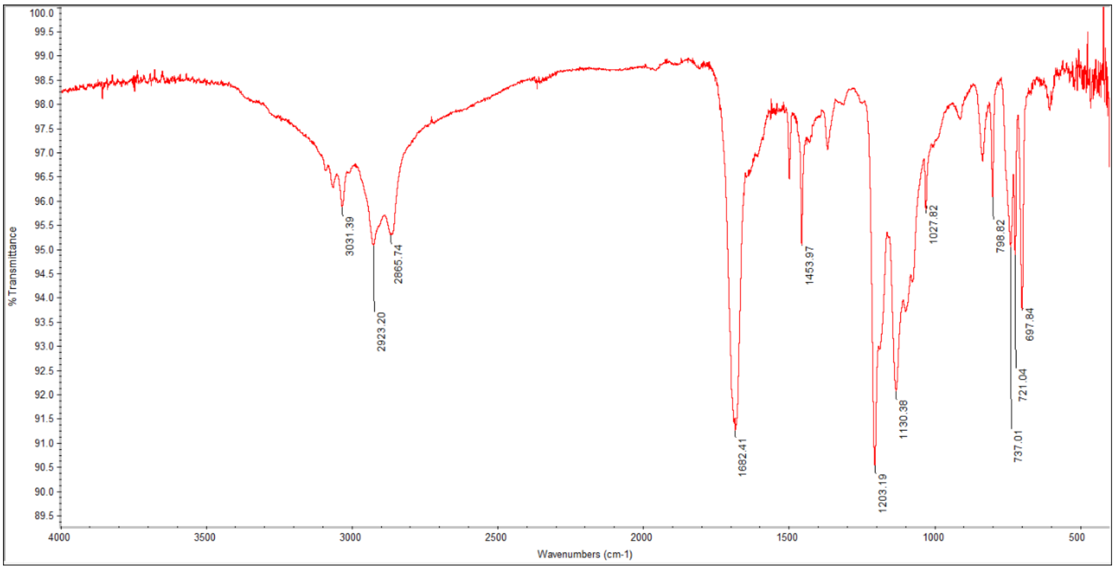
% Transmittance
Wavenumbers (cm-1)
3031.39
2923.20
2865.74
1682.41
1453.97
1203.19
1130.38
1027.82
798.82
737.01
721.04
697.84

Compound **B-3**:

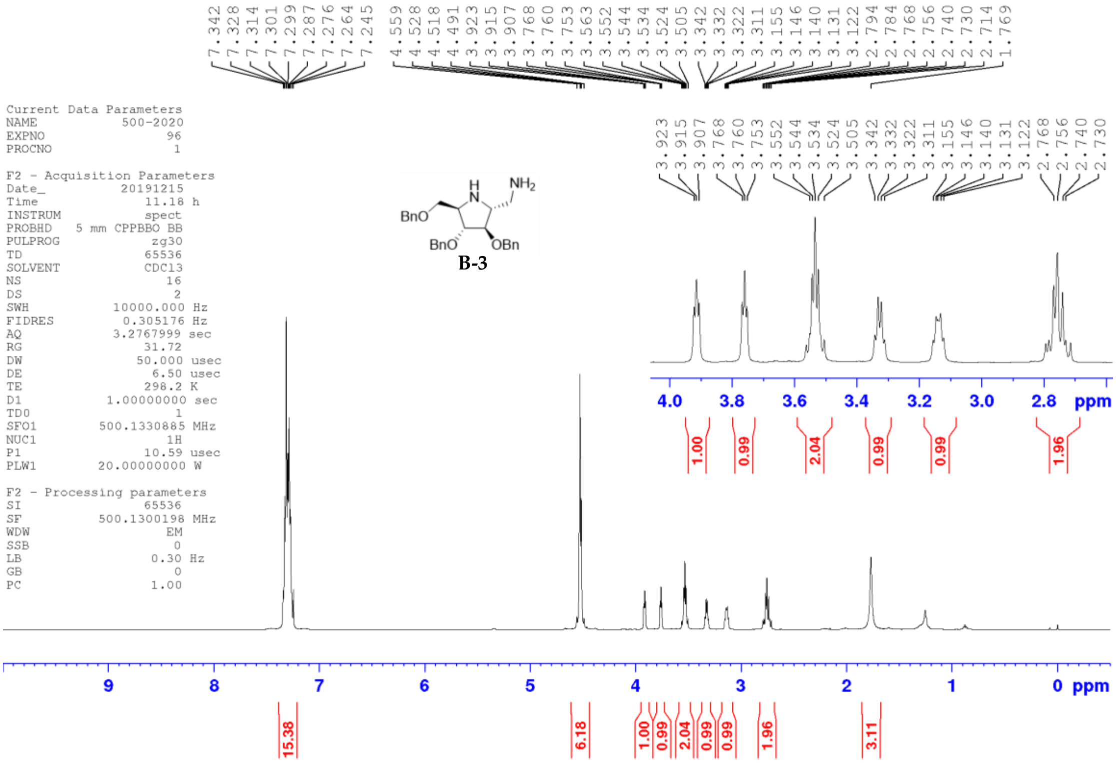
7.342 7.328 7.314 7.301 7.299 7.287 7.276 7.264 7.245
4.559 4.528 4.518 4.491 3.923 3.915 3.907 3.768 3.760 3.753 3.563 3.552 3.544 3.534 3.524 3.505 3.342 3.332 3.322 3.311 3.155 3.146 3.140 3.131 3.122 2.794 2.784 2.768 2.756 2.740 2.730 2.714 1.769
Current Data Parameters
NAME 500-2020
EXPNO 96
PROCNO 1
F2 - Acquisition Parameters
Date_ 20191215
Time 11.18 h
INSTRUM spect
PROBHD 5 mm CPPBBO BB
PULPROG zg30
TD 65536
SOLVENT CDCl3
NS 16
DS 2
SWH 10000.000 Hz
FIDRES 0.305176 Hz
AQ 3.2767999 sec
RG 31.72
DW 50.000 usec
DE 6.50 usec
TE 298.2 K
D1 1.00000000 sec
TD0 1
SFO1 500.1330885 MHz
NUC1 1H
P1 10.59 usec
PLW1 20.00000000 W
F2 - Processing parameters
SI 65536
SF 500.1300198 MHz
WDW EM
SSB 0
LB 0.30 Hz
GB 0
PC 1.00
BnO
H
N
NH2
BnO
OBn
B-3
ppm
15.38
6.18
1.00
0.99
2.04
0.99
0.99
1.96
3.11

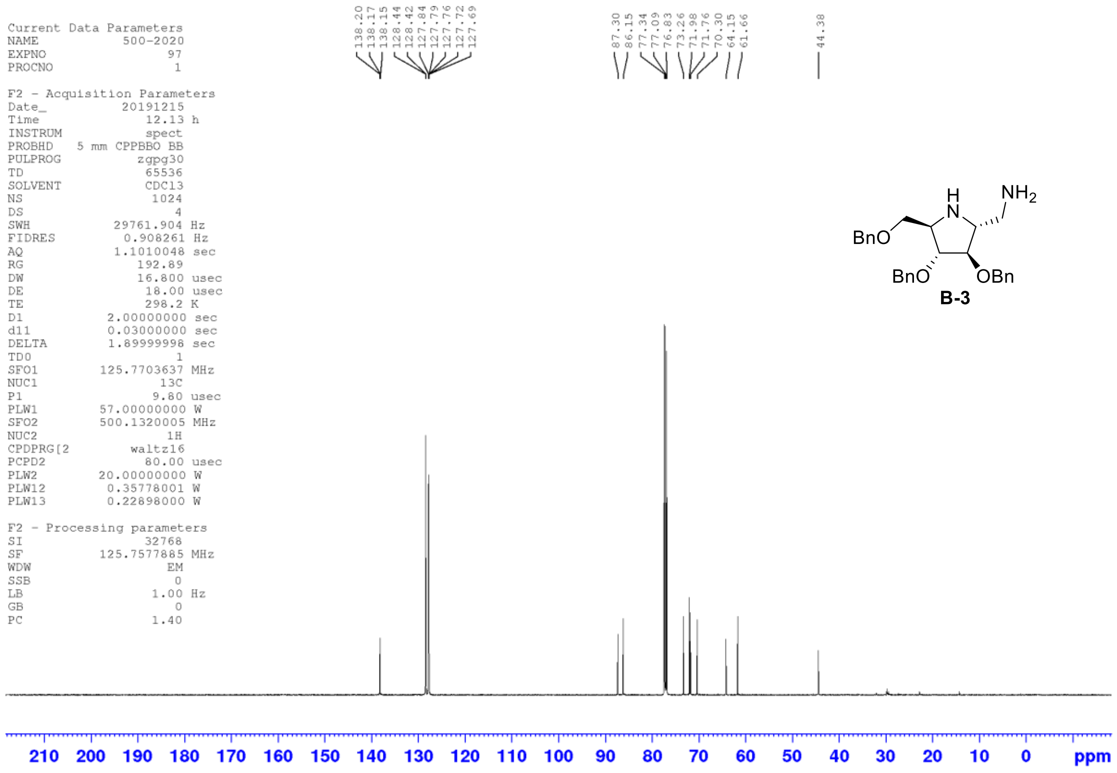
Current Data Parameters
NAME 500-2020
EXPNO 97
PROCNO 1
F2 - Acquisition Parameters
Date_ 20191215
Time 12.13 h
INSTRUM spect
PROBHD 5 mm CPPBBO BB
PULPROG zgpg30
TD 65536
SOLVENT CDCl3
NS 1024
DS 4
SWH 29761.904 Hz
FIDRES 0.908261 Hz
AQ 1.1010048 sec
RG 192.89
DW 16.800 usec
DE 18.00 usec
TE 298.2 K
D1 2.00000000 sec
d11 0.03000000 sec
DELTA 1.89999998 sec
TD0 1
SFO1 125.7703637 MHz
NUC1 13C
P1 9.80 usec
PLW1 57.00000000 W
SFO2 500.1320005 MHz
NUC2 1H
CPDPRG[2 waltz16
PCPD2 80.00 usec
PLW2 20.00000000 W
PLW12 0.35778001 W
PLW13 0.22898000 W
F2 - Processing parameters
SI 32768
SF 125.7577885 MHz
WDW EM
SSB 0
LB 1.00 Hz
GB 0
PC 1.40
138.20
138.17
138.15
128.44
128.42
127.84
127.79
127.76
127.72
127.69
87.30
86.15
77.34
77.09
76.83
73.26
71.98
71.76
70.30
64.15
61.66
44.38
BnO
H
N
NH2
BnO
OBn
B-3
210 200 190 180 170 160 150 140 130 120 110 100 90 80 70 60 50 40 30 20 10 0 ppm

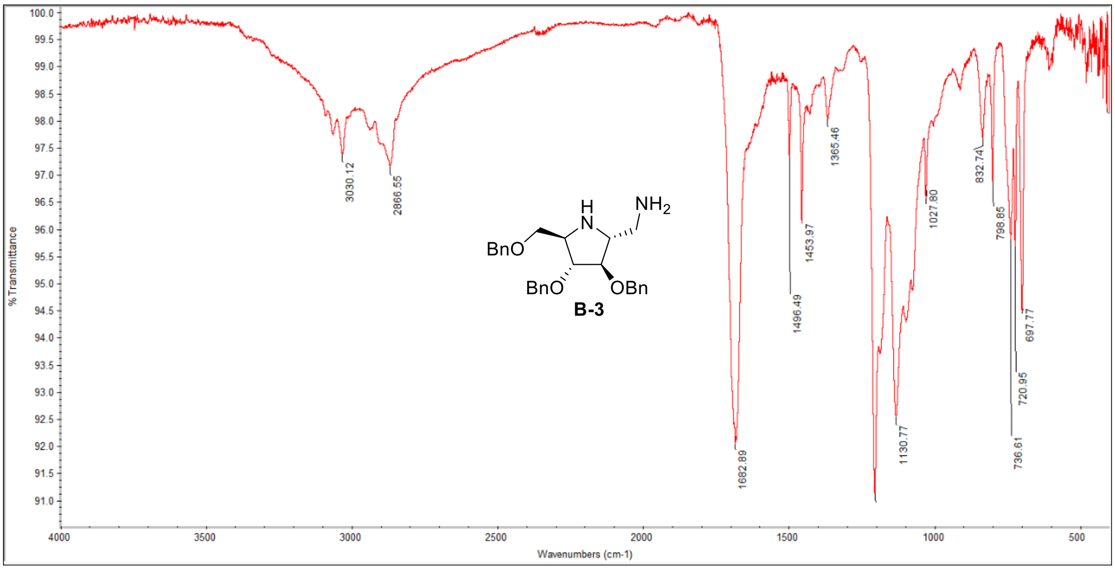
% Transmittance
Wavenumbers (cm-1)
3030.12
2866.55
1682.89
1496.49
1453.97
1365.46
1130.77
1027.80
832.74
798.85
736.61
720.95
697.77
BnO
H
N
NH2
BnO
OBn
B-3

Compound **C-3**:

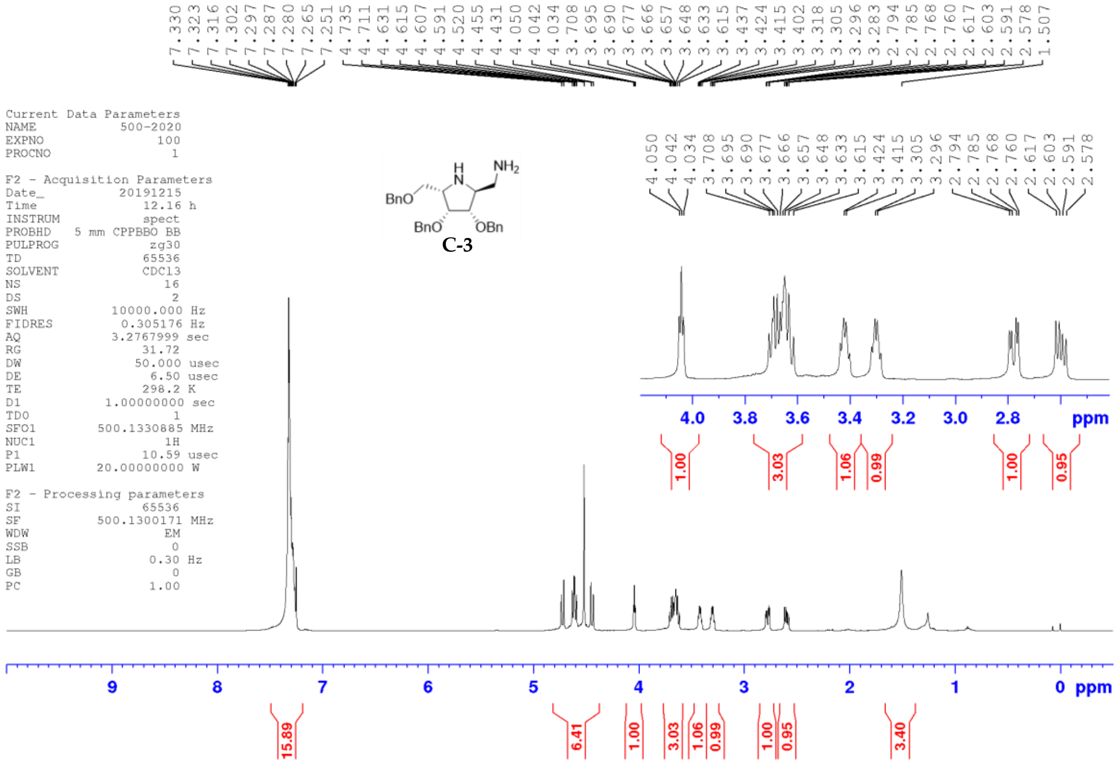
7.330 7.323 7.316 7.302 7.297 7.287 7.280 7.265 7.251 4.735 4.711 4.631 4.615 4.607 4.591 4.520 4.455 4.431 4.050 4.042 4.034 3.708 3.695 3.690 3.677 3.666 3.657 3.648 3.633 3.615 3.437 3.424 3.415 3.402 3.318 3.305 3.296 3.283 2.794 2.785 2.768 2.760 2.617 2.603 2.591 2.578 1.507
Current Data Parameters
NAME 500-2020
EXPNO 100
PROCNO 1
F2 - Acquisition Parameters
Date_ 20191215
Time 12.16 h
INSTRUM spect
PROBHD 5 mm CPPBBO BB
PULPROG zg30
TD 65536
SOLVENT CDCl3
NS 16
DS 2
SWH 10000.000 Hz
FIDRES 0.305176 Hz
AQ 3.2767999 sec
RG 31.72
DW 50.000 usec
DE 6.50 usec
TE 298.2 K
D1 1.00000000 sec
TD0 1
SFO1 500.1330885 MHz
NUC1 1H
P1 10.59 usec
PLW1 20.00000000 W
F2 - Processing parameters
SI 65536
SF 500.1300171 MHz
WDW EM
SSB 0
LB 0.30 Hz
GB 0
PC 1.00
BnO
H
N
NH2
BnO
OBn
C-3
4.050 4.042 4.034 3.708 3.695 3.690 3.677 3.666 3.657 3.648 3.633 3.615 3.424 3.415 3.305 3.296 2.794 2.785 2.768 2.760 2.617 2.603 2.591 2.578
4.0 3.8 3.6 3.4 3.2 3.0 2.8 ppm
1.00 3.03 1.06 0.99 1.00 0.95
9 8 7 6 5 4 3 2 1 0 ppm
15.89 6.41 1.00 3.03 1.06 0.99 1.00 0.95 3.40

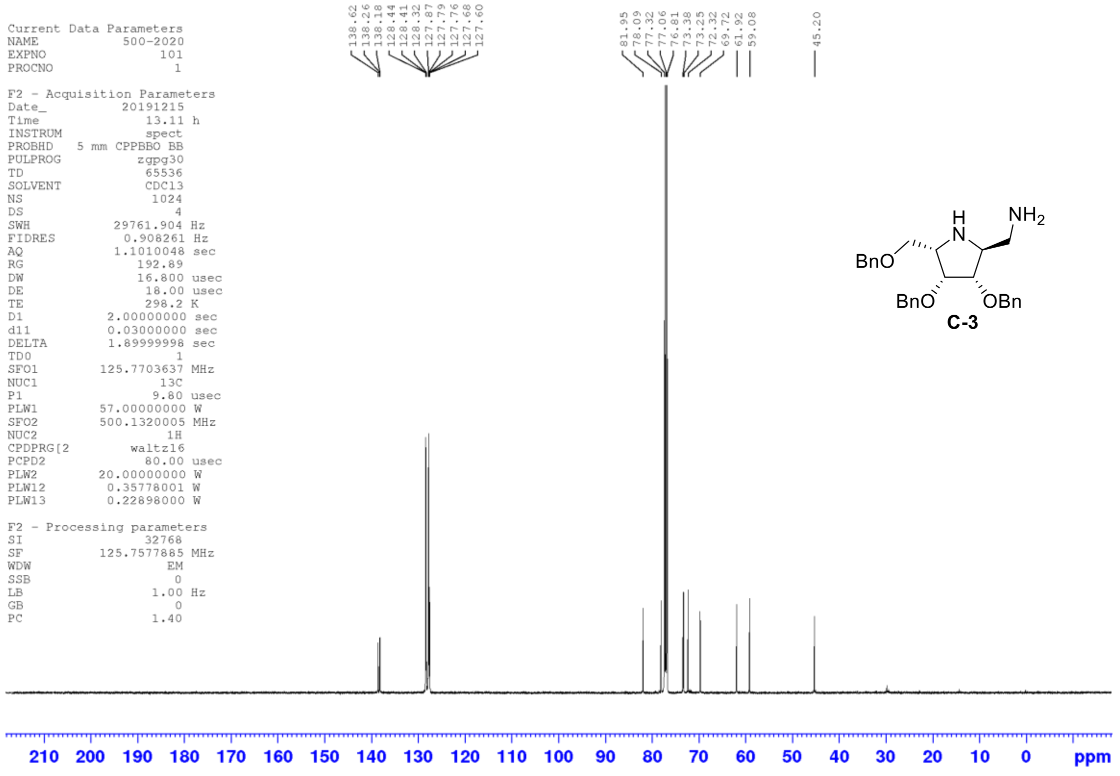
138.62 138.26 138.18 128.44 128.41 128.32 127.87 127.79 127.76 127.68 127.60 81.95 78.09 77.32 77.06 76.81 73.38 73.25 72.32 69.72 61.92 59.08 45.20
Current Data Parameters
NAME 500-2020
EXPNO 101
PROCNO 1
F2 - Acquisition Parameters
Date_ 20191215
Time 13.11 h
INSTRUM spect
PROBHD 5 mm CPPBBO BB
PULPROG zgpg30
TD 65536
SOLVENT CDCl3
NS 1024
DS 4
SWH 29761.904 Hz
FIDRES 0.908261 Hz
AQ 1.1010048 sec
RG 192.89
DW 16.800 usec
DE 18.00 usec
TE 298.2 K
D1 2.00000000 sec
d11 0.03000000 sec
DELTA 1.89999998 sec
TD0 1
SFO1 125.7703637 MHz
NUC1 13C
P1 9.80 usec
PLW1 57.00000000 W
SFO2 500.1320005 MHz
NUC2 1H
CPDPRG[2 waltz16
PCPD2 80.00 usec
PLW2 20.00000000 W
PLW12 0.35778001 W
PLW13 0.22898000 W
F2 - Processing parameters
SI 32768
SF 125.7577885 MHz
WDW EM
SSB 0
LB 1.00 Hz
GB 0
PC 1.40
H
N
NH2
BnO
BnO
OBn
C-3
210 200 190 180 170 160 150 140 130 120 110 100 90 80 70 60 50 40 30 20 10 0 ppm

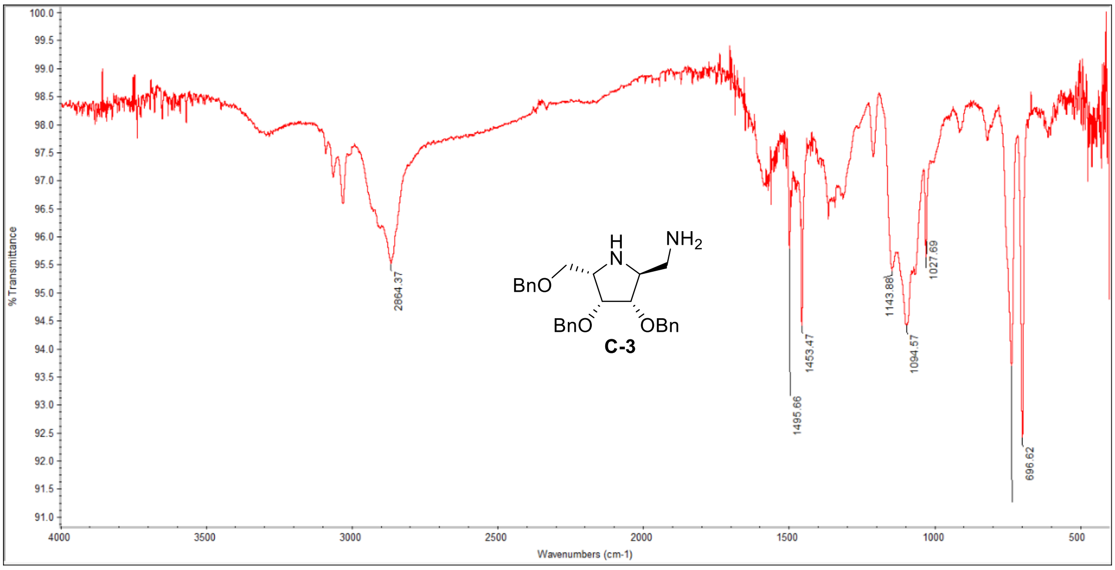

% Transmittance
Wavenumbers (cm-1)
2864.37
1495.66
1453.47
1143.88
1094.57
1027.69
696.62
BnO
BnO
OBn
H
N
NH2
C-3

Compound **D-3**:

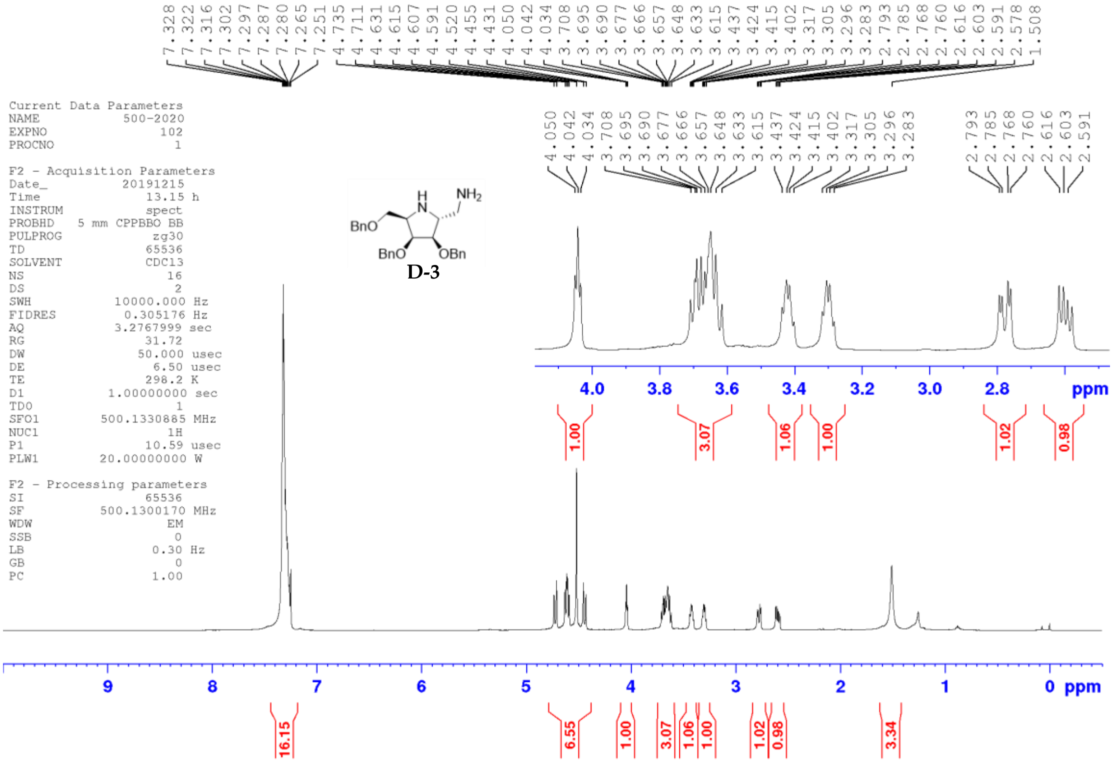

7.328 7.322 7.316 7.302 7.297 7.287 7.280 7.265 7.251 4.735 4.711 4.631 4.615 4.607 4.591 4.520 4.455 4.431 4.050 4.042 4.034 3.708 3.695 3.690 3.677 3.666 3.657 3.648 3.633 3.615 3.437 3.424 3.415 3.402 3.317 3.305 3.296 3.283 2.793 2.785 2.768 2.760 2.616 2.603 2.591 2.578 1.508
Current Data Parameters
NAME 500-2020
EXPNO 102
PROCNO 1
F2 - Acquisition Parameters
Date_ 20191215
Time 13.15 h
INSTRUM spect
PROBHD 5 mm CPPBBO BB
PULPROG zg30
TD 65536
SOLVENT CDCl3
NS 16
DS 2
SWH 10000.000 Hz
FIDRES 0.305176 Hz
AQ 3.2767999 sec
RG 31.72
DW 50.000 usec
DE 6.50 usec
TE 298.2 K
D1 1.00000000 sec
TD0 1
SFO1 500.1330885 MHz
NUC1 1H
P1 10.59 usec
PLW1 20.00000000 W
F2 - Processing parameters
SI 65536
SF 500.1300170 MHz
WDW EM
SSB 0
LB 0.30 Hz
GB 0
PC 1.00
BnO
BnO
OBn
H
N
NH2
D-3
4.050 4.042 4.034 3.708 3.695 3.690 3.677 3.666 3.657 3.648 3.633 3.615 3.437 3.424 3.415 3.402 3.317 3.305 3.296 3.283 2.793 2.785 2.768 2.760 2.616 2.603 2.591
4.0 3.8 3.6 3.4 3.2 3.0 2.8 ppm
1.00 3.07 1.06 1.00 1.02 0.98
9 8 7 6 5 4 3 2 1 0 ppm
16.15 6.55 1.00 3.07 1.06 1.00 1.02 0.98 3.34

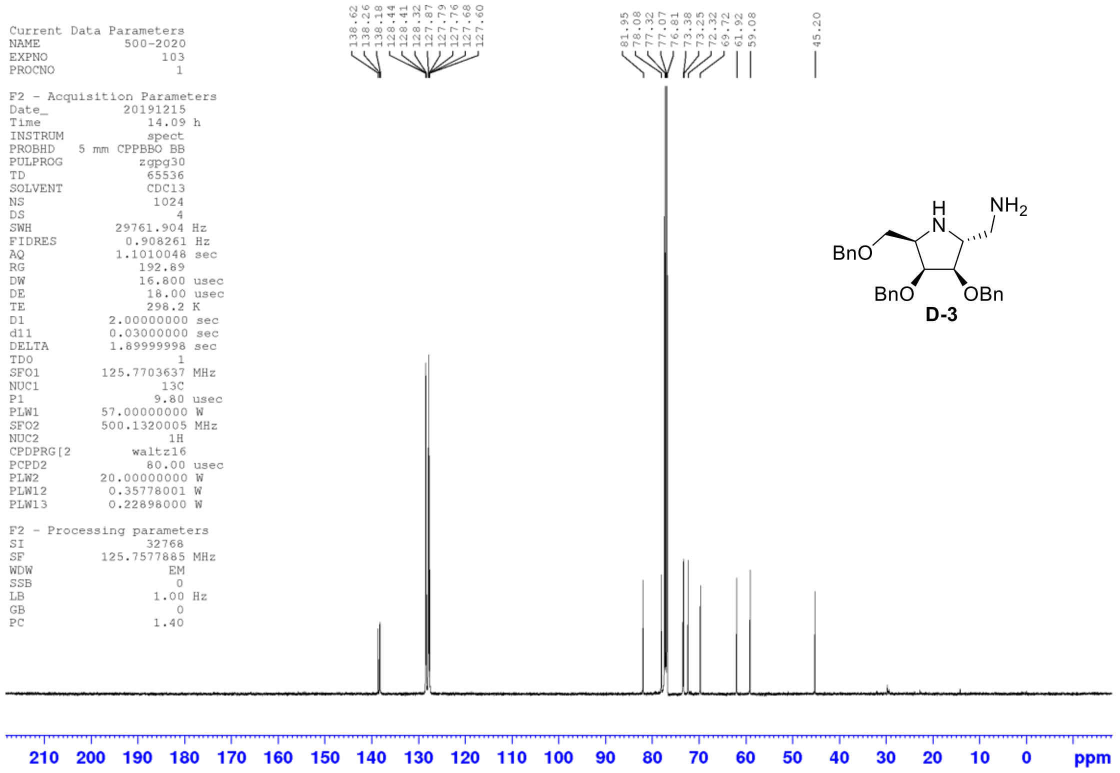
Current Data Parameters
NAME 500-2020
EXPNO 103
PROCNO 1
F2 - Acquisition Parameters
Date_ 20191215
Time 14.09 h
INSTRUM spect
PROBHD 5 mm CPPBBO BB
PULPROG zgpg30
TD 65536
SOLVENT CDCl3
NS 1024
DS 4
SWH 29761.904 Hz
FIDRES 0.908261 Hz
AQ 1.1010048 sec
RG 192.89
DW 16.800 usec
DE 18.00 usec
TE 298.2 K
D1 2.00000000 sec
d11 0.03000000 sec
DELTA 1.89999998 sec
TD0 1
SFO1 125.7703637 MHz
NUC1 13C
P1 9.80 usec
PLW1 57.00000000 W
SFO2 500.1320005 MHz
NUC2 1H
CPDPRG[2 waltz16
PCPD2 80.00 usec
PLW2 20.00000000 W
PLW12 0.35778001 W
PLW13 0.22898000 W
F2 - Processing parameters
SI 32768
SF 125.7577885 MHz
WDW EM
SSB 0
LB 1.00 Hz
GB 0
PC 1.40
138.62
138.26
138.18
128.44
128.41
128.32
127.87
127.79
127.76
127.68
127.60
81.95
78.08
77.32
77.07
76.81
73.38
73.25
72.32
69.72
61.92
59.08
45.20
H
N
NH2
BnO
BnO
OBn
D-3
210 200 190 180 170 160 150 140 130 120 110 100 90 80 70 60 50 40 30 20 10 0 ppm

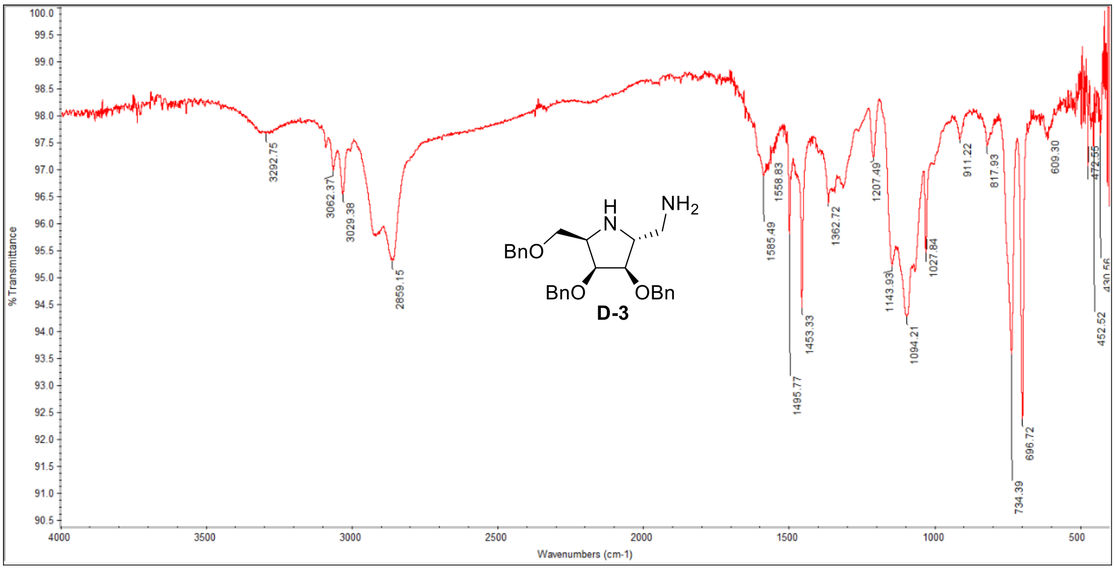
% Transmittance
100.0
99.5
99.0
98.5
98.0
97.5
97.0
96.5
96.0
95.5
95.0
94.5
94.0
93.5
93.0
92.5
92.0
91.5
91.0
90.5
3292.75
3062.37
3029.38
2859.15
1585.49
1558.83
1495.77
1453.33
1362.72
1207.49
1143.93
1094.21
1027.84
911.22
817.93
734.39
696.72
609.30
472.55
452.52
H
N
NH2
BnO
BnO
OBn
D-3
4000 3500 3000 2500 2000 1500 1000 500
Wavenumbers (cm-1)

Compound **E-3a**:

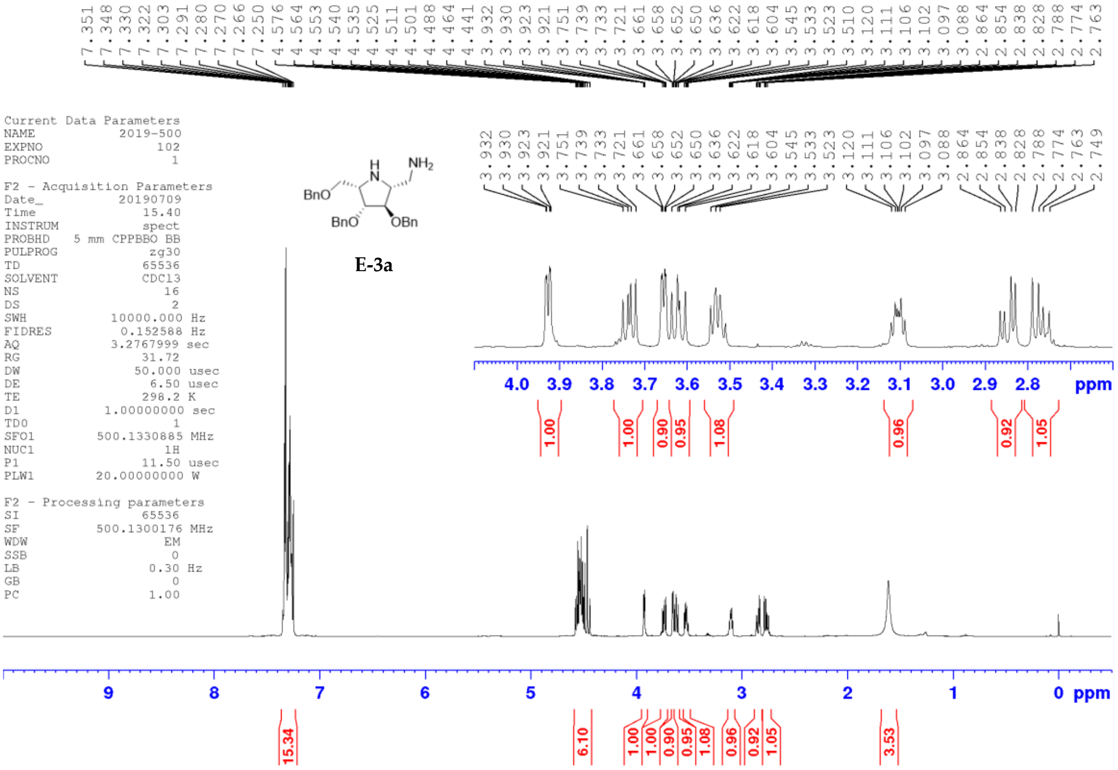

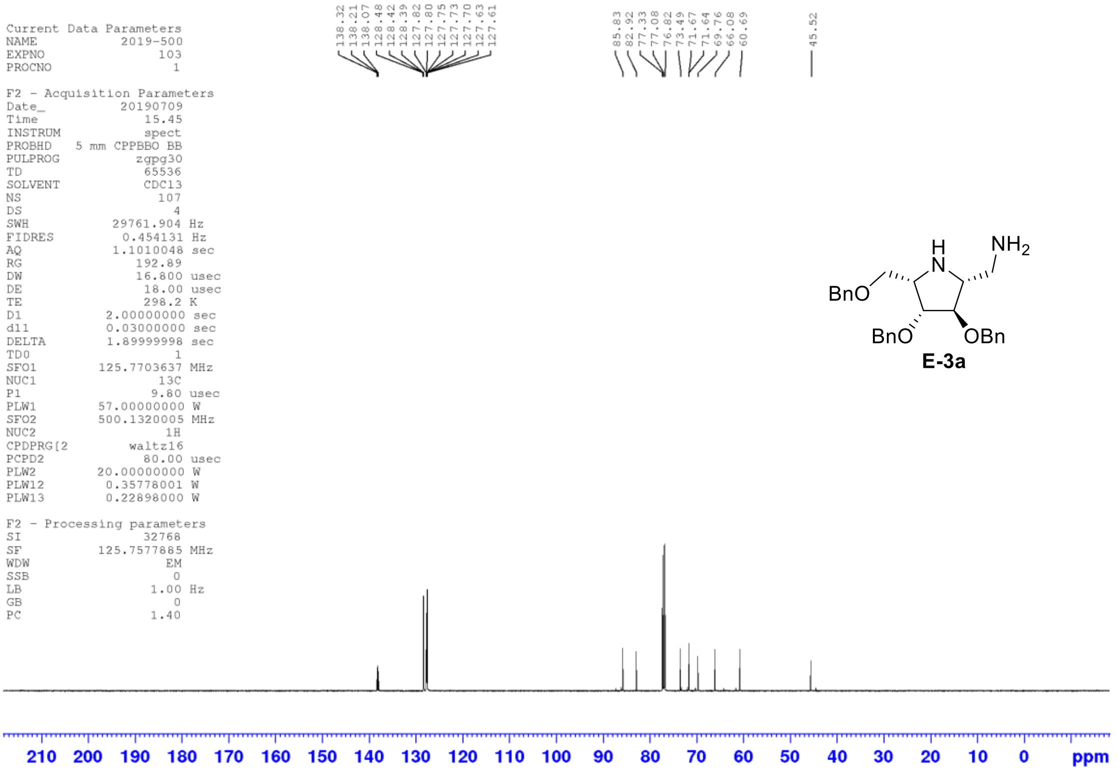

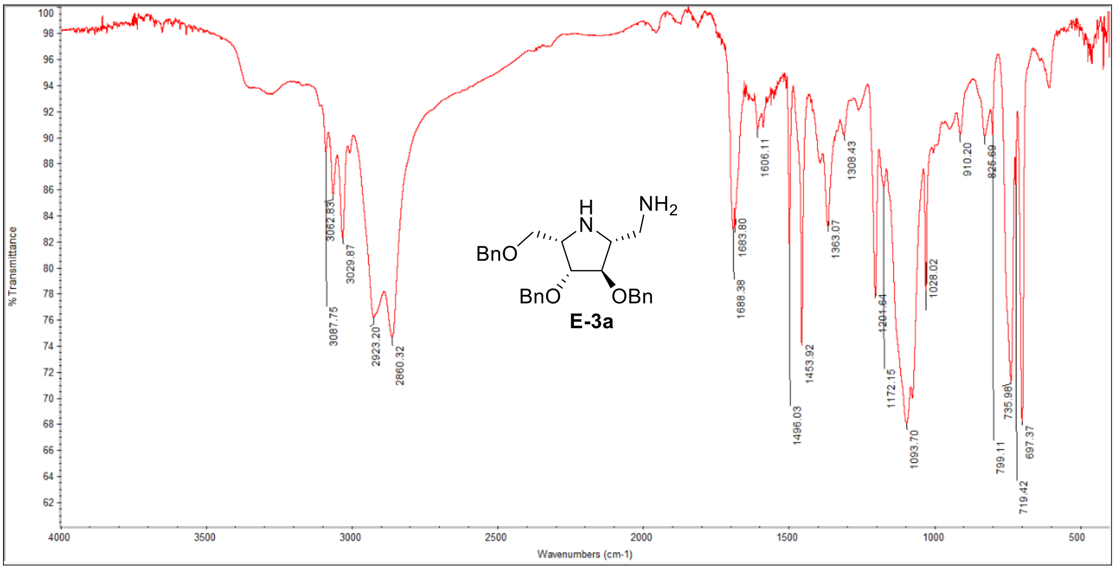

Compound **E-3b**:

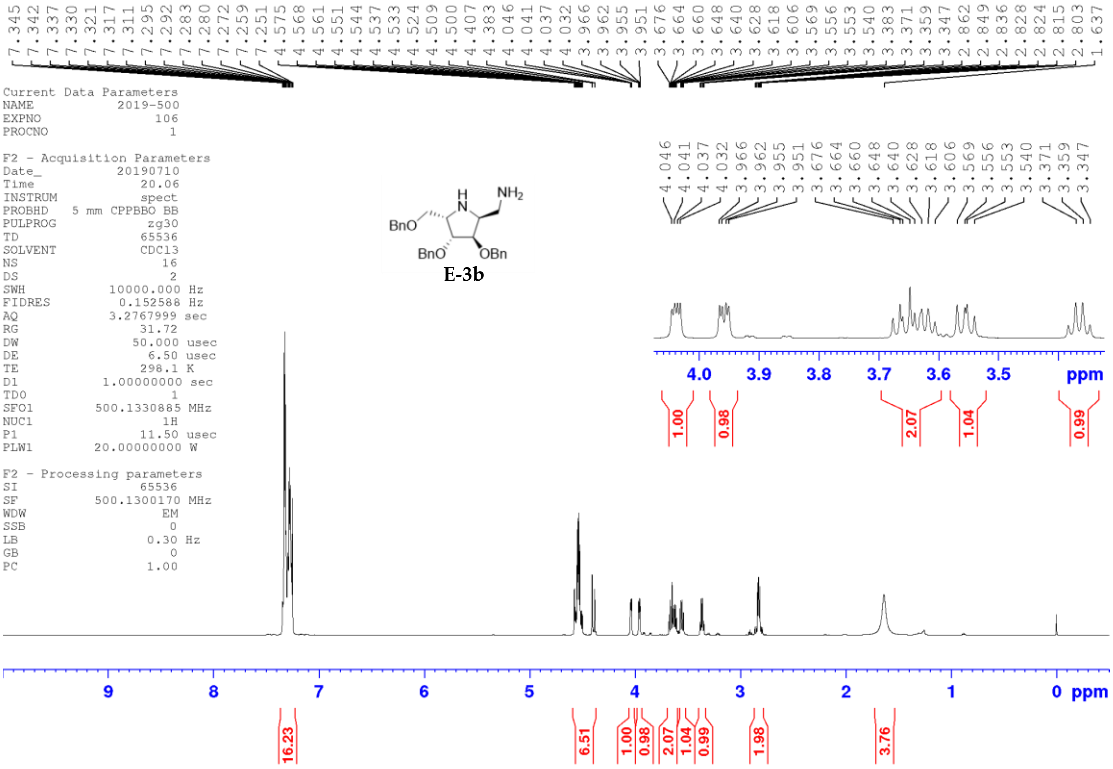

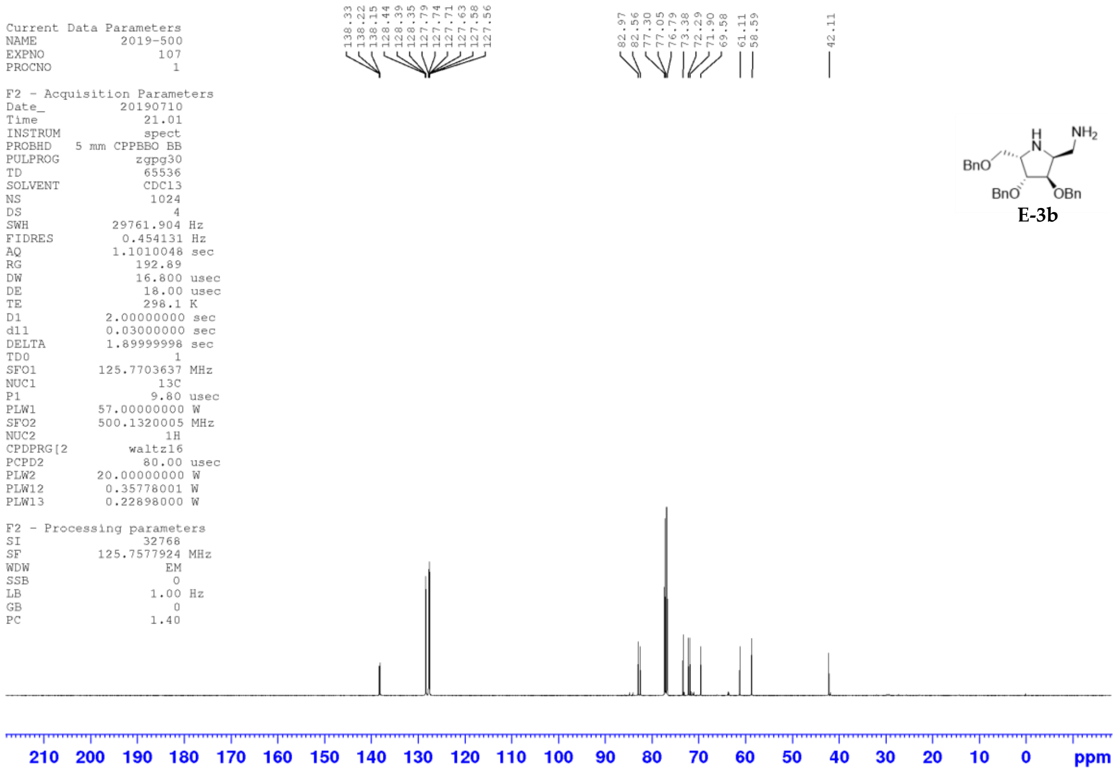

Current Data Parameters
NAME 2019-500
EXPNO 107
PROCNO 1
F2 - Acquisition Parameters
Date_ 20190710
Time 21.01
INSTRUM spect
PROBHD 5 mm CPPBBO BB
PULPROG zgpg30
TD 65536
SOLVENT CDCl3
NS 1024
DS 4
SWH 29761.904 Hz
FIDRES 0.454131 Hz
AQ 1.1010048 sec
RG 192.89
DW 16.800 usec
DE 18.00 usec
TE 298.1 K
D1 2.00000000 sec
d11 0.03000000 sec
DELTA 1.89999998 sec
TD0 1
SFO1 125.7703637 MHz
NUC1 13C
P1 9.80 usec
PLW1 57.00000000 W
SFO2 500.1320005 MHz
NUC2 1H
CPDPRG[2 waltz16
PCPD2 80.00 usec
PLW2 20.00000000 W
PLW12 0.35778001 W
PLW13 0.22898000 W
F2 - Processing parameters
SI 32768
SF 125.7577924 MHz
WDW EM
SSB 0
LB 1.00 Hz
GB 0
PC 1.40
138.33
138.22
138.15
128.44
128.39
128.35
127.79
127.74
127.71
127.63
127.58
127.56
82.97
82.56
77.30
77.05
76.79
73.38
72.29
71.90
69.58
61.11
58.59
42.11
E-3b
210 200 190 180 170 160 150 140 130 120 110 100 90 80 70 60 50 40 30 20 10 0 ppm

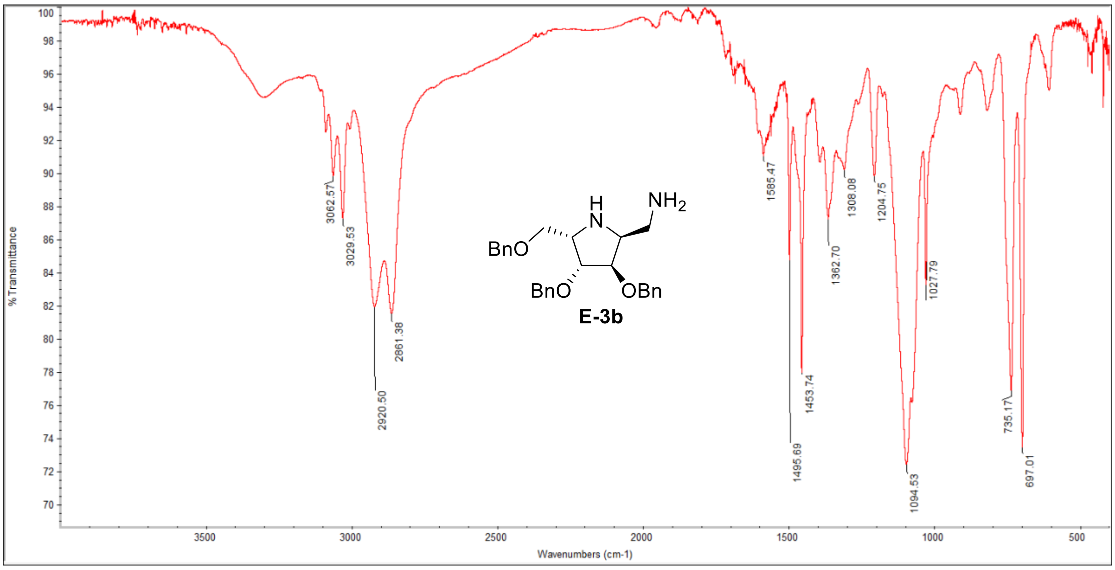

% Transmittance
Wavenumbers (cm-1)
3062.57
3029.53
2920.50
2861.38
1585.47
1495.69
1453.74
1362.70
1308.08
1204.75
1094.53
1027.79
735.17
697.01
E-3b

Compound **F-3a**:

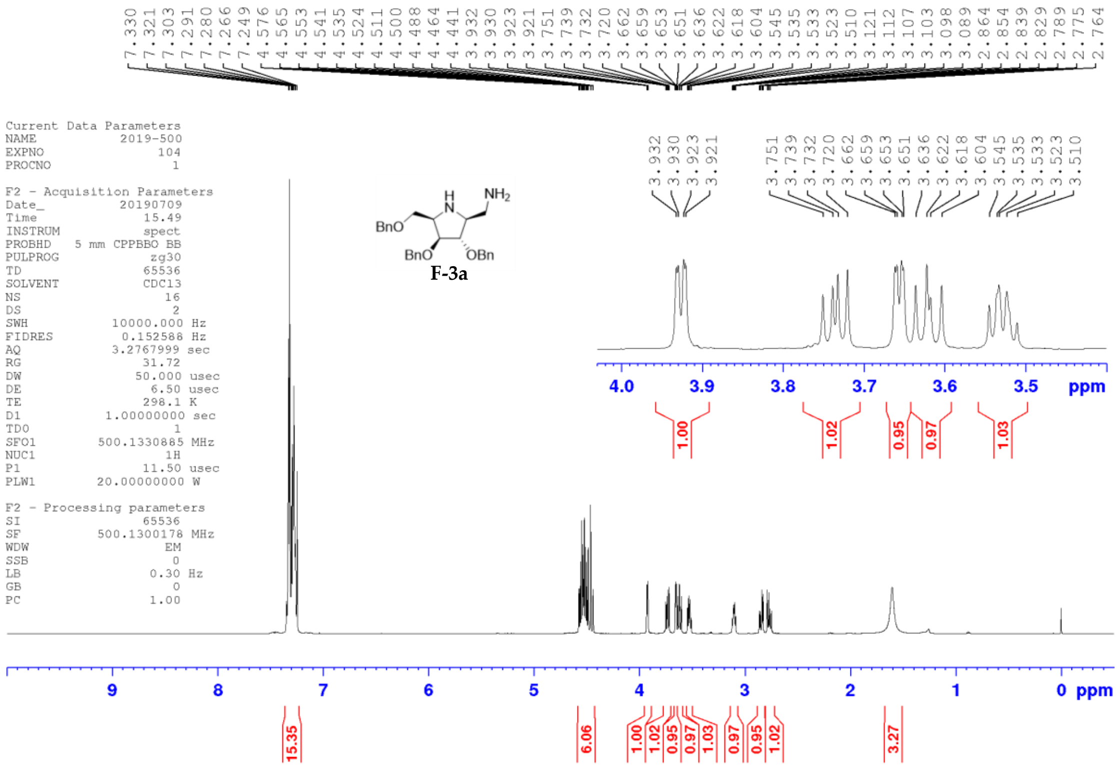

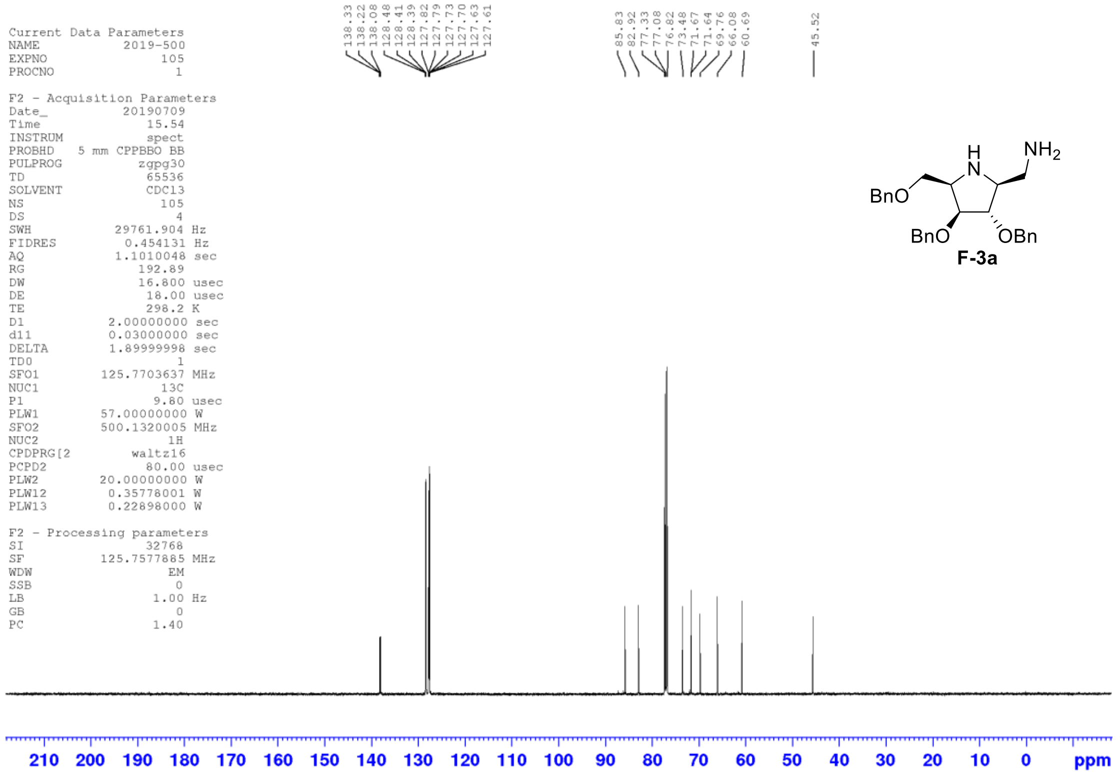

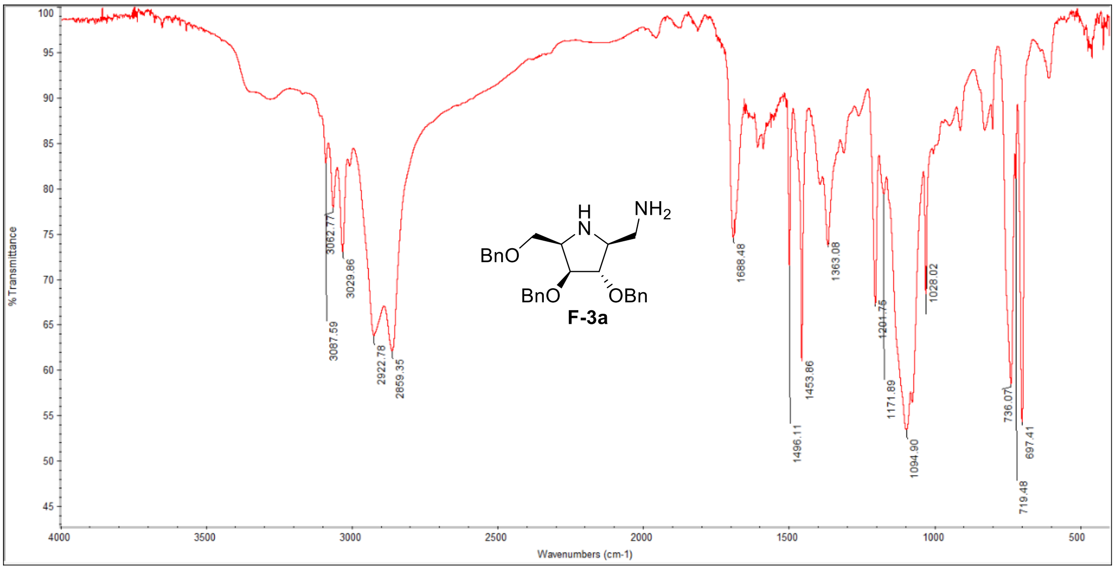

Compound **F-3b**:

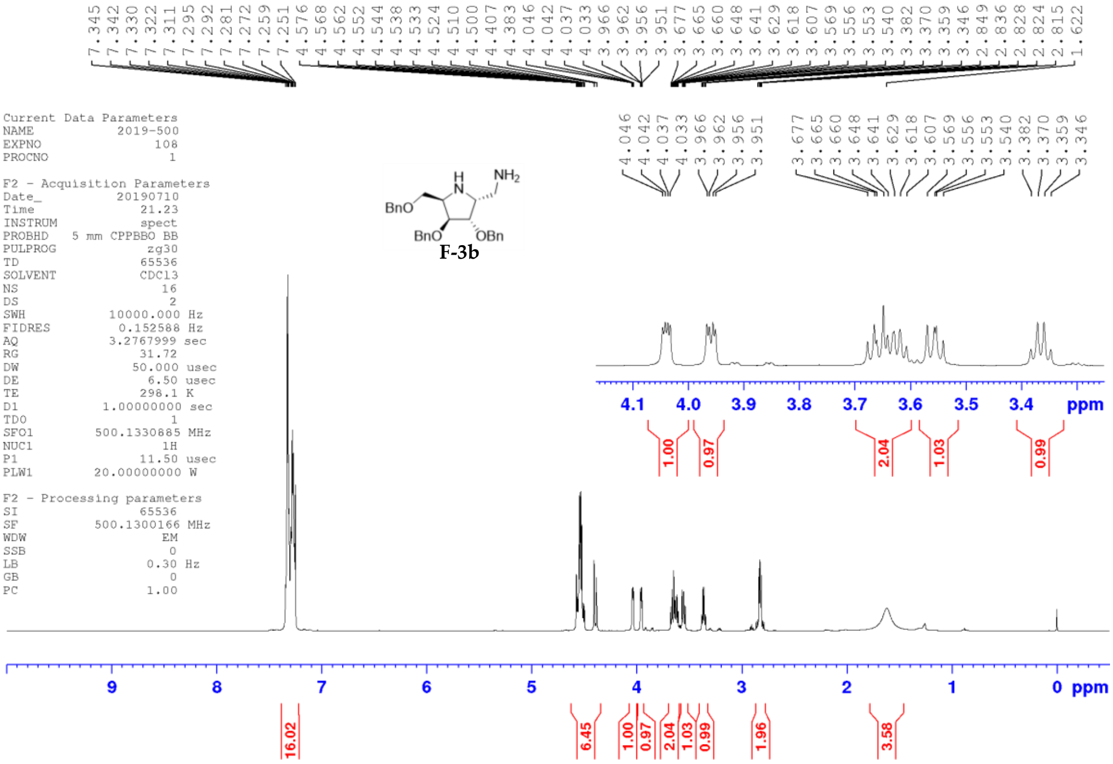

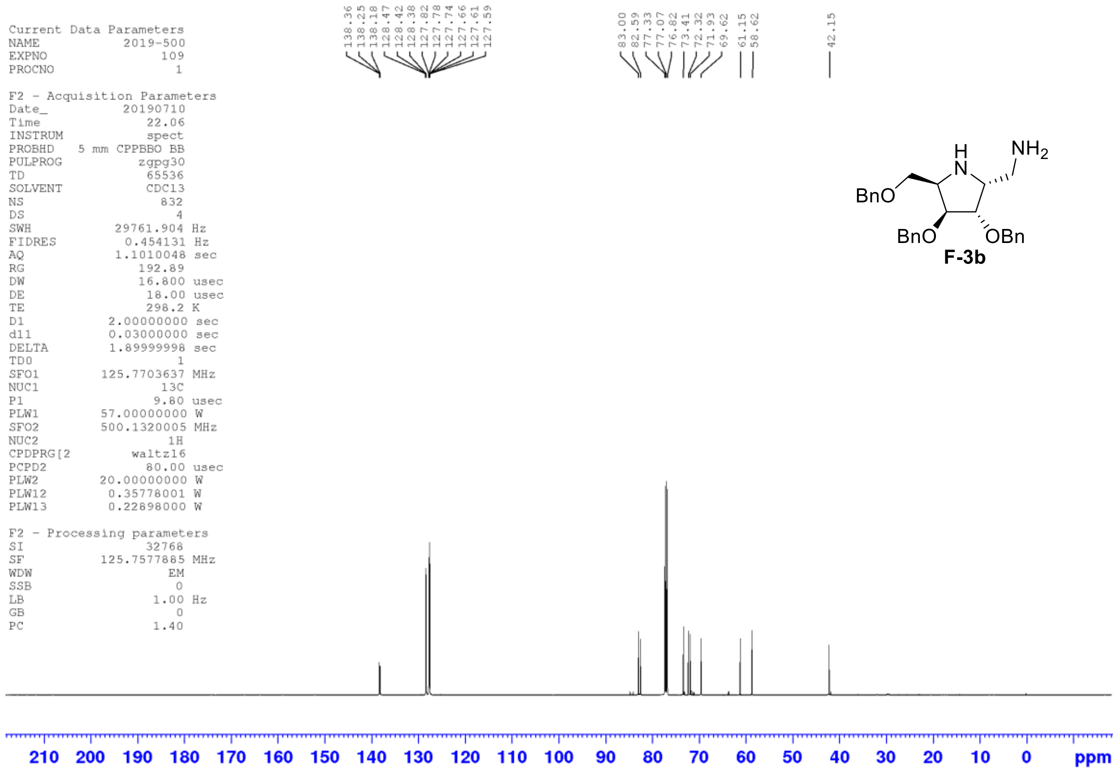

Current Data Parameters
NAME 2019-500
EXPNO 109
PROCNO 1
F2 - Acquisition Parameters
Date_ 20190710
Time 22.06
INSTRUM spect
PROBHD 5 mm CPPBBO BB
PULPROG zgpg30
TD 65536
SOLVENT CDCl3
NS 832
DS 4
SWH 29761.904 Hz
FIDRES 0.454131 Hz
AQ 1.1010048 sec
RG 192.89
DW 16.800 usec
DE 18.00 usec
TE 298.2 K
D1 2.00000000 sec
d11 0.03000000 sec
DELTA 1.89999998 sec
TD0 1
SFO1 125.7703637 MHz
NUC1 13C
P1 9.80 usec
PLW1 57.00000000 W
SFO2 500.1320005 MHz
NUC2 1H
CPDPRG[2 waltz16
PCPD2 80.00 usec
PLW2 20.00000000 W
PLW12 0.35778001 W
PLW13 0.22898000 W
F2 - Processing parameters
SI 32768
SF 125.7577865 MHz
WDW EM
SSB 0
LB 1.00 Hz
GB 0
PC 1.40
138.36
138.25
138.18
128.47
128.42
128.38
127.82
127.78
127.74
127.66
127.61
127.59
83.00
82.59
77.33
77.07
76.82
73.41
72.32
71.93
69.62
61.15
58.62
42.15
H
N
NH2
BnO
BnO
OBn
F-3b
210 200 190 180 170 160 150 140 130 120 110 100 90 80 70 60 50 40 30 20 10 0 ppm

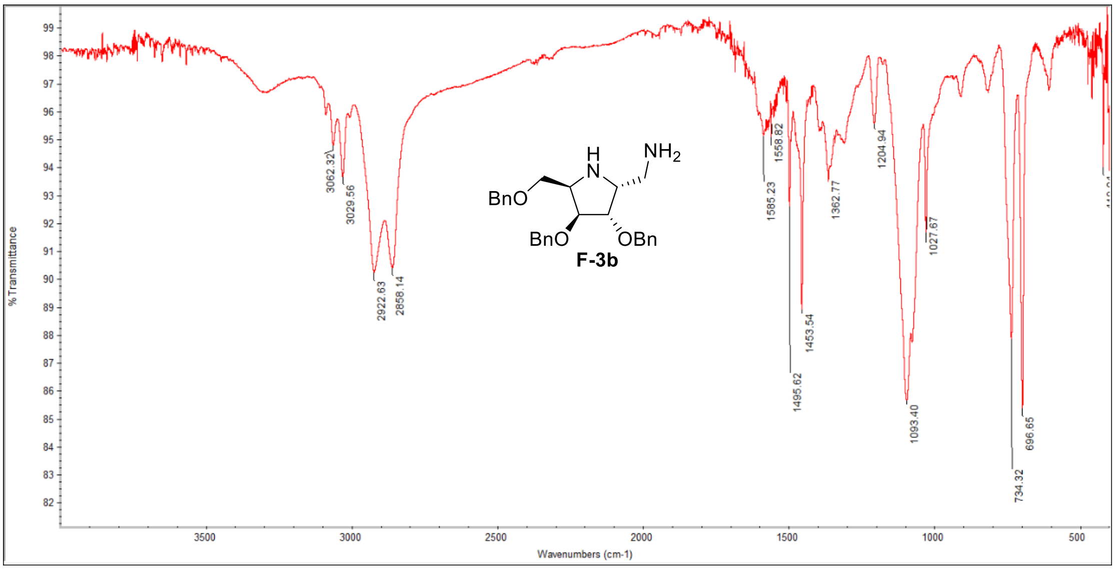

% Transmittance
99 98 97 96 95 94 93 92 91 90 89 88 87 86 85 84 83 82
3062.32
3029.56
2922.63
2858.14
1585.23
1558.82
1495.62
1453.54
1362.77
1204.94
1093.40
1027.67
734.32
696.65
H
N
NH2
BnO
BnO
OBn
F-3b
3500 3000 2500 2000 1500 1000 500
Wavenumbers (cm-1)

Compound **G-3**:

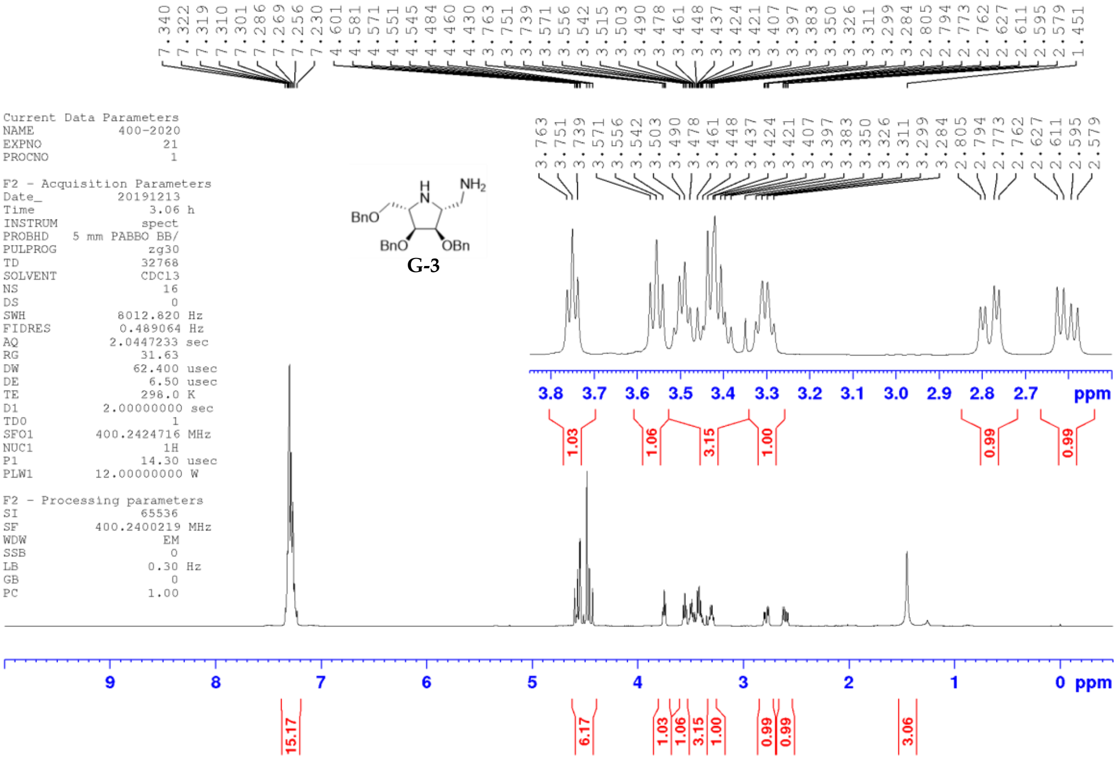
7.340 7.322 7.319 7.310 7.301 7.286 7.269 7.256 7.230 4.601 4.581 4.571 4.551 4.545 4.484 4.460 4.430 3.763 3.751 3.739 3.571 3.556 3.542 3.515 3.503 3.490 3.478 3.461 3.448 3.437 3.424 3.421 3.407 3.397 3.383 3.350 3.326 3.311 3.299 3.284 2.805 2.794 2.773 2.762 2.627 2.611 2.595 2.579 1.451
Current Data Parameters
NAME 400-2020
EXPNO 21
PROCNO 1
F2 - Acquisition Parameters
Date_ 20191213
Time 3.06 h
INSTRUM spect
PROBHD 5 mm PABBO BB/
PULPROG zg30
TD 32768
SOLVENT CDCl3
NS 16
DS 0
SWH 8012.820 Hz
FIDRES 0.489064 Hz
AQ 2.0447233 sec
RG 31.63
DW 62.400 usec
DE 6.50 usec
TE 298.0 K
D1 2.00000000 sec
TD0 1
SFO1 400.2424716 MHz
NUC1 1H
P1 14.30 usec
PLW1 12.00000000 W
F2 - Processing parameters
SI 65536
SF 400.2400219 MHz
WDW EM
SSB 0
LB 0.30 Hz
GB 0
PC 1.00
BnO
H
N
NH2
BnO
OBn
G-3
3.8 3.7 3.6 3.5 3.4 3.3 3.2 3.1 3.0 2.9 2.8 2.7 ppm
1.03 1.06 3.15 1.00 0.99 0.99
9 8 7 6 5 4 3 2 1 0 ppm
15.17 6.17 1.03 1.06 3.15 1.00 0.99 0.99 3.06

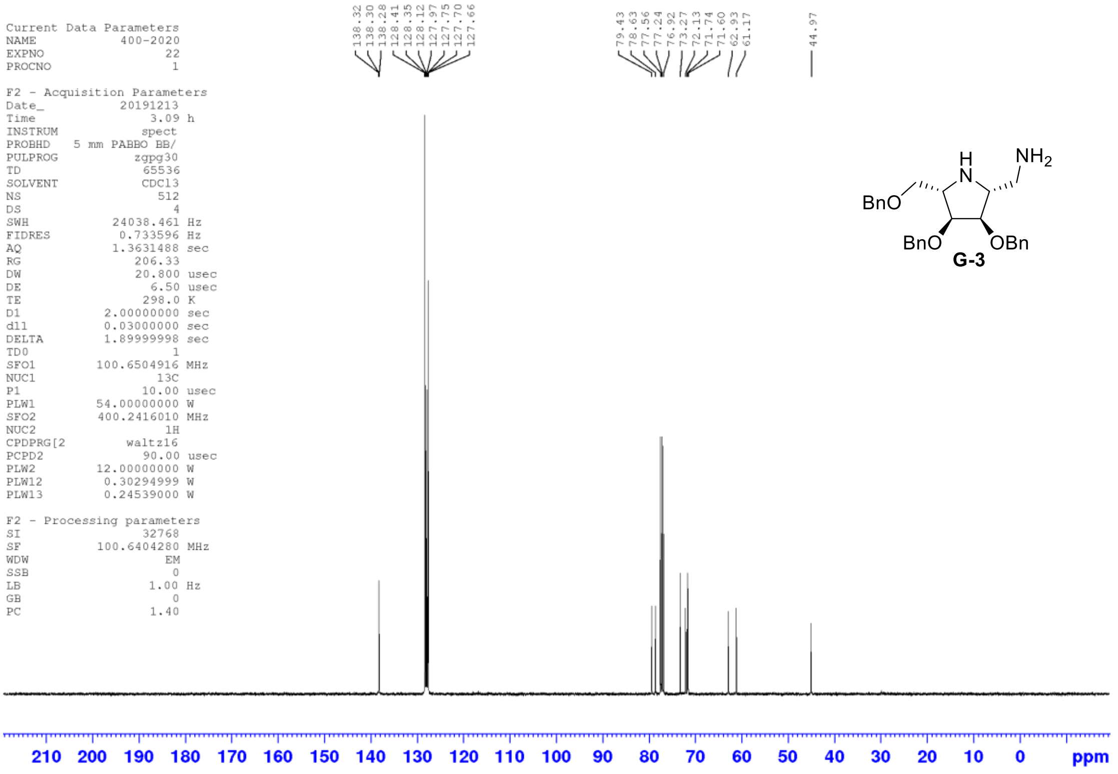
138.32 138.30 138.28 128.41 128.35 128.12 127.97 127.75 127.70 127.66
79.43 78.63 77.56 77.24 76.92 73.27 72.13 71.74 71.60 62.93 61.17
44.97
Current Data Parameters
NAME 400-2020
EXPNO 22
PROCNO 1
F2 - Acquisition Parameters
Date_ 20191213
Time 3.09 h
INSTRUM spect
PROBHD 5 mm PABBO BB/
PULPROG zgpg30
TD 65536
SOLVENT CDCl3
NS 512
DS 4
SWH 24038.461 Hz
FIDRES 0.733596 Hz
AQ 1.3631488 sec
RG 206.33
DW 20.800 usec
DE 6.50 usec
TE 298.0 K
D1 2.00000000 sec
d11 0.03000000 sec
DELTA 1.89999998 sec
TD0 1
SFO1 100.6504916 MHz
NUC1 13C
P1 10.00 usec
PLW1 54.00000000 W
SFO2 400.2416010 MHz
NUC2 1H
CPDPRG[2 waltz16
PCPD2 90.00 usec
PLW2 12.00000000 W
PLW12 0.30294999 W
PLW13 0.24539000 W
F2 - Processing parameters
SI 32768
SF 100.6404280 MHz
WDW EM
SSB 0
LB 1.00 Hz
GB 0
PC 1.40
H
N
NH2
BnO
BnO
OBn
G-3
210 200 190 180 170 160 150 140 130 120 110 100 90 80 70 60 50 40 30 20 10 0 ppm

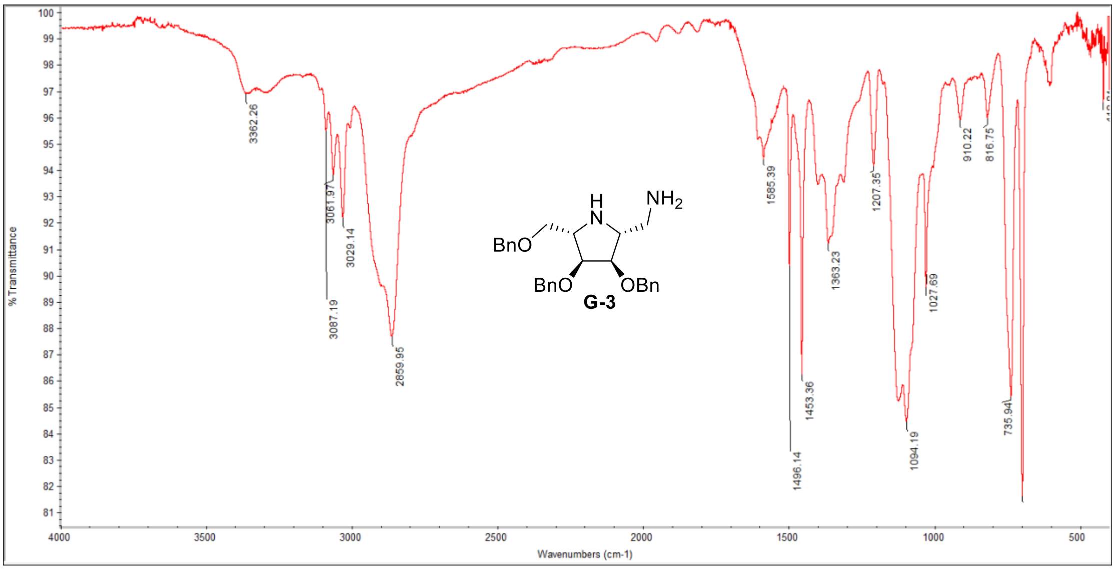
% Transmittance
Wavenumbers (cm-1)
3362.26
3087.19
3061.97
3029.14
2859.95
1585.39
1496.14
1453.36
1363.23
1207.35
1094.19
1027.69
910.22
816.75
735.94
BnO
NH2
BnO
OBn
G-3

Compound **H-3**:

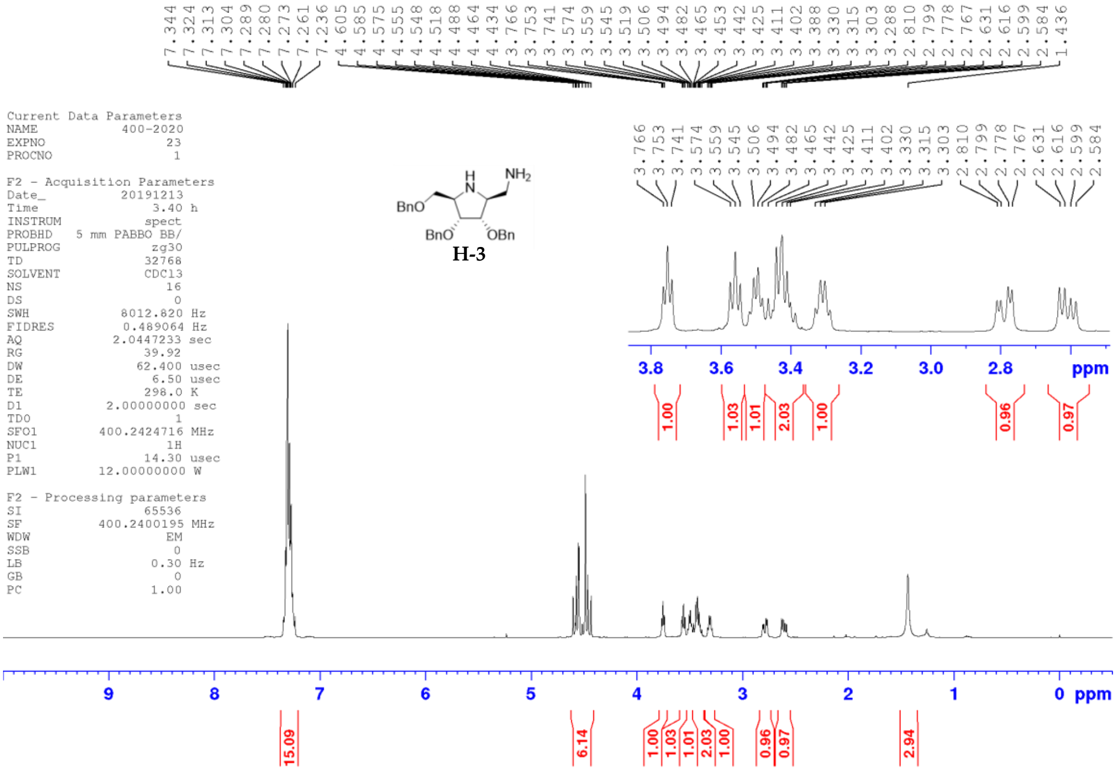
7.344
7.324
7.313
7.304
7.289
7.280
7.273
7.261
7.236
4.605
4.585
4.575
4.555
4.548
4.518
4.488
4.464
4.434
3.766
3.753
3.741
3.574
3.559
3.545
3.519
3.506
3.494
3.482
3.465
3.453
3.442
3.425
3.411
3.402
3.388
3.330
3.315
3.303
3.288
2.810
2.799
2.778
2.767
2.631
2.616
2.599
2.584
1.436
Current Data Parameters
NAME 400-2020
EXPNO 23
PROCNO 1
F2 - Acquisition Parameters
Date_ 20191213
Time 3.40 h
INSTRUM spect
PROBHD 5 mm PABBO BB/
PULPROG zg30
TD 32768
SOLVENT CDCl3
NS 16
DS 0
SWH 8012.820 Hz
FIDRES 0.489064 Hz
AQ 2.0447233 sec
RG 39.92
DW 62.400 usec
DE 6.50 usec
TE 298.0 K
D1 2.00000000 sec
TD0 1
SFO1 400.2424716 MHz
NUC1 1H
P1 14.30 usec
PLW1 12.00000000 W
F2 - Processing parameters
SI 65536
SF 400.2400195 MHz
WDW EM
SSB 0
LB 0.30 Hz
GB 0
PC 1.00
BnO
NH2
BnO
OBn
H-3
ppm
15.09
6.14
1.00
1.03
1.01
2.03
1.00
0.96
0.97
2.94

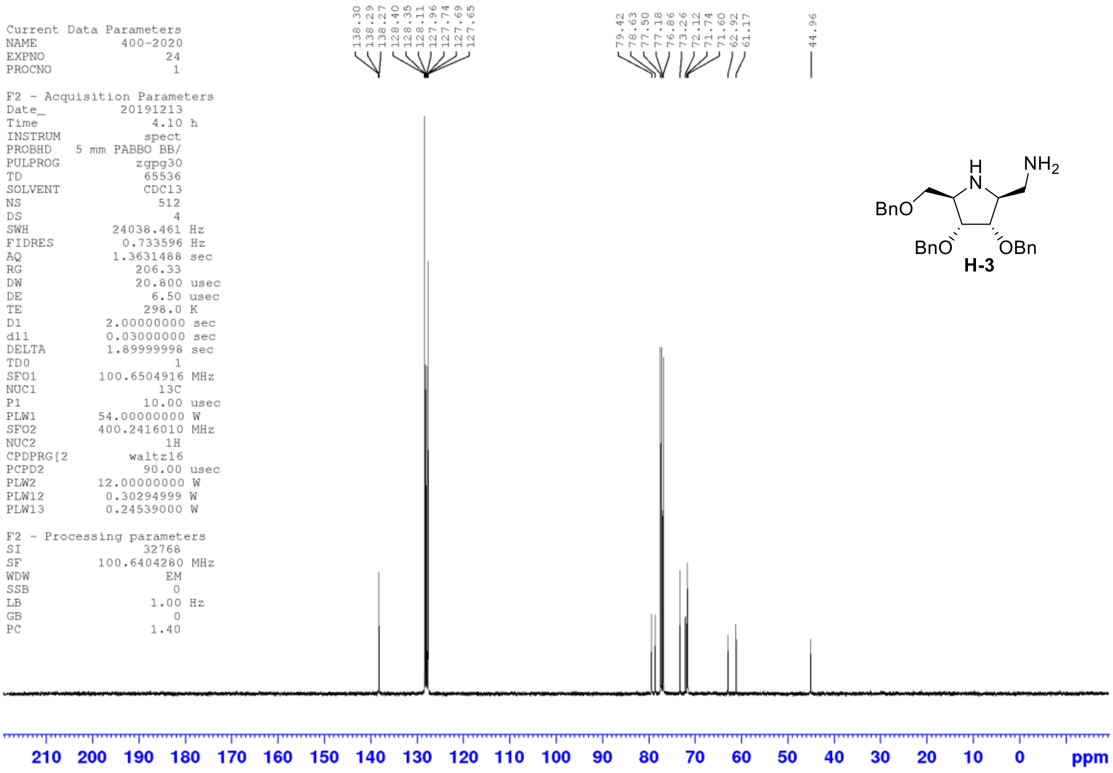
Current Data Parameters
NAME 400-2020
EXPNO 24
PROCNO 1
F2 - Acquisition Parameters
Date_ 20191213
Time 4.10 h
INSTRUM spect
PROBHD 5 mm PABBO BB/
PULPROG zgpg30
TD 65536
SOLVENT CDC13
NS 512
DS 4
SWH 24038.461 Hz
FIDRES 0.733596 Hz
AQ 1.3631488 sec
RG 206.33
DW 20.800 usec
DE 6.50 usec
TE 298.0 K
D1 2.00000000 sec
d11 0.03000000 sec
DELTA 1.89999998 sec
TD0 1
SFO1 100.6504916 MHz
NUC1 13C
P1 10.00 usec
PLW1 54.00000000 W
SFO2 400.2416010 MHz
NUC2 1H
CPDPRG[2 waltz16
PCPD2 90.00 usec
PLW2 12.00000000 W
PLW12 0.30294999 W
PLW13 0.24539000 W
F2 - Processing parameters
SI 32768
SF 100.6404280 MHz
WDW EM
SSB 0
LB 1.00 Hz
GB 0
PC 1.40
138.30
138.29
138.27
128.40
128.35
128.11
127.96
127.74
127.69
127.65
79.42
78.63
77.50
77.18
76.86
73.26
72.12
71.74
71.60
62.92
61.17
44.96
BnO
BnO
OBn
NH2
H
N
H-3
210 200 190 180 170 160 150 140 130 120 110 100 90 80 70 60 50 40 30 20 10 0 ppm

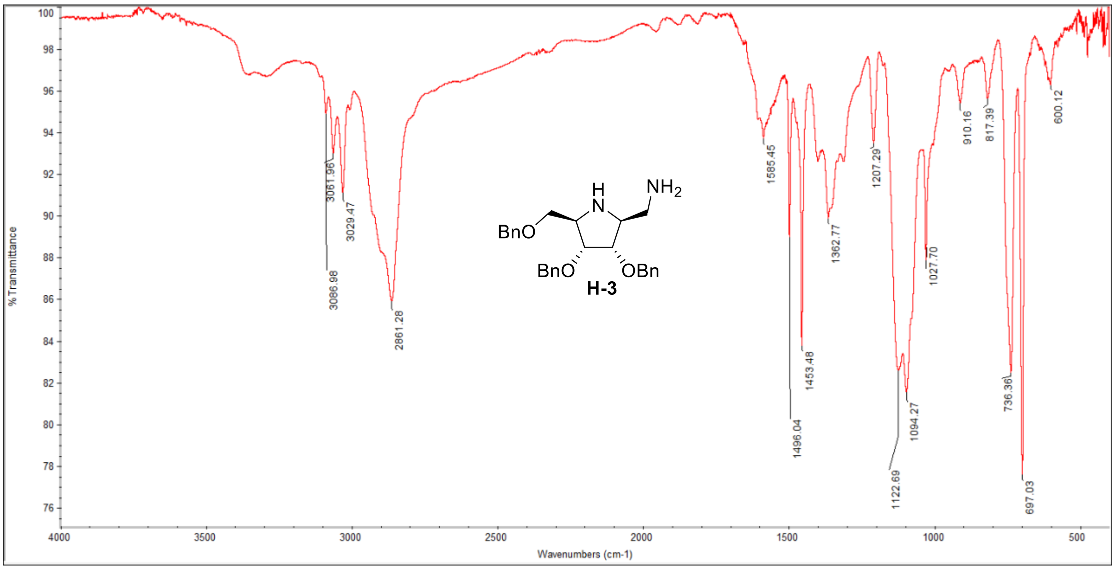
% Transmittance
Wavenumbers (cm-1)
100 98 96 94 92 90 88 86 84 82 80 78 76
4000 3500 3000 2500 2000 1500 1000 500
3086.98
3061.96
3029.47
2861.28
1585.45
1496.04
1453.48
1362.77
1207.29
1122.69
1094.27
1027.70
910.16
817.39
736.36
697.03
600.12
BnO
BnO
OBn
NH2
H
N
H-3

Compound **A-6**:

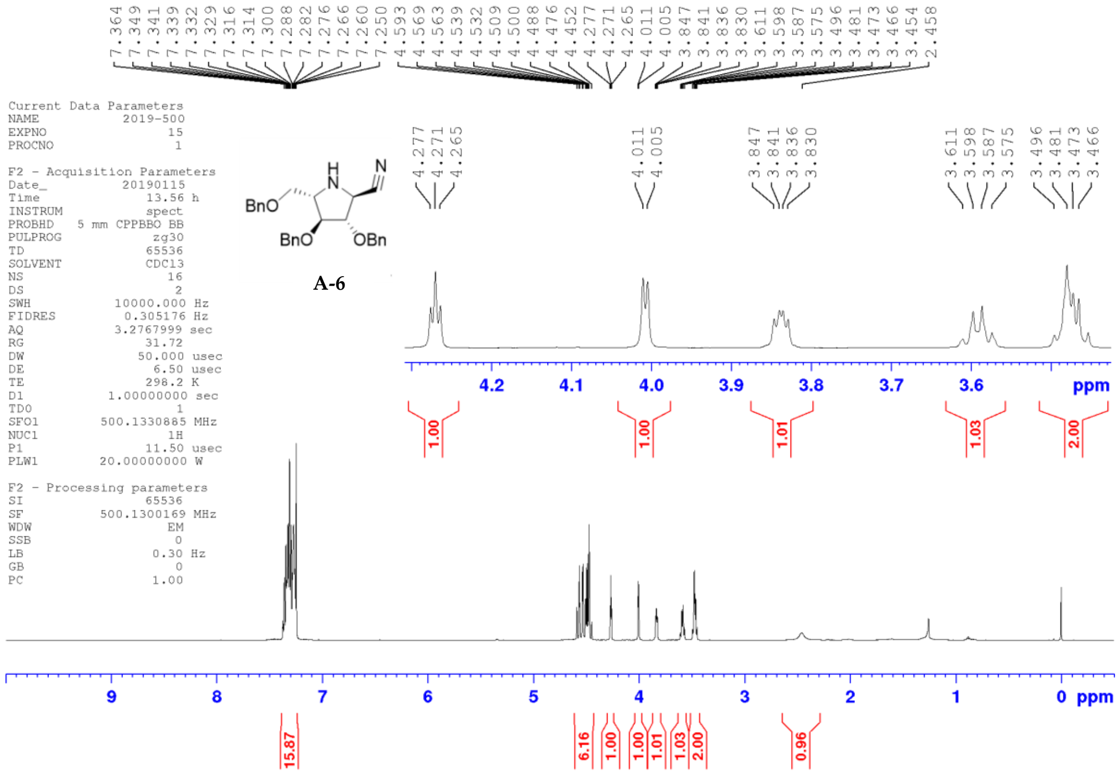
7.364 7.349 7.341 7.339 7.332 7.329 7.316 7.314 7.300 7.288 7.282 7.276 7.266 7.260 7.250 4.593 4.569 4.563 4.539 4.532 4.509 4.500 4.488 4.476 4.452 4.277 4.271 4.265 4.011 4.005 3.847 3.841 3.836 3.830 3.611 3.598 3.587 3.575 3.496 3.481 3.473 3.466 3.454 2.458
Current Data Parameters
NAME 2019-500
EXPNO 15
PROCNO 1
F2 - Acquisition Parameters
Date_ 20190115
Time 13.56 h
INSTRUM spect
PROBHD 5 mm CPPBBO BB
PULPROG zg30
TD 65536
SOLVENT CDCl3
NS 16
DS 2
SWH 10000.000 Hz
FIDRES 0.305176 Hz
AQ 3.2767999 sec
RG 31.72
DW 50.000 usec
DE 6.50 usec
TE 298.2 K
D1 1.00000000 sec
TD0 1
SFO1 500.1330885 MHz
NUC1 1H
P1 11.50 usec
PLW1 20.00000000 W
F2 - Processing parameters
SI 65536
SF 500.1300169 MHz
WDW EM
SSB 0
LB 0.30 Hz
GB 0
PC 1.00
BnO
N
H
N
BnO
OBn
A-6
4.277 4.271 4.265
4.011 4.005
3.847 3.841 3.836 3.830
3.611 3.598 3.587 3.575
3.496 3.481 3.473 3.466
4.2 4.1 4.0 3.9 3.8 3.7 3.6 ppm
1.00 1.00 1.01 1.03 2.00
9 8 7 6 5 4 3 2 1 0 ppm
15.87 6.16 1.00 1.00 1.01 1.03 2.00 0.96

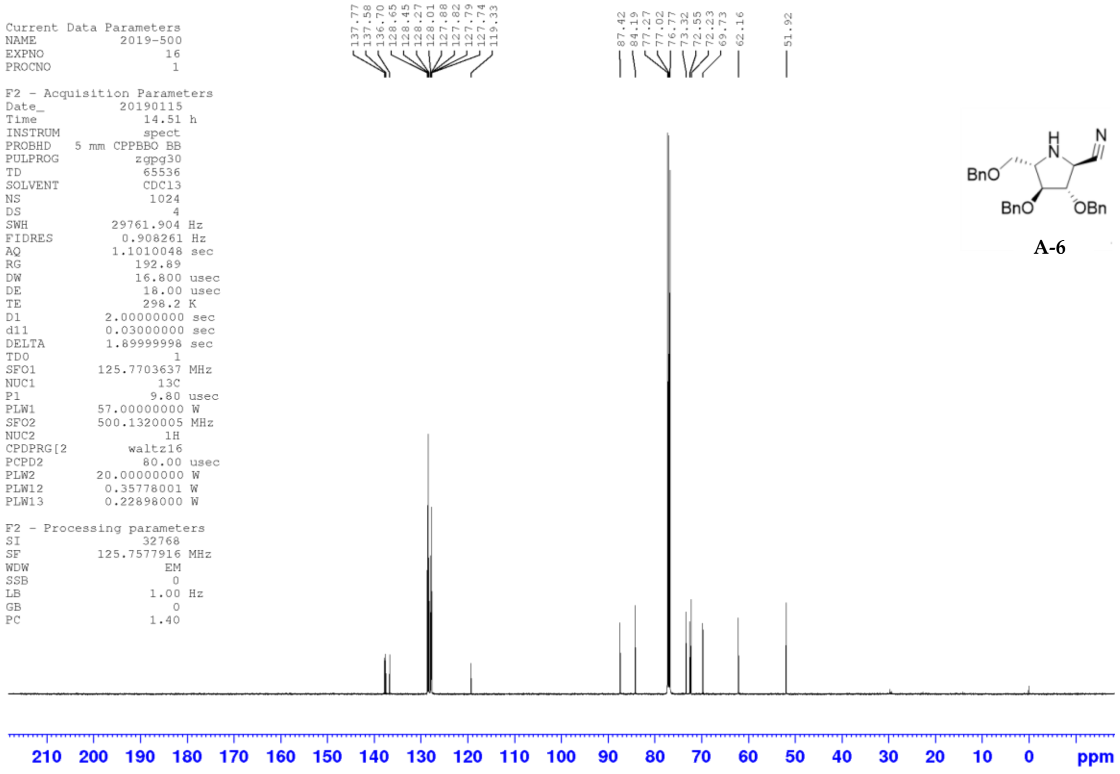
137.77 137.58 136.70 128.65 128.45 128.27 128.01 127.88 127.82 127.79 127.74 119.33
87.42 84.19 77.27 77.02 76.77 73.32 72.55 72.23 69.73 62.16 51.92
Current Data Parameters
NAME 2019-500
EXPNO 16
PROCNO 1
F2 - Acquisition Parameters
Date_ 20190115
Time 14.51 h
INSTRUM spect
PROBHD 5 mm CPPBBO BB
PULPROG zgpg30
TD 65536
SOLVENT CDCl3
NS 1024
DS 4
SWH 29761.904 Hz
FIDRES 0.908261 Hz
AQ 1.1010048 sec
RG 192.89
DW 16.800 usec
DE 18.00 usec
TE 298.2 K
D1 2.00000000 sec
d11 0.03000000 sec
DELTA 1.89999998 sec
TD0 1
SFO1 125.7703637 MHz
NUC1 13C
P1 9.80 usec
PLW1 57.00000000 W
SFO2 500.1320005 MHz
NUC2 1H
CPDPRG[2 waltz16
PCPD2 80.00 usec
PLW2 20.00000000 W
PLW12 0.35778001 W
PLW13 0.22898000 W
F2 - Processing parameters
SI 32768
SF 125.7577916 MHz
WDW EM
SSB 0
LB 1.00 Hz
GB 0
PC 1.40
BnO
N
H
N
BnO
OBn
A-6
210 200 190 180 170 160 150 140 130 120 110 100 90 80 70 60 50 40 30 20 10 0 ppm

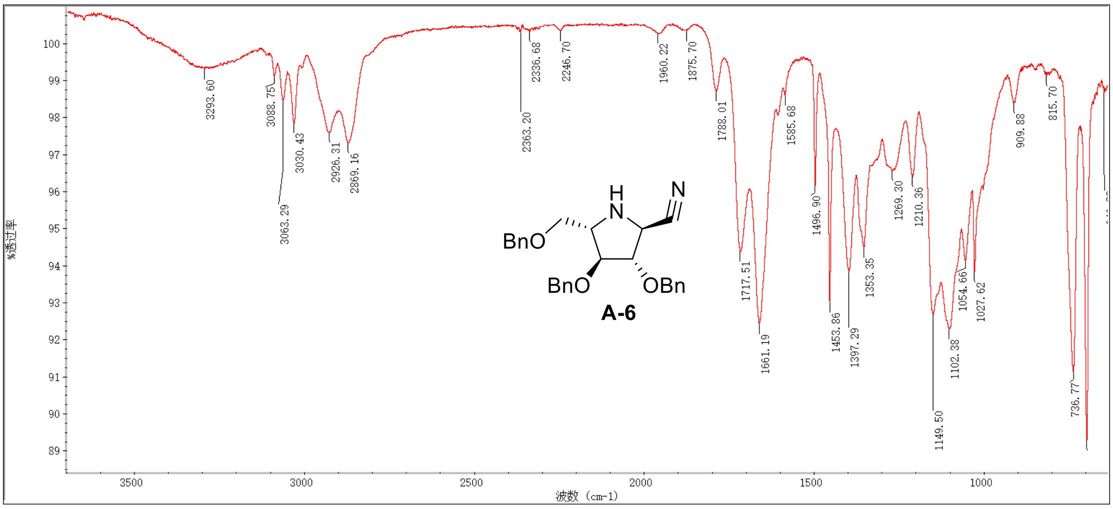
A-6
BnO
BnO
OBn
波数 (cm-1)
%透过率

Compound **A-8**:

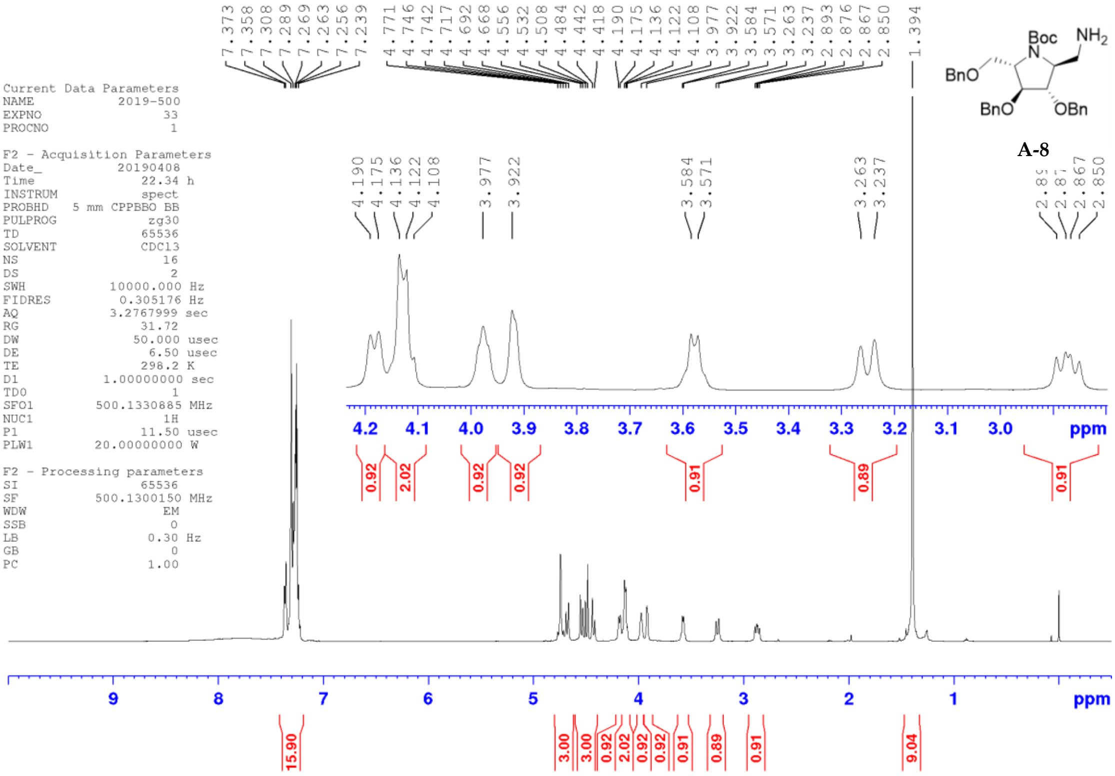
A-8
Boc
NH_2
BnO
BnO
OBn
Current Data Parameters
NAME 2019-500
EXPNO 33
PROCNO 1
F2 - Acquisition Parameters
Date_ 20190408
Time 22.34 h
INSTRUM spect
PROBHD 5 mm CPPBBO BB
PULPROG zg30
TD 65536
SOLVENT CDCl3
NS 16
DS 2
SWH 10000.000 Hz
FIDRES 0.305176 Hz
AQ 3.2767999 sec
RG 31.72
DW 50.000 usec
DE 6.50 usec
TE 298.2 K
D1 1.00000000 sec
TD0 1
SFO1 500.1330885 MHz
NUC1 1H
P1 11.50 usec
PLW1 20.00000000 W
F2 - Processing parameters
SI 65536
SF 500.1300150 MHz
WDW EM
SSB 0
LB 0.30 Hz
GB 0
PC 1.00
ppm

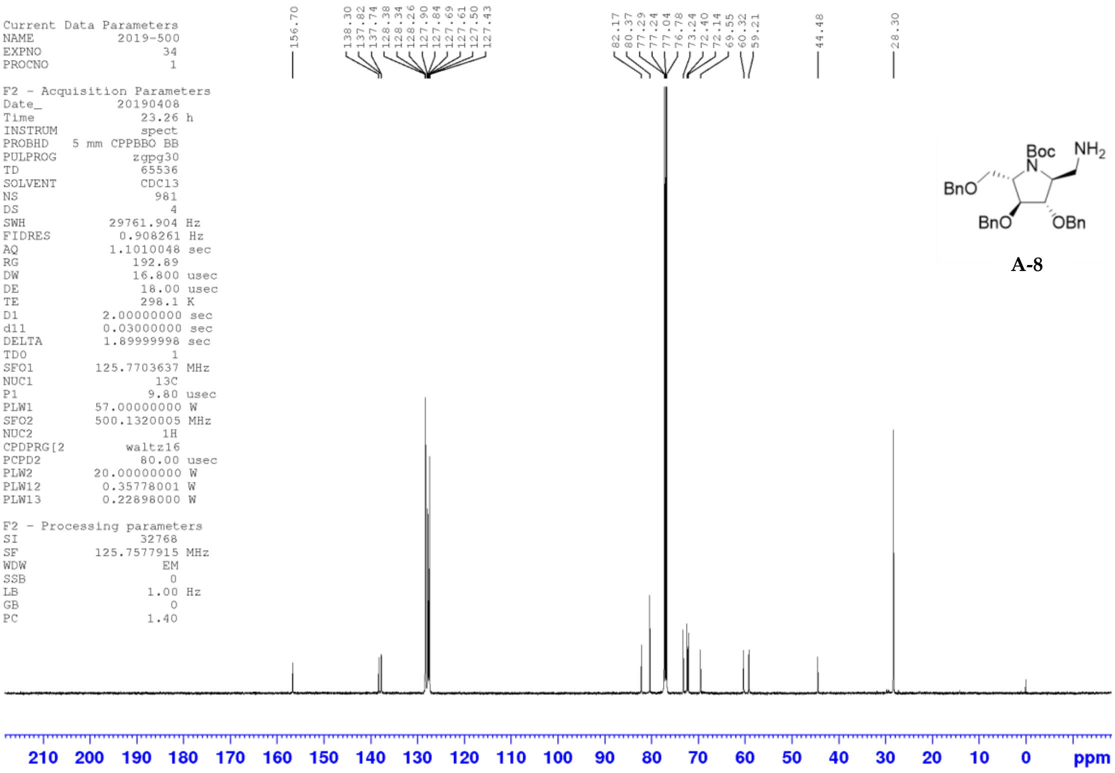
Current Data Parameters
NAME 2019-500
EXPNO 34
PROCNO 1
F2 - Acquisition Parameters
Date_ 20190408
Time 23.26 h
INSTRUM spect
PROBHD 5 mm CPPBBO BB
PULPROG zgpg30
TD 65536
SOLVENT CDCl3
NS 981
DS 4
SWH 29761.904 Hz
FIDRES 0.908261 Hz
AQ 1.1010048 sec
RG 192.89
DW 16.800 usec
DE 18.00 usec
TE 298.1 K
D1 2.00000000 sec
d11 0.03000000 sec
DELTA 1.89999998 sec
TD0 1
SFO1 125.7703637 MHz
NUC1 13C
P1 9.80 usec
PLW1 57.00000000 W
SFO2 500.1320005 MHz
NUC2 1H
CPDPRG[2 waltz16
PCPD2 80.00 usec
PLW2 20.00000000 W
PLW12 0.35778001 W
PLW13 0.22898000 W
F2 - Processing parameters
SI 32768
SF 125.7577915 MHz
WDW EM
SSB 0
LB 1.00 Hz
GB 0
PC 1.40
156.70
138.30
137.82
137.74
128.38
128.34
128.26
127.90
127.84
127.69
127.61
127.50
127.43
82.17
80.37
77.29
77.24
77.04
76.78
73.24
72.40
72.14
69.55
60.32
59.21
44.48
28.30
Boc
NH2
BnO
N
BnO
OBn
A-8
210 200 190 180 170 160 150 140 130 120 110 100 90 80 70 60 50 40 30 20 10 0 ppm

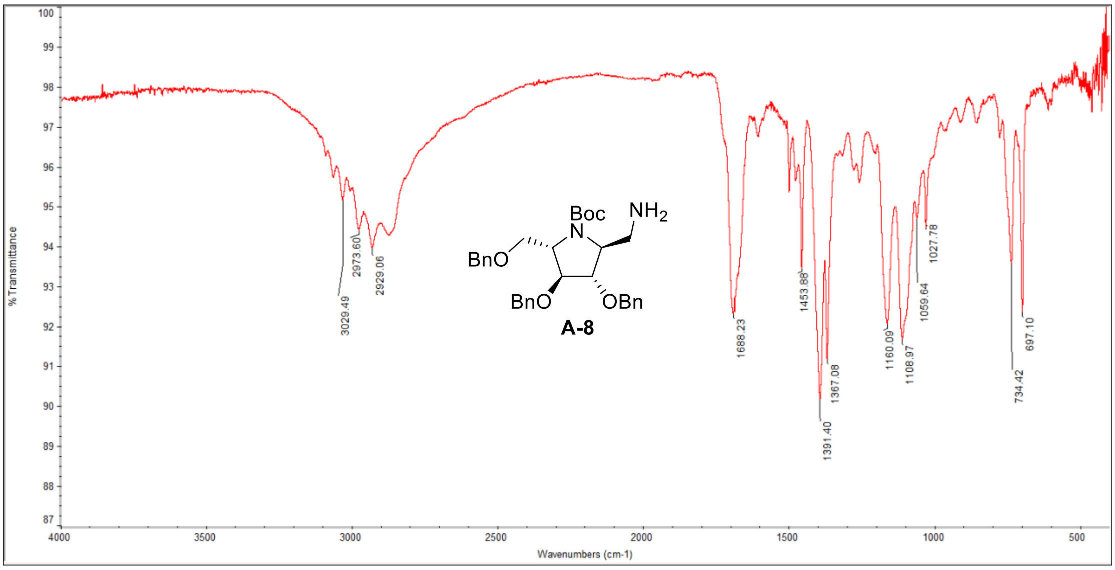
% Transmittance
100
99
98
97
96
95
94
93
92
91
90
89
88
87
3029.49
2973.60
2929.06
1688.23
1453.88
1391.40
1367.08
1160.09
1108.97
1059.64
1027.78
734.42
697.10
Boc
NH2
N
BnO
BnO
OBn
A-8
4000 3500 3000 2500 2000 1500 1000 500
Wavenumbers (cm-1)

Compound **A-9**:

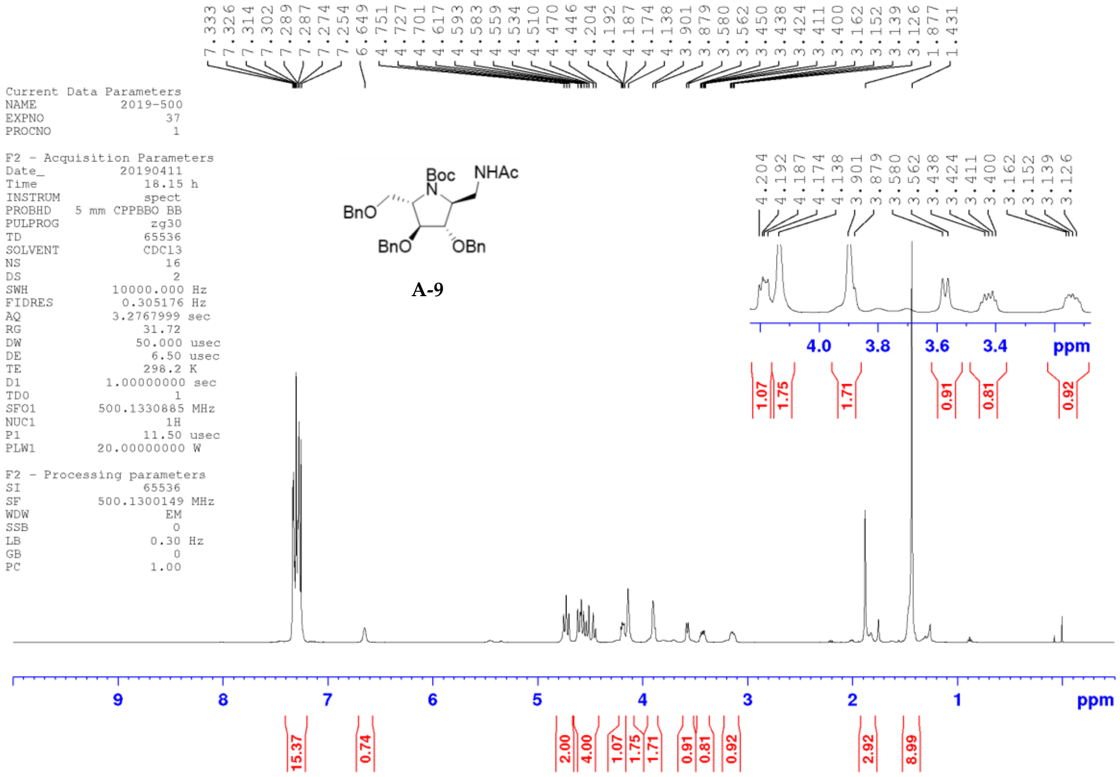
7.333
7.326
7.314
7.302
7.289
7.287
7.274
7.254
6.649
4.751
4.727
4.701
4.617
4.593
4.583
4.559
4.534
4.510
4.470
4.446
4.204
4.192
4.187
4.174
4.138
3.901
3.879
3.580
3.562
3.450
3.438
3.424
3.411
3.400
3.162
3.152
3.139
3.126
1.877
1.431
Current Data Parameters
NAME 2019-500
EXPNO 37
PROCNO 1
F2 - Acquisition Parameters
Date_ 20190411
Time 18.15 h
INSTRUM spect
PROBHD 5 mm CPPBBO BB
PULPROG zg30
TD 65536
SOLVENT CDCl3
NS 16
DS 2
SWH 10000.000 Hz
FIDRES 0.305176 Hz
AQ 3.2767999 sec
RG 31.72
DW 50.000 usec
DE 6.50 usec
TE 298.2 K
D1 1.00000000 sec
TD0 1
SFO1 500.1330885 MHz
NUC1 1H
P1 11.50 usec
PLW1 20.00000000 W
F2 - Processing parameters
SI 65536
SF 500.1300149 MHz
WDW EM
SSB 0
LB 0.30 Hz
GB 0
PC 1.00
Boc
NHAc
N
BnO
BnO
OBn
A-9
4.204
4.192
4.187
4.174
4.138
3.901
3.879
3.580
3.562
3.438
3.424
3.411
3.400
3.162
3.152
3.139
3.126
4.0
3.8
3.6
3.4
ppm
1.07
1.75
1.71
0.91
0.81
0.92
9
8
7
6
5
4
3
2
1
ppm
15.37
0.74
2.00
4.00
1.07
1.75
1.71
0.91
0.81
0.92
2.92
8.99

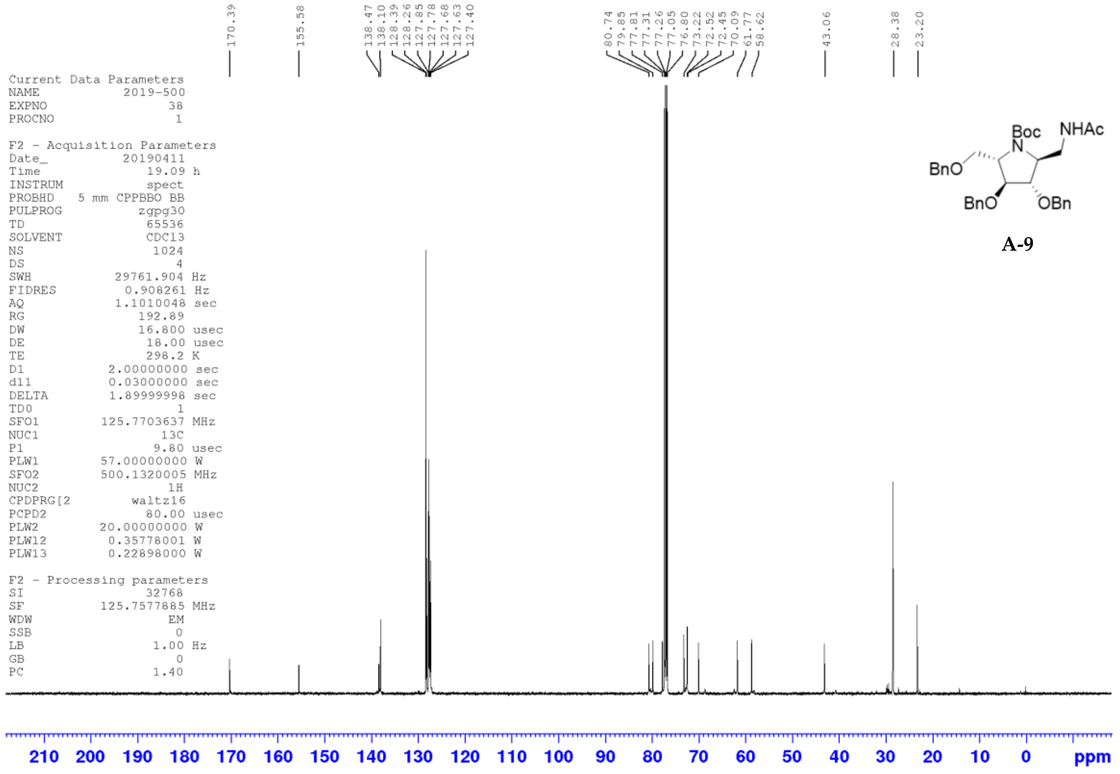
170.39
155.58
138.47
138.10
128.39
128.26
127.85
127.78
127.68
127.63
127.40
80.74
79.85
77.81
77.31
77.26
77.05
76.80
73.22
72.52
72.45
70.09
61.77
58.62
43.06
28.38
23.20
Current Data Parameters
NAME 2019-500
EXPNO 38
PROCNO 1
F2 - Acquisition Parameters
Date_ 20190411
Time 19.09 h
INSTRUM spect
PROBHD 5 mm CPPBBO BB
PULPROG zgpg30
TD 65536
SOLVENT CDCl3
NS 1024
DS 4
SWH 29761.904 Hz
FIDRES 0.908261 Hz
AQ 1.1010048 sec
RG 192.89
DW 16.800 usec
DE 18.00 usec
TE 298.2 K
D1 2.00000000 sec
d11 0.03000000 sec
DELTA 1.89999998 sec
TD0 1
SFO1 125.7703637 MHz
NUC1 13C
P1 9.80 usec
PLW1 57.00000000 W
SFO2 500.1320005 MHz
NUC2 1H
CPDPRG[2 waltz16
PCPD2 80.00 usec
PLW2 20.00000000 W
PLW12 0.35778001 W
PLW13 0.22898000 W
F2 - Processing parameters
SI 32768
SF 125.7577885 MHz
WDW EM
SSB 0
LB 1.00 Hz
GB 0
PC 1.40
Boc
NHAc
N
BnO
BnO
OBn
A-9
210
200
190
180
170
160
150
140
130
120
110
100
90
80
70
60
50
40
30
20
10
0
ppm

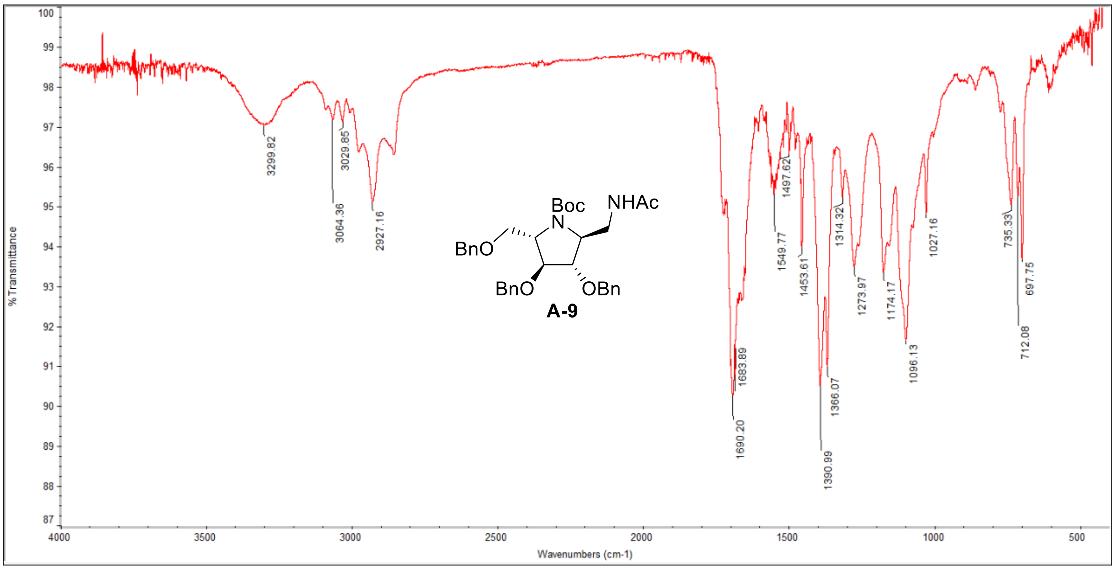

Compound **A-5**:

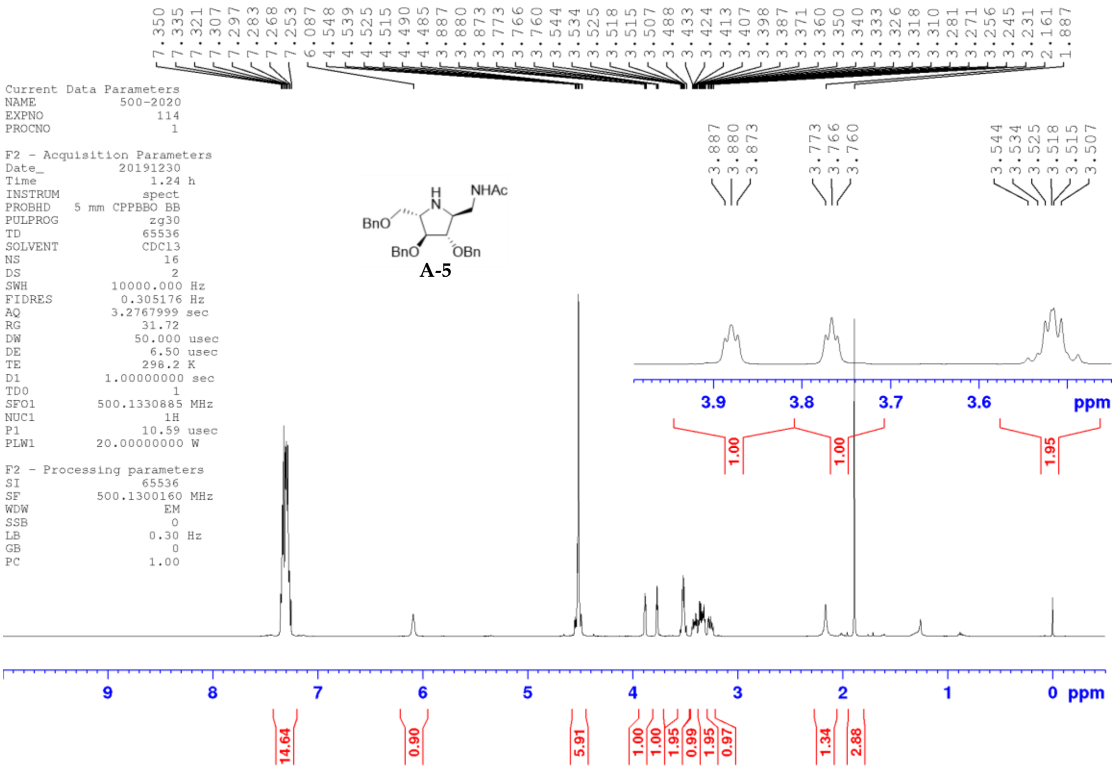

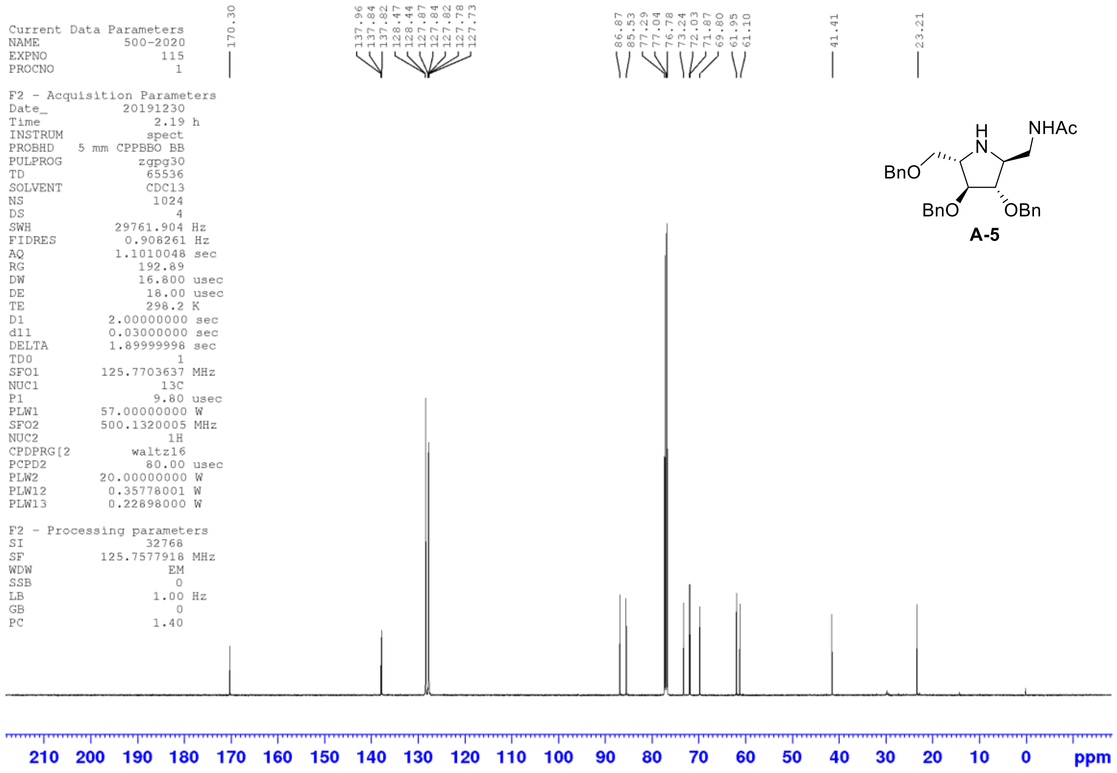
Current Data Parameters
NAME 500-2020
EXPNO 115
PROCNO 1
F2 - Acquisition Parameters
Date_ 20191230
Time 2.19 h
INSTRUM spect
PROBHD 5 mm CPPBBO BB
PULPROG zgpg30
TD 65536
SOLVENT CDCl3
NS 1024
DS 4
SWH 29761.904 Hz
FIDRES 0.908261 Hz
AQ 1.1010048 sec
RG 192.89
DW 16.800 usec
DE 18.00 usec
TE 298.2 K
D1 2.00000000 sec
d11 0.03000000 sec
DELTA 1.89999998 sec
TD0 1
SFO1 125.7703637 MHz
NUC1 13C
P1 9.80 usec
PLW1 57.00000000 W
SFO2 500.1320005 MHz
NUC2 1H
CPDPRG[2 waltz16
PCPD2 80.00 usec
PLW2 20.00000000 W
PLW12 0.35778001 W
PLW13 0.22898000 W
F2 - Processing parameters
SI 32768
SF 125.7577918 MHz
WDW EM
SSB 0
LB 1.00 Hz
GB 0
PC 1.40
170.30
137.96
137.84
137.82
128.47
128.44
127.87
127.84
127.82
127.78
127.73
86.87
85.53
77.29
77.04
76.78
73.24
72.03
71.87
69.80
61.95
61.10
41.41
23.21
H
N
NHAc
BnO
BnO
OBn
A-5
210 200 190 180 170 160 150 140 130 120 110 100 90 80 70 60 50 40 30 20 10 0 ppm

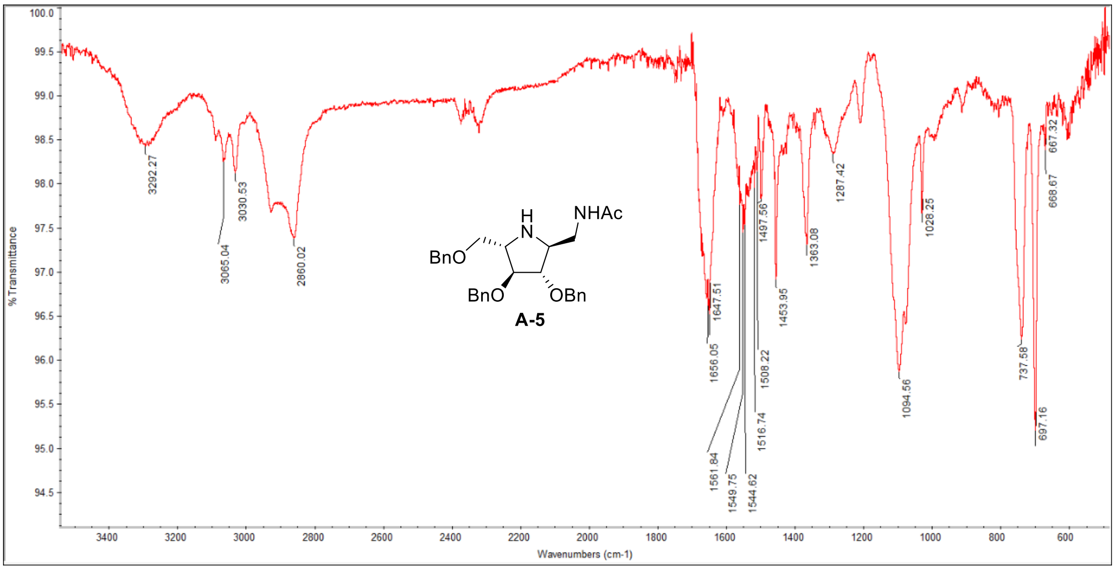
% Transmittance
Wavenumbers (cm-1)
3292.27
3065.04
3030.53
2860.02
1656.05
1647.51
1561.84
1549.75
1544.62
1516.74
1508.22
1497.56
1453.95
1363.08
1287.42
1094.56
1028.25
737.58
697.16
668.67
667.32
H
N
NHAc
BnO
BnO
OBn
A-5

Compound **B-5**:

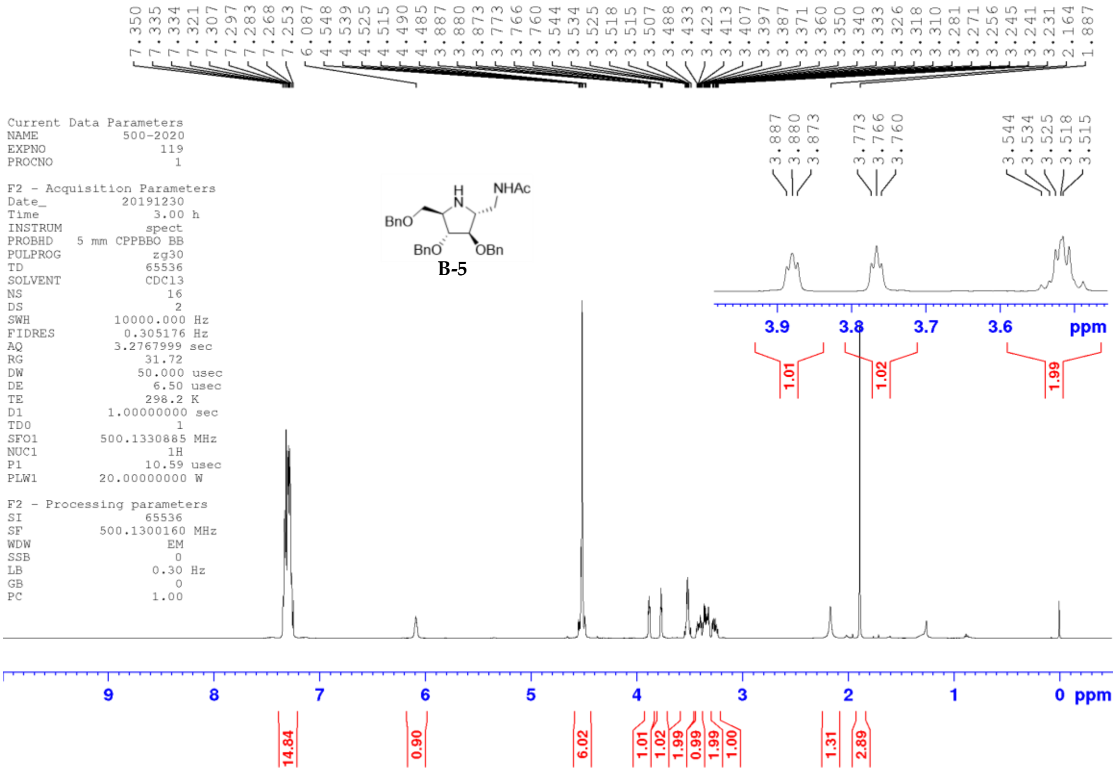
7.350
7.335
7.334
7.321
7.307
7.297
7.283
7.268
7.253
6.087
4.548
4.539
4.525
4.515
4.490
4.485
3.887
3.880
3.873
3.773
3.766
3.760
3.544
3.534
3.525
3.518
3.515
3.507
3.488
3.433
3.423
3.413
3.407
3.397
3.387
3.371
3.360
3.350
3.340
3.333
3.326
3.318
3.310
3.281
3.271
3.256
3.245
3.241
3.231
2.164
1.887
Current Data Parameters
NAME 500-2020
EXPNO 119
PROCNO 1
F2 - Acquisition Parameters
Date_ 20191230
Time 3.00 h
INSTRUM spect
PROBHD 5 mm CPPBBO BB
PULPROG zg30
TD 65536
SOLVENT CDCl3
NS 16
DS 2
SWH 10000.000 Hz
FIDRES 0.305176 Hz
AQ 3.2767999 sec
RG 31.72
DW 50.000 usec
DE 6.50 usec
TE 298.2 K
D1 1.00000000 sec
TD0 1
SFO1 500.1330885 MHz
NUC1 1H
P1 10.59 usec
PLW1 20.00000000 W
F2 - Processing parameters
SI 65536
SF 500.1300160 MHz
WDW EM
SSB 0
LB 0.30 Hz
GB 0
PC 1.00
BnO
H
N
NHAc
BnO
OBn
B-5
3.9
3.8
3.7
3.6
ppm
1.01
1.02
1.99
9
8
7
6
5
4
3
2
1
0
ppm
14.84
0.90
6.02
1.01
1.02
1.99
0.99
1.99
1.00
1.31
2.89

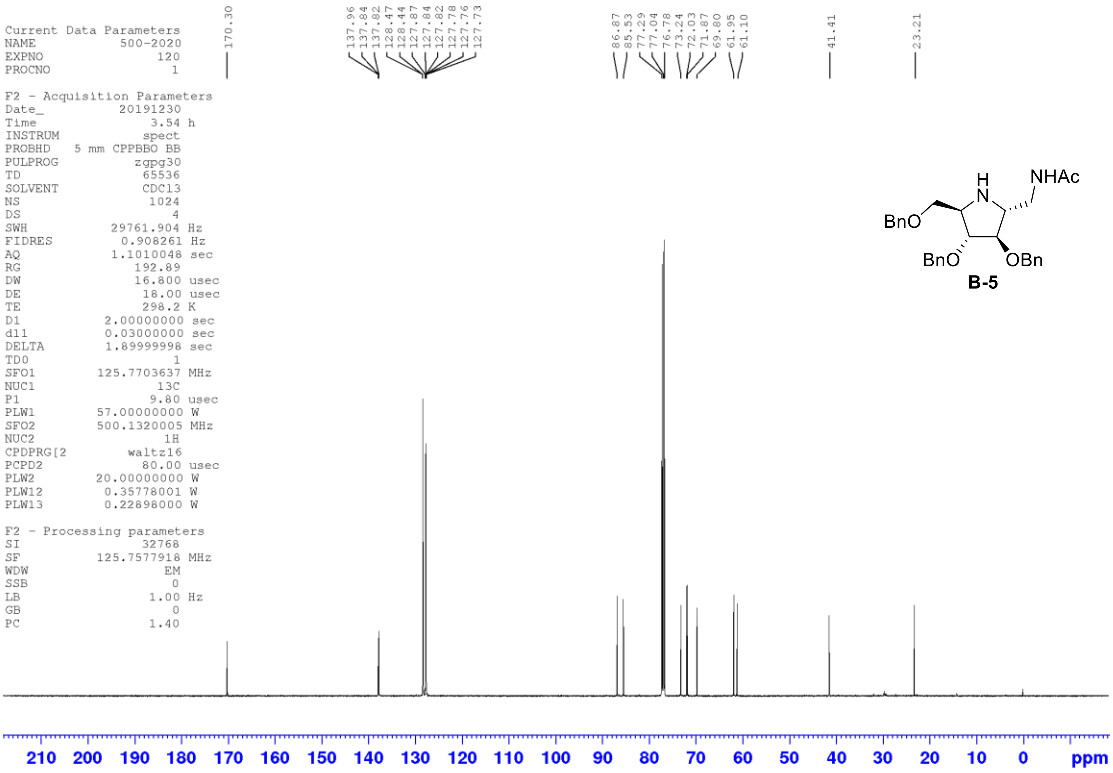
170.30
137.96
137.84
137.82
128.47
128.44
127.87
127.84
127.82
127.78
127.76
127.73
86.87
85.53
77.29
77.04
76.78
73.24
72.03
71.87
69.80
61.95
61.10
41.41
23.21
Current Data Parameters
NAME 500-2020
EXPNO 120
PROCNO 1
F2 - Acquisition Parameters
Date_ 20191230
Time 3.54 h
INSTRUM spect
PROBHD 5 mm CPPBBO BB
PULPROG zgpg30
TD 65536
SOLVENT CDCl3
NS 1024
DS 4
SWH 29761.904 Hz
FIDRES 0.908261 Hz
AQ 1.1010048 sec
RG 192.89
DW 16.800 usec
DE 18.00 usec
TE 298.2 K
D1 2.00000000 sec
d11 0.03000000 sec
DELTA 1.89999998 sec
TD0 1
SFO1 125.7703637 MHz
NUC1 13C
P1 9.80 usec
PLW1 57.00000000 W
SFO2 500.1320005 MHz
NUC2 1H
CPDPRG[2 waltz16
PCPD2 80.00 usec
PLW2 20.00000000 W
PLW12 0.35778001 W
PLW13 0.22898000 W
F2 - Processing parameters
SI 32768
SF 125.7577918 MHz
WDW EM
SSB 0
LB 1.00 Hz
GB 0
PC 1.40
H
N
NHAc
BnO
BnO
OBn
B-5
210
200
190
180
170
160
150
140
130
120
110
100
90
80
70
60
50
40
30
20
10
0
ppm

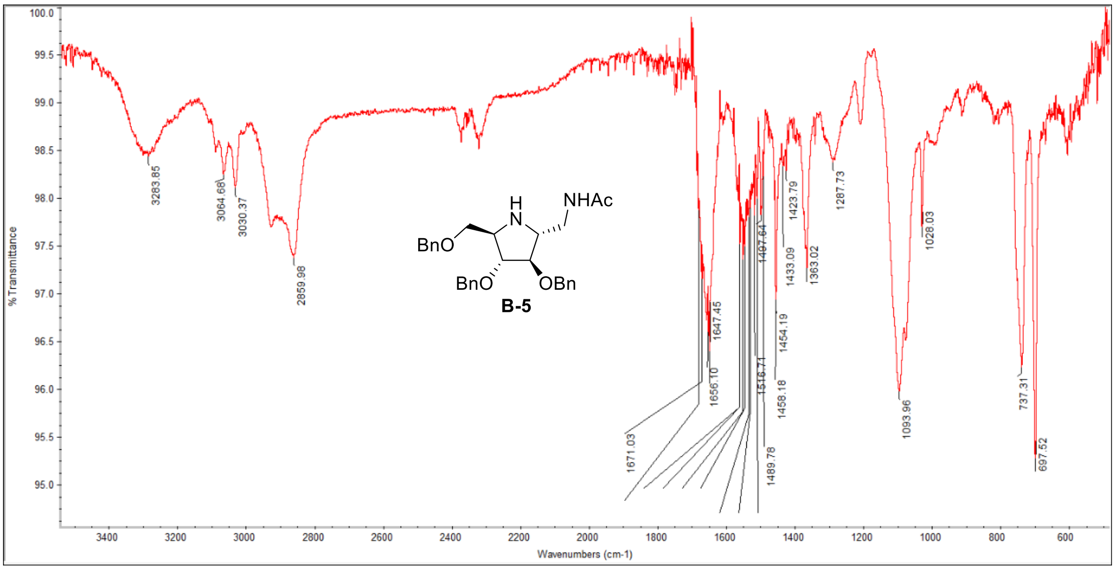
% Transmittance
Wavenumbers (cm-1)
BnO
BnO
OBn
NHAc
B-5

Compound **C-5**:

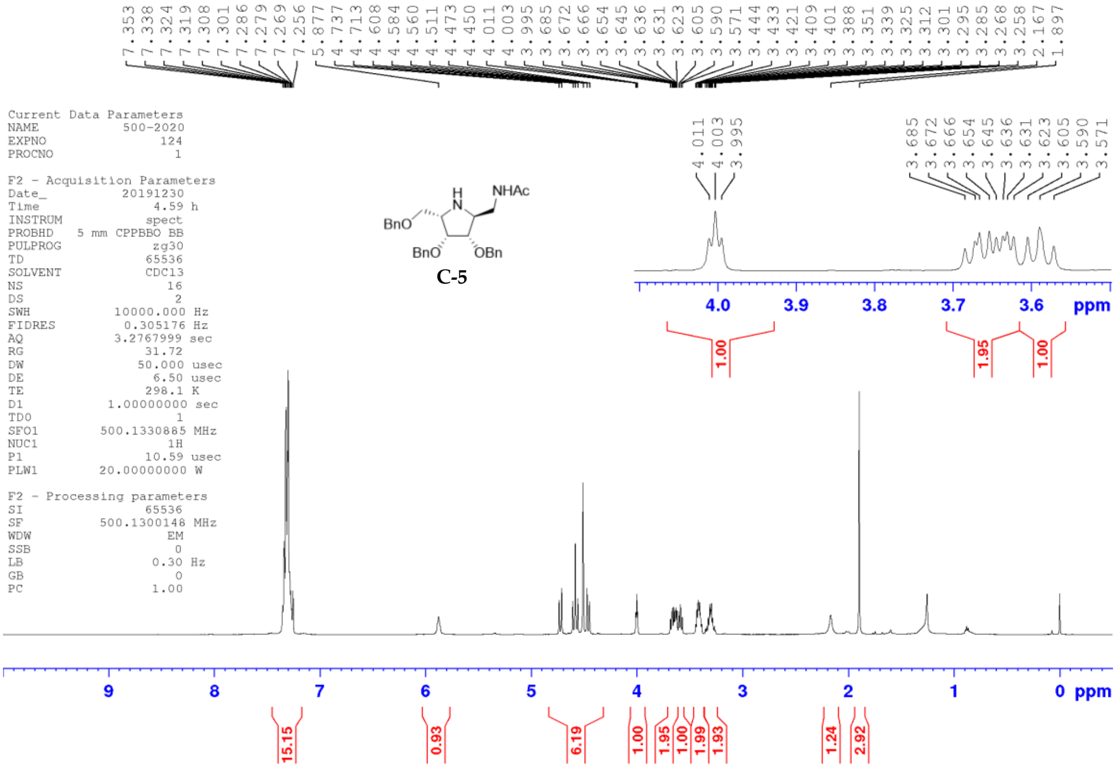
Current Data Parameters
NAME 500-2020
EXPNO 124
PROCNO 1
F2 - Acquisition Parameters
Date_ 20191230
Time 4.59 h
INSTRUM spect
PROBHD 5 mm CPPBBO BB
PULPROG zg30
TD 65536
SOLVENT CDCl3
NS 16
DS 2
SWH 10000.000 Hz
FIDRES 0.305176 Hz
AQ 3.2767999 sec
RG 31.72
DW 50.000 usec
DE 6.50 usec
TE 298.1 K
D1 1.00000000 sec
TD0 1
SFO1 500.1330885 MHz
NUC1 1H
P1 10.59 usec
PLW1 20.00000000 W
F2 - Processing parameters
SI 65536
SF 500.1300148 MHz
WDW EM
SSB 0
LB 0.30 Hz
GB 0
PC 1.00
BnO
BnO
OBn
NHAc
C-5
ppm

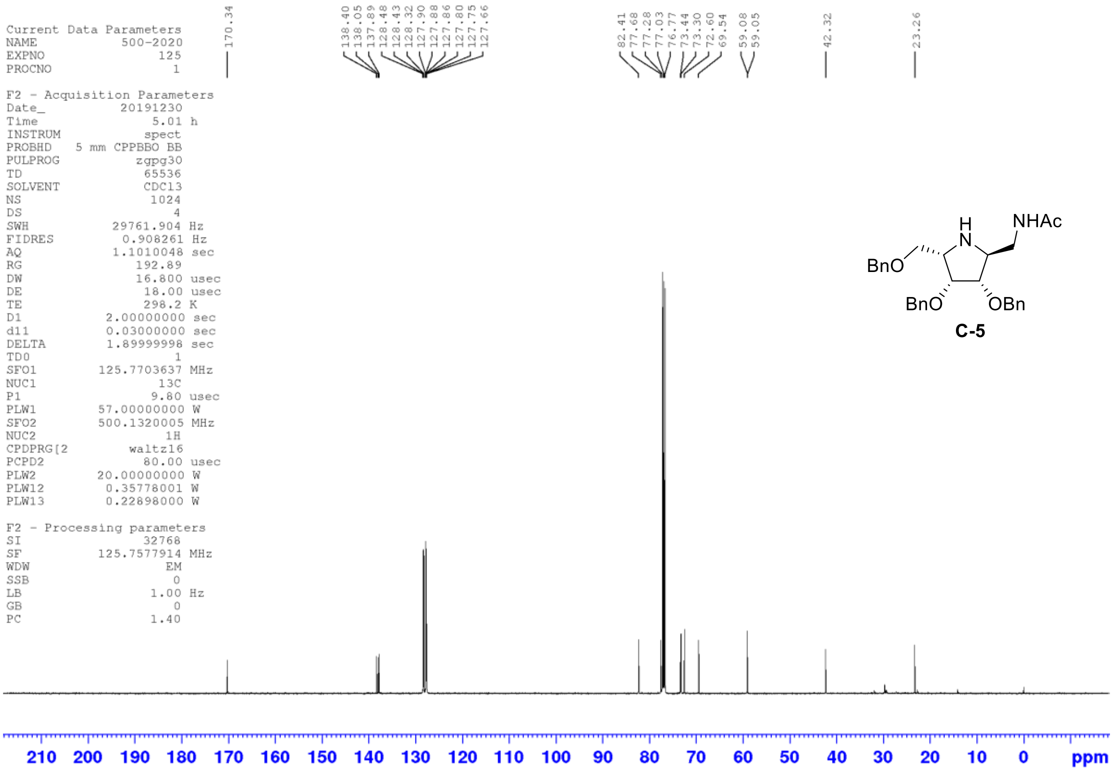
Current Data Parameters
NAME 500-2020
EXPNO 125
PROCNO 1
F2 - Acquisition Parameters
Date_ 20191230
Time 5.01 h
INSTRUM spect
PROBHD 5 mm CPPBBO BB
PULPROG zgpg30
TD 65536
SOLVENT CDCl3
NS 1024
DS 4
SWH 29761.904 Hz
FIDRES 0.908261 Hz
AQ 1.1010048 sec
RG 192.89
DW 16.800 usec
DE 18.00 usec
TE 298.2 K
D1 2.00000000 sec
d11 0.03000000 sec
DELTA 1.89999998 sec
TD0 1
SFO1 125.7703637 MHz
NUC1 13C
P1 9.80 usec
PLW1 57.00000000 W
SFO2 500.1320005 MHz
NUC2 1H
CPDPRG[2 waltz16
PCPD2 80.00 usec
PLW2 20.00000000 W
PLW12 0.35778001 W
PLW13 0.22898000 W
F2 - Processing parameters
SI 32768
SF 125.7577914 MHz
WDW EM
SSB 0
LB 1.00 Hz
GB 0
PC 1.40
170.34
138.40
138.05
137.89
128.48
128.43
128.32
127.90
127.88
127.86
127.80
127.75
127.66
82.41
77.68
77.28
77.03
76.77
73.44
73.30
72.60
69.54
59.08
59.05
42.32
23.26
H
N
NHAc
BnO
BnO
OBn
C-5
210 200 190 180 170 160 150 140 130 120 110 100 90 80 70 60 50 40 30 20 10 0 ppm

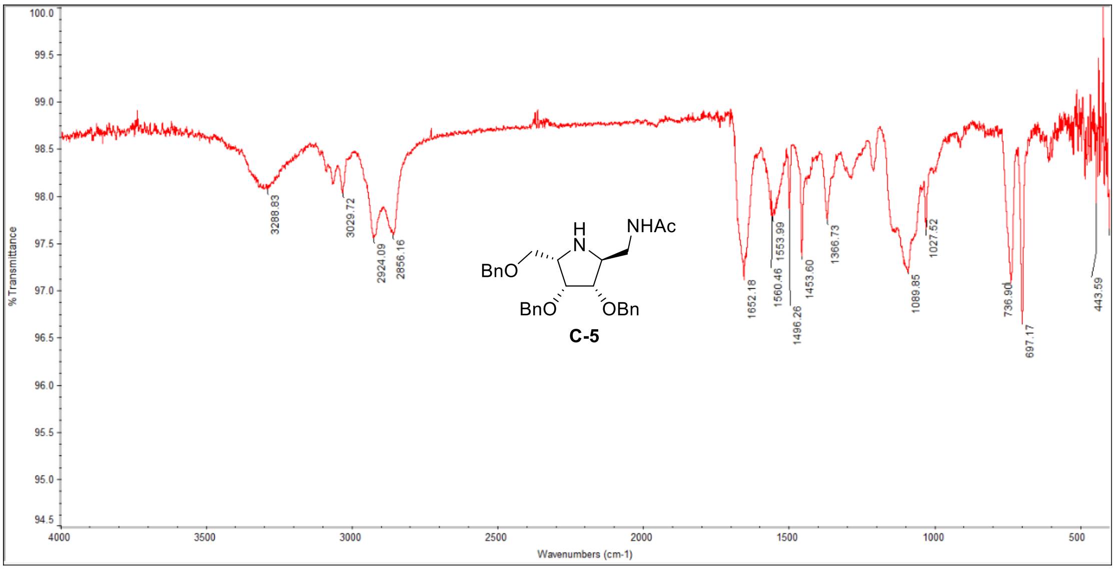
% Transmittance
100.0
99.5
99.0
98.5
98.0
97.5
97.0
96.5
96.0
95.5
95.0
94.5
3288.83
3029.72
2924.09
2856.16
1652.18
1560.46
1553.99
1496.26
1453.60
1366.73
1089.85
1027.52
736.90
697.17
443.59
H
N
NHAc
BnO
BnO
OBn
C-5
4000 3500 3000 2500 2000 1500 1000 500
Wavenumbers (cm-1)

Compound **D-5**:

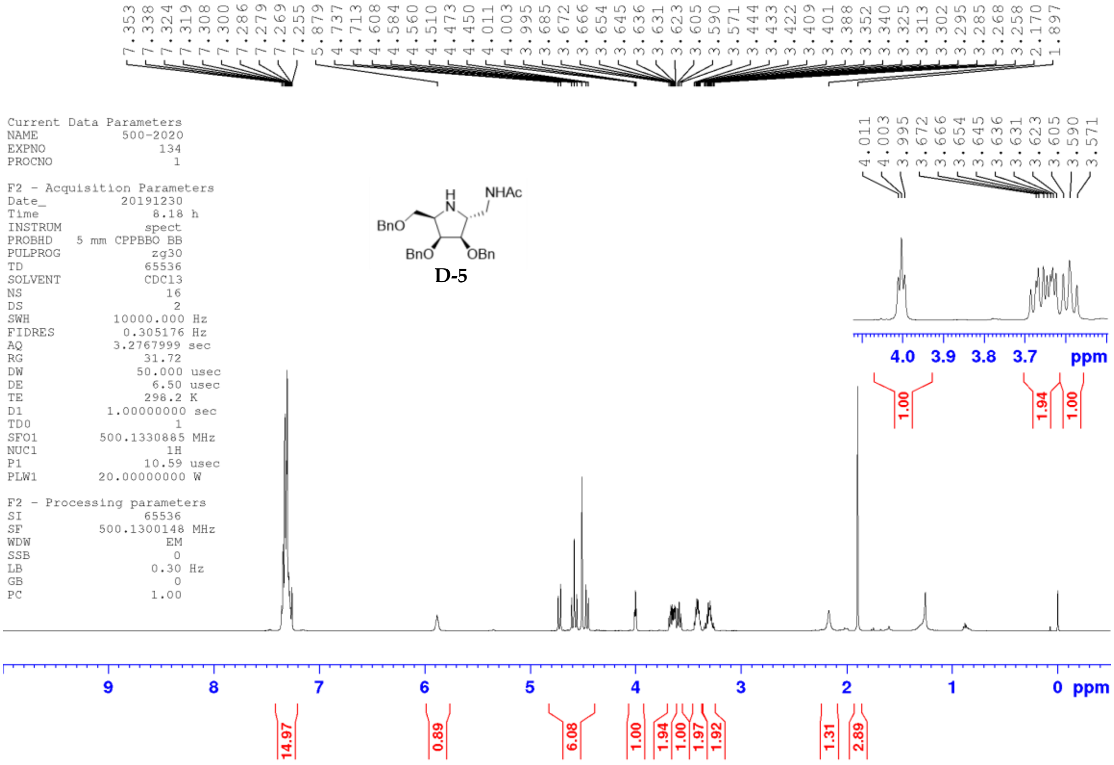

7.353 7.338 7.324 7.319 7.308 7.300 7.286 7.279 7.269 7.255 5.879 4.737 4.713 4.608 4.584 4.560 4.510 4.473 4.450 4.011 4.003 3.995 3.685 3.672 3.666 3.654 3.645 3.636 3.631 3.623 3.605 3.590 3.571 3.444 3.433 3.422 3.409 3.401 3.388 3.352 3.340 3.325 3.313 3.302 3.295 3.285 3.268 3.258 2.170 1.897
4.011 4.003 3.995 3.672 3.666 3.654 3.645 3.636 3.631 3.623 3.605 3.590 3.571
Current Data Parameters
NAME 500-2020
EXPNO 134
PROCNO 1
F2 - Acquisition Parameters
Date_ 20191230
Time 8.18 h
INSTRUM spect
PROBHD 5 mm CPPBBO BB
PULPROG zg30
TD 65536
SOLVENT CDCl3
NS 16
DS 2
SWH 10000.000 Hz
FIDRES 0.305176 Hz
AQ 3.2767999 sec
RG 31.72
DW 50.000 usec
DE 6.50 usec
TE 298.2 K
D1 1.00000000 sec
TD0 1
SFO1 500.1330885 MHz
NUC1 1H
P1 10.59 usec
PLW1 20.00000000 W
F2 - Processing parameters
SI 65536
SF 500.1300148 MHz
WDW EM
SSB 0
LB 0.30 Hz
GB 0
PC 1.00
H
NHAc
BnO
BnO
OBn
D-5
4.0 3.9 3.8 3.7 ppm
1.00 1.94 1.00
9 8 7 6 5 4 3 2 1 0 ppm
14.97 0.89 6.08 1.00 1.94 1.00 1.97 1.92 1.31 2.89

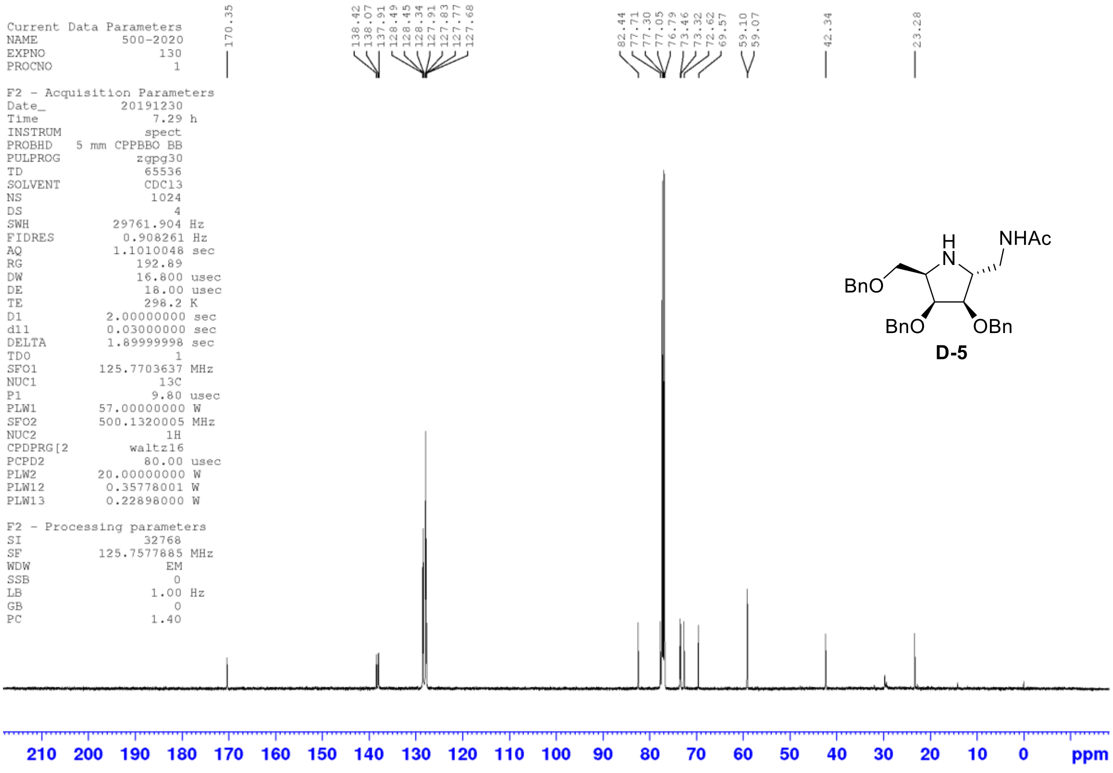

170.35
138.42 138.07 137.91 128.49 128.45 128.34 127.91 127.83 127.77 127.68
82.44 77.71 77.30 77.05 76.79 73.46 73.32 72.62 69.57 59.10 59.07 42.34 23.28
Current Data Parameters
NAME 500-2020
EXPNO 130
PROCNO 1
F2 - Acquisition Parameters
Date_ 20191230
Time 7.29 h
INSTRUM spect
PROBHD 5 mm CPPBBO BB
PULPROG zgpg30
TD 65536
SOLVENT CDCl3
NS 1024
DS 4
SWH 29761.904 Hz
FIDRES 0.908261 Hz
AQ 1.1010048 sec
RG 192.89
DW 16.800 usec
DE 18.00 usec
TE 298.2 K
D1 2.00000000 sec
d11 0.03000000 sec
DELTA 1.89999998 sec
TD0 1
SFO1 125.7703637 MHz
NUC1 13C
P1 9.80 usec
PLW1 57.00000000 W
SFO2 500.1320005 MHz
NUC2 1H
CPDPRG[2 waltz16
PCPD2 80.00 usec
PLW2 20.00000000 W
PLW12 0.35778001 W
PLW13 0.22898000 W
F2 - Processing parameters
SI 32768
SF 125.7577885 MHz
WDW EM
SSB 0
LB 1.00 Hz
GB 0
PC 1.40
H
NHAc
BnO
BnO
OBn
D-5
210 200 190 180 170 160 150 140 130 120 110 100 90 80 70 60 50 40 30 20 10 0 ppm

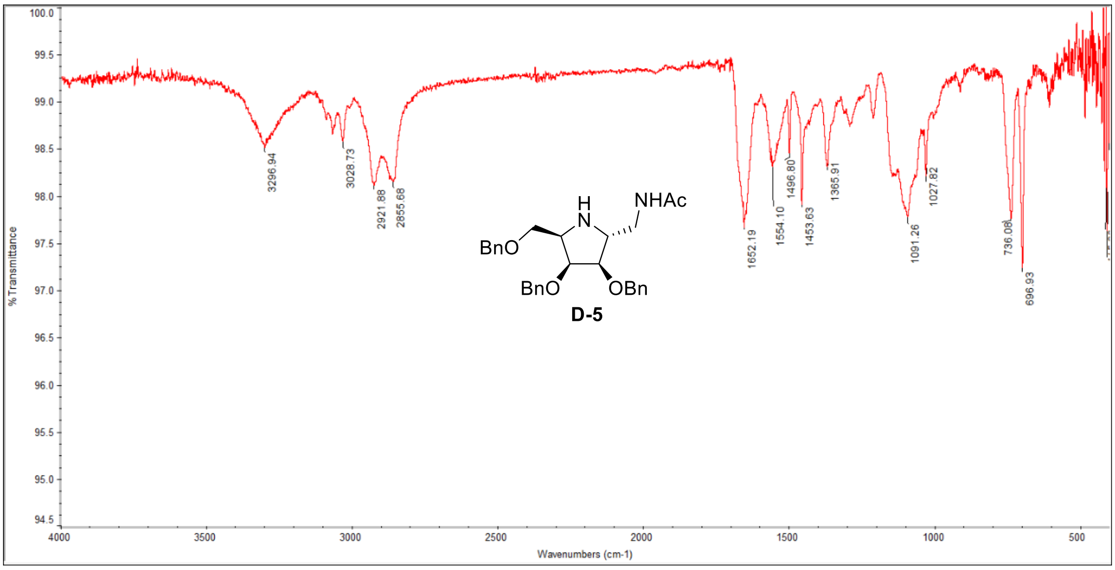

Compound E-5a:

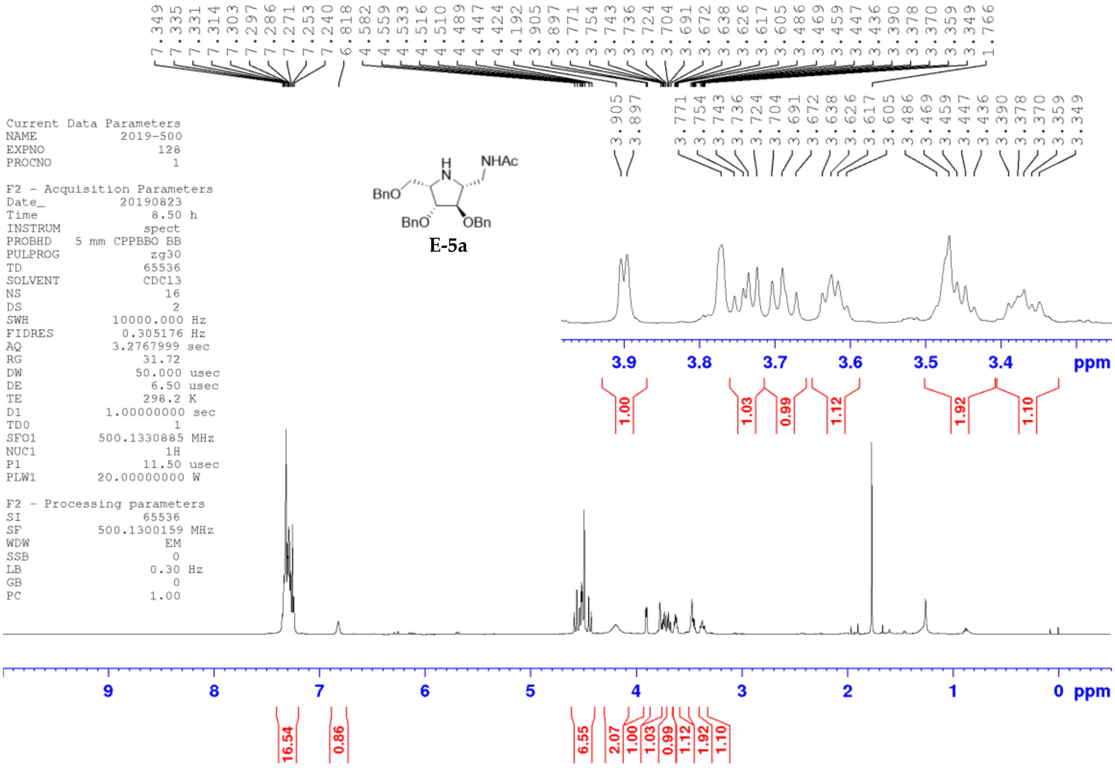

:

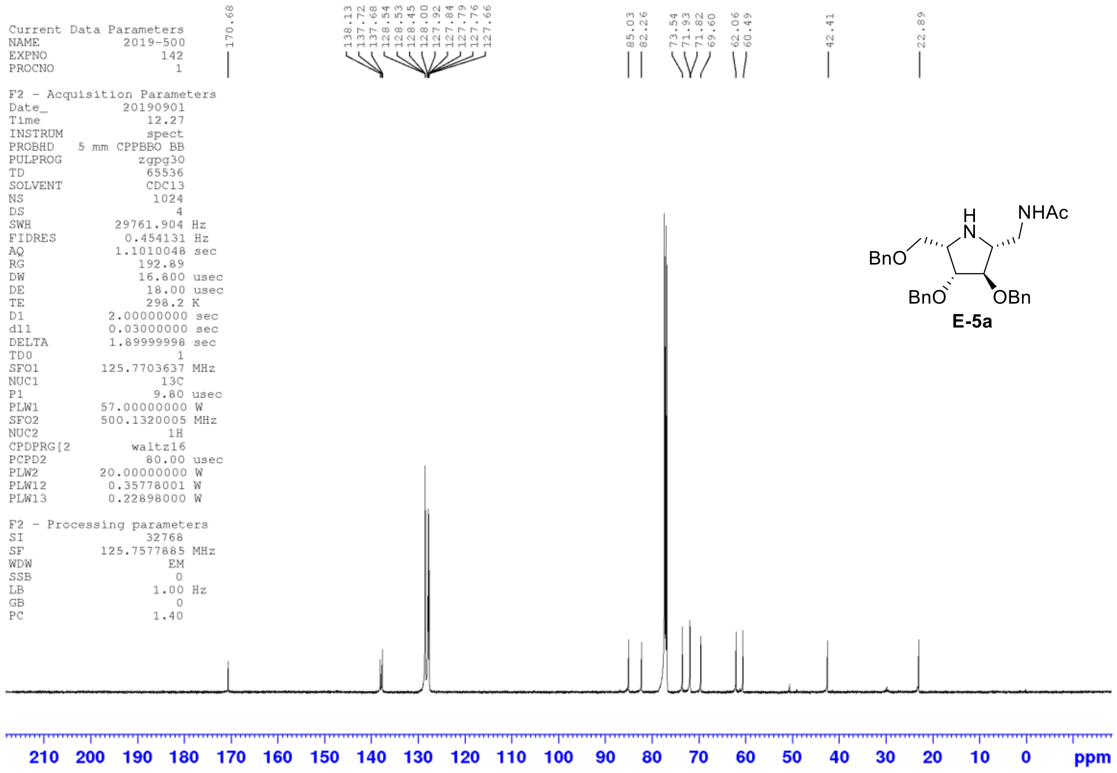

Current Data Parameters
NAME 2019-500
EXPNO 142
PROCNO 1
F2 - Acquisition Parameters
Date_ 20190901
Time 12.27
INSTRUM spect
PROBHD 5 mm CPPBBO BB
PULPROG zgpg30
TD 65536
SOLVENT CDCl3
NS 1024
DS 4
SWH 29761.904 Hz
FIDRES 0.454131 Hz
AQ 1.1010048 sec
RG 192.89
DW 16.800 usec
DE 18.00 usec
TE 298.2 K
D1 2.00000000 sec
d11 0.03000000 sec
DELTA 1.89999998 sec
TD0 1
SFO1 125.7703637 MHz
NUC1 13C
P1 9.80 usec
PLW1 57.00000000 W
SFO2 500.1320005 MHz
NUC2 1H
CPDPRG[2 waltz16
PCPD2 80.00 usec
PLW2 20.00000000 W
PLW12 0.35778001 W
PLW13 0.22898000 W
F2 - Processing parameters
SI 32768
SF 125.7577885 MHz
WDW EM
SSB 0
LB 1.00 Hz
GB 0
PC 1.40
170.68
138.13
137.72
137.68
128.54
128.53
128.45
128.00
127.92
127.84
127.79
127.76
127.66
85.03
82.26
73.54
71.93
71.82
69.60
62.06
60.49
42.41
22.89
H
N
NHAc
BnO
BnO
OBn
E-5a
210 200 190 180 170 160 150 140 130 120 110 100 90 80 70 60 50 40 30 20 10 0 ppm

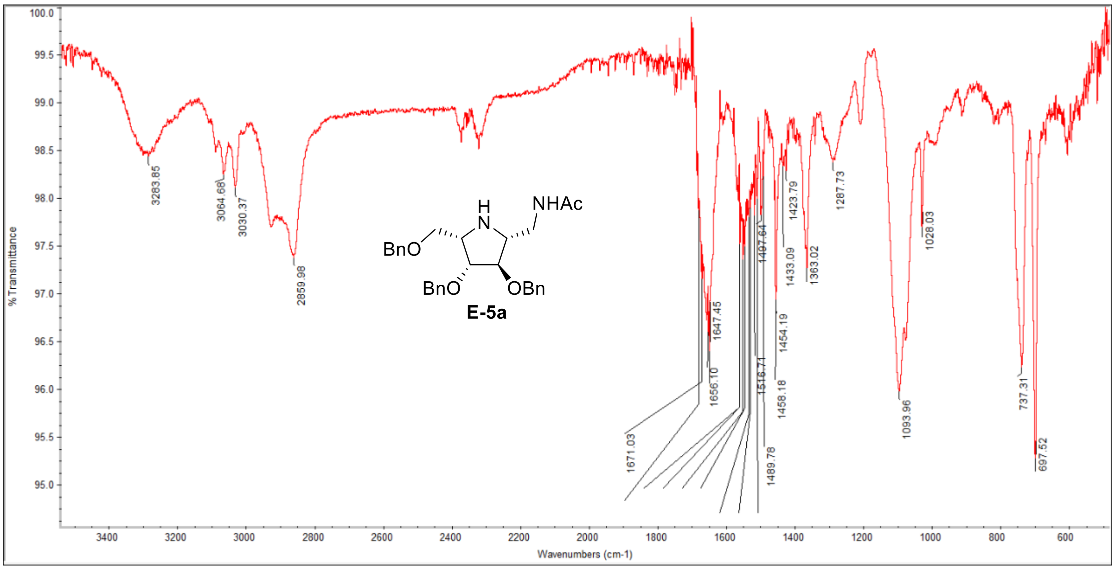

100.0
99.5
99.0
98.5
98.0
97.5
97.0
96.5
96.0
95.5
95.0
%Transmittance
3283.85
3064.68
3030.37
2859.98
1671.03
1656.10
1647.45
1516.71
1497.64
1489.78
1458.18
1454.19
1433.09
1423.79
1363.02
1287.73
1093.96
1028.03
737.31
697.52
H
N
NHAc
BnO
BnO
OBn
E-5a
3400 3200 3000 2800 2600 2400 2200 2000 1800 1600 1400 1200 1000 800 600
Wavenumbers (cm-1)

Compound **E-5b**:

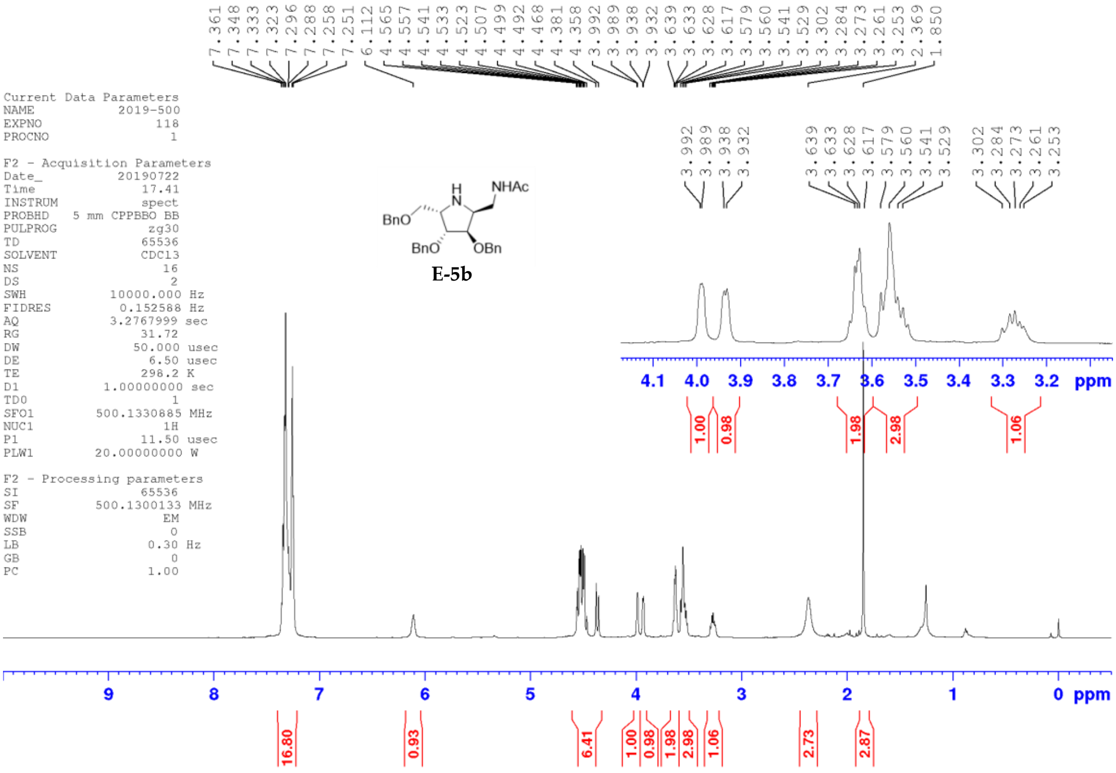

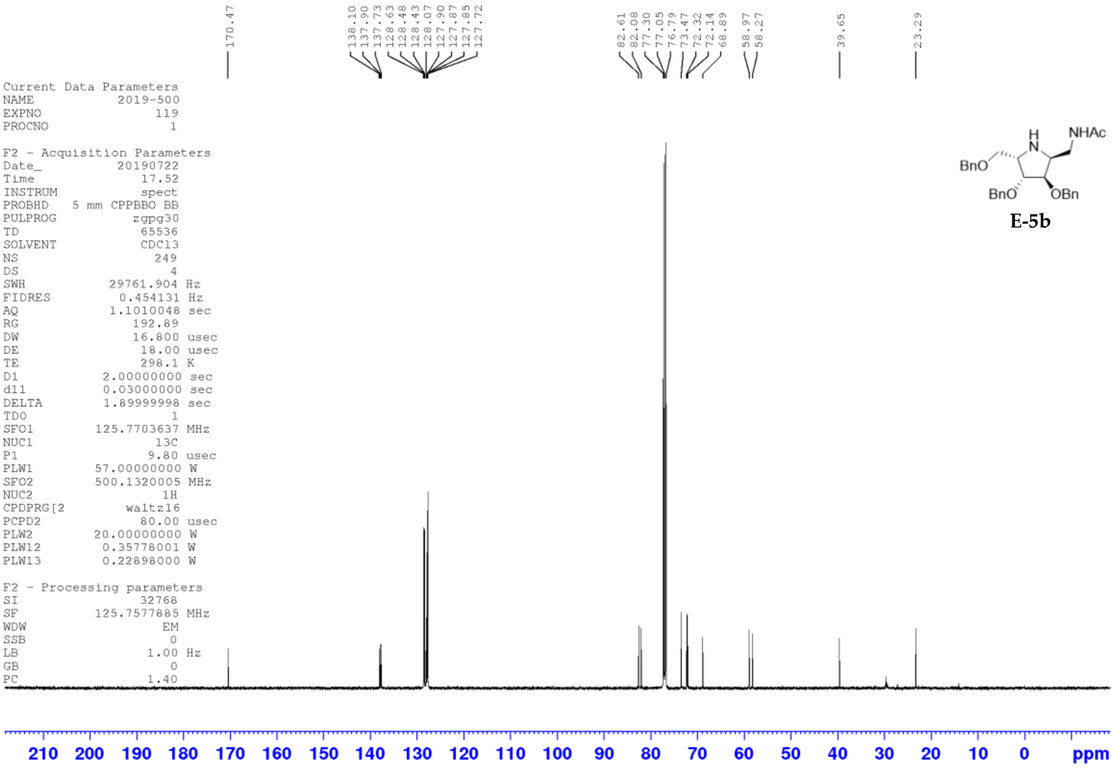

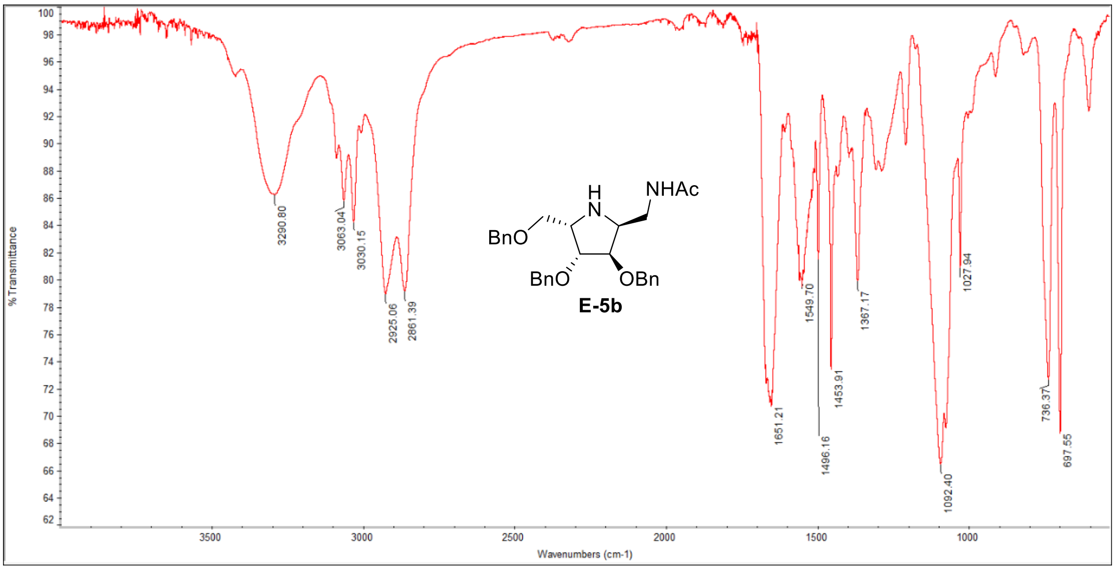

Compound **F-5a**:

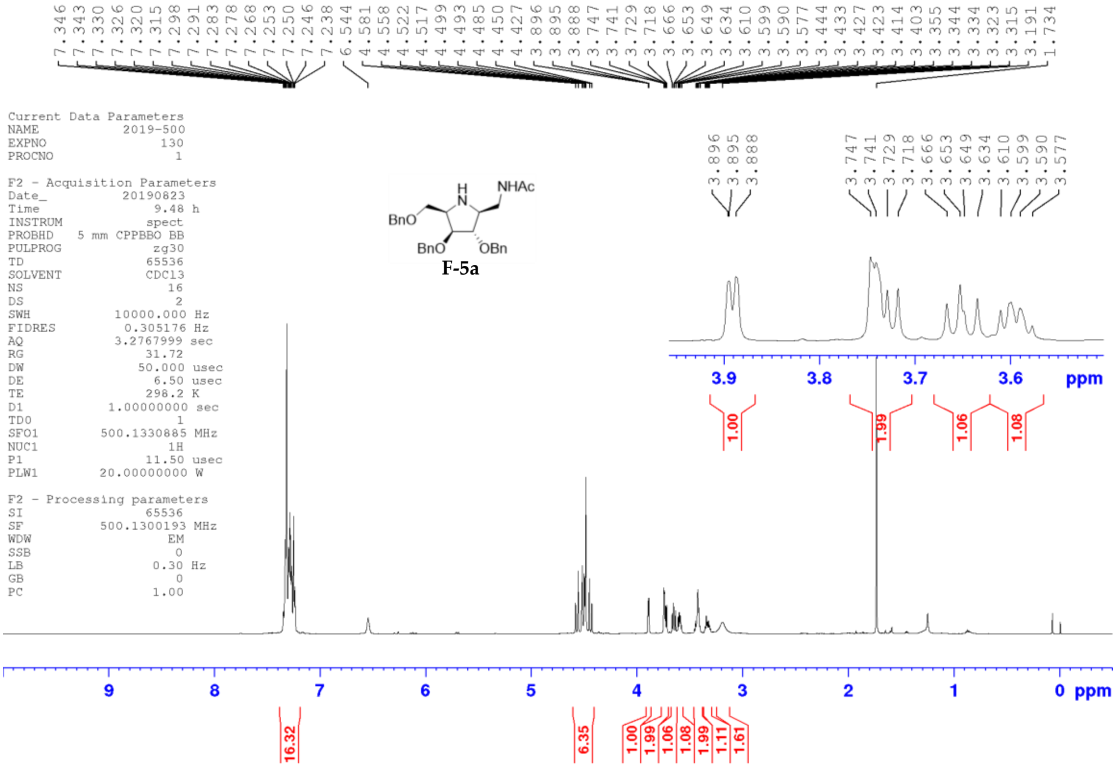

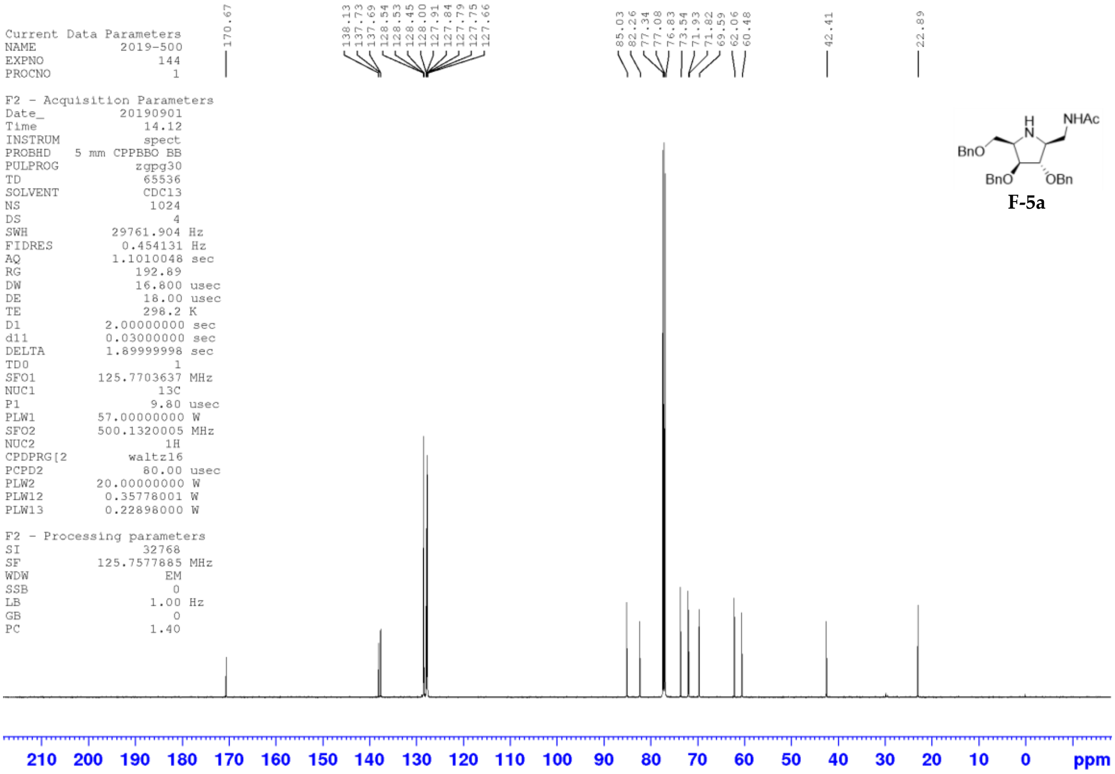
Current Data Parameters
NAME 2019-500
EXPNO 144
PROCNO 1
F2 - Acquisition Parameters
Date_ 20190901
Time 14.12
INSTRUM spect
PROBHD 5 mm CPPBBO BB
PULPROG zgpg30
TD 65536
SOLVENT CDCl3
NS 1024
DS 4
SWH 29761.904 Hz
FIDRES 0.454131 Hz
AQ 1.1010048 sec
RG 192.89
DW 16.800 usec
DE 18.00 usec
TE 298.2 K
D1 2.00000000 sec
d11 0.03000000 sec
DELTA 1.89999998 sec
TD0 1
SFO1 125.7703637 MHz
NUC1 13C
P1 9.80 usec
PLW1 57.00000000 W
SFO2 500.1320005 MHz
NUC2 1H
CPDPRG[2 waltz16
PCPD2 80.00 usec
PLW2 20.00000000 W
PLW12 0.35778001 W
PLW13 0.22898000 W
F2 - Processing parameters
SI 32768
SF 125.7577885 MHz
WDW EM
SSB 0
LB 1.00 Hz
GB 0
PC 1.40
170.67
138.13
137.73
137.69
128.54
128.53
128.45
128.00
127.91
127.84
127.79
127.75
127.66
85.03
82.26
77.34
77.08
76.83
73.54
71.93
71.82
69.59
62.06
60.48
42.41
22.89
H
N
NHAc
BnO
BnO
OBn
F-5a
210 200 190 180 170 160 150 140 130 120 110 100 90 80 70 60 50 40 30 20 10 0 ppm

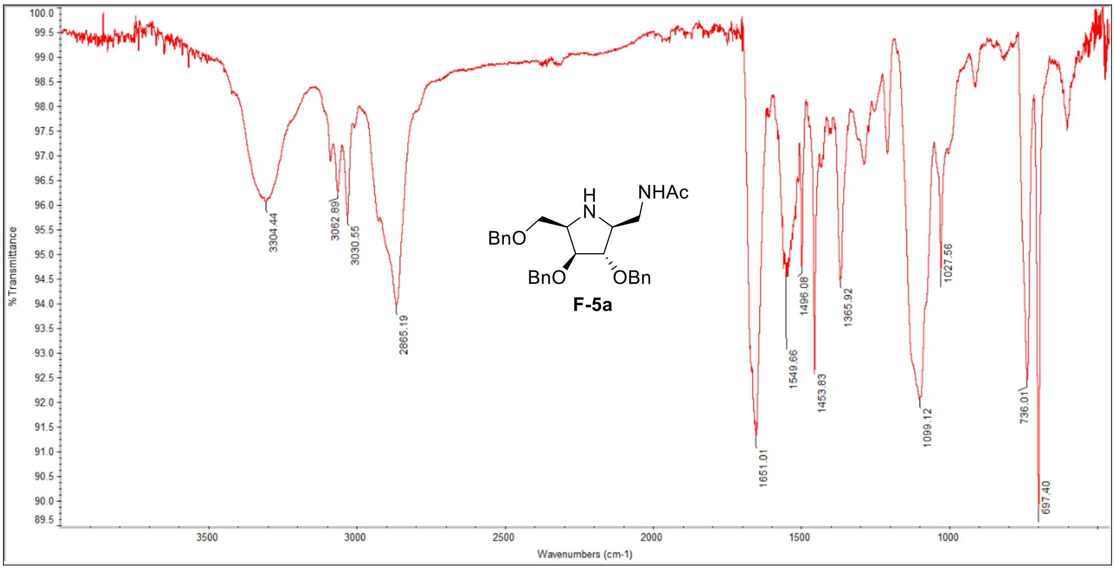
% Transmittance
100.0
99.5
99.0
98.5
98.0
97.5
97.0
96.5
96.0
95.5
95.0
94.5
94.0
93.5
93.0
92.5
92.0
91.5
91.0
90.5
90.0
89.5
3304.44
3062.89
3030.55
2865.19
1651.01
1549.66
1496.08
1453.83
1365.92
1099.12
1027.56
736.01
697.40
H
N
NHAc
BnO
BnO
OBn
F-5a
3500
3000
2500
2000
1500
1000
Wavenumbers (cm-1)

Compound **F-5b**:

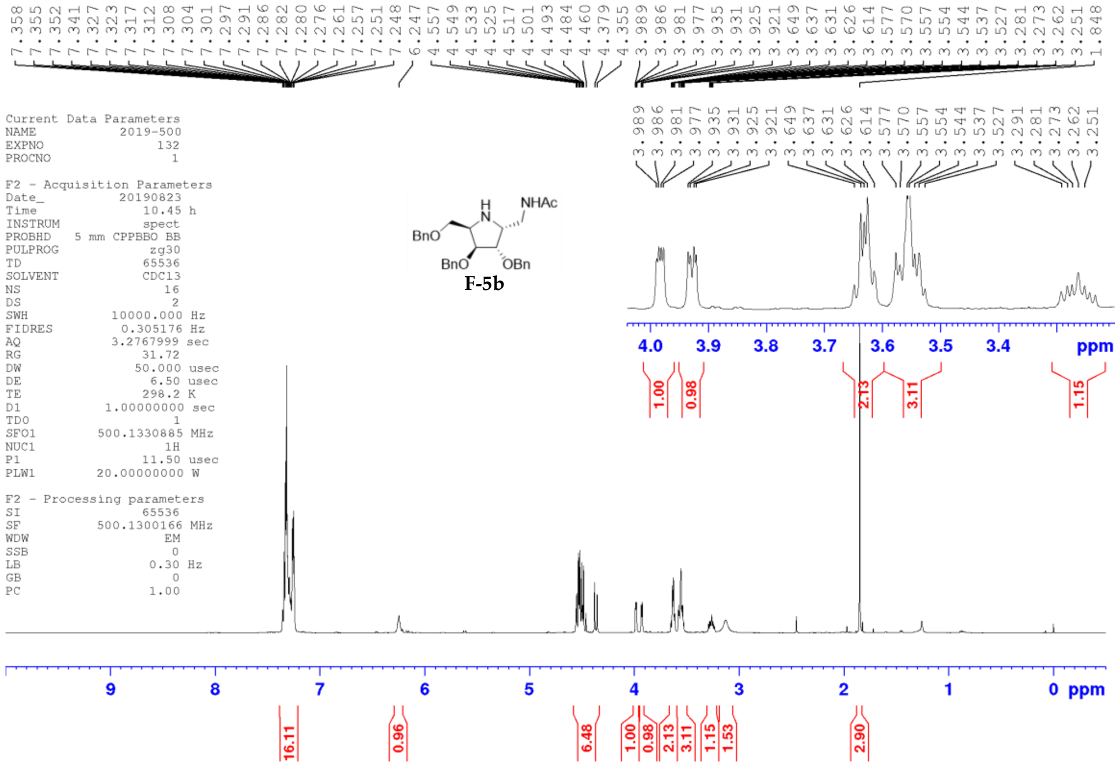

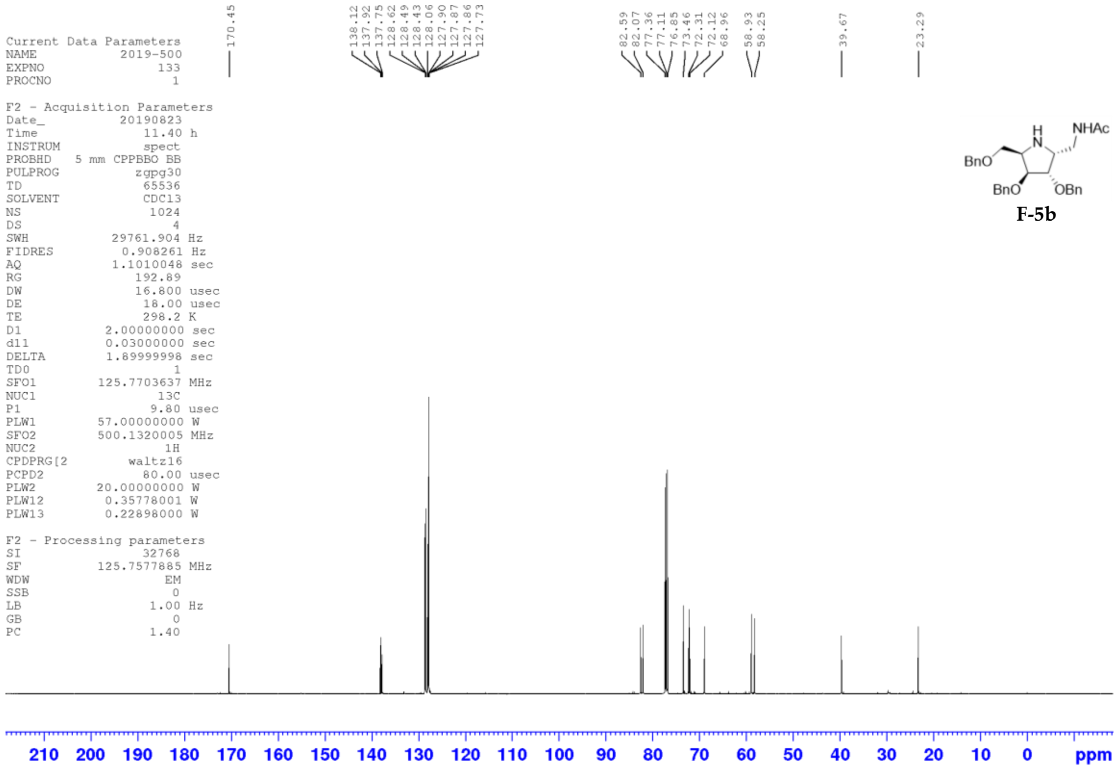

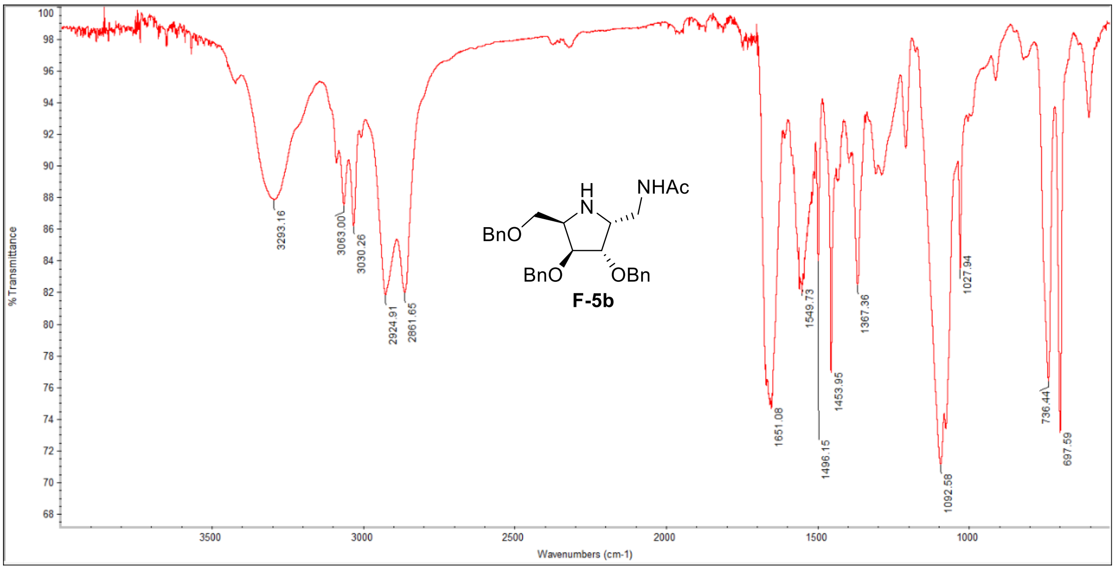

% Transmittance
Wavenumbers (cm-1)
3293.16
3063.00
3030.26
2924.91
2861.65
1651.08
1549.73
1496.15
1453.95
1367.36
1092.58
1027.94
736.44
697.59
NHAc
BnO
BnO
OBn
F-5b

Compound **G-5**:

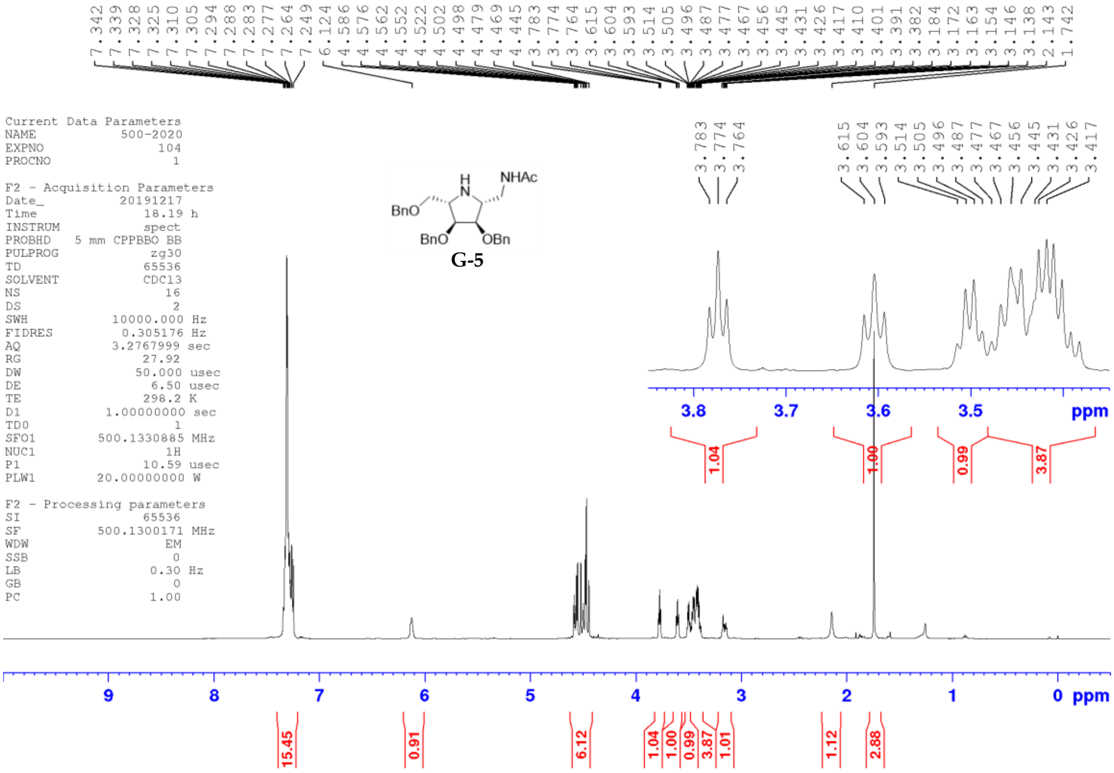

Current Data Parameters
NAME 500-2020
EXPNO 104
PROCNO 1
F2 - Acquisition Parameters
Date_ 20191217
Time 18.19 h
INSTRUM spect
PROBHD 5 mm CPPBBO BB
PULPROG zg30
TD 65536
SOLVENT CDCl3
NS 16
DS 2
SWH 10000.000 Hz
FIDRES 0.305176 Hz
AQ 3.2767999 sec
RG 27.92
DW 50.000 usec
DE 6.50 usec
TE 298.2 K
D1 1.00000000 sec
TD0 1
SFO1 500.1330885 MHz
NUC1 1H
P1 10.59 usec
PLW1 20.00000000 W
F2 - Processing parameters
SI 65536
SF 500.1300171 MHz
WDW EM
SSB 0
LB 0.30 Hz
GB 0
PC 1.00
NHAc
BnO
BnO
OBn
G-5
ppm

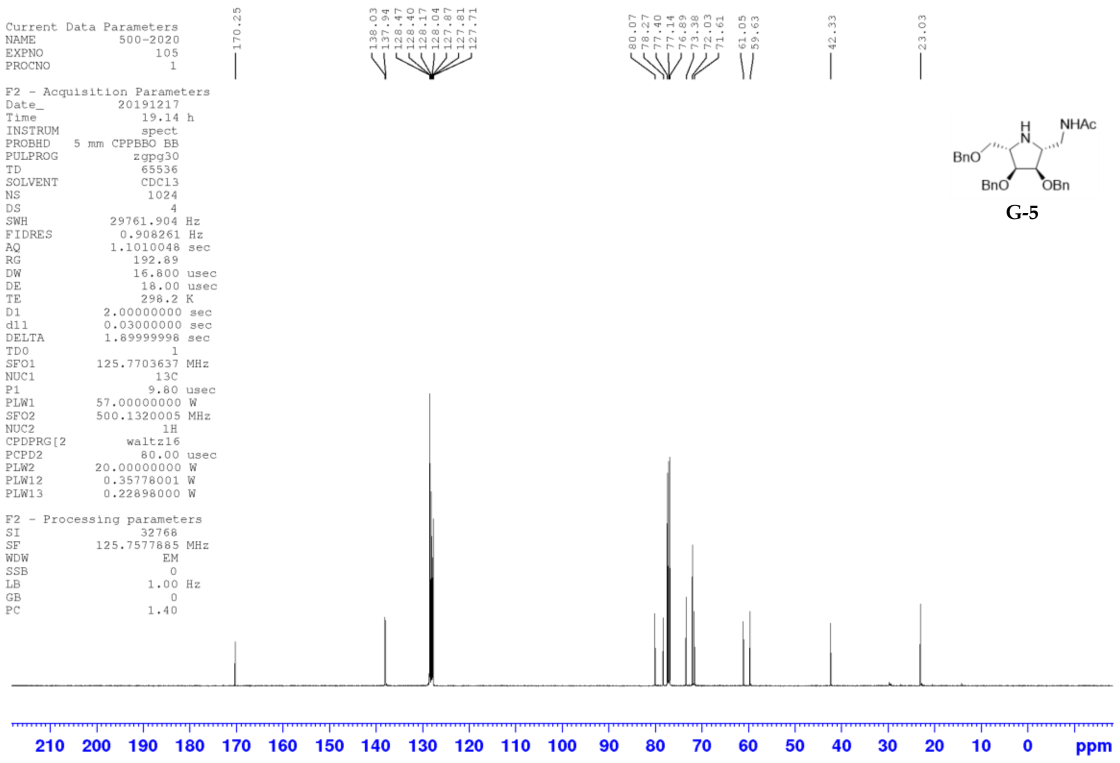
Current Data Parameters
NAME 500-2020
EXPNO 105
PROCNO 1
F2 - Acquisition Parameters
Date_ 20191217
Time 19.14 h
INSTRUM spect
PROBHD 5 mm CPPBBO BB
PULPROG zgpg30
TD 65536
SOLVENT CDCl3
NS 1024
DS 4
SWH 29761.904 Hz
FIDRES 0.908261 Hz
AQ 1.1010048 sec
RG 192.89
DW 16.800 usec
DE 18.00 usec
TE 298.2 K
D1 2.00000000 sec
d11 0.03000000 sec
DELTA 1.89999998 sec
TD0 1
SFO1 125.7703637 MHz
NUC1 13C
P1 9.80 usec
PLW1 57.00000000 W
SFO2 500.1320005 MHz
NUC2 1H
CPDPRG[2 waltz16
PCPD2 80.00 usec
PLW2 20.00000000 W
PLW12 0.35778001 W
PLW13 0.22898000 W
F2 - Processing parameters
SI 32768
SF 125.7577685 MHz
WDW EM
SSB 0
LB 1.00 Hz
GB 0
PC 1.40
170.25
138.03
137.94
128.47
128.40
128.17
128.04
127.87
127.81
127.71
80.07
78.27
77.40
77.14
76.89
73.38
72.03
71.61
61.05
59.63
42.33
23.03
NHAc
H
N
BnO
BnO
OBn
G-5
210 200 190 180 170 160 150 140 130 120 110 100 90 80 70 60 50 40 30 20 10 0 ppm

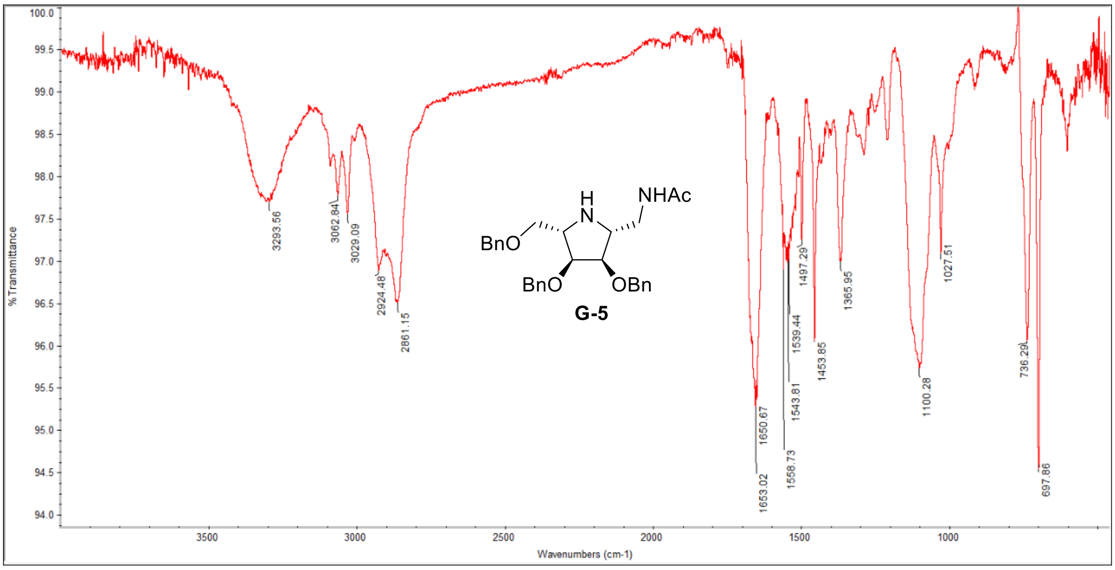
% Transmittance
100.0
99.5
99.0
98.5
98.0
97.5
97.0
96.5
96.0
95.5
95.0
94.5
94.0
3293.56
3062.84
3029.09
2924.48
2861.15
1653.02
1650.67
1558.73
1543.81
1539.44
1497.29
1453.85
1365.95
1100.28
1027.51
736.29
697.86
NHAc
H
N
BnO
BnO
OBn
G-5
3500
3000
2500
2000
1500
1000
Wavenumbers (cm-1)

Compound **H-5**:

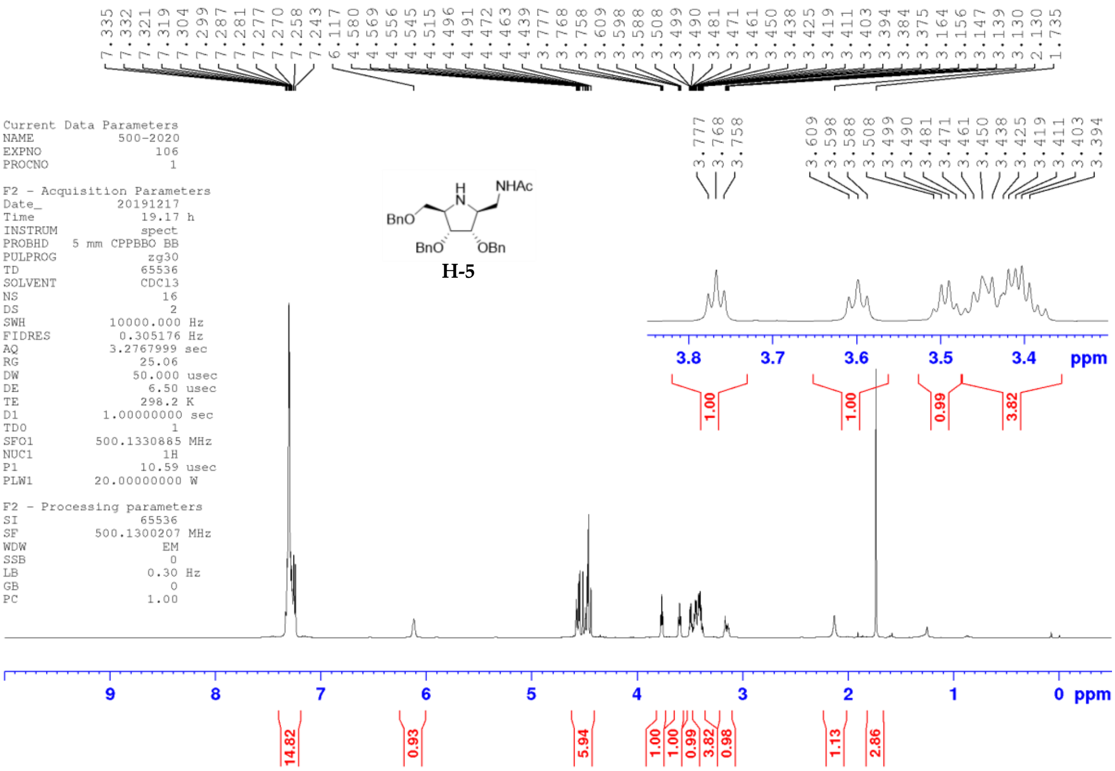

7.335 7.332 7.321 7.319 7.304 7.299 7.287 7.281 7.277 7.270 7.258 7.243 6.117 4.580 4.569 4.556 4.545 4.515 4.496 4.491 4.472 4.463 4.439 3.777 3.768 3.758 3.609 3.598 3.588 3.508 3.499 3.490 3.481 3.471 3.461 3.450 3.438 3.425 3.419 3.411 3.403 3.394 3.384 3.375 3.164 3.156 3.147 3.139 3.130 2.130 1.735
Current Data Parameters
NAME 500-2020
EXPNO 106
PROCNO 1
F2 - Acquisition Parameters
Date_ 20191217
Time 19.17 h
INSTRUM spect
PROBHD 5 mm CPPBBO BB
PULPROG zg30
TD 65536
SOLVENT CDCl3
NS 16
DS 2
SWH 10000.000 Hz
FIDRES 0.305176 Hz
AQ 3.2767999 sec
RG 25.06
DW 50.000 usec
DE 6.50 usec
TE 298.2 K
D1 1.00000000 sec
TD0 1
SFO1 500.1330885 MHz
NUC1 1H
P1 10.59 usec
PLW1 20.00000000 W
F2 - Processing parameters
SI 65536
SF 500.1300207 MHz
WDW EM
SSB 0
LB 0.30 Hz
GB 0
PC 1.00
NHAc
H
N
BnO
BnO
OBn
H-5
3.777 3.768 3.758
3.609 3.598 3.588 3.508 3.499 3.490 3.481 3.471 3.461 3.450 3.438 3.425 3.419 3.411 3.403 3.394
3.8 3.7 3.6 3.5 3.4 ppm
1.00 1.00 0.99 3.82
9 8 7 6 5 4 3 2 1 0 ppm
14.82 0.93 5.94 1.00 1.00 0.99 3.82 0.98 1.13 2.86

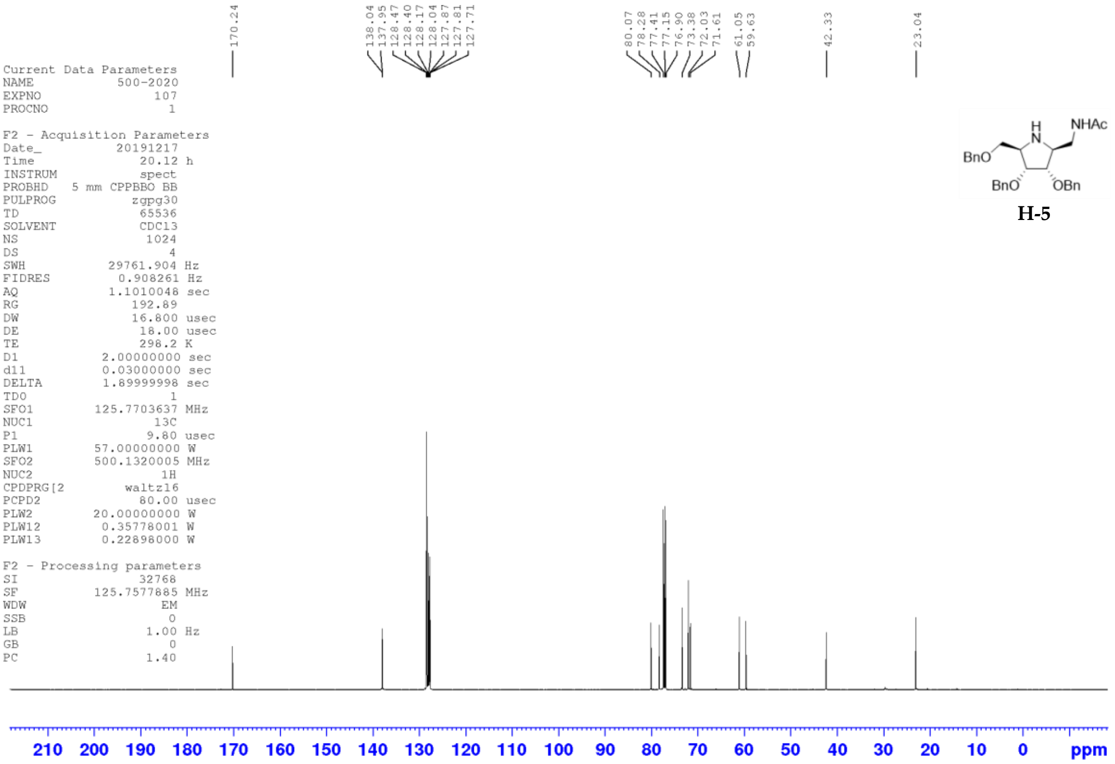

170.24
138.04 137.95 128.47 128.40 128.17 128.04 127.87 127.81 127.71
80.07 78.28 77.41 77.15 76.90 73.38 72.03 71.61 61.05 59.63
42.33
23.04
Current Data Parameters
NAME 500-2020
EXPNO 107
PROCNO 1
F2 - Acquisition Parameters
Date_ 20191217
Time 20.12 h
INSTRUM spect
PROBHD 5 mm CPPBBO BB
PULPROG zgpg30
TD 65536
SOLVENT CDCl3
NS 1024
DS 4
SWH 29761.904 Hz
FIDRES 0.908261 Hz
AQ 1.1010048 sec
RG 192.89
DW 16.800 usec
DE 18.00 usec
TE 298.2 K
D1 2.00000000 sec
d11 0.03000000 sec
DELTA 1.89999998 sec
TD0 1
SFO1 125.7703637 MHz
NUC1 13C
P1 9.80 usec
PLW1 57.00000000 W
SFO2 500.1320005 MHz
NUC2 1H
CPDPRG[2 waltz16
PCPD2 80.00 usec
PLW2 20.00000000 W
PLW12 0.35778001 W
PLW13 0.22898000 W
F2 - Processing parameters
SI 32768
SF 125.7577885 MHz
WDW EM
SSB 0
LB 1.00 Hz
GB 0
PC 1.40
NHAc
H
N
BnO
BnO
OBn
H-5
210 200 190 180 170 160 150 140 130 120 110 100 90 80 70 60 50 40 30 20 10 0 ppm

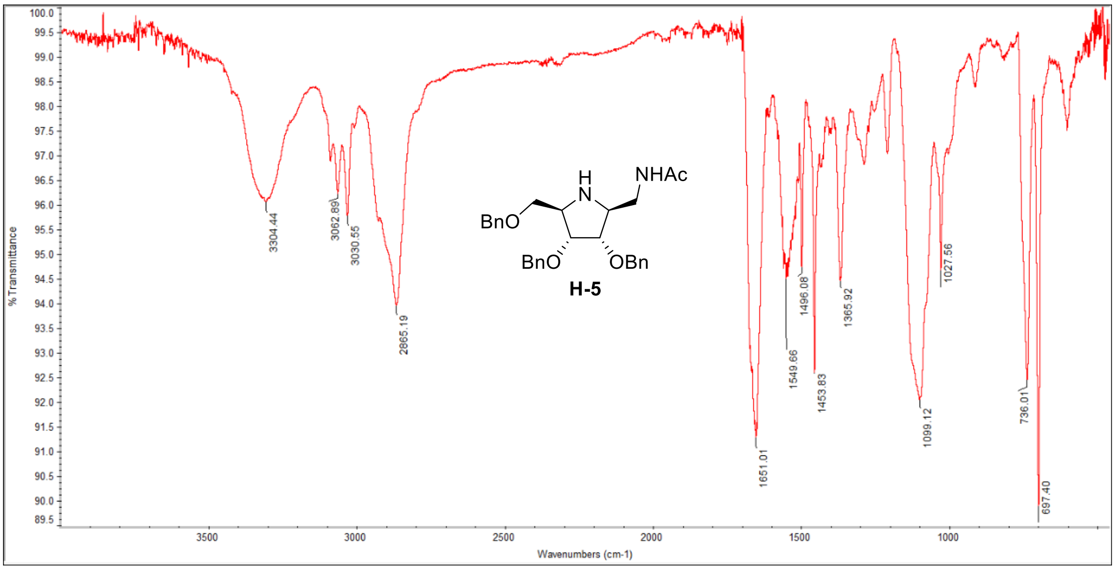

Compound **A-10**:

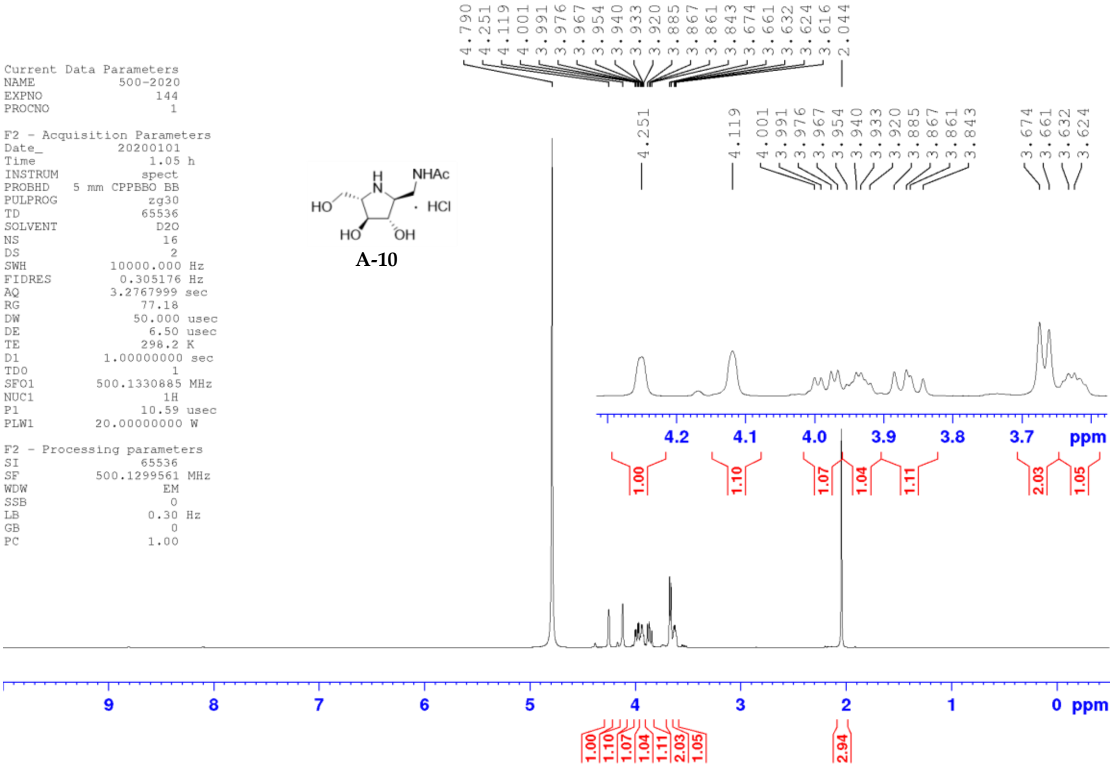

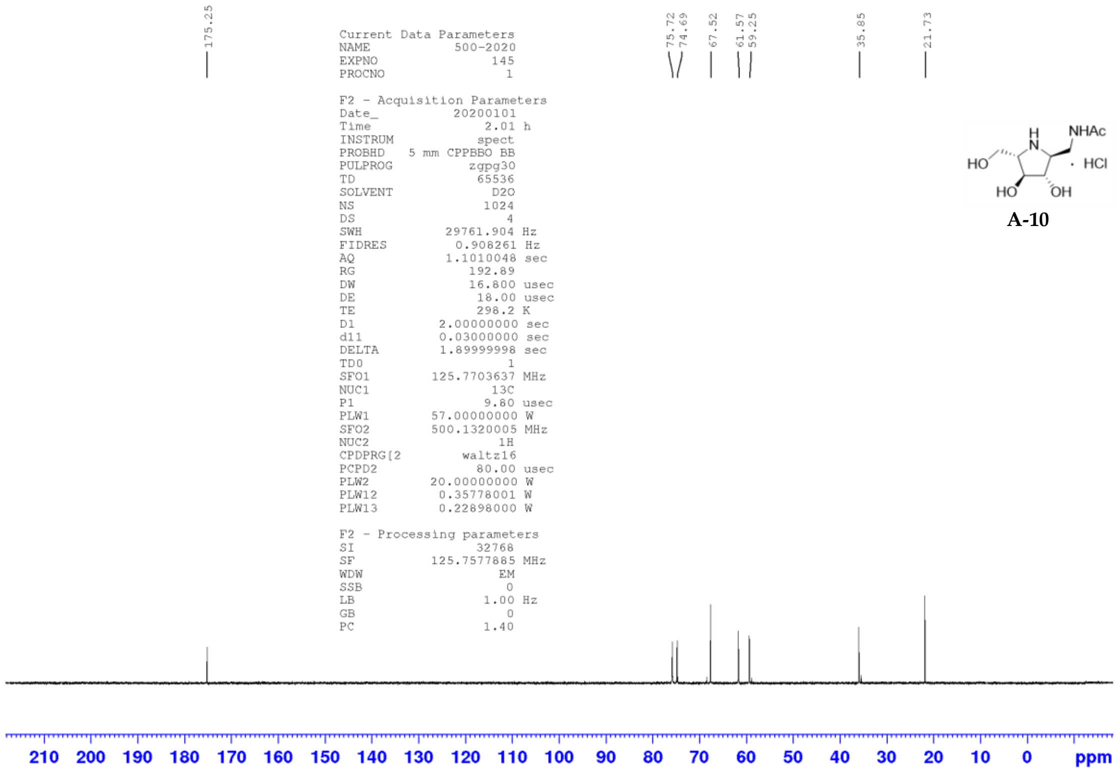
175.25
75.72
74.69
67.52
61.57
59.25
35.85
21.73
Current Data Parameters
NAME 500-2020
EXPNO 145
PROCNO 1
F2 - Acquisition Parameters
Date_ 20200101
Time 2.01 h
INSTRUM spect
PROBHD 5 mm CPPBBO BB
PULPROG zgpg30
TD 65536
SOLVENT D2O
NS 1024
DS 4
SWH 29761.904 Hz
FIDRES 0.908261 Hz
AQ 1.1010048 sec
RG 192.89
DW 16.800 usec
DE 18.00 usec
TE 298.2 K
D1 2.00000000 sec
d11 0.03000000 sec
DELTA 1.89999998 sec
TD0 1
SFO1 125.7703637 MHz
NUC1 13C
P1 9.80 usec
PLW1 57.00000000 W
SFO2 500.1320005 MHz
NUC2 1H
CPDPRG[2 waltz16
PCPD2 80.00 usec
PLW2 20.00000000 W
PLW12 0.35778001 W
PLW13 0.22898000 W
F2 - Processing parameters
SI 32768
SF 125.7577885 MHz
WDW EM
SSB 0
LB 1.00 Hz
GB 0
PC 1.40
NHAc
HO
HO
OH
HCl
A-10
210 200 190 180 170 160 150 140 130 120 110 100 90 80 70 60 50 40 30 20 10 0 ppm

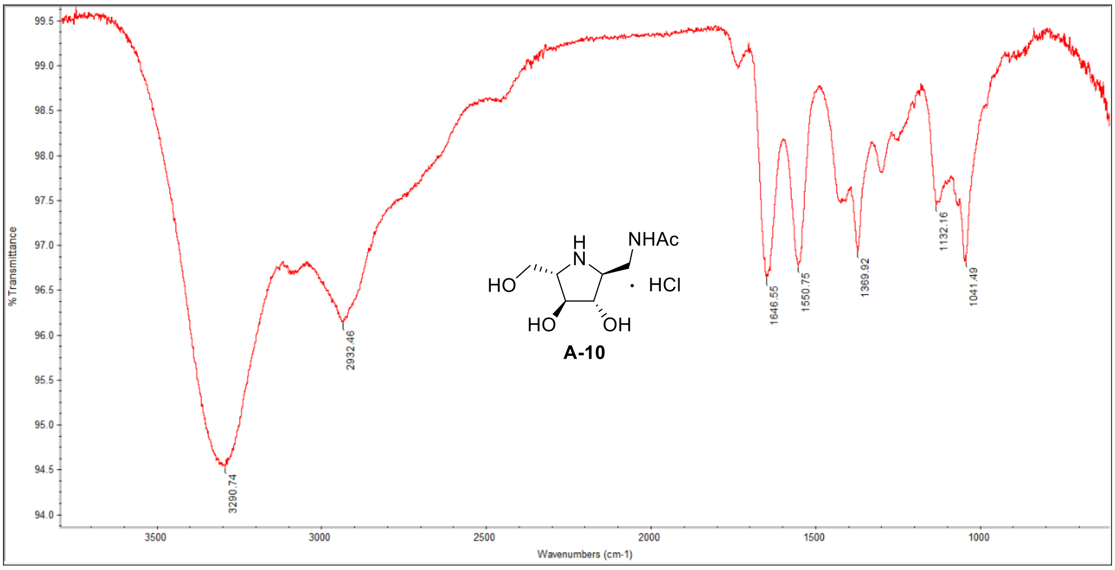
% Transmittance
99.5
99.0
98.5
98.0
97.5
97.0
96.5
96.0
95.5
95.0
94.5
94.0
3290.74
2932.46
1646.55
1550.75
1369.92
1132.16
1041.49
NHAc
HO
HO
OH
HCl
A-10
3500
3000
2500
2000
1500
1000
Wavenumbers (cm-1)

Compound **4-HCl**:

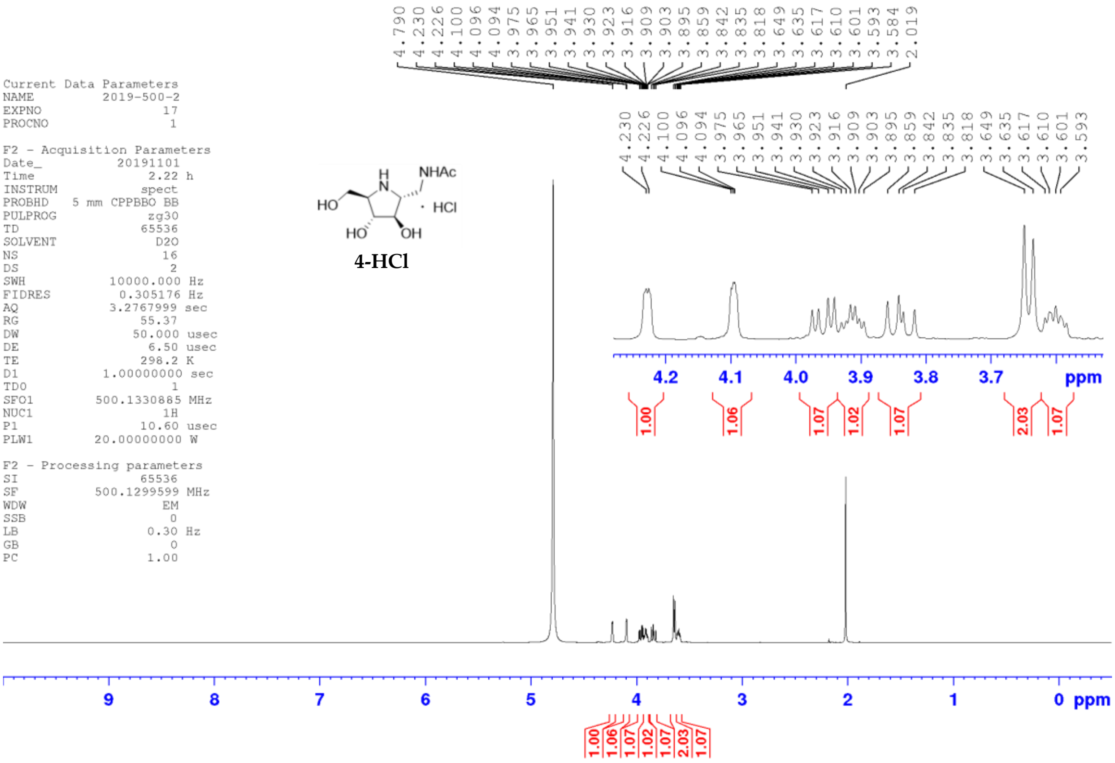

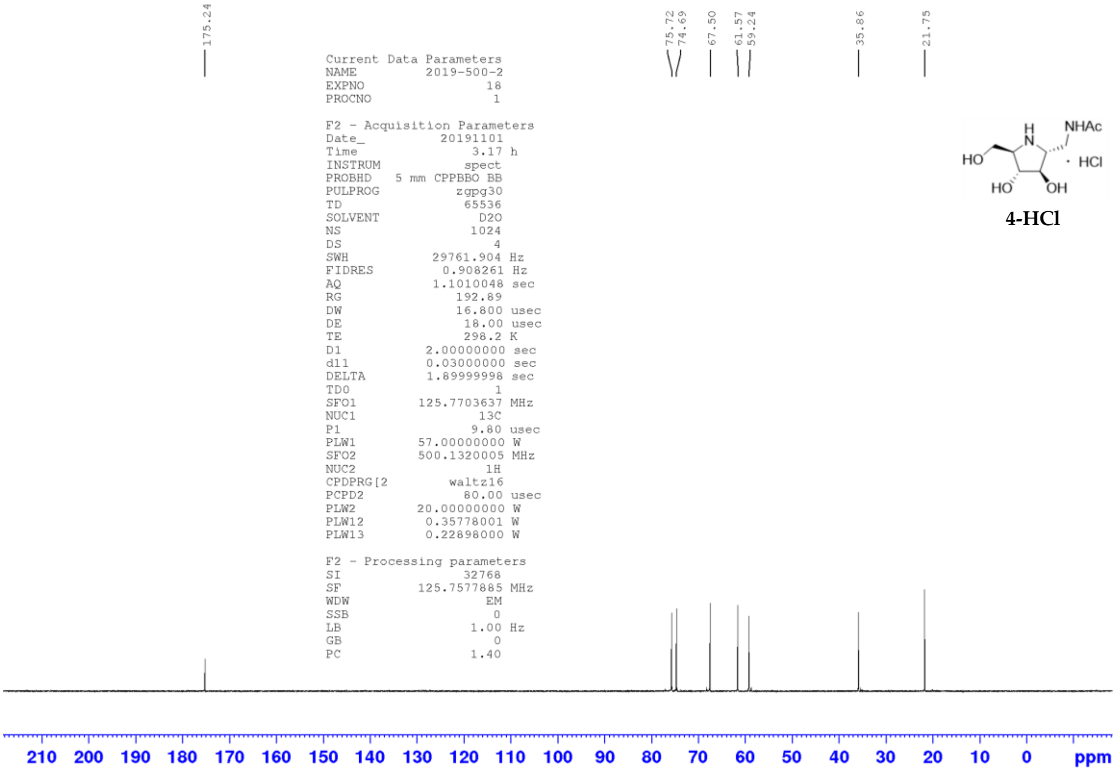

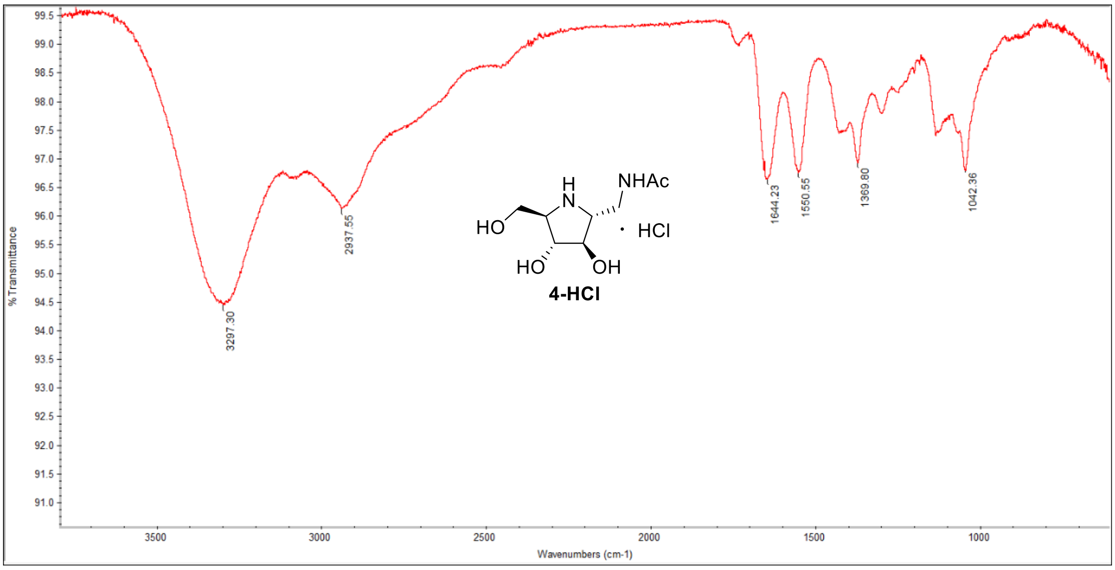

Compound **C-10**:

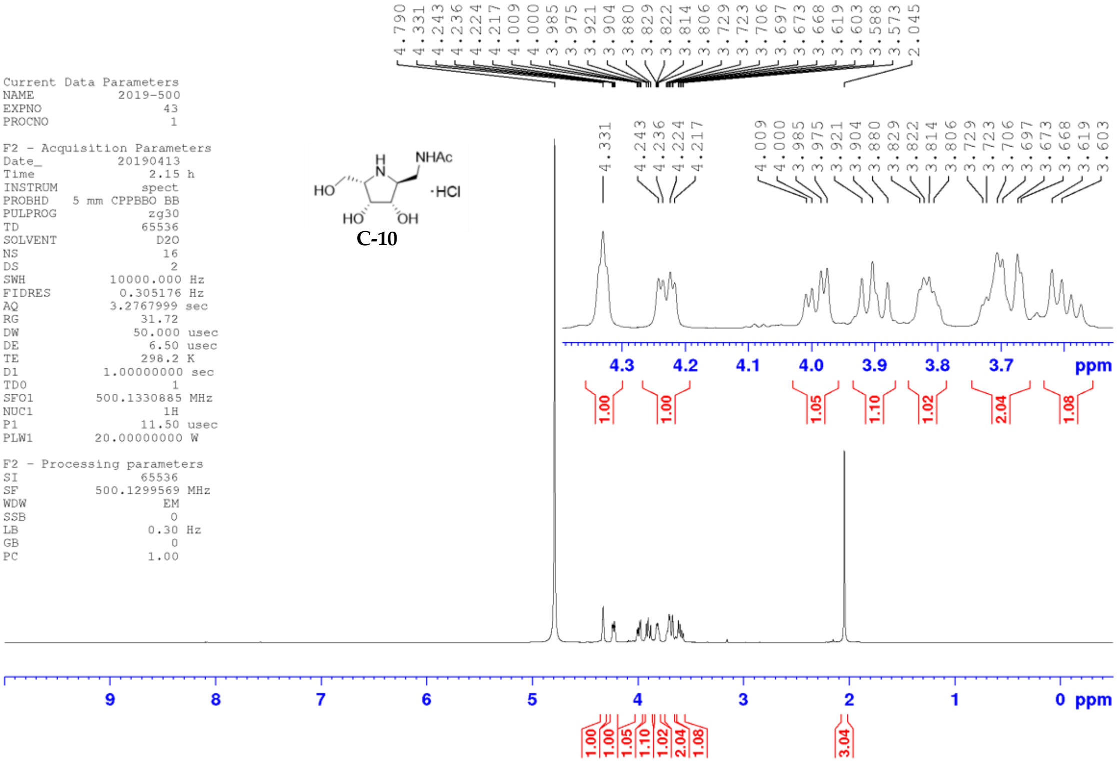

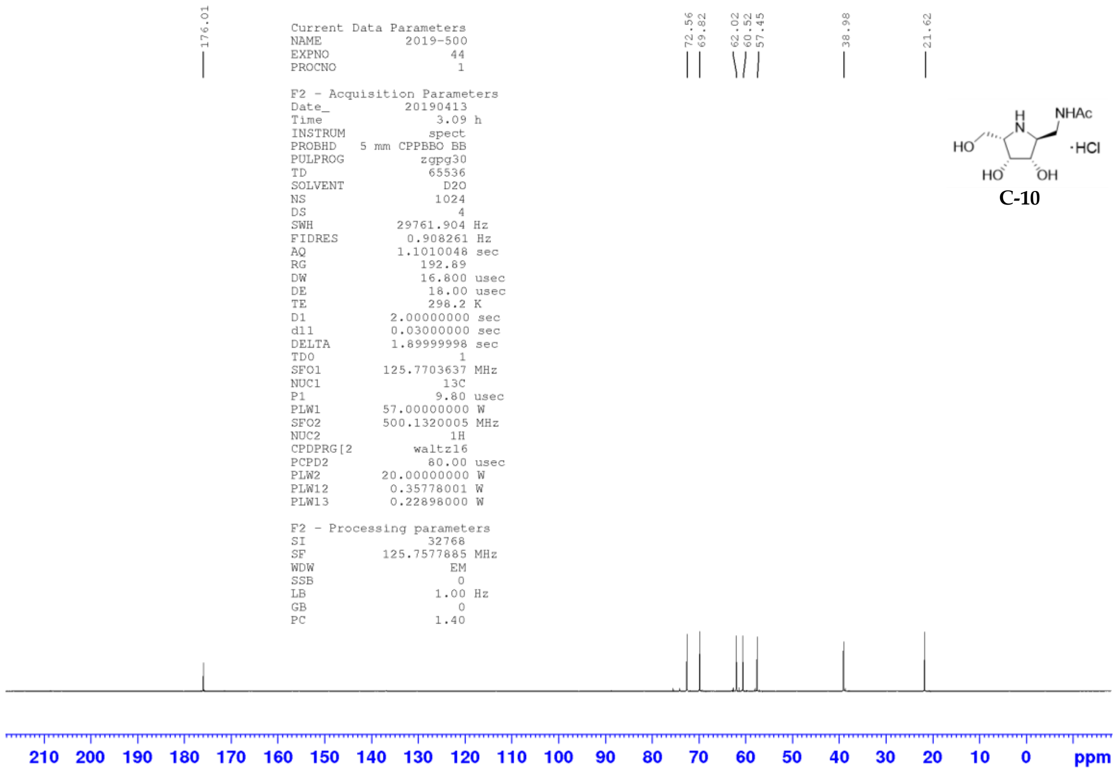
176.01
72.56
69.82
62.02
60.52
57.45
38.98
21.62
Current Data Parameters
NAME 2019-500
EXPNO 44
PROCNO 1
F2 - Acquisition Parameters
Date_ 20190413
Time 3.09 h
INSTRUM spect
PROBHD 5 mm CPPBBO BB
PULPROG zgpg30
TD 65536
SOLVENT D2O
NS 1024
DS 4
SWH 29761.904 Hz
FIDRES 0.908261 Hz
AQ 1.1010048 sec
RG 192.89
DW 16.800 usec
DE 18.00 usec
TE 298.2 K
D1 2.00000000 sec
d11 0.03000000 sec
DELTA 1.89999998 sec
TD0 1
SFO1 125.7703637 MHz
NUC1 13C
P1 9.80 usec
PLW1 57.00000000 W
SFO2 500.1320005 MHz
NUC2 1H
CPDPRG[2 waltz16
PCPD2 80.00 usec
PLW2 20.00000000 W
PLW12 0.35778001 W
PLW13 0.22898000 W
F2 - Processing parameters
SI 32768
SF 125.7577885 MHz
WDW EM
SSB 0
LB 1.00 Hz
GB 0
PC 1.40
NHAc
HO
HCl
OH
C-10
210 200 190 180 170 160 150 140 130 120 110 100 90 80 70 60 50 40 30 20 10 0 ppm

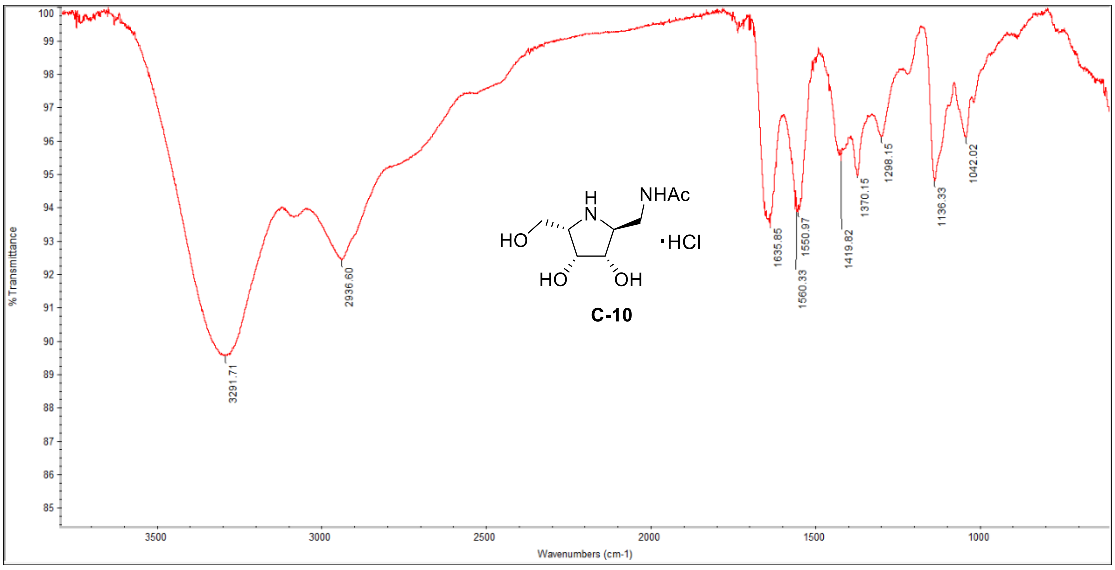
% Transmittance
100
99
98
97
96
95
94
93
92
91
90
89
88
87
86
85
3291.71
2936.60
1635.85
1560.33
1550.97
1419.82
1370.15
1298.15
1136.33
1042.02
NHAc
HO
HCl
OH
C-10
3500
3000
2500
2000
1500
1000
Wavenumbers (cm-1)

Compound **D-10**:

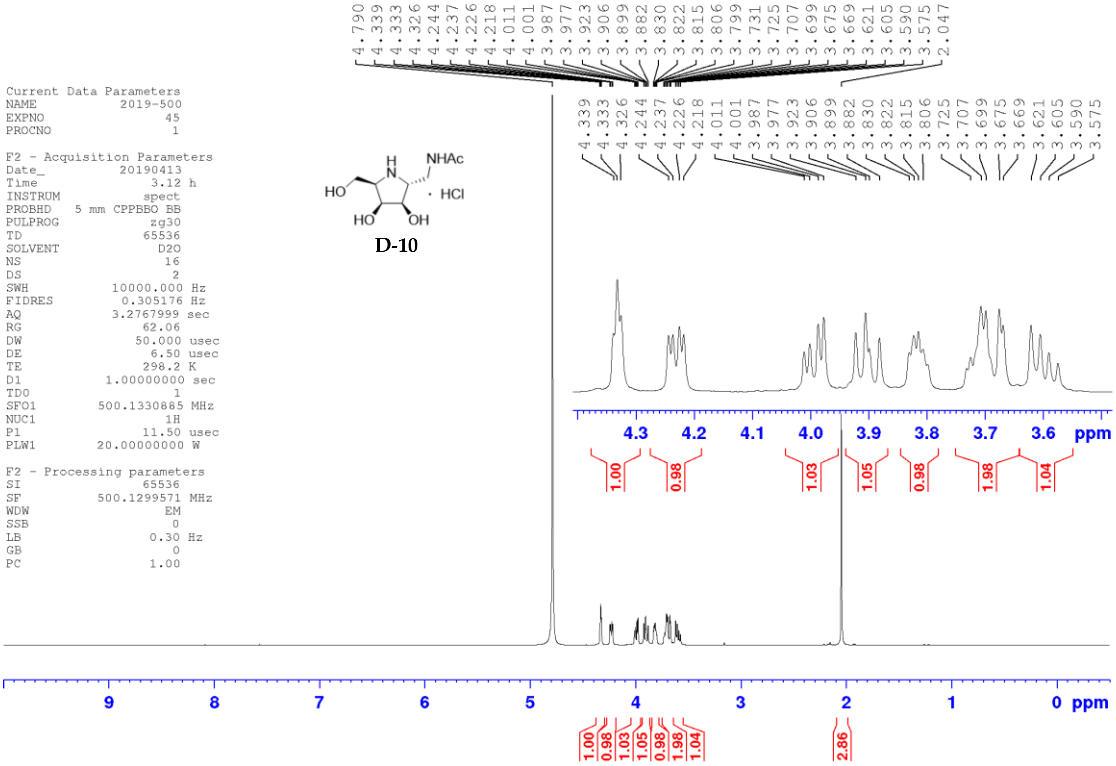

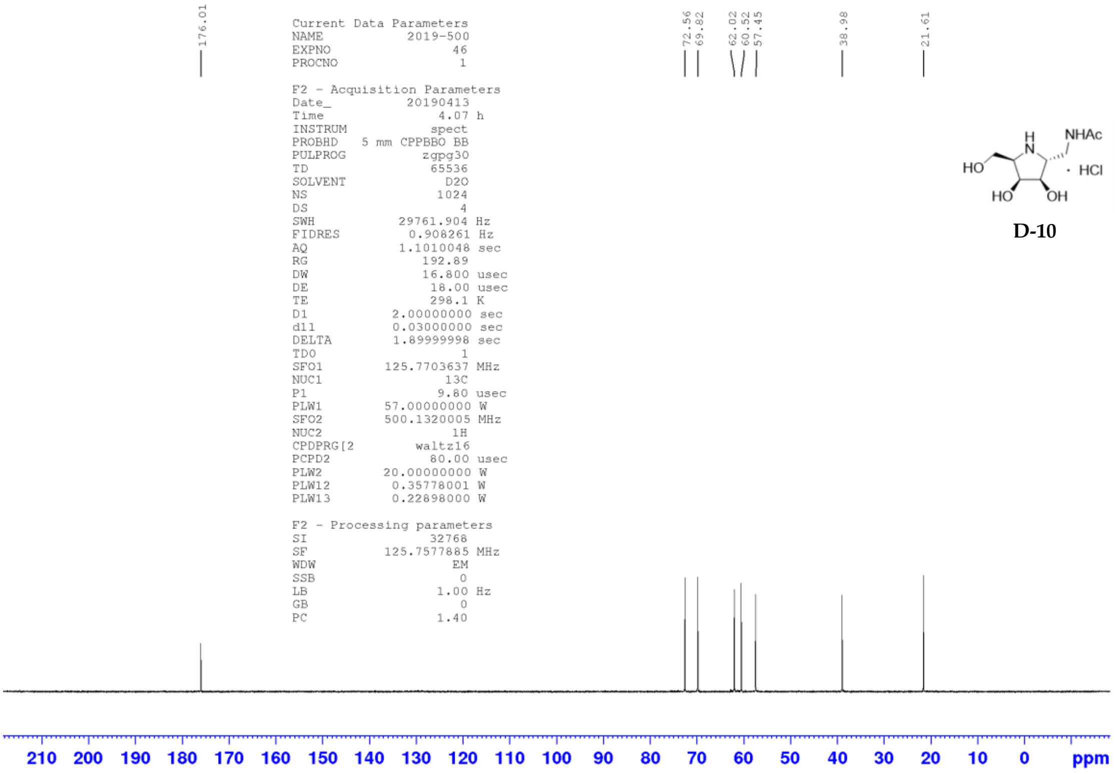

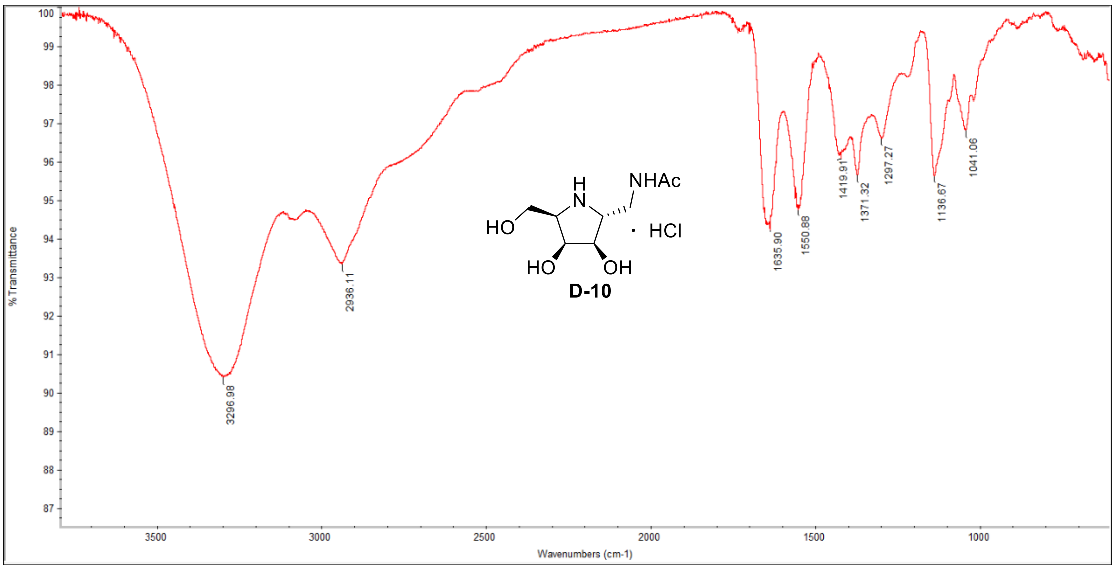

Compound **E-10a**:

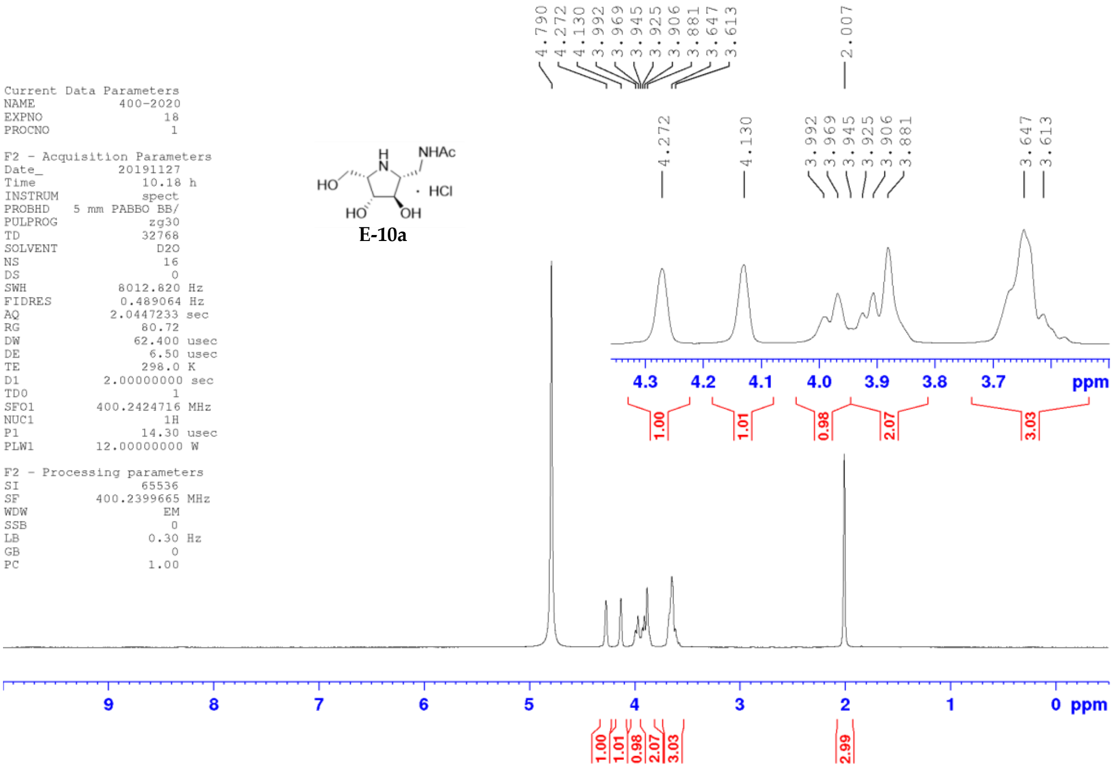

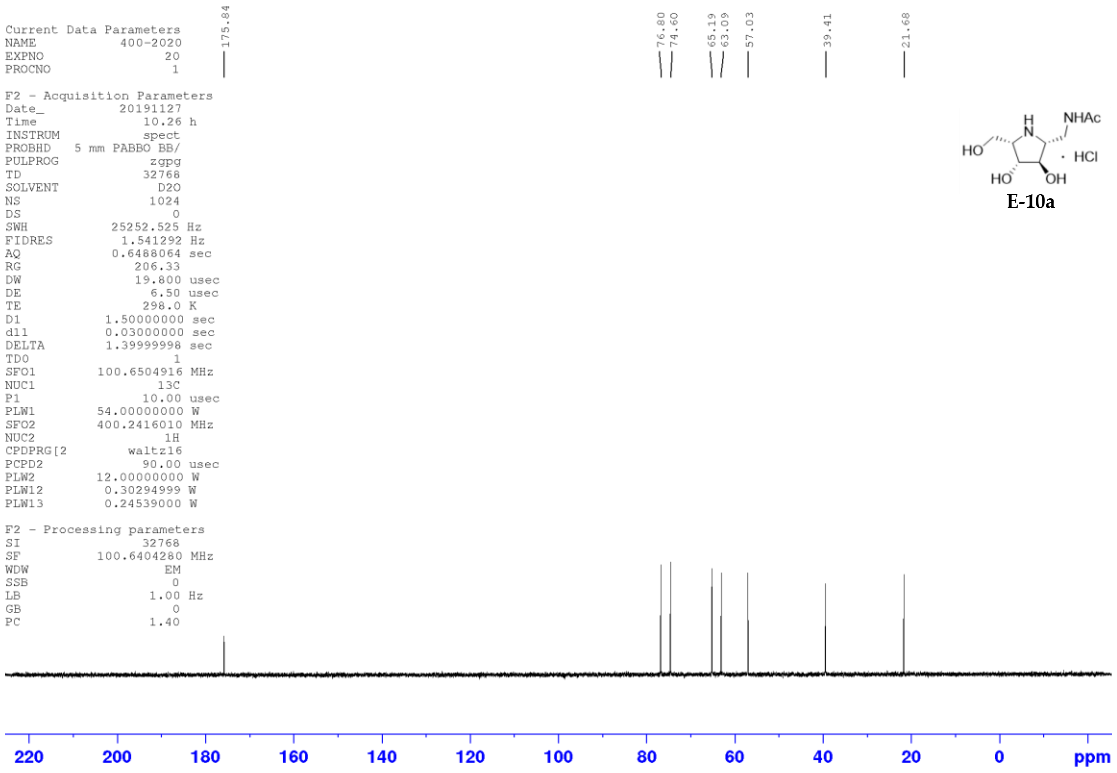
Current Data Parameters
NAME 400-2020
EXPNO 20
PROCNO 1
F2 - Acquisition Parameters
Date_ 20191127
Time 10.26 h
INSTRUM spect
PROBHD 5 mm PABBO BB/
PULPROG zgpg
TD 32768
SOLVENT D2O
NS 1024
DS 0
SWH 25252.525 Hz
FIDRES 1.541292 Hz
AQ 0.6488064 sec
RG 206.33
DW 19.800 usec
DE 6.50 usec
TE 298.0 K
D1 1.50000000 sec
d11 0.03000000 sec
DELTA 1.39999998 sec
TD0 1
SFO1 100.6504916 MHz
NUC1 13C
P1 10.00 usec
PLW1 54.00000000 W
SFO2 400.2416010 MHz
NUC2 1H
CPDPRG[2 waltz16
PCPD2 90.00 usec
PLW2 12.00000000 W
PLW12 0.30294999 W
PLW13 0.24539000 W
F2 - Processing parameters
SI 32768
SF 100.6404280 MHz
WDW EM
SSB 0
LB 1.00 Hz
GB 0
PC 1.40
175.84
76.80
74.60
65.19
63.09
57.03
39.41
21.68
HO
H
N
NHAc
· HCl
HO
OH
E-10a
220
200
180
160
140
120
100
80
60
40
20
0
ppm

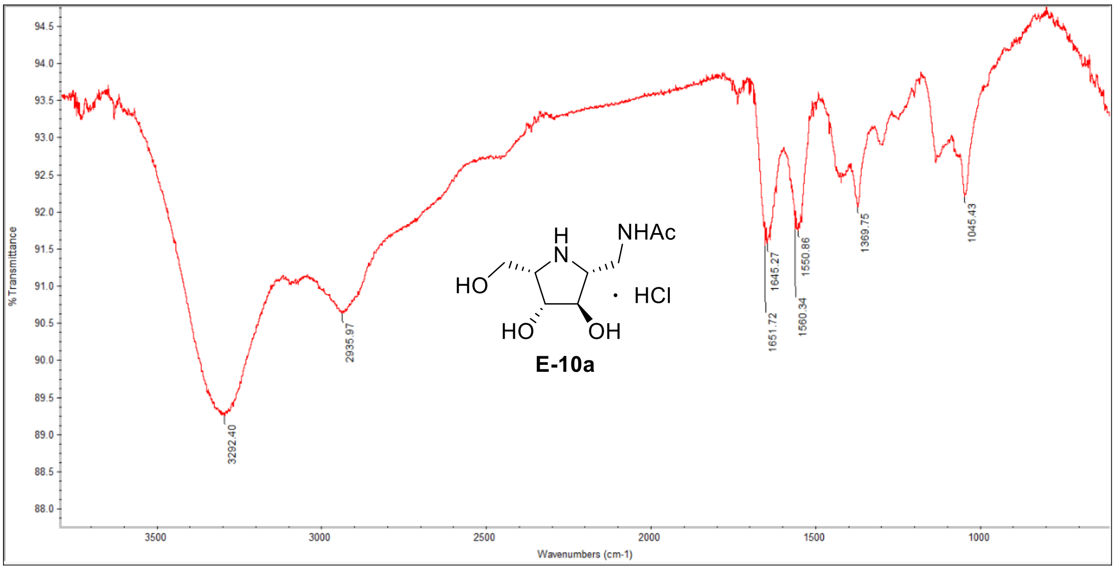
% Transmittance
94.5
94.0
93.5
93.0
92.5
92.0
91.5
91.0
90.5
90.0
89.5
89.0
88.5
88.0
3292.40
2935.97
1651.72
1645.27
1560.34
1550.86
1369.75
1045.43
HO
H
N
NHAc
· HCl
HO
OH
E-10a
3500
3000
2500
2000
1500
1000
Wavenumbers (cm-1)

Compound **E-10b**:

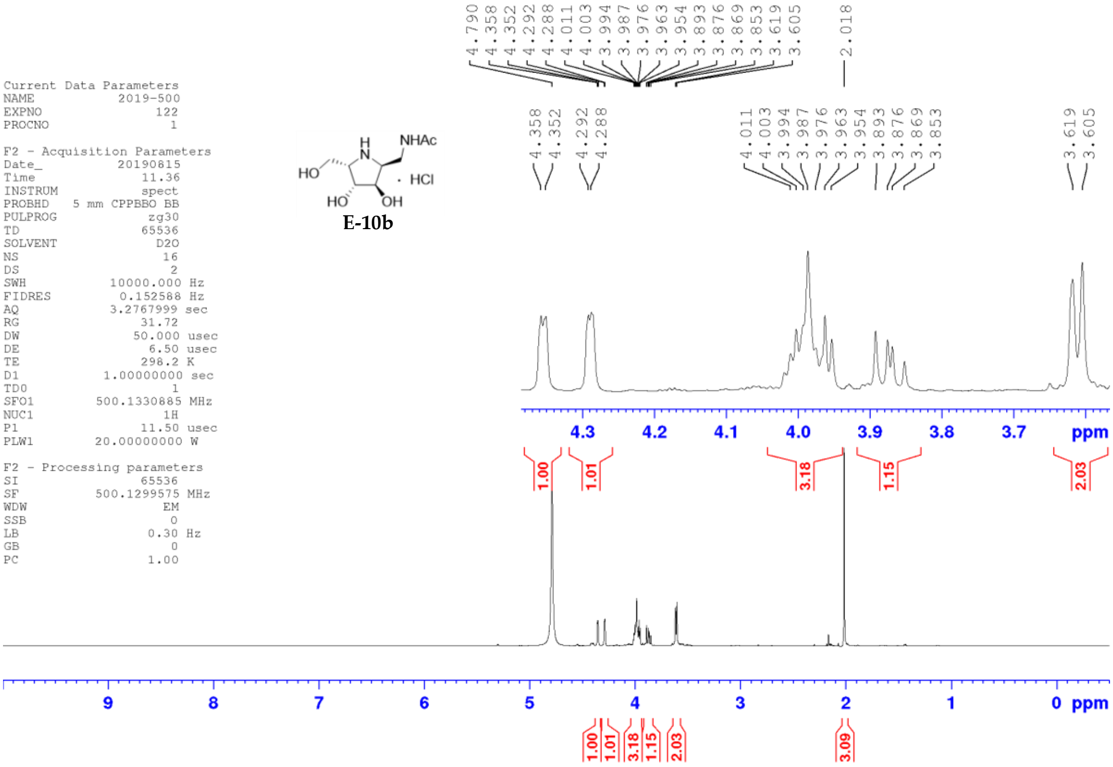

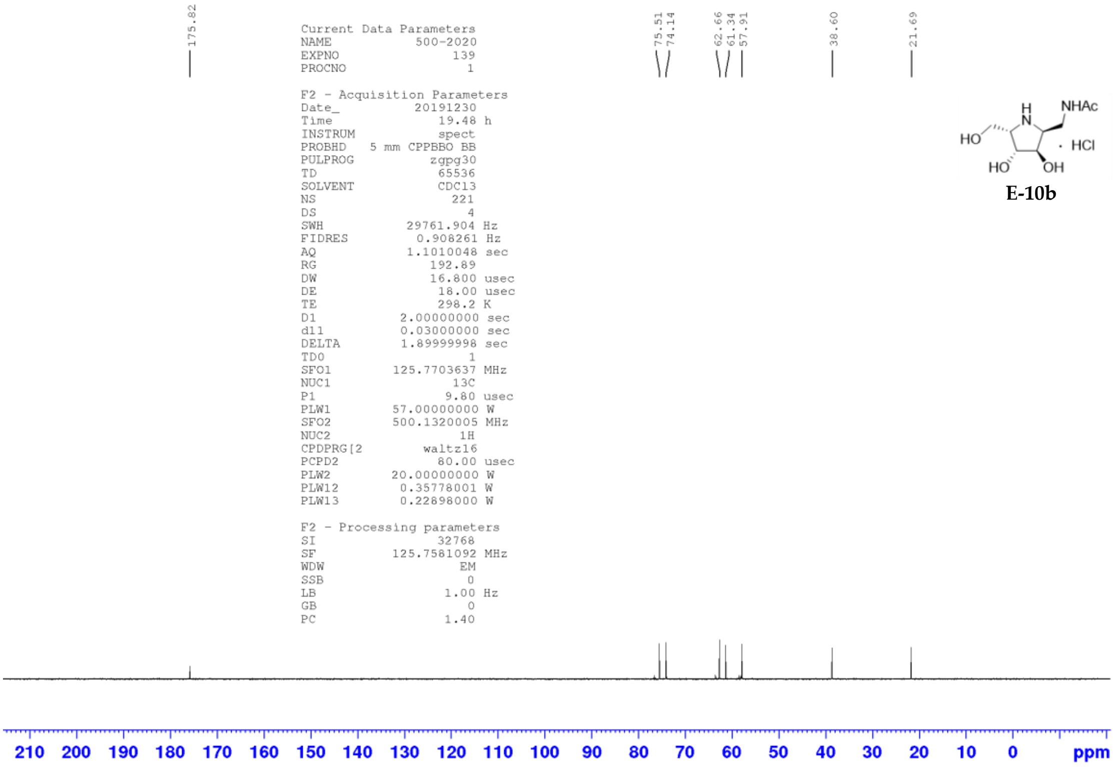

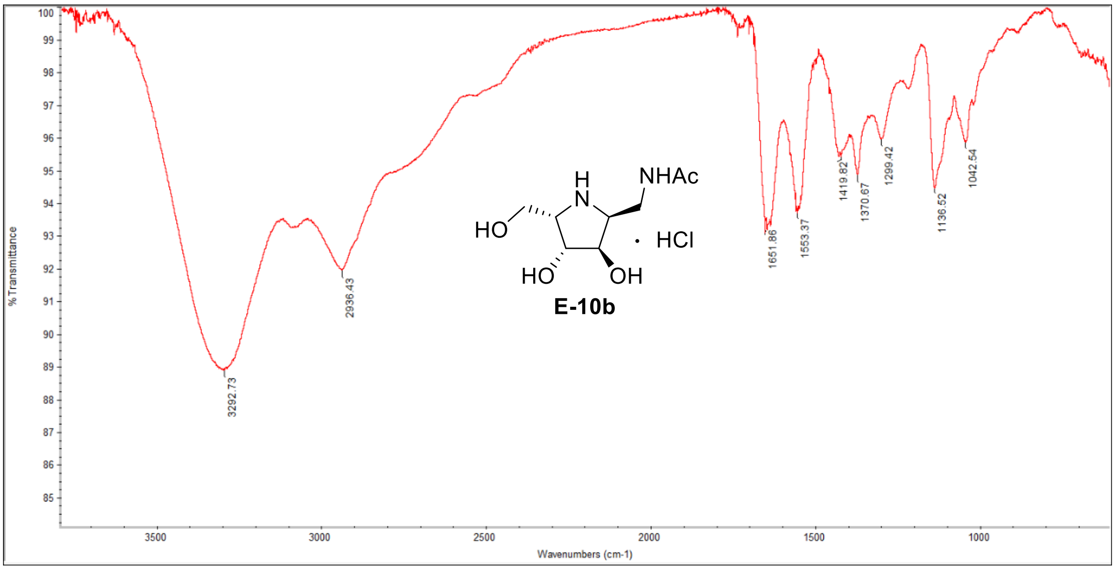

Compound **F-10a**:

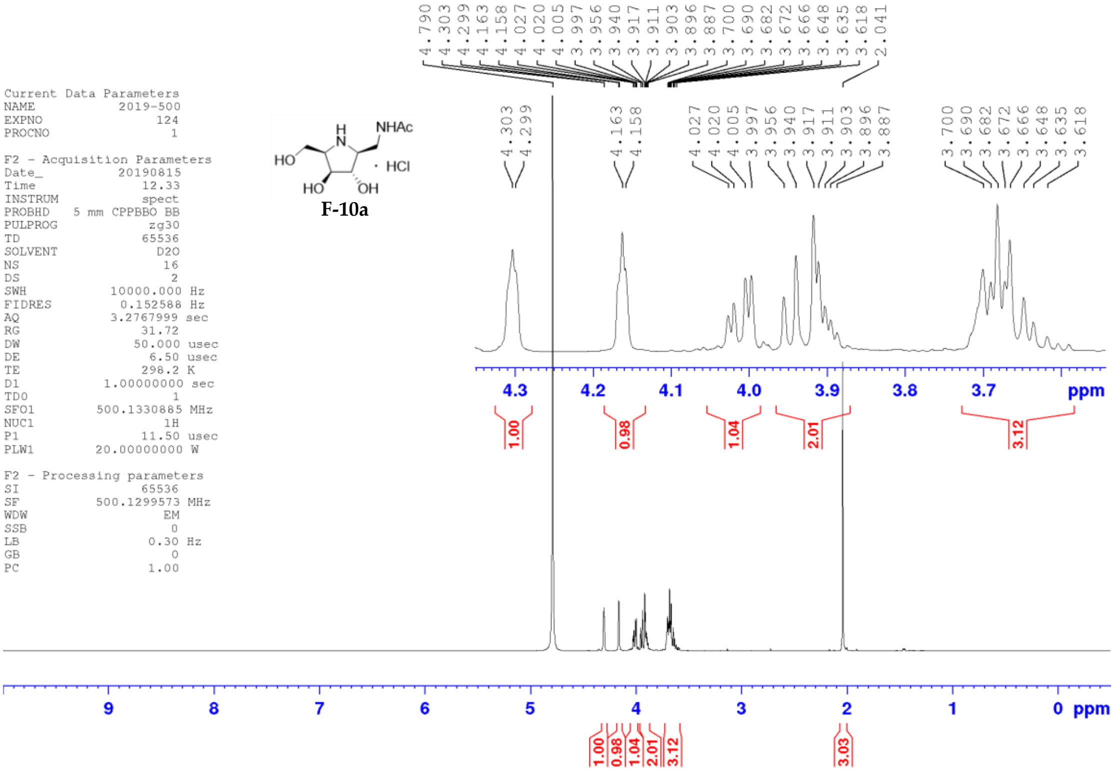

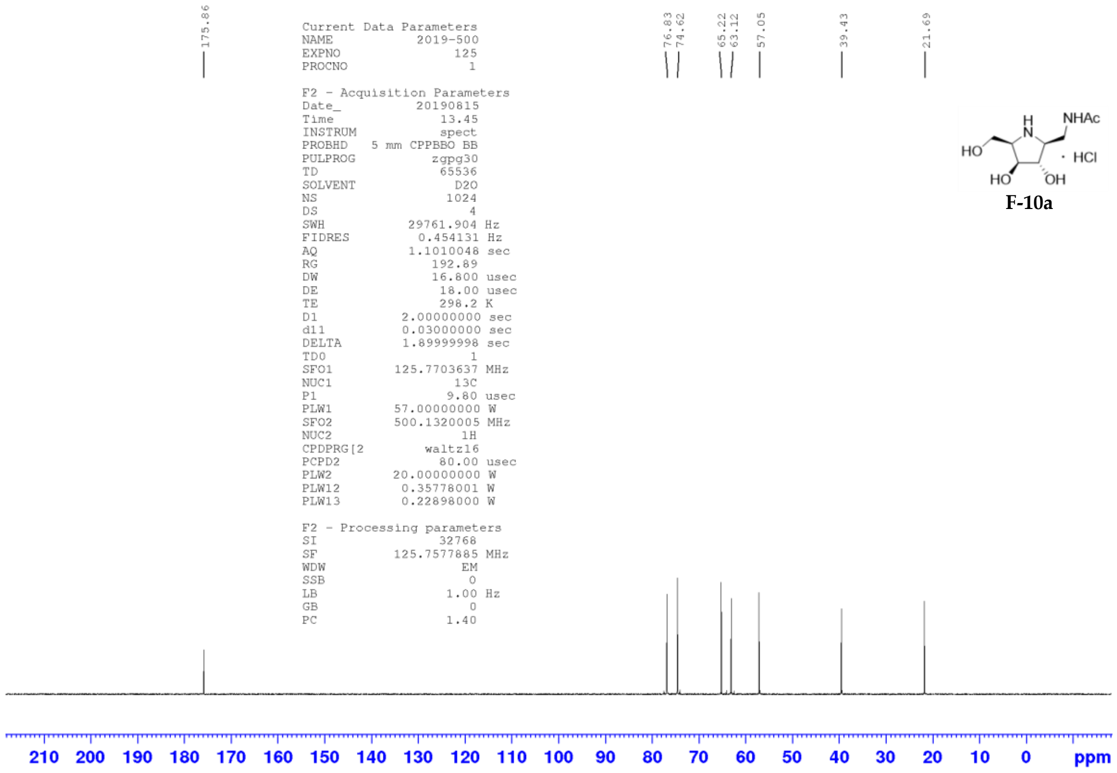
175.86
76.83
74.62
65.22
63.12
57.05
39.43
21.69
Current Data Parameters
NAME 2019-500
EXPNO 125
PROCNO 1
F2 - Acquisition Parameters
Date_ 20190815
Time 13.45
INSTRUM spect
PROBHD 5 mm CPPBBO BB
PULPROG zgpg30
TD 65536
SOLVENT D2O
NS 1024
DS 4
SWH 29761.904 Hz
FIDRES 0.454131 Hz
AQ 1.1010048 sec
RG 192.89
DW 16.800 usec
DE 18.00 usec
TE 298.2 K
D1 2.00000000 sec
d11 0.03000000 sec
DELTA 1.89999998 sec
TD0 1
SFO1 125.7703637 MHz
NUC1 13C
P1 9.80 usec
PLW1 57.00000000 W
SFO2 500.1320005 MHz
NUC2 1H
CPDPRG[2 waltz16
PCPD2 80.00 usec
PLW2 20.00000000 W
PLW12 0.35778001 W
PLW13 0.22898000 W
F2 - Processing parameters
SI 32768
SF 125.7577885 MHz
WDW EM
SSB 0
LB 1.00 Hz
GB 0
PC 1.40
210 200 190 180 170 160 150 140 130 120 110 100 90 80 70 60 50 40 30 20 10 0 ppm
NHAc
HO
HO
OH
HCl

F-10a

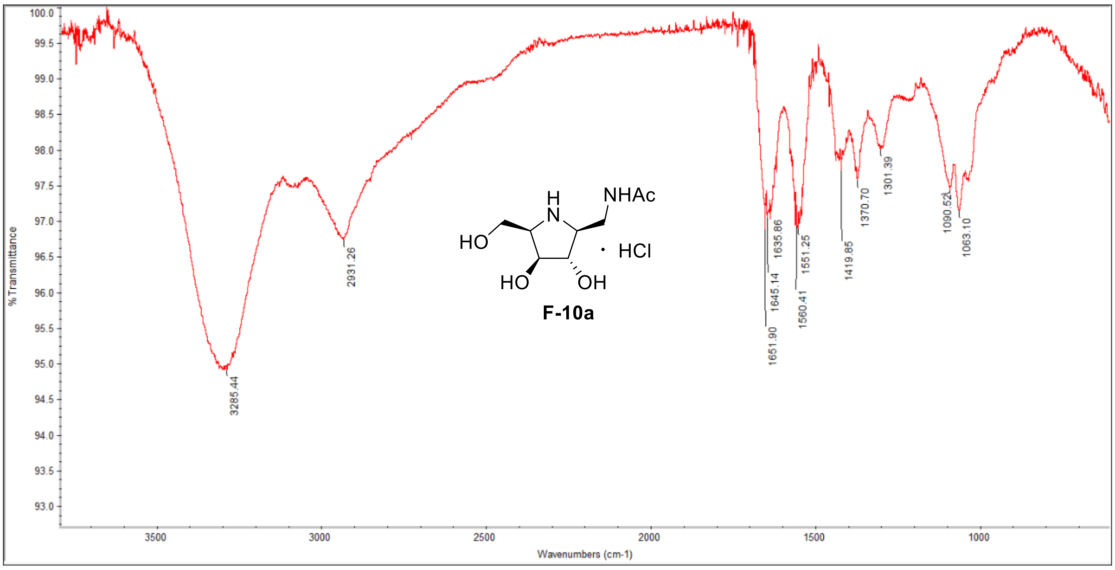
% Transmittance
Wavenumbers (cm-1)
3285.44
2931.26
1651.90
1645.14
1635.86
1560.41
1551.25
1419.85
1370.70
1301.39
1090.52
1063.10
F-10a

Compound **F-10b**:

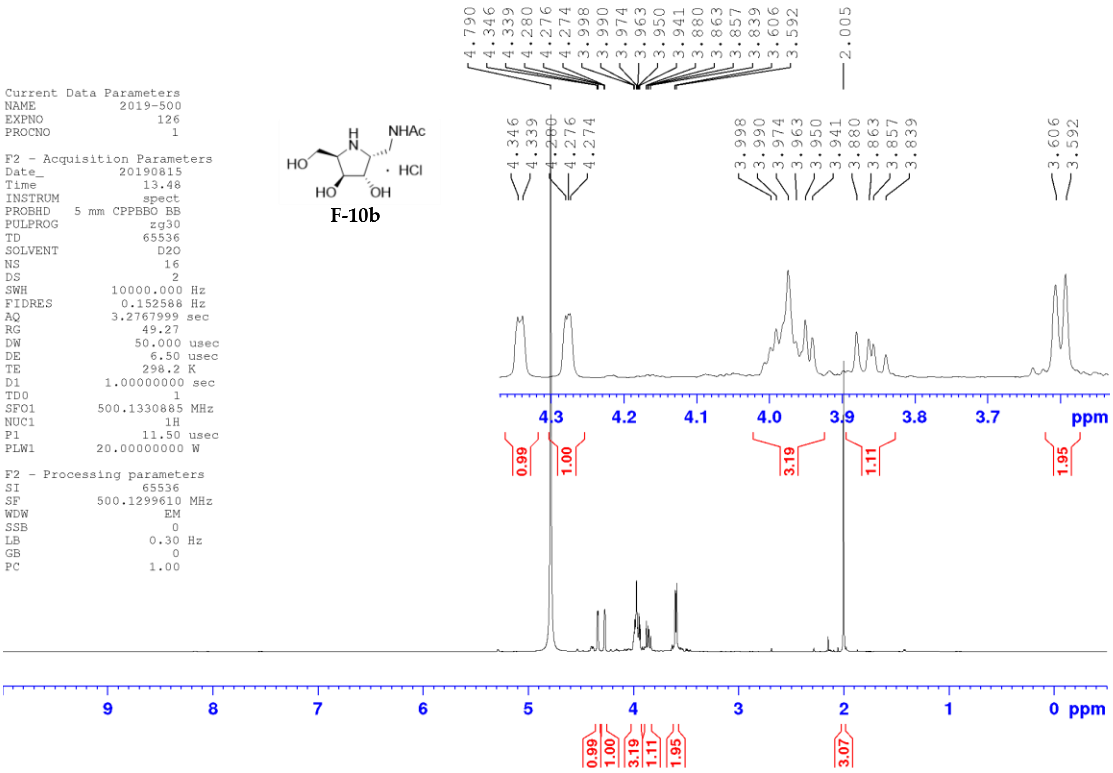

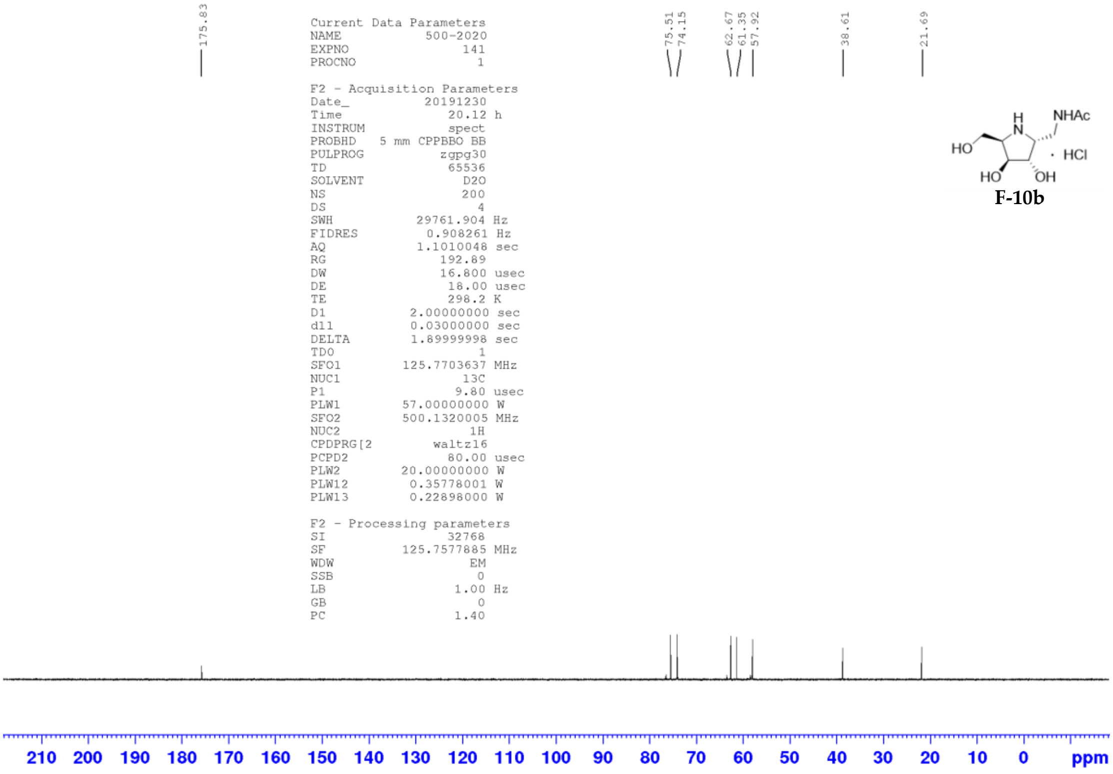

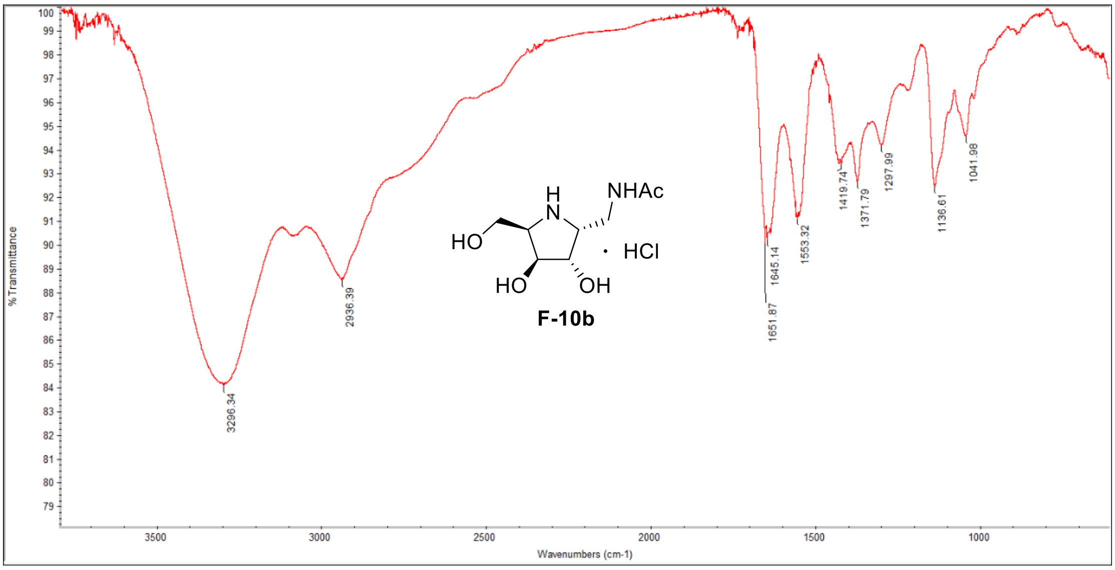

Compound **G-10**:

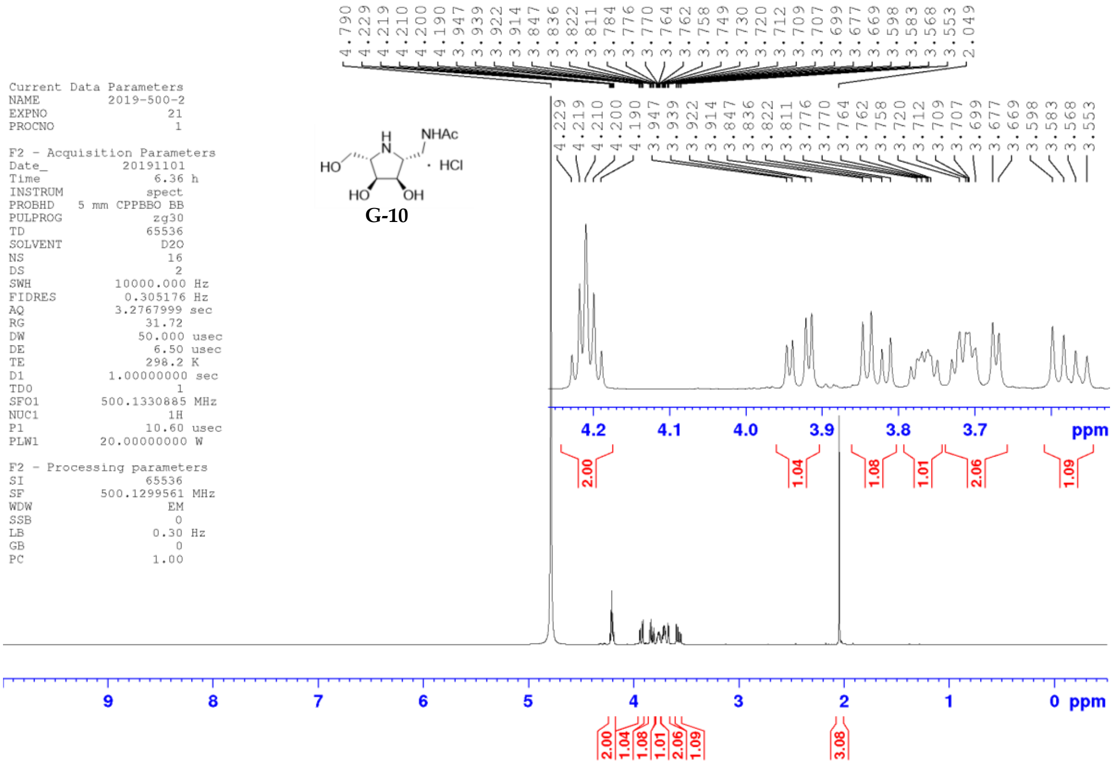

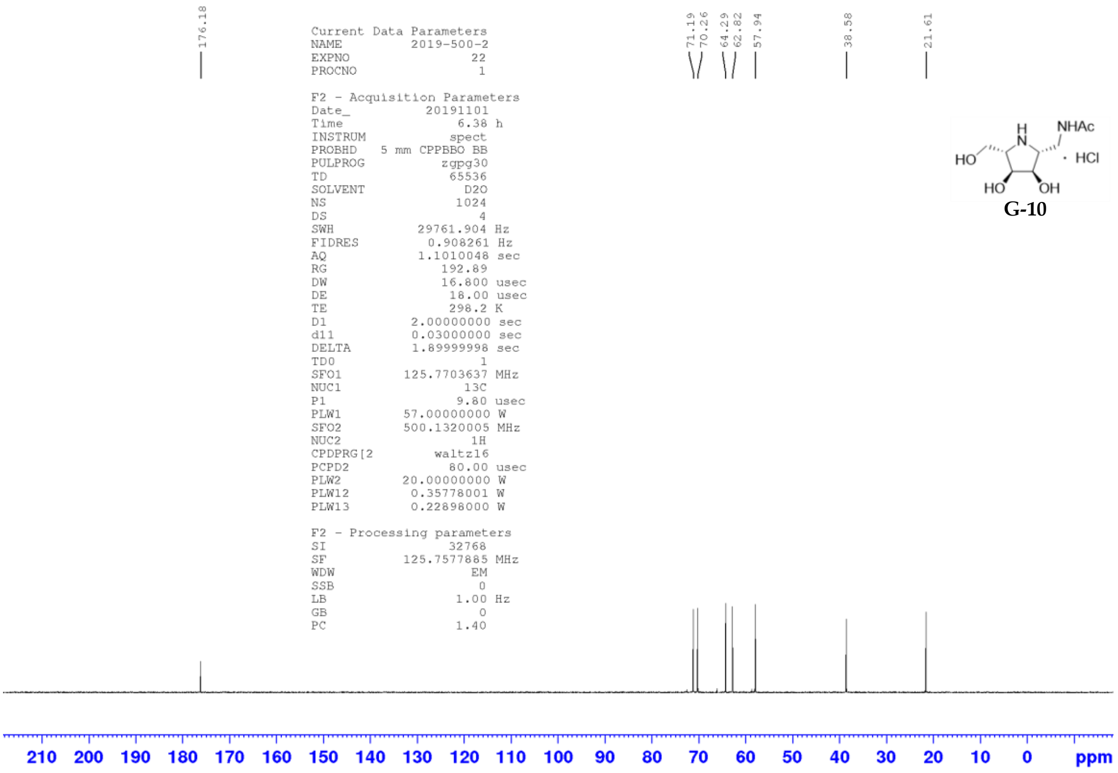

176.18
Current Data Parameters
NAME 2019-500-2
EXPNO 22
PROCNO 1
F2 - Acquisition Parameters
Date_ 20191101
Time 6.38 h
INSTRUM spect
PROBHD 5 mm CPPBBO BB
PULPROG zgpg30
TD 65536
SOLVENT D2O
NS 1024
DS 4
SWH 29761.904 Hz
FIDRES 0.908261 Hz
AQ 1.1010048 sec
RG 192.89
DW 16.800 usec
DE 18.00 usec
TE 298.2 K
D1 2.00000000 sec
d11 0.03000000 sec
DELTA 1.89999998 sec
TD0 1
SFO1 125.7703637 MHz
NUC1 13C
P1 9.80 usec
PLW1 57.00000000 W
SFO2 500.1320005 MHz
NUC2 1H
CPDPRG[2 waltz16
PCPD2 80.00 usec
PLW2 20.00000000 W
PLW12 0.35778001 W
PLW13 0.22898000 W
F2 - Processing parameters
SI 32768
SF 125.7577885 MHz
WDW EM
SSB 0
LB 1.00 Hz
GB 0
PC 1.40
71.19
70.26
64.29
62.82
57.94
38.58
21.61
HO
H
N
NHAc
· HCl
HO
OH
G-10
210 200 190 180 170 160 150 140 130 120 110 100 90 80 70 60 50 40 30 20 10 0 ppm

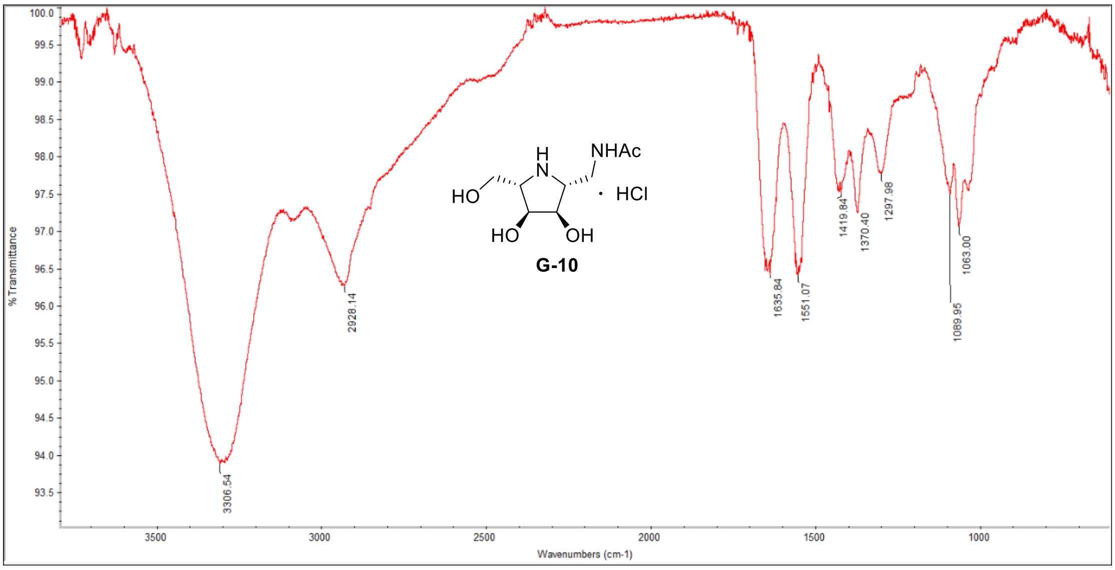

% Transmittance
100.0
99.5
99.0
98.5
98.0
97.5
97.0
96.5
96.0
95.5
95.0
94.5
94.0
93.5
3306.54
2928.14
1635.84
1551.07
1419.84
1370.40
1297.98
1089.95
1063.00
HO
H
N
NHAc
· HCl
HO
OH
G-10
3500 3000 2500 2000 1500 1000
Wavenumbers (cm-1)

Compound **H-10**:

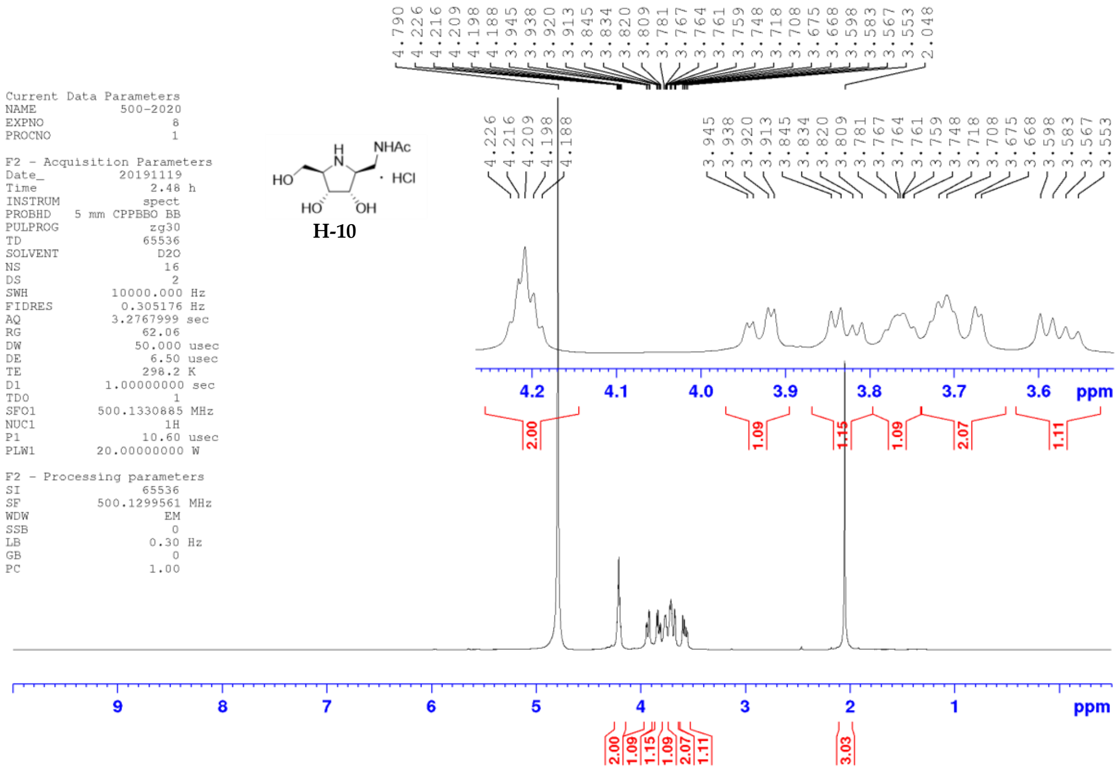

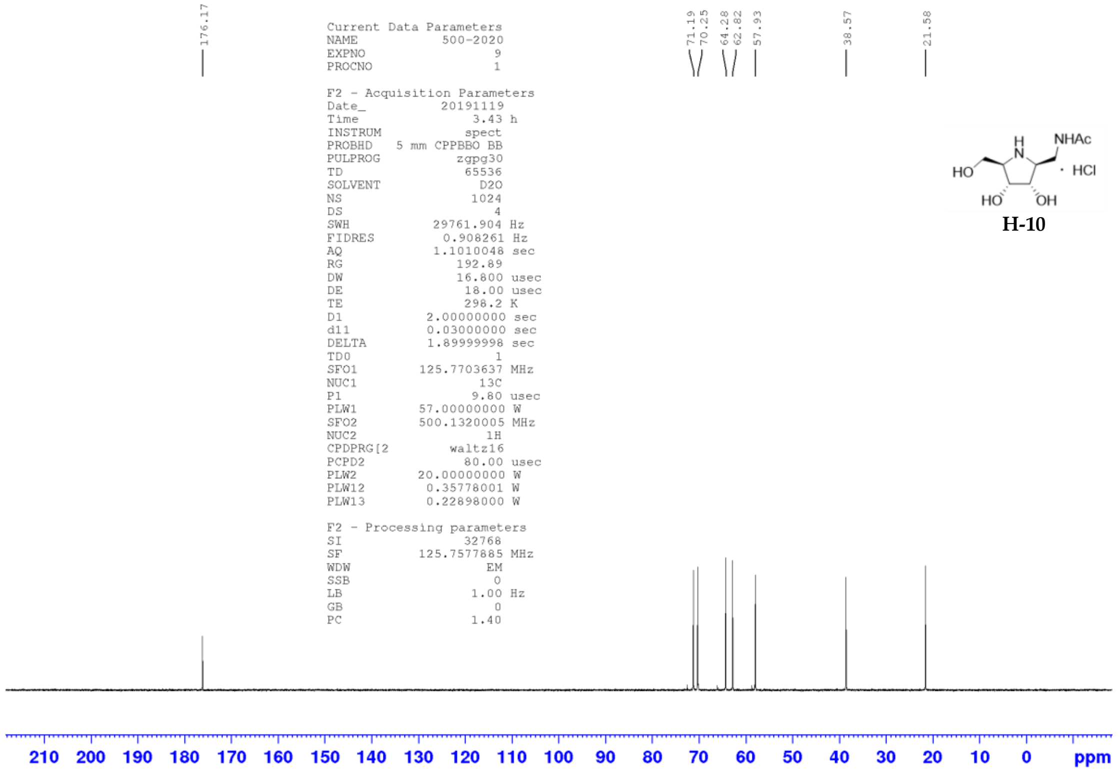

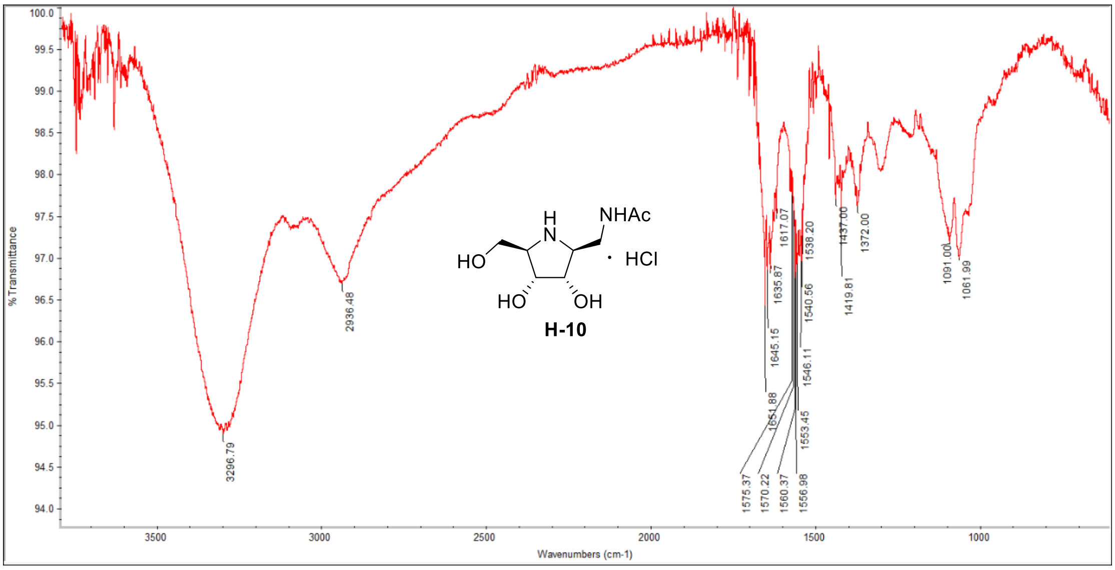

Compound **A-11**:

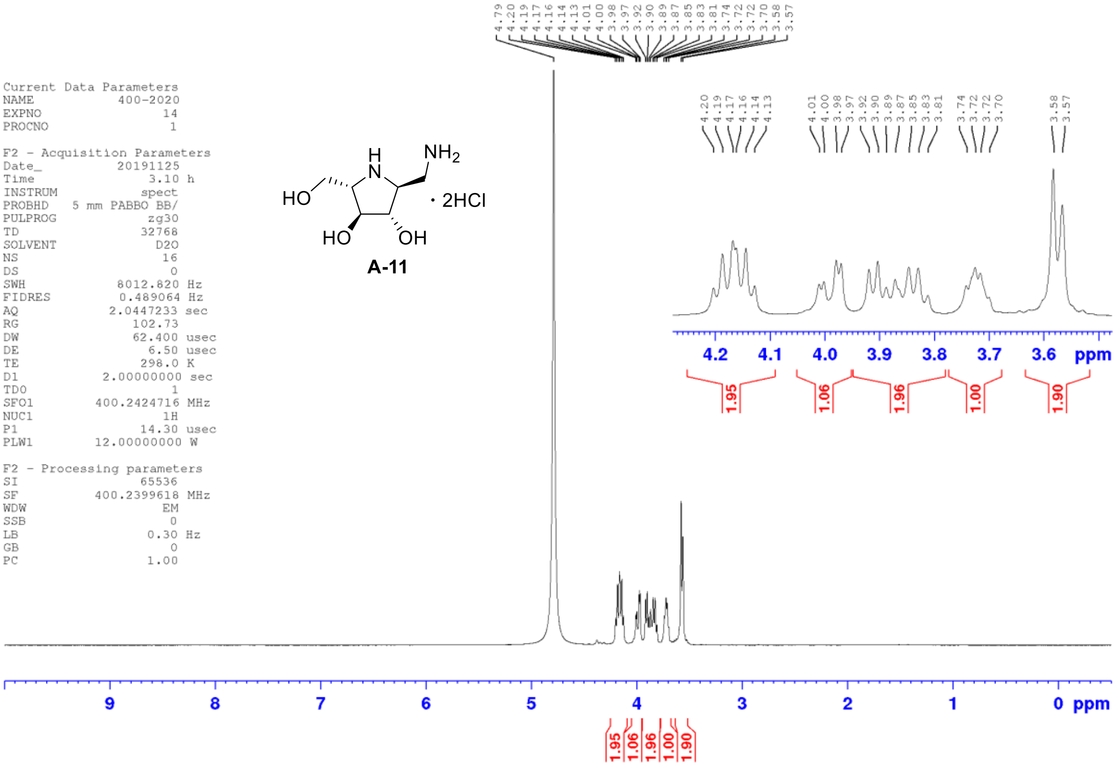

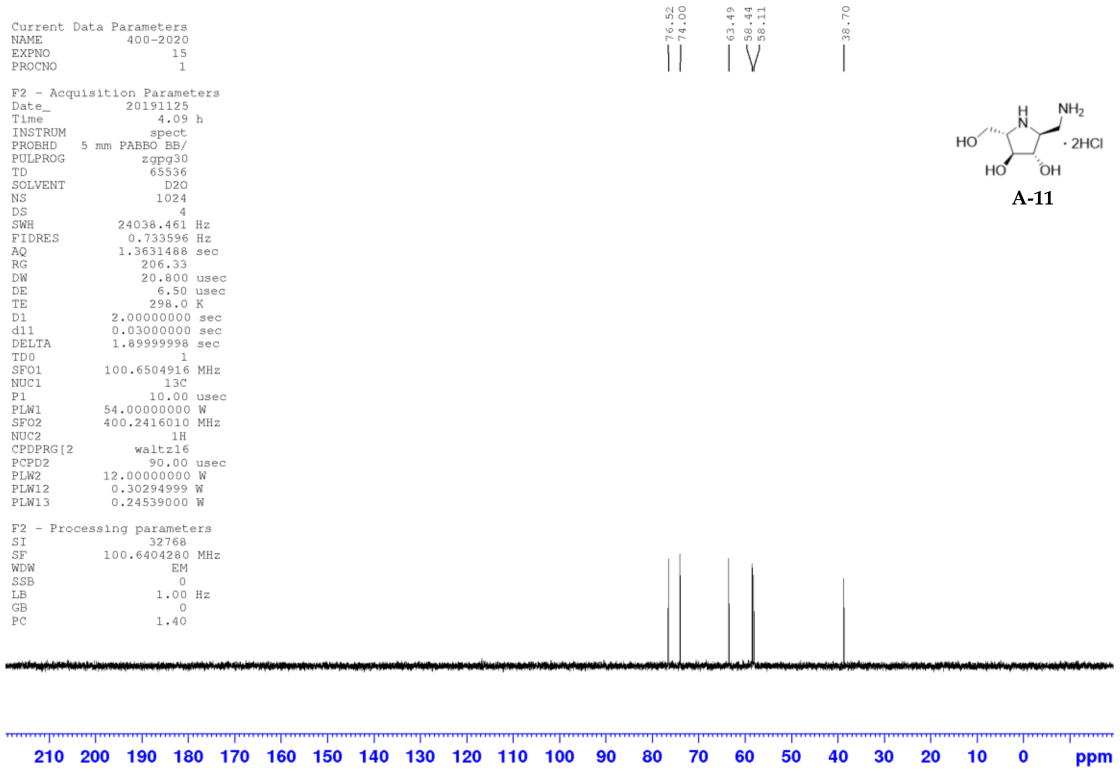
Current Data Parameters
NAME 400-2020
EXPNO 15
PROCNO 1
F2 - Acquisition Parameters
Date_ 20191125
Time 4.09 h
INSTRUM spect
PROBHD 5 mm PABBO BB/
PULPROG zgpg30
TD 65536
SOLVENT D2O
NS 1024
DS 4
SWH 24038.461 Hz
FIDRES 0.733596 Hz
AQ 1.3631488 sec
RG 206.33
DW 20.800 usec
DE 6.50 usec
TE 298.0 K
D1 2.00000000 sec
d11 0.03000000 sec
DELTA 1.89999998 sec
TD0 1
SFO1 100.6504916 MHz
NUC1 13C
P1 10.00 usec
PLW1 54.00000000 W
SFO2 400.2416010 MHz
NUC2 1H
CPDPRG[2 waltz16
PCPD2 90.00 usec
PLW2 12.00000000 W
PLW12 0.30294999 W
PLW13 0.24539000 W
F2 - Processing parameters
SI 32768
SF 100.6404280 MHz
WDW EM
SSB 0
LB 1.00 Hz
GB 0
PC 1.40
76.52
74.00
63.49
58.44
58.11
38.70
210 200 190 180 170 160 150 140 130 120 110 100 90 80 70 60 50 40 30 20 10 0 ppm
H
N
NH2
HO
· 2HCl
HO
OH

A-11

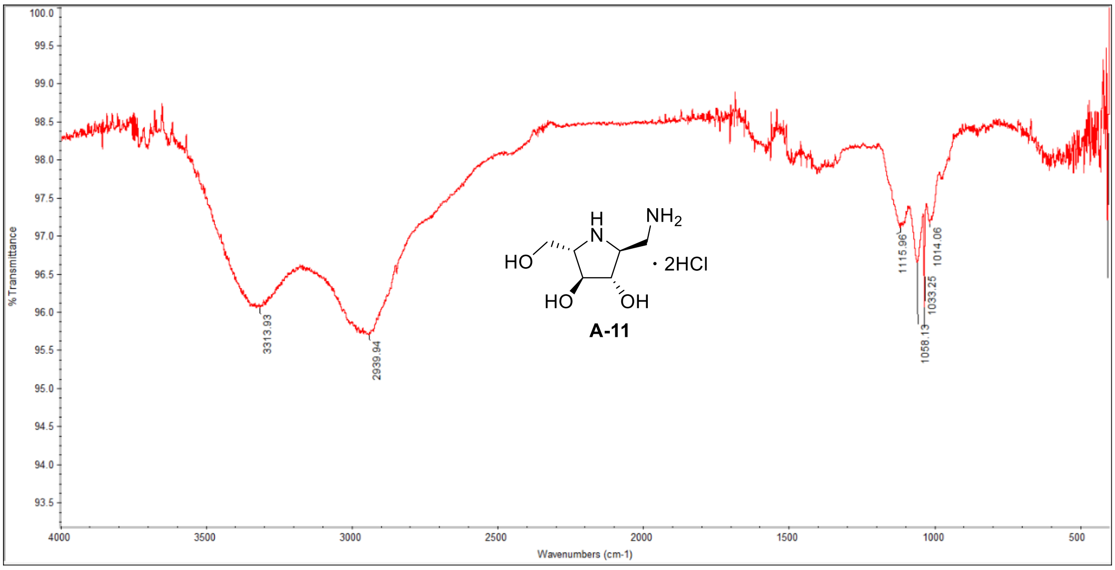
100.0
99.5
99.0
98.5
98.0
97.5
97.0
96.5
96.0
95.5
95.0
94.5
94.0
93.5
% Transmittance
3313.93
2939.94
1115.96
1058.13
1033.25
1014.06
H
N
NH2
HO
· 2HCl
HO
OH
A-11
4000
3500
3000
2500
2000
1500
1000
500
Wavenumbers (cm-1)

Compound **B-11**:

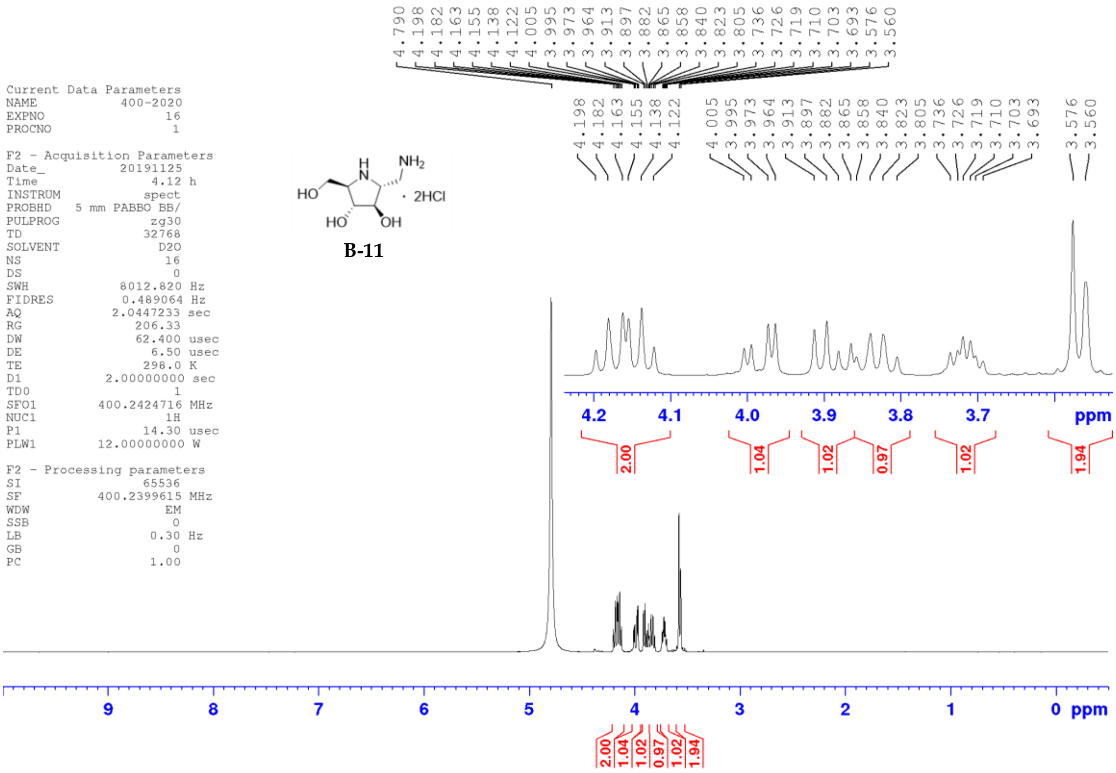

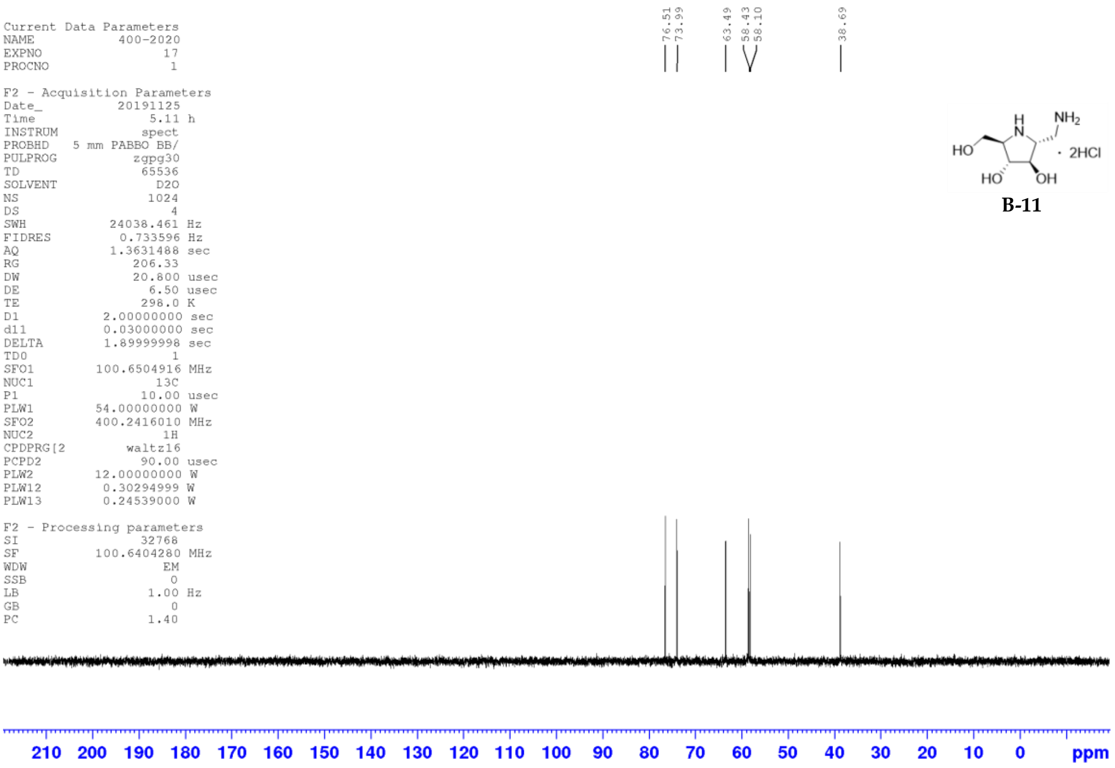

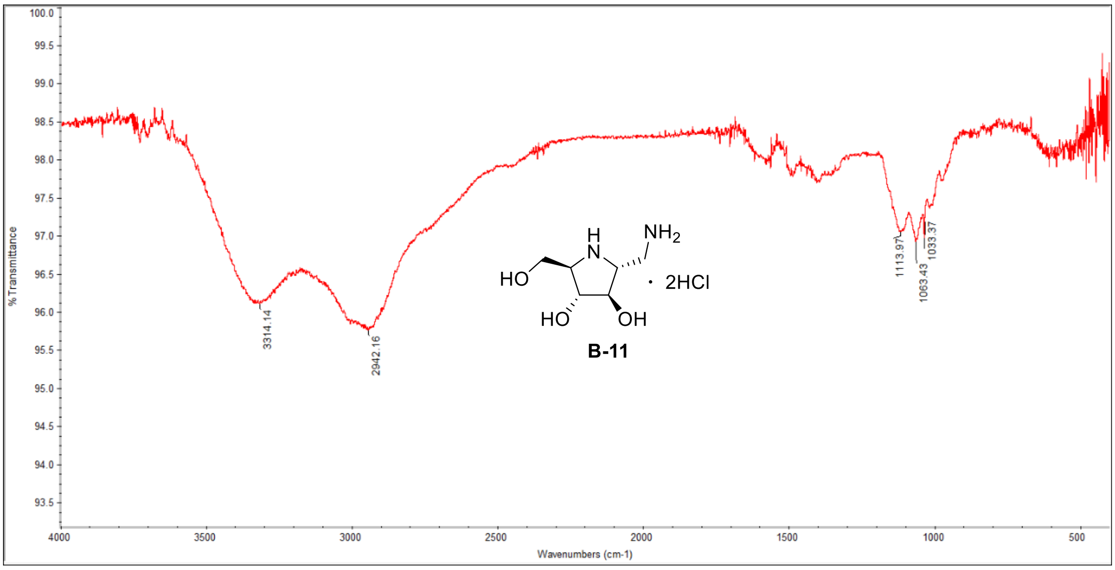

Compound **C-11**:

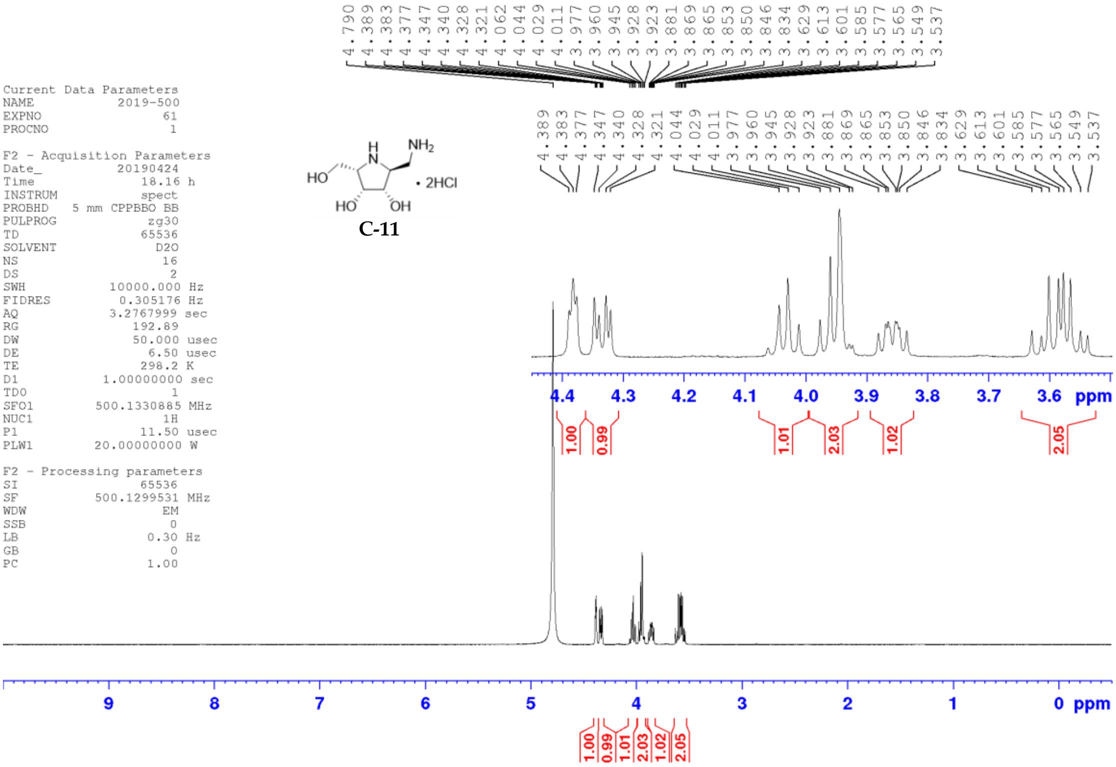

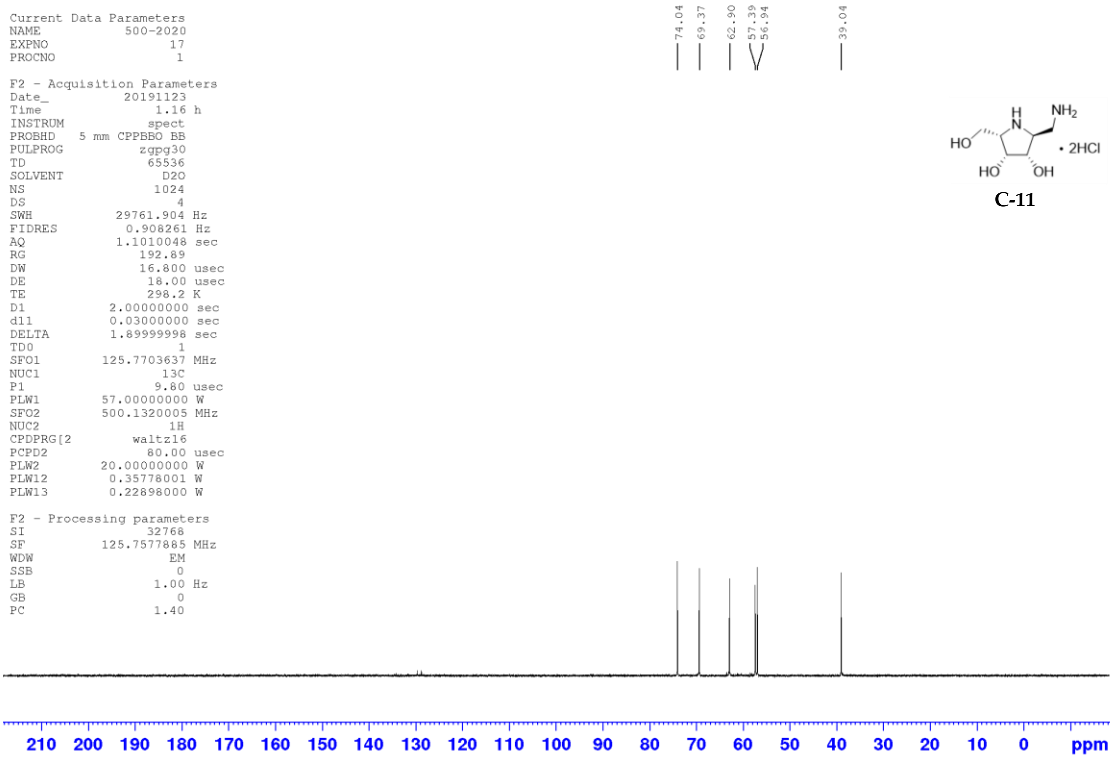
Current Data Parameters
NAME 500-2020
EXPNO 17
PROCNO 1
F2 - Acquisition Parameters
Date_ 20191123
Time 1.16 h
INSTRUM spect
PROBHD 5 mm CPPBBO BB
PULPROG zgpg30
TD 65536
SOLVENT D2O
NS 1024
DS 4
SWH 29761.904 Hz
FIDRES 0.908261 Hz
AQ 1.1010048 sec
RG 192.89
DW 16.800 usec
DE 18.00 usec
TE 298.2 K
D1 2.00000000 sec
d11 0.03000000 sec
DELTA 1.89999998 sec
TD0 1
SFO1 125.7703637 MHz
NUC1 13C
P1 9.80 usec
PLW1 57.00000000 W
SFO2 500.1320005 MHz
NUC2 1H
CPDPRG[2 waltz16
PCPD2 80.00 usec
PLW2 20.00000000 W
PLW12 0.35778001 W
PLW13 0.22898000 W
F2 - Processing parameters
SI 32768
SF 125.7577885 MHz
WDW EM
SSB 0
LB 1.00 Hz
GB 0
PC 1.40
74.04
69.37
62.90
57.39
56.94
39.04
HO
H
N
NH2
• 2HCl
HO
OH
C-11
210 200 190 180 170 160 150 140 130 120 110 100 90 80 70 60 50 40 30 20 10 0 ppm

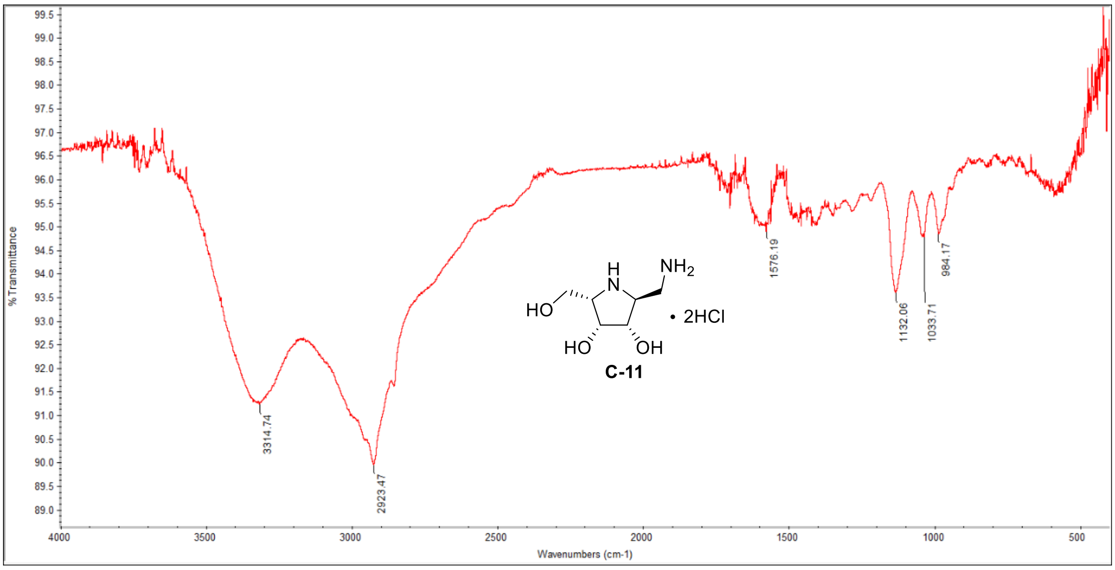
% Transmittance
99.5
99.0
98.5
98.0
97.5
97.0
96.5
96.0
95.5
95.0
94.5
94.0
93.5
93.0
92.5
92.0
91.5
91.0
90.5
90.0
89.5
89.0
3314.74
2923.47
1576.19
1132.06
1033.71
984.17
HO
H
N
NH2
• 2HCl
HO
OH
C-11
4000 3500 3000 2500 2000 1500 1000 500
Wavenumbers (cm-1)

Compound **D-11**:

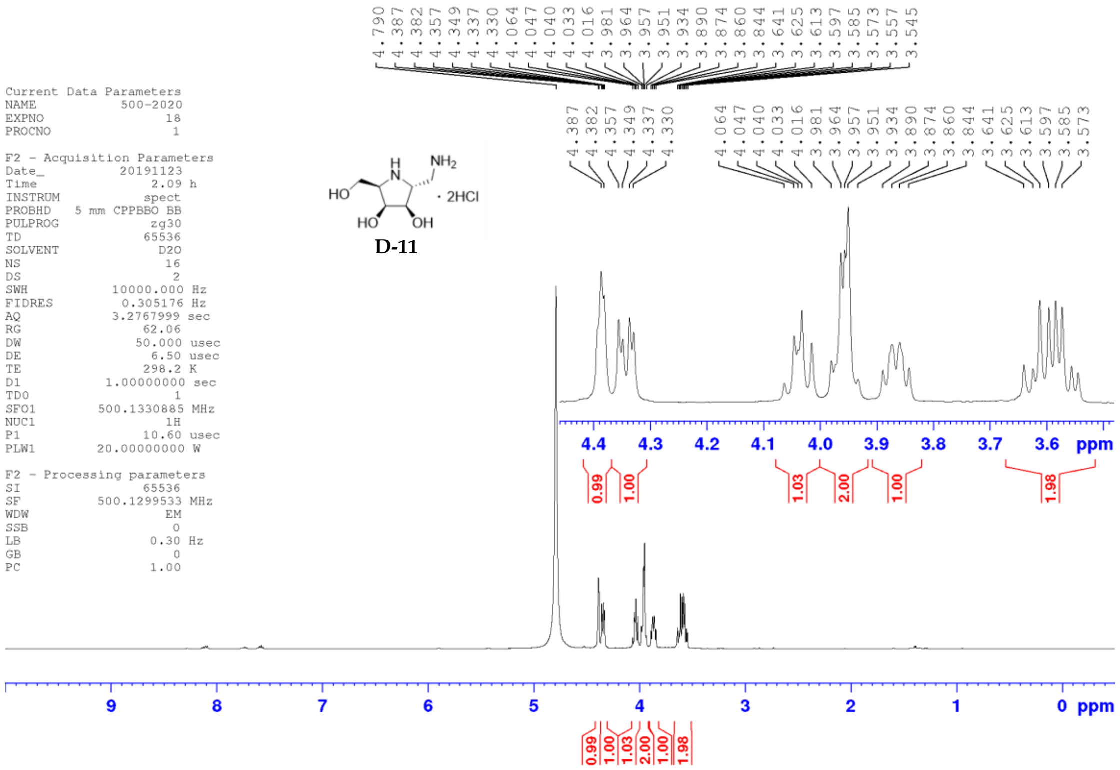

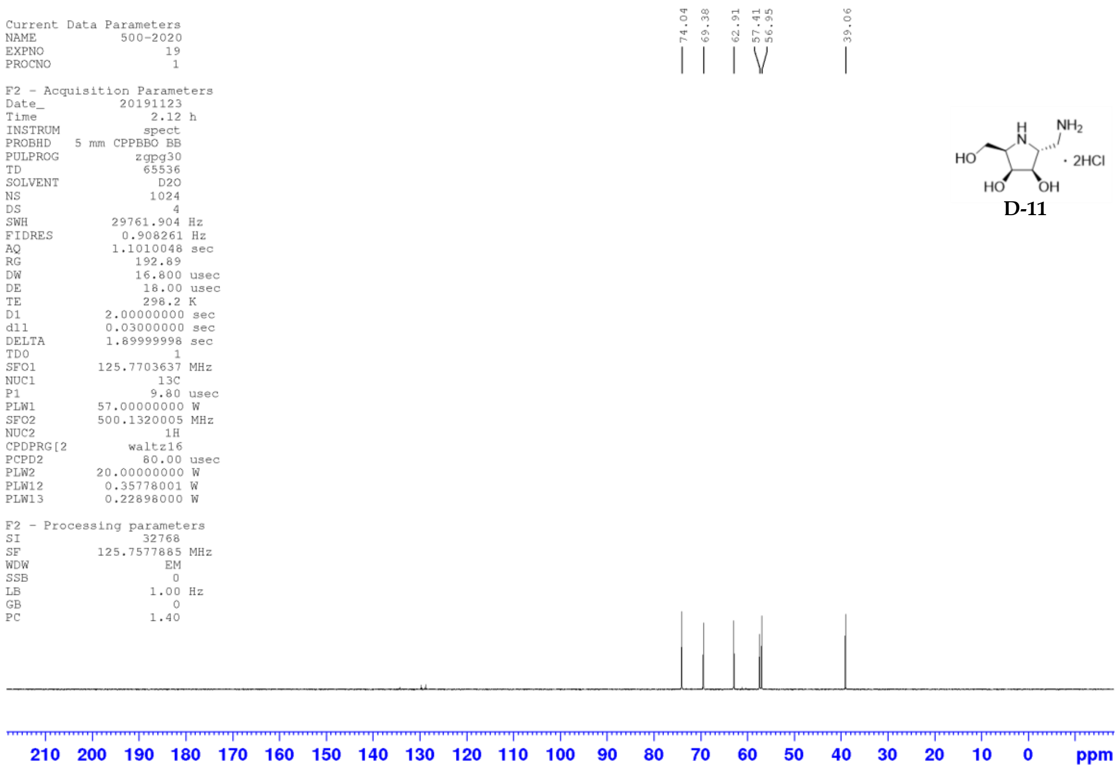

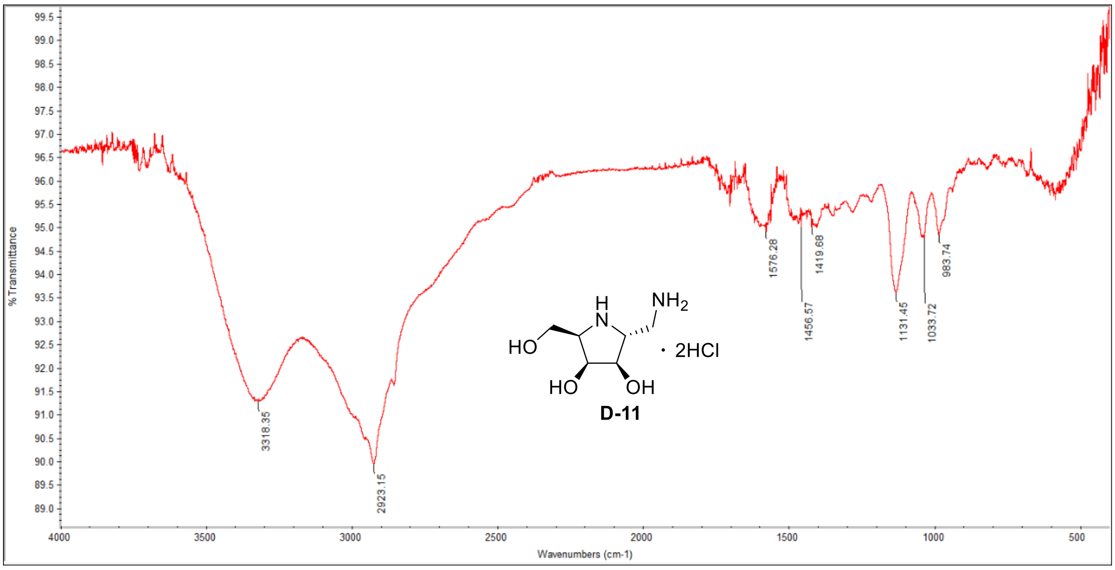

Compound **E-11a**:

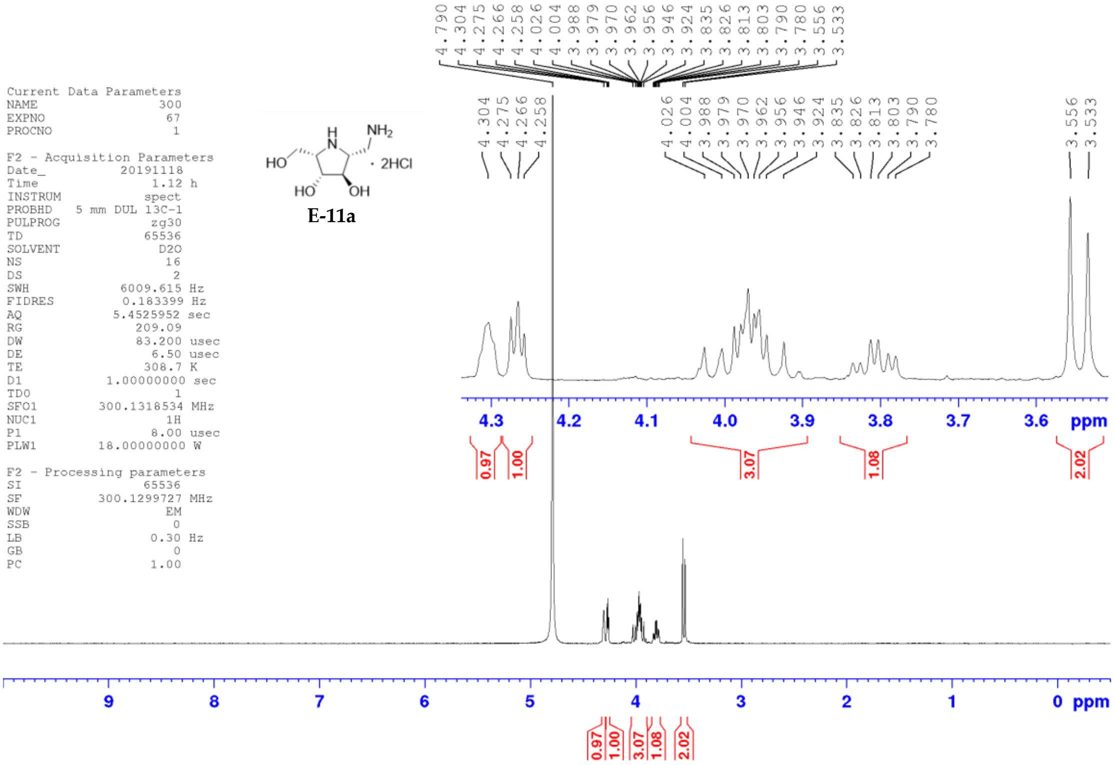

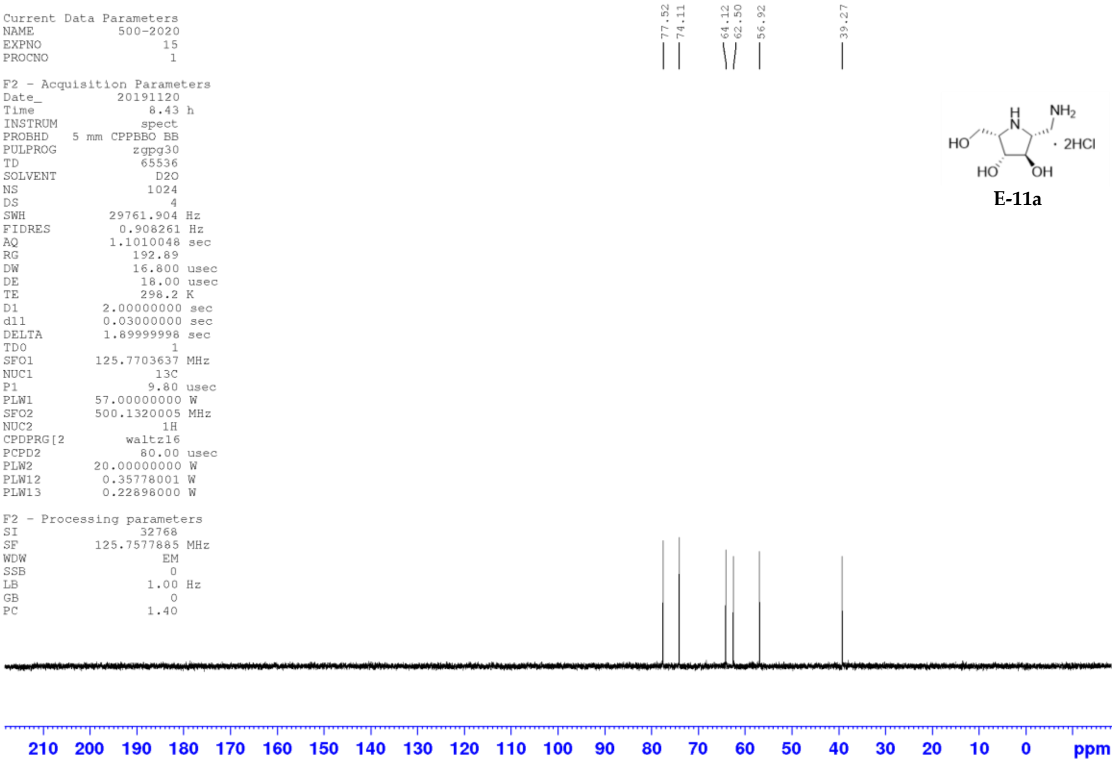
Current Data Parameters
NAME 500-2020
EXPNO 15
PROCNO 1
F2 - Acquisition Parameters
Date_ 20191120
Time 8.43 h
INSTRUM spect
PROBHD 5 mm CPPBBO BB
PULPROG zgpg30
TD 65536
SOLVENT D2O
NS 1024
DS 4
SWH 29761.904 Hz
FIDRES 0.908261 Hz
AQ 1.1010048 sec
RG 192.89
DW 16.800 usec
DE 18.00 usec
TE 298.2 K
D1 2.00000000 sec
d11 0.03000000 sec
DELTA 1.89999998 sec
TD0 1
SFO1 125.7703637 MHz
NUC1 13C
P1 9.80 usec
PLW1 57.00000000 W
SFO2 500.1320005 MHz
NUC2 1H
CPDPRG[2 waltz16
PCPD2 80.00 usec
PLW2 20.00000000 W
PLW12 0.35778001 W
PLW13 0.22898000 W
F2 - Processing parameters
SI 32768
SF 125.7577885 MHz
WDW EM
SSB 0
LB 1.00 Hz
GB 0
PC 1.40
77.52
74.11
64.12
62.50
56.92
39.27
NH2
HO
H
N
· 2HCl
HO
OH
E-11a
210 200 190 180 170 160 150 140 130 120 110 100 90 80 70 60 50 40 30 20 10 0 ppm

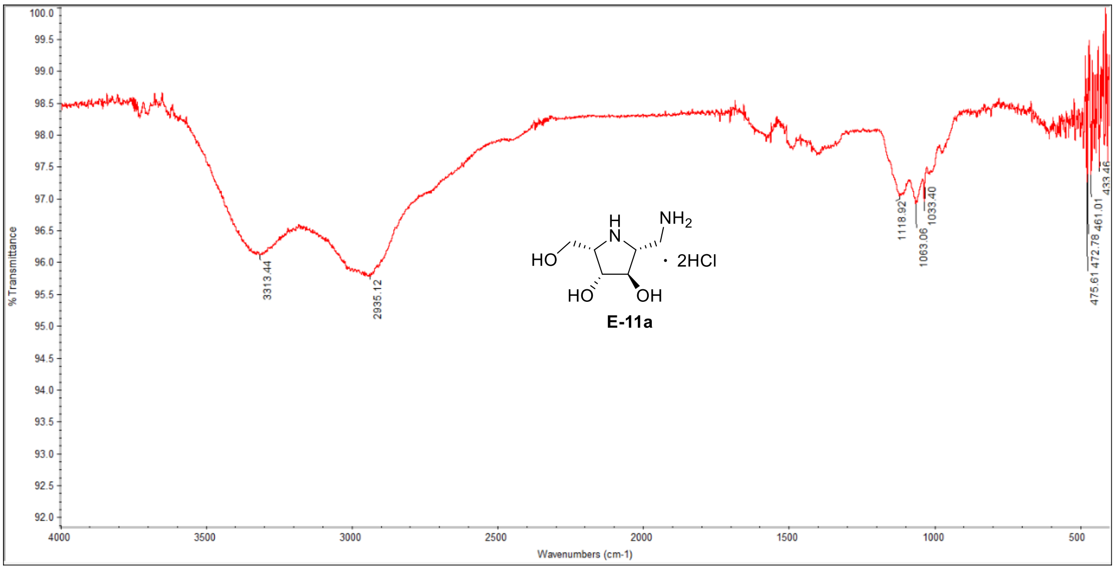
%Transmittance
100.0
99.5
99.0
98.5
98.0
97.5
97.0
96.5
96.0
95.5
95.0
94.5
94.0
93.5
93.0
92.5
92.0
3313.44
2935.12
1118.92
1063.06
1033.40
475.61
472.78
461.01
433.46
H
NH2
HO
N
· 2HCl
HO
OH
E-11a
4000
3500
3000
2500
2000
1500
1000
500
Wavenumbers (cm-1)

Compound **E-11b**:

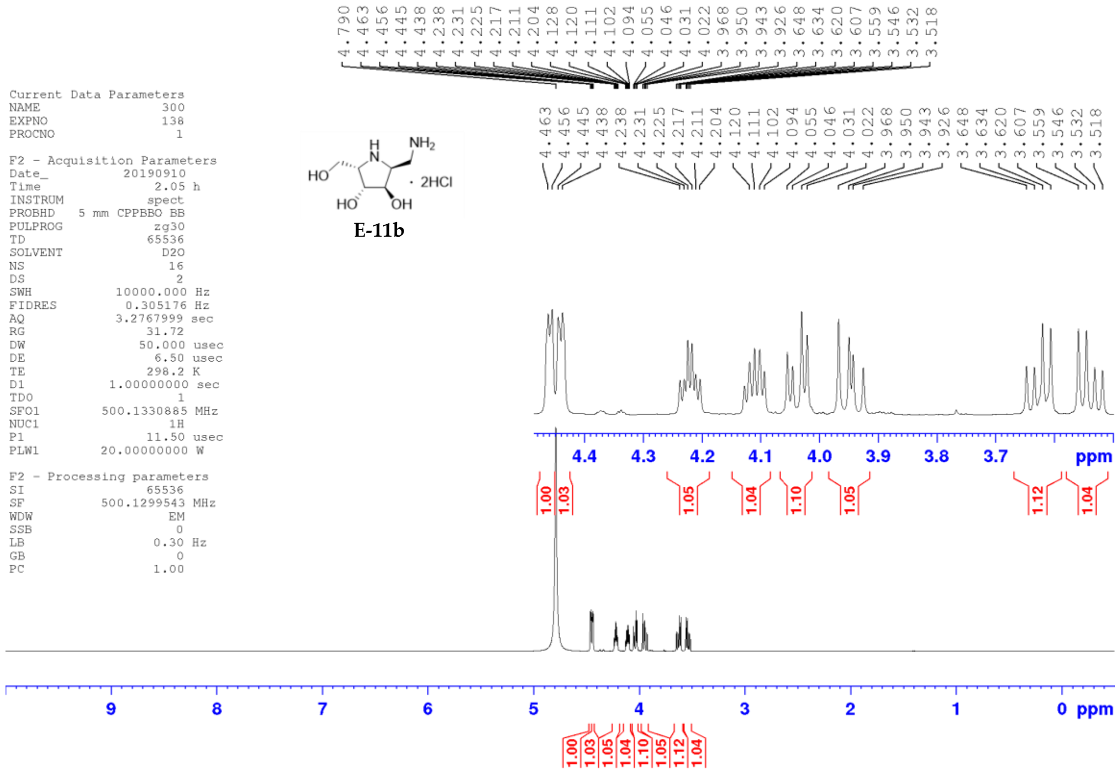

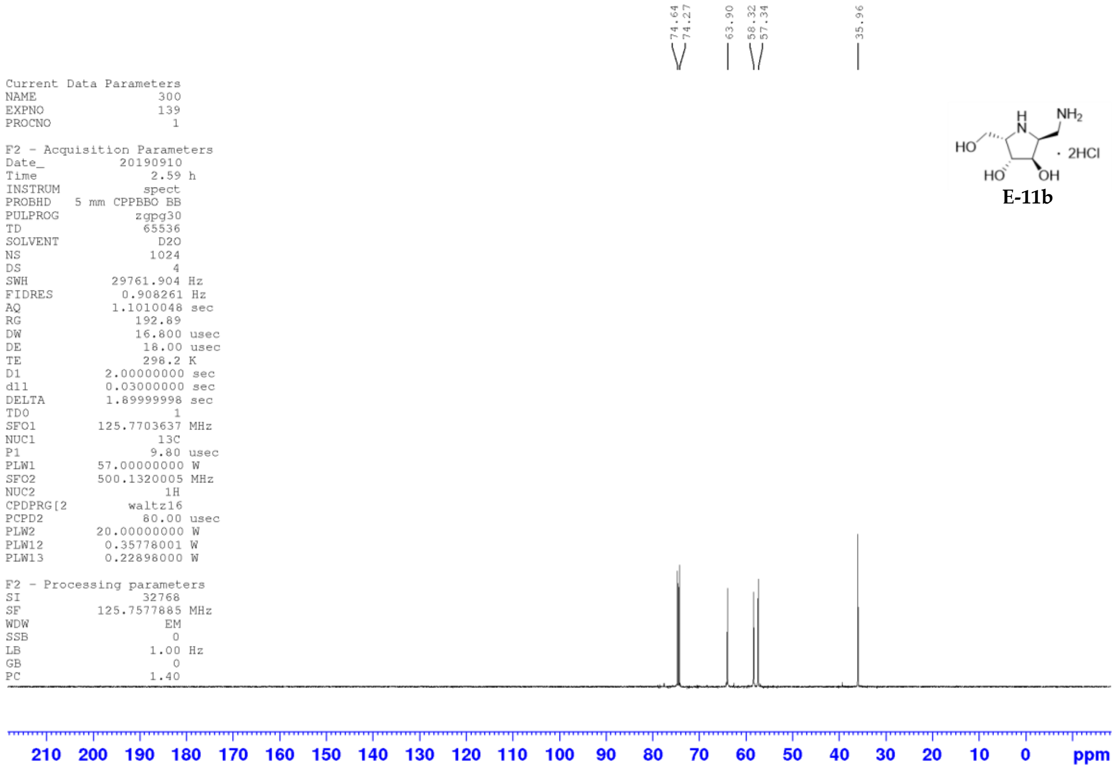

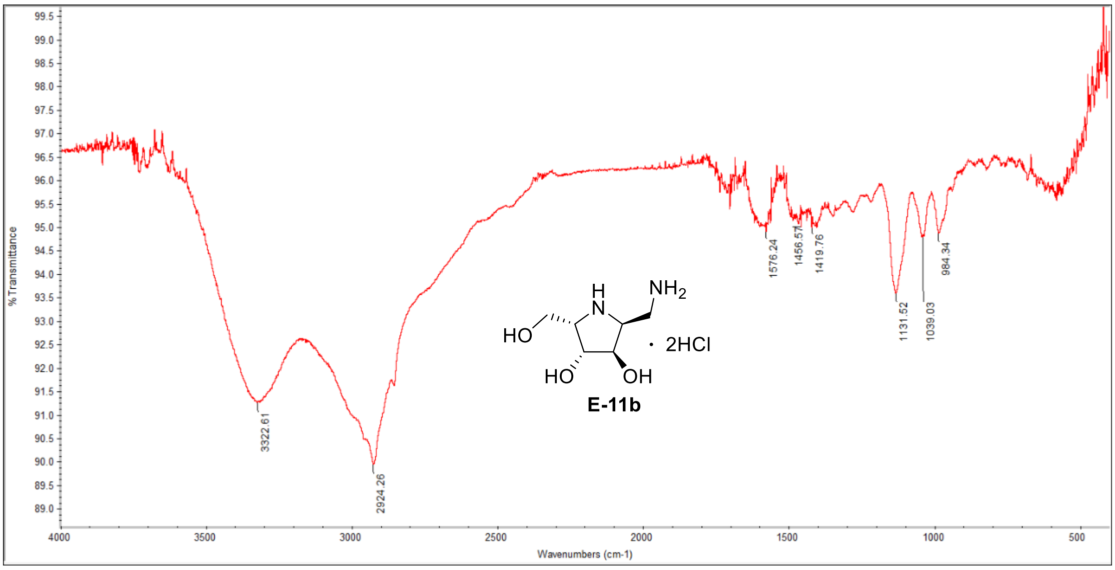

Compound **F-11a**:

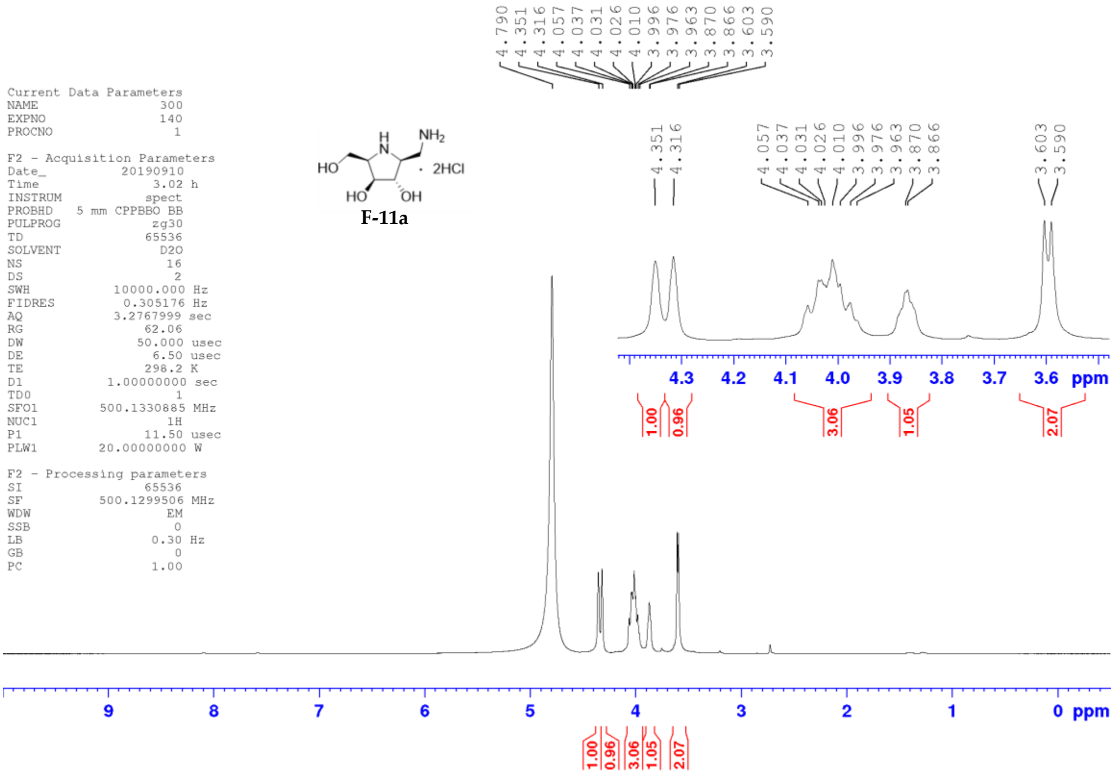

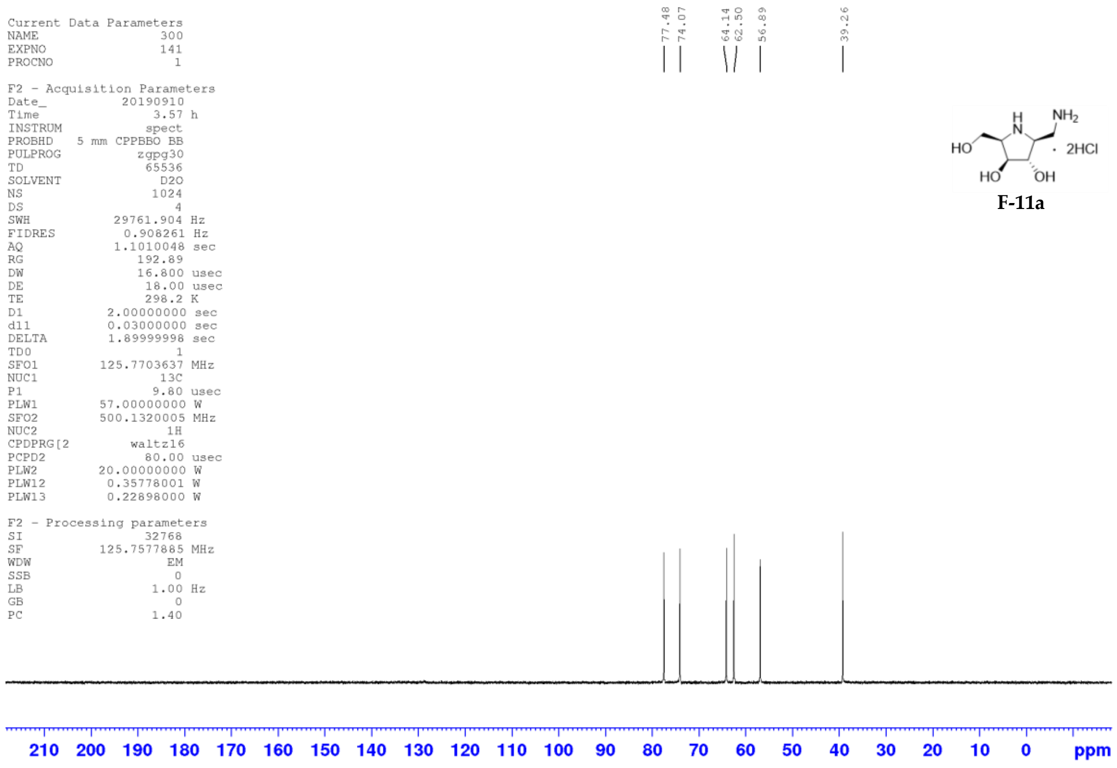

Current Data Parameters
NAME 300
EXPNO 141
PROCNO 1
F2 - Acquisition Parameters
Date_ 20190910
Time 3.57 h
INSTRUM spect
PROBHD 5 mm CPPBBO BB
PULPROG zgpg30
TD 65536
SOLVENT D2O
NS 1024
DS 4
SWH 29761.904 Hz
FIDRES 0.908261 Hz
AQ 1.1010048 sec
RG 192.89
DW 16.800 usec
DE 18.00 usec
TE 298.2 K
D1 2.00000000 sec
d11 0.03000000 sec
DELTA 1.89999998 sec
TD0 1
SFO1 125.7703637 MHz
NUC1 13C
P1 9.80 usec
PLW1 57.00000000 W
SFO2 500.1320005 MHz
NUC2 1H
CPDPRG[2 waltz16
PCPD2 80.00 usec
PLW2 20.00000000 W
PLW12 0.35778001 W
PLW13 0.22898000 W
F2 - Processing parameters
SI 32768
SF 125.7577885 MHz
WDW EM
SSB 0
LB 1.00 Hz
GB 0
PC 1.40
77.48
74.07
64.14
62.50
56.89
39.26
HO
H
N
NH2
· 2HCl
HO
OH
F-11a
210 200 190 180 170 160 150 140 130 120 110 100 90 80 70 60 50 40 30 20 10 0 ppm

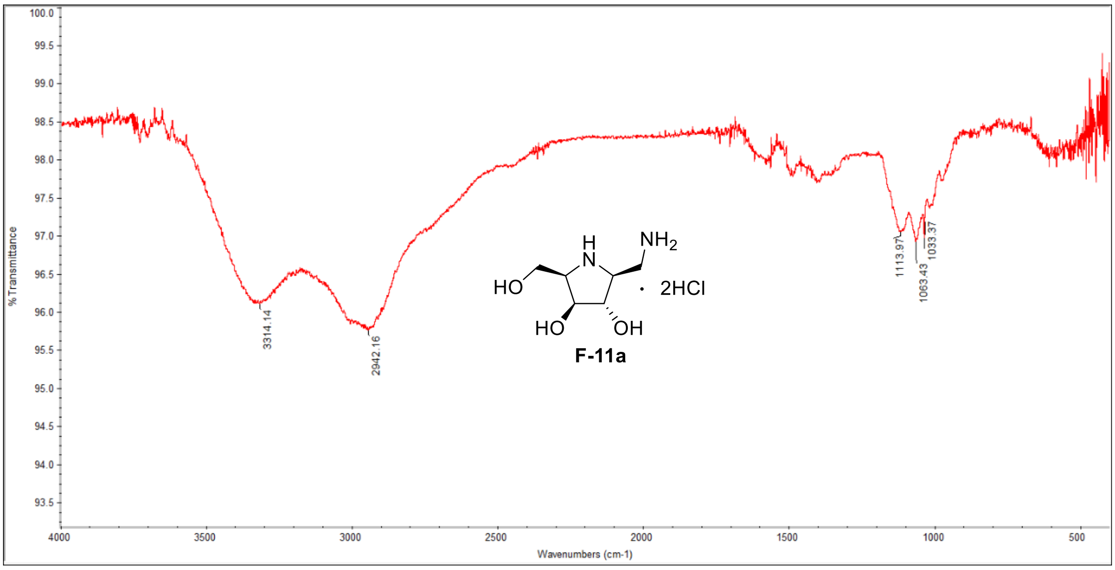

% Transmittance
Wavenumbers (cm-1)
3314.14
2942.16
1113.97
1063.43
1033.37
HO
H
N
NH2
· 2HCl
HO
OH
F-11a

Compound **F-11b**:

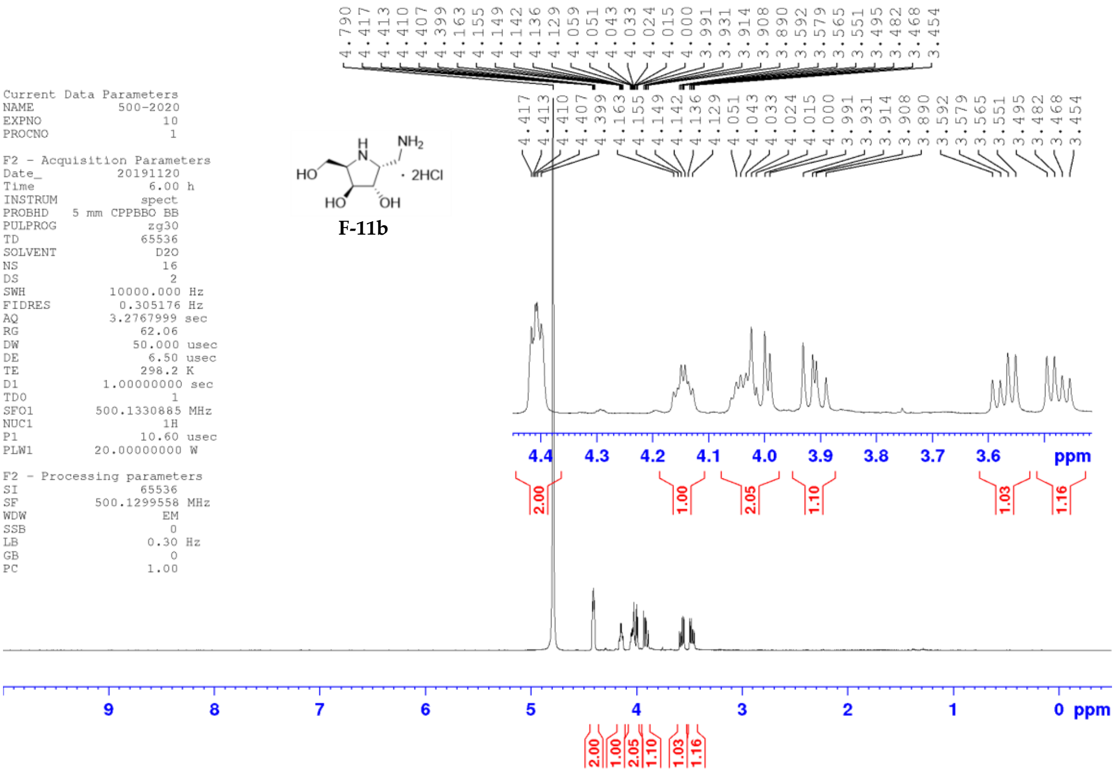

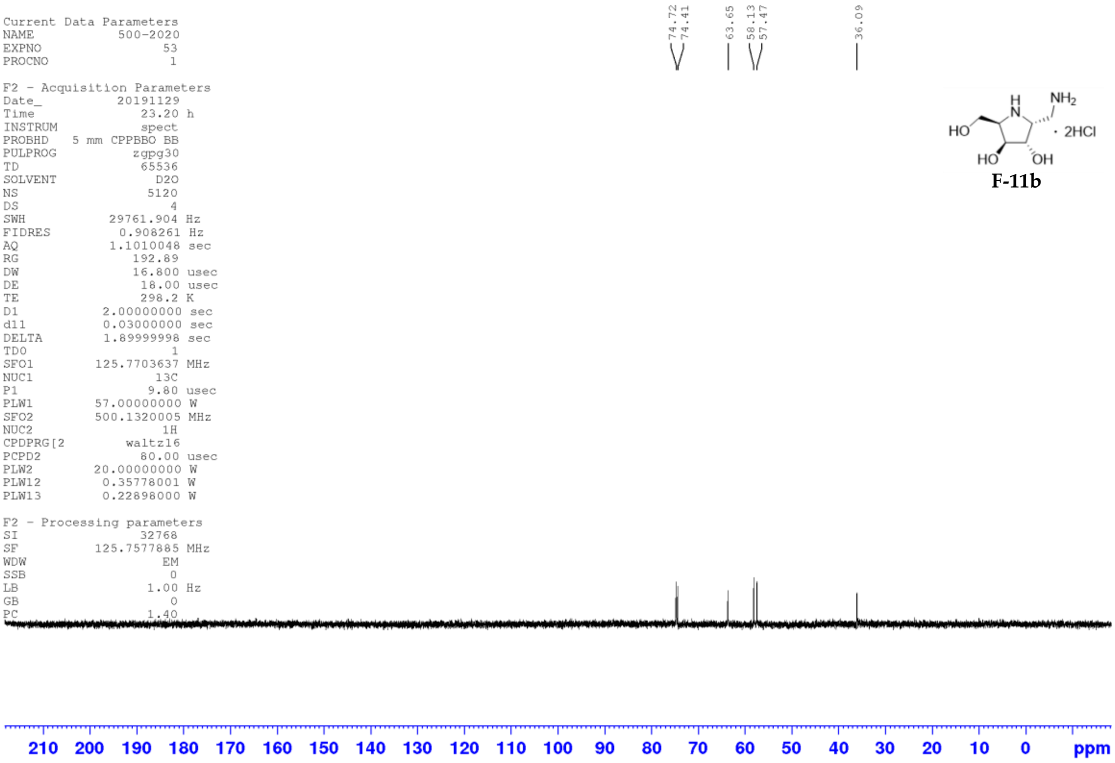

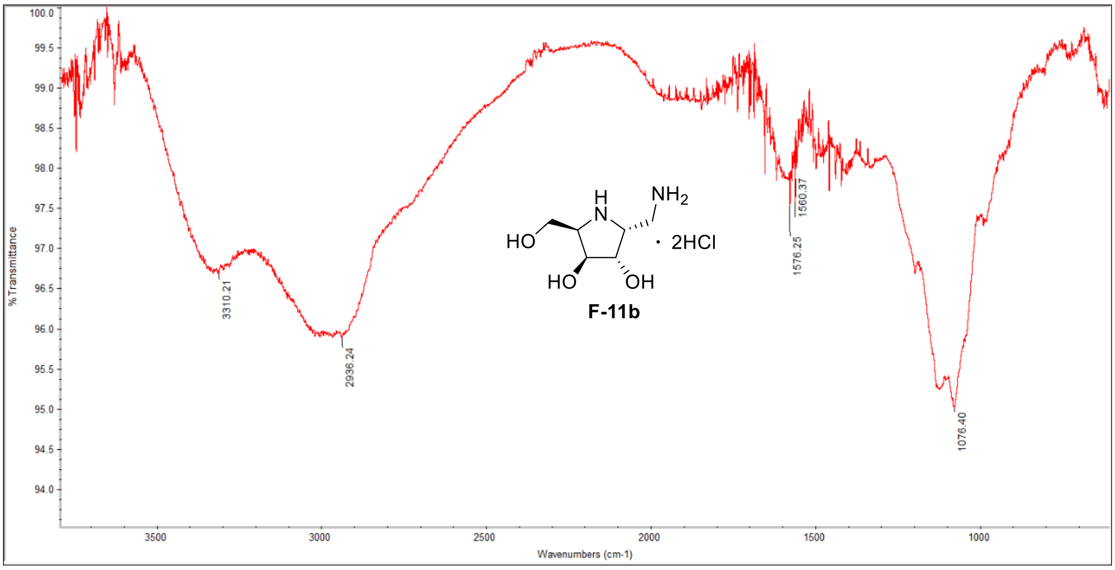

Compound **G-11**:

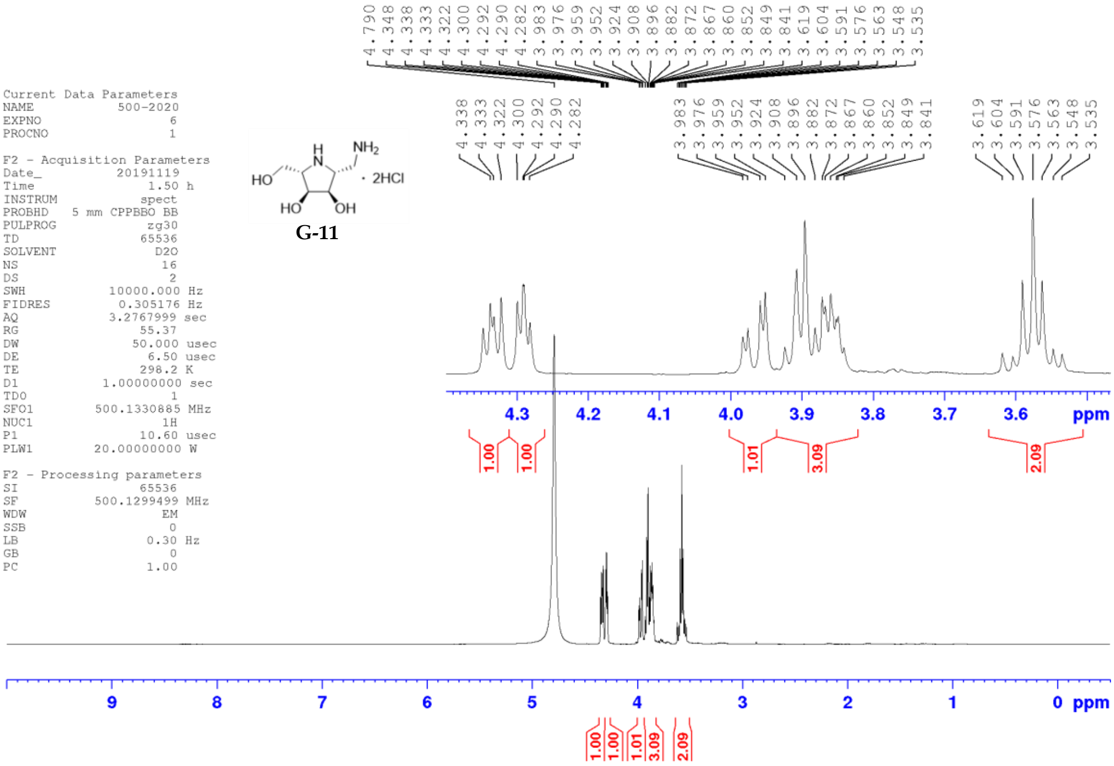

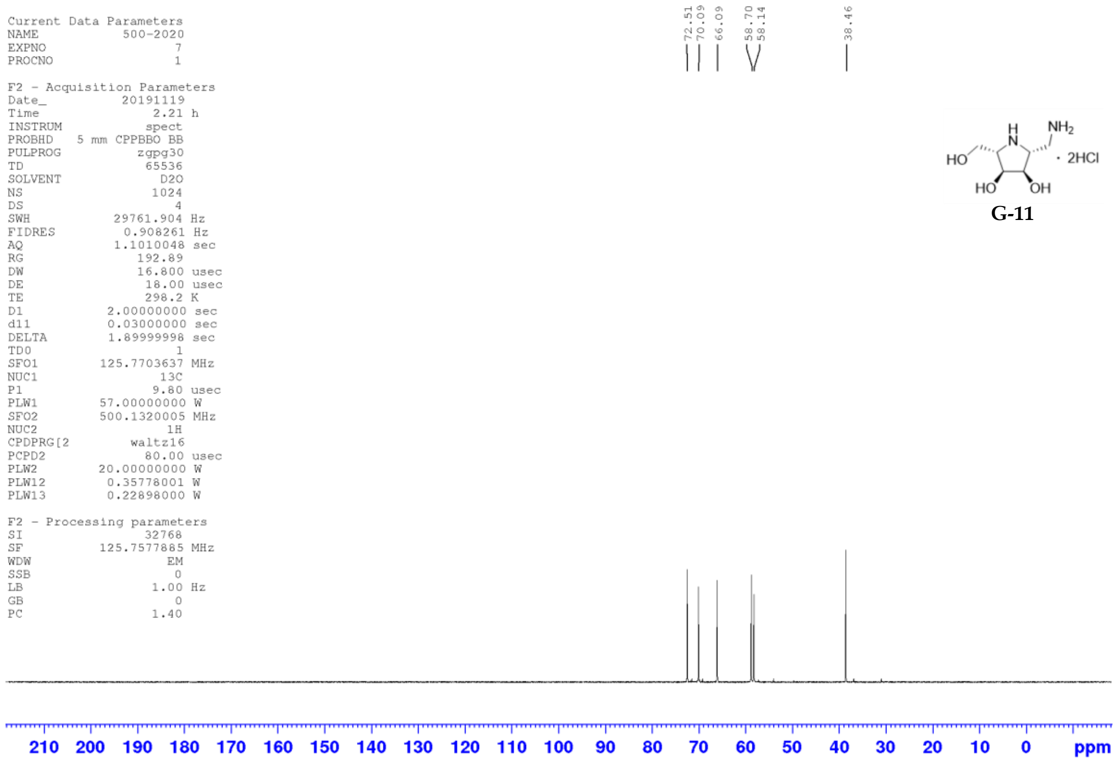
Current Data Parameters
NAME 500-2020
EXPNO 7
PROCNO 1
F2 - Acquisition Parameters
Date_ 20191119
Time 2.21 h
INSTRUM spect
PROBHD 5 mm CPPBBO BB
PULPROG zgpg30
TD 65536
SOLVENT D2O
NS 1024
DS 4
SWH 29761.904 Hz
FIDRES 0.908261 Hz
AQ 1.1010048 sec
RG 192.89
DW 16.800 usec
DE 18.00 usec
TE 298.2 K
D1 2.00000000 sec
d11 0.03000000 sec
DELTA 1.89999998 sec
TD0 1
SFO1 125.7703637 MHz
NUC1 13C
P1 9.80 usec
PLW1 57.00000000 W
SFO2 500.1320005 MHz
NUC2 1H
CPDPRG[2 waltz16
PCPD2 80.00 usec
PLW2 20.00000000 W
PLW12 0.35778001 W
PLW13 0.22898000 W
F2 - Processing parameters
SI 32768
SF 125.7577885 MHz
WDW EM
SSB 0
LB 1.00 Hz
GB 0
PC 1.40
72.51
70.09
66.09
58.70
58.14
38.46
HO
H
N
NH2
· 2HCl
HO
OH
G-11
210 200 190 180 170 160 150 140 130 120 110 100 90 80 70 60 50 40 30 20 10 0 ppm

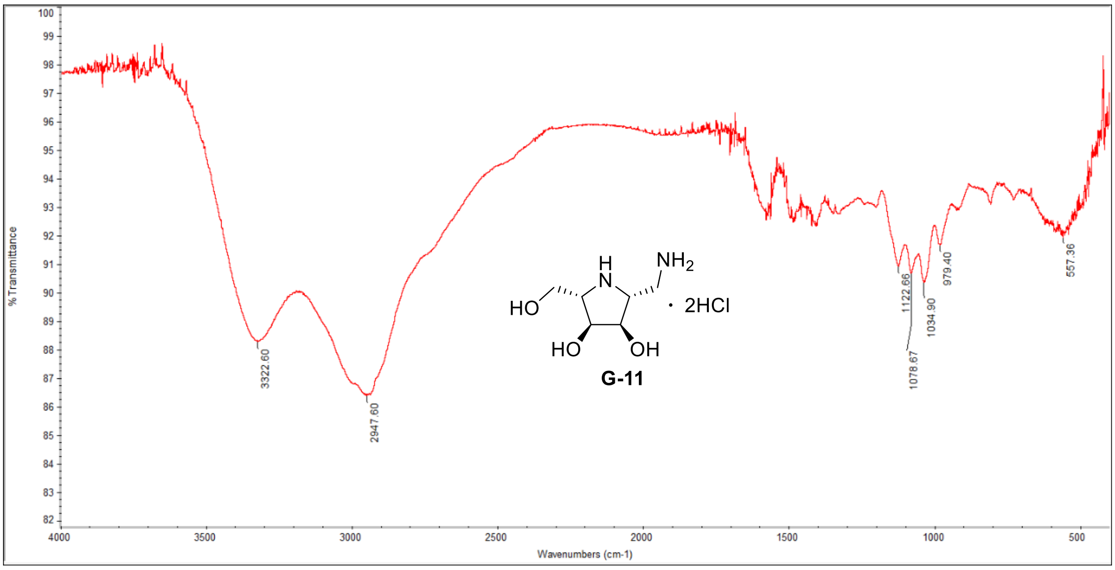
% Transmittance
100 99 98 97 96 95 94 93 92 91 90 89 88 87 86 85 84 83 82
3322.60
2947.60
1122.66
1078.67
1034.90
979.40
557.36
HO
H
N
NH2
· 2HCl
HO
OH
G-11
4000 3500 3000 2500 2000 1500 1000 500
Wavenumbers (cm-1)

Compound **H-11**:

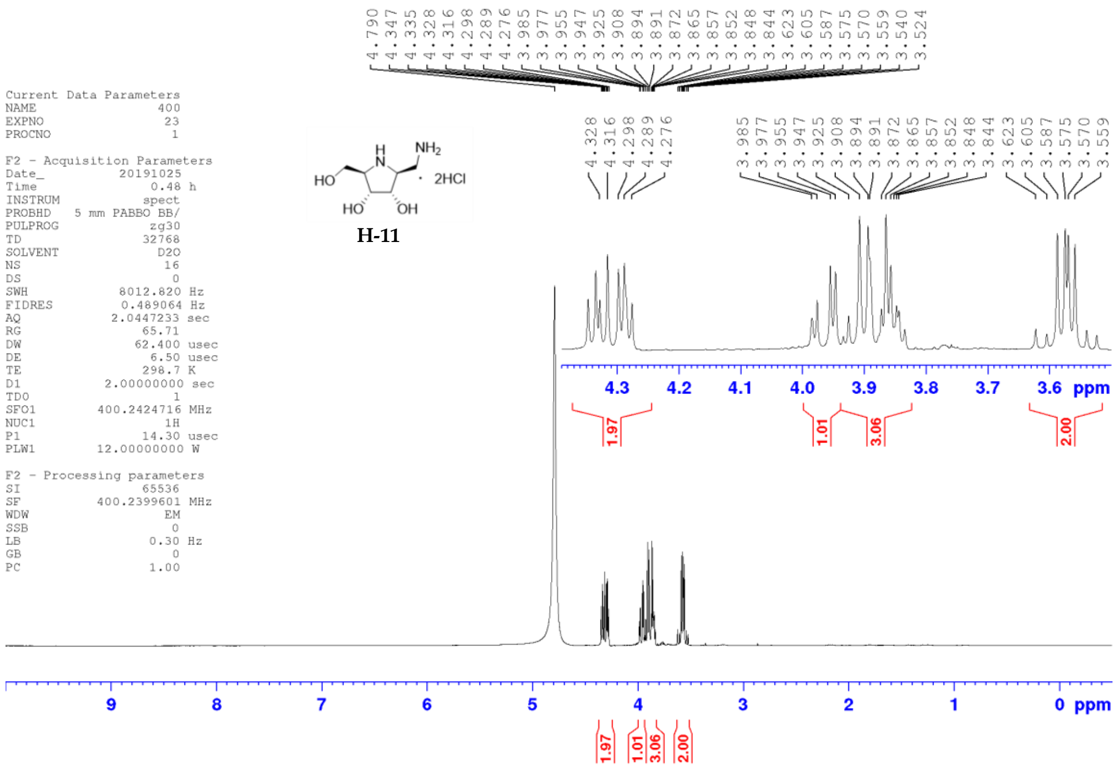

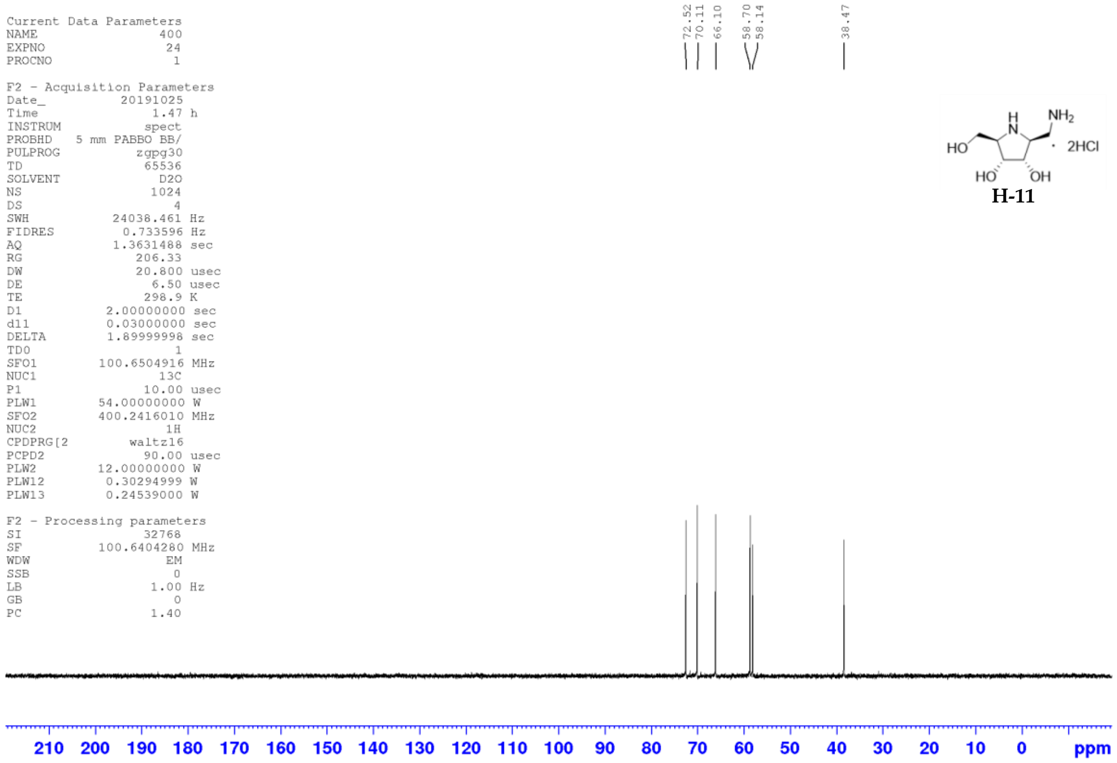

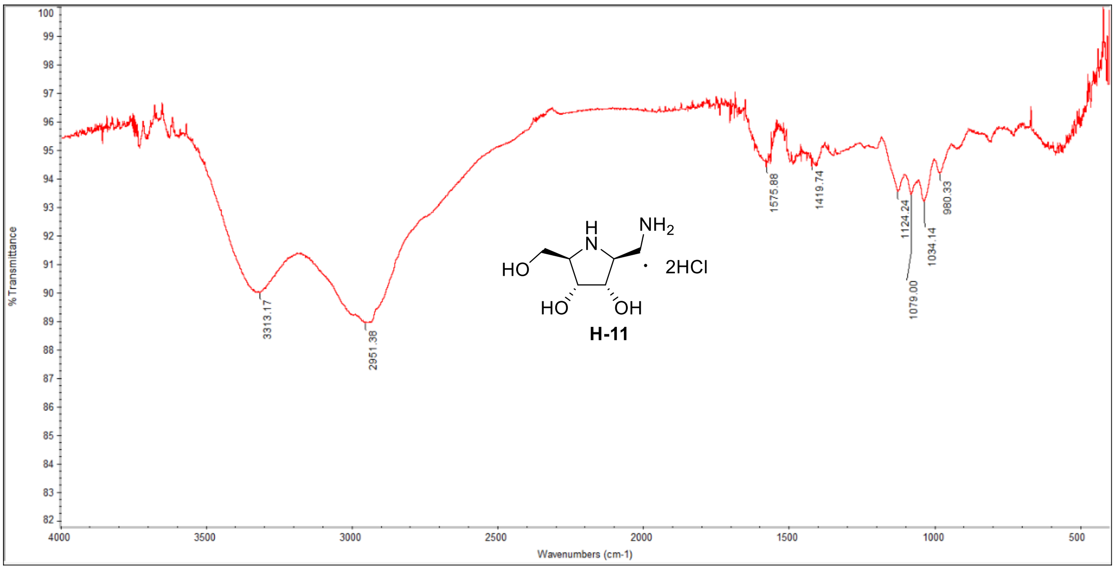

% Transmittance
Wavenumbers (cm-1)
3313.17
2951.38
1575.88
1419.74
1124.24
1079.00
1034.14
980.33
HO
H
N
NH2
· 2HCl
HO
OH
H-11

2. X-Ray Crystallographic data for compound D-2, F-2b and H-2

1) X-Ray Crystallographic Data for compound D-2

Structure deposited at the Cambridge Crystallographic Data Centre (**CCDC 1982117**)

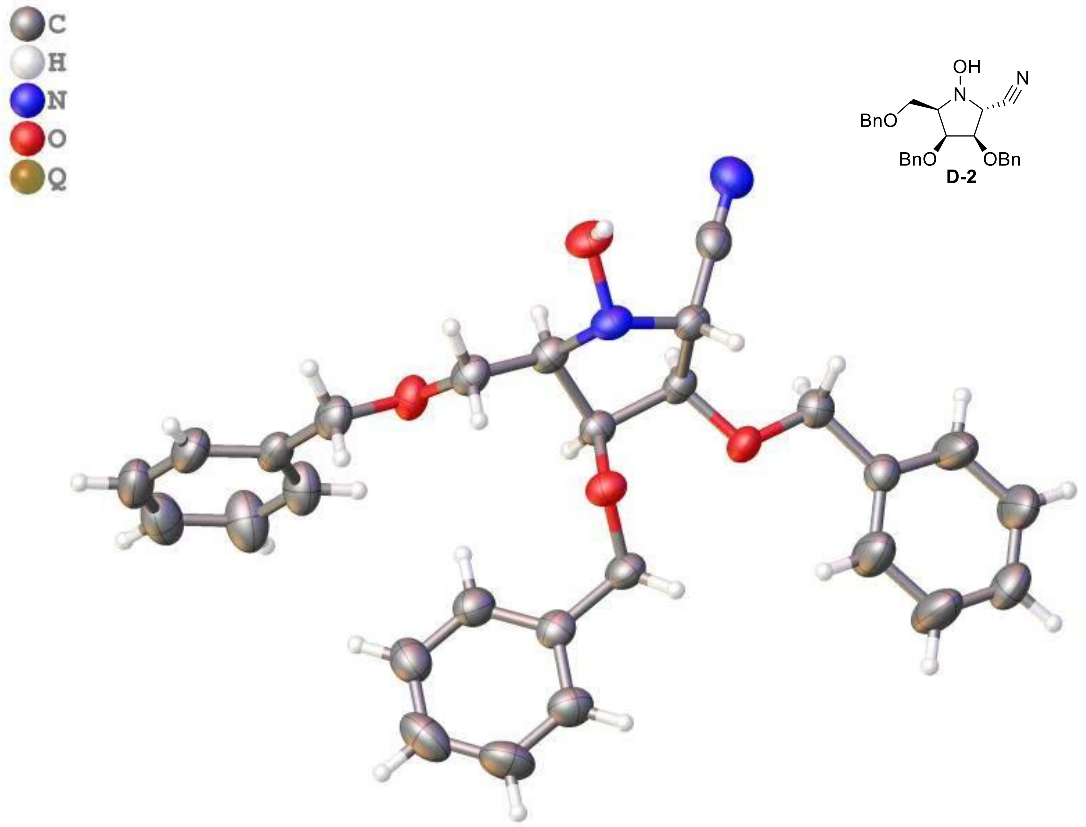

Fig1. X-Ray ellipsoid plots of **D-2**

Table 1 Crystal data and structure refinement for D-2.

Identification code	TX2155
Empirical formula	$C_{27}H_{28}N_2O_4$
Formula weight	444.51
Temperature/K	170.00(10)
Crystal system	monoclinic
Space group	$P2_1$
a/Å	14.5041(7)
b/Å	4.9247(3)
c/Å	16.1593(6)
α/°	90
β/°	91.658(4)
γ/°	90
Volume/Å^3	1153.75(10)
Z	2
ρ_{calc}g/cm^3	1.280

μ/mm^{-1}	0.695
F(000)	472.0
Crystal size/mm^3	0.100 × 0.050 × 0.020
Radiation	CuKα (λ = 1.54184)
2Θ range for data collection/°	5.472 to 151.108
Index ranges	$-16 \leq h \leq 18, -5 \leq k \leq 6, -19 \leq l \leq 20$
Reflections collected	13817
Independent reflections	4509 [R_{int} = 0.0626, R_{sigma} = 0.0589]
Data/restraints/parameters	4509/1/299
Goodness-of-fit on F^2	1.043
Final R indexes [I>=2σ (I)]	R_1 = 0.0592, wR_2 = 0.1402
Final R indexes [all data]	R_1 = 0.0726, wR_2 = 0.1482
Largest diff. peak/hole / e $Å^{-3}$	0.22/-0.21
Flack parameter	-0.06(18)

Table 2 Fractional Atomic Coordinates (×10^4) and Equivalent Isotropic Displacement Parameters ($Å^2$×10^3) for D-2. U_{eq} is defined as 1/3 of of the trace of the orthogonalised U_{IJ} tensor.

Atom	*x*	*y*	*z*	U(eq)
O1	7217.2(16)	6566(5)	2195.7(15)	40.4(6)
O2	7250.5(16)	2526(5)	1007.6(14)	40.2(6)
O3	6123.6(18)	5455(6)	4098.8(14)	45.7(6)
O4	4386.9(16)	5696(7)	2015.0(18)	53.5(7)
N1	5367.1(18)	5804(7)	1918.8(18)	41.4(7)
N2	4354(2)	432(8)	836(2)	52.8(9)
C1	5636(2)	3948(8)	1236(2)	38.7(8)
C2	6522(2)	2506(8)	1568(2)	36.6(8)
C3	6775(2)	4086(8)	2358(2)	35.8(8)
C4	5816(2)	4731(8)	2675(2)	38.6(8)
C5	5769(3)	6725(9)	3372(2)	44.1(8)
C6	4911(3)	2006(9)	1015(2)	43.3(9)
C7	7101(2)	609(9)	357(2)	44.7(9)
C8	7914(2)	555(9)	-204(2)	41.4(8)
C9	8677(3)	2149(12)	-92(3)	75.8(16)
C10	9407(4)	2006(15)	-631(4)	89.0(19)
C11	9360(3)	265(11)	-1290(3)	61.2(12)
C12	8625(3)	-1328(15)	-1395(3)	82.8(18)

C13	7899(3)	-1203(15)	-856(3)	84.7(19)
C14	8205(2)	6489(9)	2144(2)	46.1(9)
C15	8650(2)	8122(8)	2831(2)	41.3(8)
C16	8355(3)	7848(10)	3641(3)	51.6(10)
C17	8775(3)	9265(11)	4275(3)	58.4(12)
C18	9491(4)	11006(12)	4129(3)	71.6(14)
C19	9799(3)	11287(12)	3324(3)	70.3(14)
C20	9376(3)	9880(9)	2689(3)	53.8(11)
C21	6102(3)	7230(9)	4795(2)	51.6(10)
C22	6592(3)	5996(9)	5526(2)	47.3(9)
C23	6403(3)	6939(11)	6319(3)	61.4(12)
C24	6859(3)	5932(12)	7001(3)	67.9(14)
C25	7506(4)	3942(12)	6931(3)	68.0(13)
C26	7708(4)	2979(13)	6144(3)	76.9(15)
C27	7256(4)	3997(10)	5460(3)	60.5(12)

Table 3 Anisotropic Displacement Parameters ($Å^2 \times 10^3$) for D-2. The Anisotropic displacement factor exponent takes the form: $-2\pi^2[h^2a^{*2}U_{11}+2hka^*b^*U_{12}+\ldots]$.

Atom	U_{11}	U_{22}	U_{33}	U_{23}	U_{13}	U_{12}
O1	33.6(12)	37.9(15)	50.0(13)	0.2(11)	5.8(10)	1.5(11)
O2	38.1(12)	44.7(15)	38.3(12)	-5.1(10)	11.6(9)	-3.6(11)
O3	55.3(15)	44.1(15)	38.1(12)	-3.3(11)	11.0(10)	3.5(13)
O4	31.6(12)	63(2)	66.3(17)	-0.2(14)	6.8(11)	6.7(13)
N1	29.3(13)	44.3(18)	50.9(16)	2.2(14)	4.5(11)	5.1(14)
N2	47.9(18)	58(2)	52.2(18)	-4.1(18)	-4.8(14)	3.6(19)
C1	35.6(17)	42(2)	38.2(18)	3.3(16)	4.9(13)	-0.5(16)
C2	36.7(17)	37(2)	36.7(17)	2.4(14)	10.0(13)	-2.1(15)
C3	35.1(16)	35(2)	37.1(17)	2.6(14)	3.1(13)	1.2(15)
C4	36.5(17)	36(2)	43.6(18)	1.0(14)	8.1(14)	2.1(15)
C5	44.0(19)	40(2)	49(2)	-3.3(17)	10.0(15)	4.7(17)
C6	40.7(19)	53(2)	35.6(18)	-0.3(16)	-1.1(14)	8.1(19)
C7	43.7(18)	50(2)	40.6(18)	-2.8(17)	3.0(14)	-1.8(19)
C8	39.4(18)	46(2)	39.2(17)	-1.7(16)	3.9(13)	4.7(17)
C9	70(3)	85(4)	74(3)	-32(3)	30(2)	-24(3)
C10	71(3)	101(5)	98(4)	-32(4)	43(3)	-33(3)
C11	57(2)	70(3)	58(2)	-5(2)	22.7(19)	5(2)
C12	61(3)	115(5)	73(3)	-48(3)	24(2)	-12(3)
C13	53(3)	118(5)	85(3)	-51(4)	27(2)	-31(3)
C14	34.6(17)	53(3)	51(2)	-3.3(18)	8.4(14)	1.4(17)

C15	37.5(18)	41(2)	46(2)	0.1(16)	3.2(14)	8.3(16)
C16	43(2)	61(3)	51(2)	2.6(19)	3.0(16)	-1.2(19)
C17	54(2)	68(3)	52(2)	-11(2)	-5.0(19)	7(2)
C18	74(3)	65(3)	74(3)	-21(3)	-24(3)	1(3)
C19	59(3)	65(3)	86(3)	-4(3)	-11(2)	-22(3)
C20	48(2)	54(3)	59(2)	4.4(19)	3.8(18)	-6(2)
C21	56(2)	51(3)	48(2)	-13.7(18)	14.2(17)	-1(2)
C22	53(2)	45(2)	45.2(19)	-9.1(17)	16.3(15)	-12.9(19)
C23	58(2)	74(3)	53(2)	-13(2)	19.4(19)	-4(2)
C24	79(3)	82(4)	44(2)	-10(2)	15(2)	-18(3)
C25	89(3)	67(3)	48(2)	2(2)	-6(2)	-11(3)
C26	104(4)	71(4)	55(3)	-8(2)	-3(3)	18(3)
C27	82(3)	54(3)	46(2)	-8(2)	8(2)	5(2)

Table 4 Bond Lengths for D-2.

Atom	Atom	Length/Å	Atom	Atom	Length/Å
O1	C3	1.407(4)	C9	C10	1.392(7)
O1	C14	1.438(4)	C10	C11	1.368(7)
O2	C2	1.411(4)	C11	C12	1.330(8)
O2	C7	1.425(5)	C12	C13	1.388(7)
O3	C5	1.414(5)	C14	C15	1.501(5)
O3	C21	1.426(5)	C15	C16	1.395(6)
O4	N1	1.436(4)	C15	C20	1.388(6)
N1	C1	1.493(5)	C16	C17	1.368(6)
N1	C4	1.466(5)	C17	C18	1.372(8)
N2	C6	1.150(5)	C18	C19	1.395(8)
C1	C2	1.550(5)	C19	C20	1.368(7)
C1	C6	1.458(6)	C21	C22	1.490(6)
C2	C3	1.531(5)	C22	C23	1.399(6)
C3	C4	1.530(5)	C22	C27	1.384(6)
C4	C5	1.497(5)	C23	C24	1.362(7)
C7	C8	1.507(5)	C24	C25	1.364(8)
C8	C9	1.364(6)	C25	C26	1.396(7)
C8	C13	1.363(6)	C26	C27	1.364(7)

Table 5 Bond Angles for D-2.

Atom	Atom	Atom	Angle/°	Atom	Atom	Atom	Angle/°

C3	O1	C14	116.6(3)	C8	C9	C10	121.1(5)
C2	O2	C7	111.6(3)	C11	C10	C9	119.8(5)
C5	O3	C21	111.6(3)	C12	C11	C10	119.4(4)
O4	N1	C1	109.7(3)	C11	C12	C13	120.9(5)
O4	N1	C4	108.3(3)	C8	C13	C12	121.2(5)
C4	N1	C1	106.0(3)	O1	C14	C15	110.6(3)
N1	C1	C2	104.9(3)	C16	C15	C14	120.3(4)
C6	C1	N1	112.4(3)	C20	C15	C14	121.6(3)
C6	C1	C2	111.7(3)	C20	C15	C16	118.0(4)
O2	C2	C1	113.8(3)	C17	C16	C15	120.8(4)
O2	C2	C3	111.4(3)	C16	C17	C18	120.9(4)
C3	C2	C1	103.4(3)	C17	C18	C19	119.1(4)
O1	C3	C2	112.7(3)	C20	C19	C18	120.1(5)
O1	C3	C4	107.8(3)	C19	C20	C15	121.2(4)
C4	C3	C2	100.7(3)	O3	C21	C22	110.7(3)
N1	C4	C3	100.5(3)	C23	C22	C21	119.3(4)
N1	C4	C5	111.2(3)	C27	C22	C21	123.2(3)
C5	C4	C3	116.7(3)	C27	C22	C23	117.5(4)
O3	C5	C4	108.1(3)	C24	C23	C22	121.1(5)
N2	C6	C1	178.4(4)	C23	C24	C25	120.9(4)
O2	C7	C8	110.5(3)	C24	C25	C26	118.9(4)
C9	C8	C7	123.7(4)	C27	C26	C25	120.3(5)
C13	C8	C7	118.7(4)	C26	C27	C22	121.3(4)
C13	C8	C9	117.6(4)				

Table 6 Hydrogen Atom Coordinates (Å×10^4) and Isotropic Displacement Parameters (Å^2×10^3) for D-2.

Atom	*x*	*y*	*z*	U(eq)
H4	4141	6945	1755	80
H1	5783	5028	748	46
H2	6375	625	1712	44
H3	7135	2974	2753	43
H4A	5512	3039	2833	46
H5A	6130	8326	3249	53
H5B	5136	7281	3447	53
H7A	6548	1093	38	54
H7B	7011	-1182	590	54
H9	8709	3351	352	91
H10	9925	3091	-542	107

H11	9837	195	-1661	73
H12	8598	-2541	-1836	99
H13	7393	-2339	-940	102
H14A	8384	7215	1615	55
H14B	8416	4623	2181	55
H16	7868	6690	3751	62
H17	8573	9045	4812	70
H18	9768	11985	4562	86
H19	10291	12432	3218	84
H20	9579	10106	2153	65
H21A	5466	7598	4929	62
H21B	6392	8941	4659	62
H23	5957	8276	6383	74
H24	6728	6611	7521	81
H25	7808	3239	7400	82
H26	8153	1637	6086	92
H27	7398	3335	4940	73

2) X-Ray Crystallographic Data for compound F-2b

Structure deposited at the Cambridge Crystallographic Data Centre (**CCDC 1982119**)

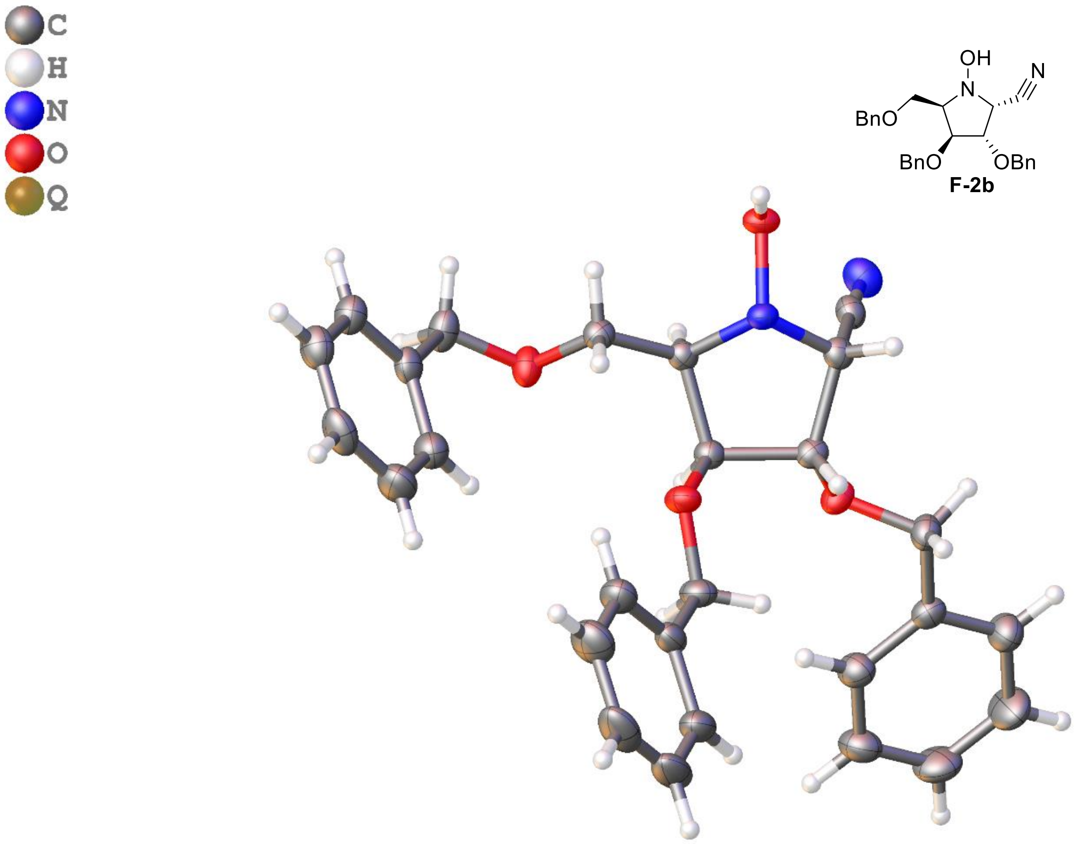

Fig2. X-Ray ellipsoid plots of **F-2b**

Table 1 Crystal data and structure refinement for F-2b.

Identification code	tx1085
Empirical formula	$C_{27}H_{28}N_2O_4$
Formula weight	444.51
Temperature/K	169.99(13)
Crystal system	monoclinic
Space group	$P2_1$
a/Å	12.55905(15)
b/Å	4.72901(5)
c/Å	19.9900(2)
α/°	90
β/°	102.7817(11)
γ/°	90
Volume/Å^3	1157.82(2)
Z	2
ρ_{calc}g/cm^3	1.275

μ/mm^{-1}	0.692
F(000)	472.0
Crystal size/mm^3	0.35 × 0.12 × 0.08
Radiation	CuKα (λ = 1.54184)
2Θ range for data collection/°	4.532 to 150.762
Index ranges	$-15 \leq h \leq 15$, $-5 \leq k \leq 5$, $-25 \leq l \leq 24$
Reflections collected	22252
Independent reflections	4661 [R_{int} = 0.0884, R_{sigma} = 0.0442]
Data/restraints/parameters	4661/1/299
Goodness-of-fit on F^2	1.063
Final R indexes [I>=2σ (I)]	R_1 = 0.0384, wR_2 = 0.1015
Final R indexes [all data]	R_1 = 0.0414, wR_2 = 0.1050
Largest diff. peak/hole / e $Å^{-3}$	0.14/-0.24
Flack parameter	0.18(13)

Table 2 Fractional Atomic Coordinates (×10^4) and Equivalent Isotropic Displacement Parameters ($Å^2$×10^3) for F-2b. U_{eq} is defined as 1/3 of of the trace of the orthogonalised U_{IJ} tensor.

**Atom**	***x***	***y***	***z***	**U(eq)**
O1	5234.5(12)	3948(3)	4706.4(6)	25.7(3)
O2	2336.4(12)	7858(3)	3727.0(8)	30.4(3)
O3	3254.0(11)	4102(3)	2596.7(7)	27.3(3)
O4	5696.7(12)	7201(3)	2584.3(7)	27.5(3)
N1	4875.9(13)	3747(3)	3969.8(8)	22.1(3)
N2	7019.4(16)	8562(4)	4276.9(10)	35.8(4)
C1	5803.1(16)	4401(4)	3659.7(10)	23.8(4)
C2	5209.6(16)	5073(4)	2910.5(10)	23.6(4)
C3	4047.6(16)	6016(4)	2956.1(9)	23.1(4)
C4	4062.6(16)	5979(4)	3723.5(9)	22.1(4)
C5	2989.5(16)	5400(4)	3917(1)	24.4(4)
C6	6472.1(16)	6804(4)	3998(1)	25.6(4)
C7	1433.4(18)	8016(5)	4044.5(12)	32.9(5)
C8	538.0(17)	5912(4)	3763.2(11)	27.8(4)
C9	-155.2(18)	4951(5)	4168.5(12)	33.1(5)
C10	-992.4(18)	3067(5)	3909.2(13)	36.9(5)
C11	-1147.2(19)	2133(6)	3243.0(14)	41.6(6)
C12	-462(2)	3096(6)	2832.8(13)	43.9(6)
C13	374.5(19)	4971(6)	3091.8(12)	36.2(5)
C14	3142.6(18)	4302(5)	1875.4(10)	30.8(4)
C15	2306.6(17)	2214(5)	1518.7(10)	29.6(4)

C16	1590.3(19)	857(5)	1845.7(12)	36.2(5)
C17	816(2)	-987(7)	1484.8(15)	48.3(6)
C18	758(2)	-1516(7)	795.9(15)	52.1(7)
C19	1471(2)	-177(7)	469.4(13)	49.9(7)
C20	2246(2)	1685(6)	822.3(11)	39.1(5)
C21	6543.1(18)	6242(5)	2265.8(11)	30.8(5)
C22	6573.5(17)	8132(4)	1666.9(10)	27.5(4)
C23	5625.7(19)	9285(5)	1270.4(11)	34.4(5)
C24	5663(2)	10979(6)	707.9(12)	41.0(6)
C25	6653(2)	11500(7)	536.0(13)	49.3(7)
C26	7593(2)	10367(8)	923.9(15)	51.7(7)
C27	7559.2(19)	8680(6)	1489.5(12)	38.7(5)

Table 3 Anisotropic Displacement Parameters ($Å^2 \times 10^3$) for F-2b. The Anisotropic displacement factor exponent takes the form: $-2\pi^2[h^2a^{*2}U_{11}+2hka^*b^*U_{12}+\ldots]$.

Atom	U_{11}	U_{22}	U_{33}	U_{23}	U_{13}	U_{12}
O1	33.7(8)	23.8(6)	18.4(6)	1.8(5)	3.4(5)	-0.9(6)
O2	26.6(8)	26.1(7)	41.7(8)	4.6(6)	14.3(6)	2.5(6)
O3	28.5(7)	32.9(7)	19.4(6)	-1.1(5)	2.7(5)	-4.6(6)
O4	32.4(8)	24.7(7)	28.8(7)	4.2(6)	14.2(6)	0.4(6)
N1	25.2(8)	23.0(7)	17.9(7)	0.6(6)	4.6(6)	0.2(6)
N2	31.8(10)	33.4(10)	39.8(10)	2.1(8)	2.9(8)	-3.9(8)
C1	25.4(10)	19.3(9)	26.5(9)	2.1(7)	5.8(7)	2.2(7)
C2	27.6(10)	20.1(8)	24.0(9)	1.7(7)	7.3(7)	-0.3(7)
C3	23.9(10)	23.1(9)	22.1(9)	1.0(7)	4.8(7)	0.0(7)
C4	22.6(9)	20.9(8)	22.1(9)	-0.9(7)	3.2(7)	-0.2(7)
C5	26.5(10)	22.4(9)	24.6(9)	1.2(7)	6.4(7)	0.3(7)
C6	21.9(9)	25.8(10)	29.2(9)	5.0(8)	5.4(8)	2.2(8)
C7	28.4(11)	27.4(10)	46.1(12)	-4.7(9)	15.1(9)	-0.2(9)
C8	23.8(10)	24.5(9)	35.5(10)	1.4(8)	7.5(8)	5.6(8)
C9	30.1(11)	32.8(10)	38.4(11)	0.2(9)	11.7(9)	2.0(9)
C10	27.5(11)	35.9(11)	48.6(13)	3.5(10)	11.1(10)	-0.6(9)
C11	23.6(11)	39.0(12)	57.3(15)	-4.9(11)	-1.4(10)	-1.3(10)
C12	34.6(12)	56.0(15)	37.4(12)	-7.8(11)	-0.1(10)	2.3(11)
C13	31.6(11)	43.6(12)	33.8(11)	2.4(10)	7.8(9)	4.3(10)
C14	33.5(11)	37.8(11)	20.2(9)	1.6(8)	3.9(8)	-0.5(9)
C15	28.1(10)	33.5(11)	24.5(9)	-3.5(8)	-0.3(8)	8.2(9)
C16	32.1(11)	44.1(13)	30.3(10)	-5.4(9)	2.5(9)	0.1(10)

C17	36.6(13)	53.8(15)	48.9(14)	-7.1(13)	-2.4(11)	-5.4(12)
C18	37.6(14)	55.7(17)	51.7(15)	-21.7(13)	-14.3(12)	4.8(12)
C19	46.9(15)	63.5(18)	30.9(11)	-18.9(12)	-9.3(10)	19.1(13)
C20	39.2(13)	50.5(14)	25.5(10)	-2.9(10)	2.6(9)	11.7(11)
C21	28.1(11)	33.0(11)	33.7(10)	5.6(8)	11.8(9)	5.0(8)
C22	31.8(11)	27.5(9)	24.6(9)	-2.1(8)	9.0(8)	-1.7(8)
C23	33.3(11)	39.4(12)	30.9(10)	3.5(9)	7.9(9)	-2.7(10)
C24	42.3(14)	48.3(14)	29.9(11)	6.5(10)	2.6(10)	-1.1(11)
C25	60.3(17)	56.6(17)	33.8(12)	13.5(11)	16.5(12)	-2.4(13)
C26	42.2(14)	70.9(18)	47.8(14)	16.4(14)	22.4(12)	-3.5(14)
C27	31.8(11)	47.6(14)	38.4(12)	6.8(10)	11.4(9)	0.9(10)

Table 4 Bond Lengths for F-2b.

Atom	Atom	Length/Å	Atom	Atom	Length/Å
O1	N1	1.4444(19)	C9	C10	1.388(3)
O2	C5	1.425(2)	C10	C11	1.375(4)
O2	C7	1.419(2)	C11	C12	1.390(4)
O3	C3	1.418(2)	C12	C13	1.384(4)
O3	C14	1.421(2)	C14	C15	1.501(3)
O4	C2	1.410(2)	C15	C16	1.382(3)
O4	C21	1.428(2)	C15	C20	1.400(3)
N1	C1	1.468(2)	C16	C17	1.384(4)
N1	C4	1.475(2)	C17	C18	1.386(4)
N2	C6	1.143(3)	C18	C19	1.373(5)
C1	C2	1.551(3)	C19	C20	1.385(4)
C1	C6	1.484(3)	C21	C22	1.501(3)
C2	C3	1.548(3)	C22	C23	1.387(3)
C3	C4	1.530(2)	C22	C27	1.385(3)
C4	C5	1.507(3)	C23	C24	1.390(3)
C7	C8	1.514(3)	C24	C25	1.383(4)
C8	C9	1.390(3)	C25	C26	1.371(4)
C8	C13	1.386(3)	C26	C27	1.392(4)

Table 5 Bond Angles for F-2b.

Atom	Atom	Atom	Angle/°	Atom	Atom	Atom	Angle/°
C7	O2	C5	113.18(15)	C10	C9	C8	120.8(2)
C3	O3	C14	111.84(15)	C11	C10	C9	120.1(2)

C2	O4	C21	114.93(15)	C10	C11	C12	119.5(2)
O1	N1	C1	108.61(14)	C13	C12	C11	120.4(2)
O1	N1	C4	109.27(13)	C12	C13	C8	120.4(2)
C1	N1	C4	105.29(14)	O3	C14	C15	109.87(17)
N1	C1	C2	101.20(15)	C16	C15	C14	122.75(18)
N1	C1	C6	112.99(16)	C16	C15	C20	119.3(2)
C6	C1	C2	112.69(15)	C20	C15	C14	117.9(2)
O4	C2	C1	115.32(16)	C15	C16	C17	120.1(2)
O4	C2	C3	109.43(16)	C16	C17	C18	120.5(3)
C3	C2	C1	105.08(15)	C19	C18	C17	119.5(2)
O3	C3	C2	110.62(15)	C18	C19	C20	120.6(2)
O3	C3	C4	110.61(15)	C19	C20	C15	119.8(3)
C4	C3	C2	104.66(15)	O4	C21	C22	108.62(17)
N1	C4	C3	101.36(14)	C23	C22	C21	121.30(19)
N1	C4	C5	112.13(15)	C27	C22	C21	119.8(2)
C5	C4	C3	116.49(16)	C27	C22	C23	118.9(2)
O2	C5	C4	106.15(15)	C22	C23	C24	120.7(2)
N2	C6	C1	176.7(2)	C25	C24	C23	119.8(2)
O2	C7	C8	113.64(18)	C26	C25	C24	120.0(2)
C9	C8	C7	120.5(2)	C25	C26	C27	120.4(2)
C13	C8	C7	120.6(2)	C22	C27	C26	120.3(2)
C13	C8	C9	118.8(2)				

Table 6 Hydrogen Atom Coordinates (Å×10^4) and Isotropic Displacement Parameters (Å^2×10^3) for F-2b.

**Atom**	***x***	***y***	***z***	**U(eq)**
H1	5085.61	2476.68	4882.78	39
H1A	6262.95	2720.34	3669.19	29
H2	5155.8	3335.26	2637.85	28
H3	3900.23	7931.9	2772	28
H4	4350.27	7789.1	3924.49	27
H5A	2638.75	3754.43	3674.15	29
H5B	3101.59	5060.99	4406.14	29
H7A	1131.09	9910.42	3984.39	39
H7B	1688.73	7691.44	4532.81	39
H9	-56.76	5578.54	4618.97	40
H10	-1449.01	2436.18	4186.17	44
H11	-1705.95	865.85	3068.33	50
H12	-566.17	2477.46	2381.36	53

H13	829.06	5602.03	2813.54	43
H14A	3839.33	3917.62	1760.29	37
H14B	2919.51	6201.95	1723.13	37
H16	1628.39	1182.96	2309.19	43
H17	330.49	-1877.32	1706.39	58
H18	240.93	-2768.87	556.39	63
H19	1432.07	-524.6	6.67	60
H20	2725.81	2581.59	597.22	47
H21A	6402.65	4306.93	2110.47	37
H21B	7240.49	6299.42	2592.25	37
H23	4958.42	8919.71	1382.24	41
H24	5024.17	11759.46	448.06	49
H25	6679.88	12618.02	157.36	59
H26	8258.42	10728.13	808.56	62
H27	8201.14	7917.17	1749.59	46

3) X-Ray Crystallographic Data for compound H-2

Structure deposited at the Cambridge Crystallographic Data Centre (**CCDC 1982118**)

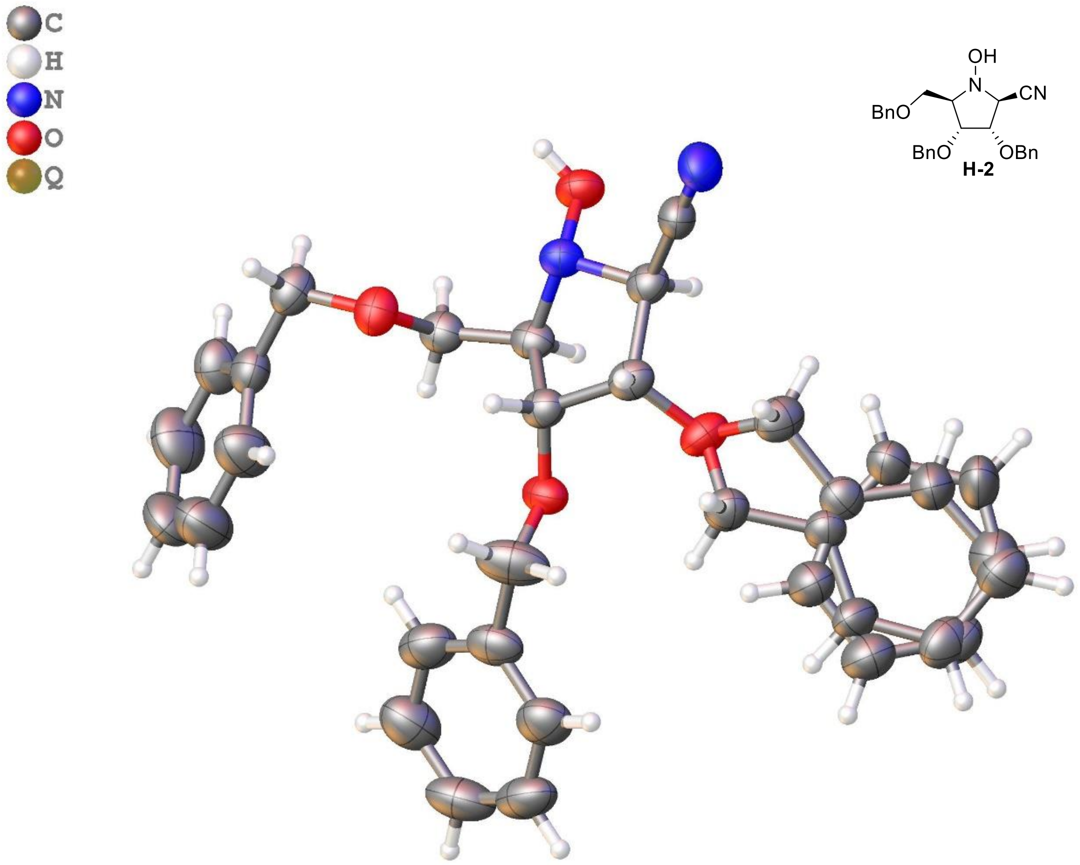

Fig1. X-Ray ellipsoid plots of **H-2**

Table 1 Crystal data and structure refinement for H-2.

Identification code	TX2156
Empirical formula	$C_{27}H_{28}N_2O_4$
Formula weight	444.51
Temperature/K	169.98(11)
Crystal system	orthorhombic
Space group	$P2_12_12_1$
a/Å	4.57238(10)
b/Å	21.9064(4)
c/Å	23.4943(6)
α/°	90
β/°	90
γ/°	90
Volume/Å^3	2353.29(9)
Z	4
ρ_{calc}g/cm^3	1.255

μ/mm^{-1}	0.681
F(000)	944.0
Crystal size/mm^3	0.15 × 0.05 × 0.05
Radiation	CuKα (λ = 1.54184)
2Θ range for data collection/°	5.516 to 151.634
Index ranges	-5 ≤ h ≤ 5, -26 ≤ k ≤ 26, -26 ≤ l ≤ 29
Reflections collected	27807
Independent reflections	4765 [R_{int} = 0.0712, R_{sigma} = 0.0364]
Data/restraints/parameters	4765/284/366
Goodness-of-fit on F^2	1.044
Final R indexes [I>=2σ (I)]	R_1 = 0.0481, wR_2 = 0.1161
Final R indexes [all data]	R_1 = 0.0575, wR_2 = 0.1207
Largest diff. peak/hole / e Å^{-3}	0.22/-0.16
Flack parameter	0.16(12)

Table 2 Fractional Atomic Coordinates (×10^4) and Equivalent Isotropic Displacement Parameters (Å^2 ×10^3) for H-2. U_{eq} is defined as 1/3 of of the trace of the orthogonalised U_{IJ} tensor.

Atom	*x*	*y*	*z*	U(eq)
O1	3878(4)	4235.9(8)	3602.0(7)	46.4(4)
O2	3635(4)	4806.7(8)	4621.0(8)	48.9(4)
O3	4866(5)	2855.3(9)	5100.8(8)	48.7(4)
O4	7981(4)	2766.2(8)	3694.7(8)	47.9(4)
N1	6221(5)	3323.5(9)	4761.6(8)	40.8(5)
N2	9213(7)	4050.5(12)	5912.9(10)	61.2(7)
C1	5764(5)	4001.2(11)	4020.9(10)	41.9(5)
C2	5977(5)	4391.4(11)	4573.1(11)	42.8(5)
C3	5680(5)	3906.0(11)	5050.6(10)	42.6(5)
C4	4618(5)	3388.9(11)	4223.5(10)	40.1(5)
C5	5225(9)	4615.1(19)	3199.3(15)	79.4(11)
C6	3220(7)	4694.8(15)	2700.0(13)	57.7(7)
C6AB	3806(15)	5389(2)	4415(3)	50.1(16)
C7	2019(9)	5252.4(16)	2576.7(15)	68.2(9)
C8	234(9)	5325.0(18)	2110.2(17)	77.0(11)
C9	-418(9)	4849(2)	1767.4(16)	81.6(11)
C10	767(11)	4286.7(19)	1883.6(16)	82.5(11)
C11	2587(9)	4214.2(17)	2350.9(16)	71.6(9)

C12	3977(13)	5213(2)	5058(2)	55.6(15)
C13	2050(20)	5764(4)	4964(4)	46.1(19)
C13A	1990(30)	5838(5)	4747(5)	44(2)
C14	2053(17)	6062(4)	4451(3)	63.3(17)
C14A	1490(20)	5766(4)	5323(4)	59(2)
C15	420(20)	6589(4)	4367(4)	71(2)
C15A	-130(20)	6187(4)	5625(4)	72(3)
C16	-1270(40)	6789(9)	4821(7)	63(3)
C16A	-1190(60)	6688(7)	5326(9)	72(3)
C17	-1230(50)	6536(7)	5349(8)	80(4)
C17A	-710(50)	6803(10)	4761(8)	65(4)
C18	460(20)	6011(4)	5402(4)	70(2)
C18A	780(20)	6343(4)	4478(4)	58(2)
C19	7674(6)	3984.7(11)	5535.9(11)	45.0(6)
C20	4993(5)	2858.0(12)	3829.6(11)	43.4(6)
C21	8371(6)	2210.5(12)	3388.2(12)	51.2(6)
C22	7048(6)	2225.0(12)	2803.6(12)	49.5(6)
C23	7599(10)	2712.7(16)	2444.2(16)	76.9(10)
C24	6480(13)	2728.3(19)	1910.1(17)	90.7(13)
C25	4754(11)	2262(2)	1714.0(15)	88.7(13)
C26	4131(11)	1777(2)	2066.7(16)	84.4(12)
C27	5293(8)	1761.5(14)	2609.3(13)	60.8(8)

Table 3 Anisotropic Displacement Parameters (Å^2 ×10^3) for H-2. The Anisotropic displacement factor exponent takes the form: $-2\pi^2[h^2a^{*2}U_{11}+2hka^*b^*U_{12}+…]$.

Atom	U_{11}	U_{22}	U_{33}	U_{23}	U_{13}	U_{12}
O1	41.3(9)	56.3(10)	41.5(9)	10.1(7)	0.6(7)	4.1(8)
O2	43.8(9)	38.3(9)	64.5(11)	0.7(8)	-8.6(9)	4.0(8)
O3	50.4(10)	44.0(10)	51.6(10)	11.5(8)	0.1(9)	-0.2(9)
O4	36.4(9)	50.8(10)	56.6(10)	-7.2(8)	-1.8(8)	-2.6(8)
N1	40.4(10)	40.4(10)	41.7(11)	5.2(8)	-2.4(9)	-2.0(9)
N2	69.0(17)	66.7(15)	48.0(13)	-10.4(11)	-6.0(13)	12.3(13)
C1	34.5(12)	44.3(12)	47.0(13)	7.0(10)	1.9(10)	1.6(10)
C2	34.7(11)	42.2(13)	51.6(14)	4.9(10)	-2.6(11)	-1.6(10)
C3	34.7(12)	44.7(13)	48.3(13)	3.4(10)	1.2(10)	3.4(10)
C4	30.4(11)	48.2(13)	41.8(12)	5.2(10)	-2.9(9)	-0.5(10)
C5	71(2)	92(3)	76(2)	39.2(19)	-13.6(19)	-28(2)
C6	50.5(15)	68.8(19)	54.0(16)	24.2(14)	3.1(13)	-7.4(14)
C6AB	54(3)	45(3)	52(3)	5(2)	4(3)	2(3)

C7	75(2)	65.0(19)	65.0(19)	16.7(15)	7.2(17)	-4.2(17)
C8	71(2)	77(2)	83(2)	36(2)	9(2)	15.7(19)
C9	77(2)	104(3)	64(2)	35(2)	-12.5(19)	-11(2)
C10	102(3)	83(2)	62.1(19)	6.1(17)	-2(2)	-14(2)
C11	73(2)	65.1(19)	76(2)	22.0(17)	4.6(18)	6.4(17)
C12	59(3)	47(3)	61(3)	-2(2)	-11(3)	1(2)
C13	51(3)	39(3)	48(5)	1(4)	1(4)	-5(2)
C13A	50(3)	36(4)	45(6)	5(4)	6(5)	0(3)
C14	69(4)	62(4)	59(4)	8(3)	5(3)	16(3)
C14A	77(5)	50(4)	50(5)	3(4)	9(4)	8(4)
C15	77(4)	63(5)	72(5)	10(4)	-2(4)	15(4)
C15A	96(5)	60(5)	60(5)	-6(4)	24(4)	16(4)
C16	63(6)	57(5)	70(6)	-3(4)	-5(4)	17(4)
C16A	86(6)	54(6)	74(6)	-1(5)	-2(5)	9(5)
C17	91(6)	78(8)	71(5)	-3(5)	8(5)	19(7)
C17A	74(8)	48(5)	74(7)	-9(5)	-10(5)	6(5)
C18	90(5)	70(5)	52(4)	0(4)	6(4)	24(4)
C18A	79(5)	45(5)	50(4)	1(4)	-6(4)	15(4)
C19	48.4(14)	45.5(13)	41.1(13)	-4.3(10)	3.5(11)	8.6(11)
C20	34.1(11)	49.8(14)	46.2(13)	3.5(11)	-1.7(10)	-3.2(11)
C21	45.4(14)	46.4(14)	61.8(16)	-7.0(12)	-4.1(13)	0.8(12)
C22	46.3(14)	48.7(15)	53.5(15)	-6.8(11)	4.1(12)	6.8(12)
C23	100(3)	64(2)	66(2)	4.3(15)	0(2)	-10.1(19)
C24	130(4)	81(2)	61(2)	5.7(18)	0(2)	4(3)
C25	113(3)	104(3)	48.5(18)	-10.8(19)	-14(2)	22(3)
C26	90(3)	97(3)	66(2)	-30(2)	-11(2)	-8(2)
C27	64.3(18)	57.2(16)	61.1(17)	-12.3(13)	2.3(15)	-7.0(14)

Table 4 Bond Lengths for H-2.

Atom	Atom	Length/Å	Atom	Atom	Length/Å
O1	C1	1.406(3)	C9	C10	1.373(6)
O1	C5	1.402(4)	C10	C11	1.387(6)
O2	C2	1.410(3)	C12	C13	1.511(8)
O2	C6AB	1.366(6)	C13	C14	1.372(8)
O2	C12	1.369(5)	C13	C18	1.370(10)
O3	N1	1.439(3)	C13A	C14A	1.382(9)
O4	C20	1.417(3)	C13A	C18A	1.388(10)
O4	C21	1.426(3)	C14	C15	1.389(8)
N1	C3	1.466(3)	C14A	C15A	1.380(9)

N1	C4	1.468(3)	C15	C16	1.388(11)
N2	C19	1.140(4)	C15A	C16A	1.391(11)
C1	C2	1.557(4)	C16	C17	1.357(11)
C1	C4	1.517(3)	C16A	C17A	1.368(12)
C2	C3	1.552(3)	C17	C18	1.393(11)
C3	C19	1.470(4)	C17A	C18A	1.387(12)
C4	C20	1.496(4)	C21	C22	1.501(4)
C5	C6	1.499(5)	C22	C23	1.385(4)
C6	C7	1.370(5)	C22	C27	1.372(4)
C6	C11	1.366(5)	C23	C24	1.356(6)
C6AB	C13A	1.505(9)	C24	C25	1.371(7)
C7	C8	1.376(6)	C25	C26	1.376(6)
C8	C9	1.350(6)	C26	C27	1.382(5)

Table 5 Bond Angles for H-2.

Atom	Atom	Atom	Angle/°	Atom	Atom	Atom	Angle/°
C5	O1	C1	114.8(2)	O2	C12	C13	110.1(5)
C6AB	O2	C2	122.0(3)	C14	C13	C12	120.5(8)
C12	O2	C2	113.1(3)	C18	C13	C12	121.0(8)
C20	O4	C21	110.79(19)	C18	C13	C14	118.2(8)
O3	N1	C3	106.93(18)	C14A	C13A	C6AB	121.6(9)
O3	N1	C4	109.36(18)	C14A	C13A	C18A	118.1(8)
C3	N1	C4	103.27(18)	C18A	C13A	C6AB	120.3(9)
O1	C1	C2	114.9(2)	C13	C14	C15	121.3(8)
O1	C1	C4	109.3(2)	C15A	C14A	C13A	121.1(9)
C4	C1	C2	104.22(19)	C16	C15	C14	116.9(11)
O2	C2	C1	111.9(2)	C14A	C15A	C16A	117.0(12)
O2	C2	C3	108.5(2)	C17	C16	C15	124.4(17)
C3	C2	C1	102.76(19)	C17A	C16A	C15A	125(2)
N1	C3	C2	104.29(19)	C16	C17	C18	115.3(16)
N1	C3	C19	110.89(19)	C16A	C17A	C18A	114(2)
C19	C3	C2	115.2(2)	C13	C18	C17	123.5(10)
N1	C4	C1	100.60(18)	C17A	C18A	C13A	123.8(12)
N1	C4	C20	113.6(2)	N2	C19	C3	179.4(3)
C20	C4	C1	117.0(2)	O4	C20	C4	111.0(2)
O1	C5	C6	109.2(3)	O4	C21	C22	113.2(2)
C7	C6	C5	121.0(3)	C23	C22	C21	120.1(3)
C11	C6	C5	120.7(3)	C27	C22	C21	121.6(3)
C11	C6	C7	118.4(3)	C27	C22	C23	118.3(3)

O2	C6AB	C13A	113.3(6)	C24	C23	C22	121.0(4)
C6	C7	C8	120.6(3)	C23	C24	C25	120.6(4)
C9	C8	C7	121.1(3)	C24	C25	C26	119.4(3)
C8	C9	C10	119.1(3)	C25	C26	C27	119.7(4)
C9	C10	C11	119.8(4)	C22	C27	C26	120.9(3)
C6	C11	C10	121.0(3)				

Table 6 Hydrogen Atom Coordinates (Å×10^4) and Isotropic Displacement Parameters (Å^2×10^3) for H-2.

Atom	*x*	*y*	*z*	U(eq)
H1	7722.81	3947.91	3858.84	50
H2	7863.05	4602.27	4596.19	51
H3A	3662.29	3909.23	5191.35	51
H4	2532.04	3432.38	4310.36	48
H5A	7053.26	4434.21	3075.32	95
H5B	5650.64	5009.22	3368.31	95
H6AA	3162.85	5390.38	4021.58	60
H6AB	5832.49	5519.24	4422.08	60
H7	2412.93	5584.81	2810.67	82
H8	-536.17	5708.26	2029.51	92
H9	-1652.23	4901.9	1456.67	98
H10	348.89	3955.52	1649.72	99
H11	3388.18	3832.75	2427.65	86
H12A	6005.94	5340.74	5079.8	67
H12B	3466.16	5018.75	5415.49	67
H14	3175.98	5909.22	4153.57	76
H14A	2260.74	5428.3	5510.3	70
H15	448.96	6797.54	4022.43	85
H15A	-501.42	6137.2	6011.76	86
H16	-2519.89	7117.79	4759.87	76
H16A	-2312.3	6969.08	5526.31	86
H17	-2263.71	6702.23	5652.98	96
H17A	-1333	7158.42	4582.5	78
H18	519	5817.05	5754.43	84
H18A	989.44	6375.08	4085.37	70
H20A	4215.47	2492.85	4007.48	52
H20B	3898.69	2931.13	3482.47	52
H21A	7494.59	1879.28	3602.64	61
H21B	10447.34	2126.81	3354.9	61

H23	8754.07	3034.41	2571.26	92
H24	6886.95	3058.62	1674.42	109
H25	4009.26	2272.96	1345.79	106
H26	2933.79	1462.12	1940.23	101
H27	4880.46	1432.29	2846.02	73
H3	6000(100)	2567(19)	5130(16)	77(12)

Table 7 Atomic Occupancy for H-2.

**Atom**	***Occupancy***	**Atom**	***Occupancy***	**Atom**	***Occupancy***
C6AB	0.456(6)	H6AA	0.456(6)	H6AB	0.456(6)
C12	0.544(6)	H12A	0.544(6)	H12B	0.544(6)
C13	0.544(6)	C13A	0.456(6)	C14	0.544(6)
H14	0.544(6)	C14A	0.456(6)	H14A	0.456(6)
C15	0.544(6)	H15	0.544(6)	C15A	0.456(6)
H15A	0.456(6)	C16	0.544(6)	H16	0.544(6)
C16A	0.456(6)	H16A	0.456(6)	C17	0.544(6)
H17	0.544(6)	C17A	0.456(6)	H17A	0.456(6)
C18	0.544(6)	H18	0.544(6)	C18A	0.456(6)
H18A	0.456(6)				
